# Supplementary material for: Mineralocorticoid receptor knockout alters hippocampal CA2 neurons to become like those in CA1
Source: Commun Biol. 2025 Jul 10;8:1037. doi: 10.1038/s42003-025-08378-0 (PMC12246475; doi:10.1038/s42003-025-08378-0)
Supplement: Supplementary file 2 — Supplementary Information [file 42003_2025_8378_MOESM2_ESM.pdf]

Supplementary Data Fig. 1

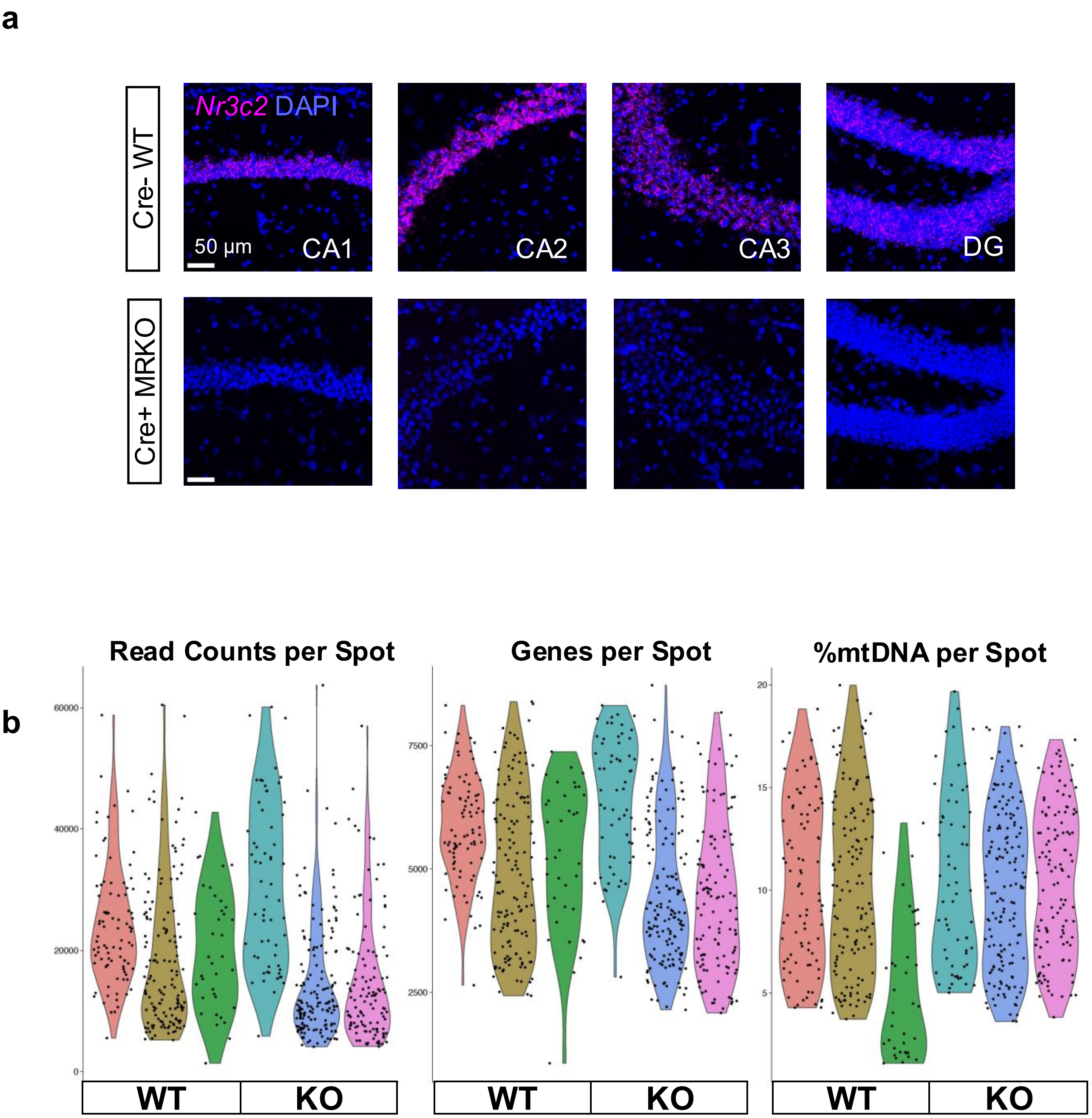

Supplementary Data Fig. 1. **a.** smFISH for MR (*Nr3c2*) to validate hippocampal knockout of MR using the in Emx1 Cre: MR fl/fl mice. Scale bar = 50 µm. **b.** Quality control measures in spots from the hippocampal cluster. Distribution of hippocampal read counts per spot, number of genes per spot, and percentage of mitochondrial DNA per spot are shown, with each color representing an animal (3 WT and 3 KO).

Supplementary Data Fig. 2

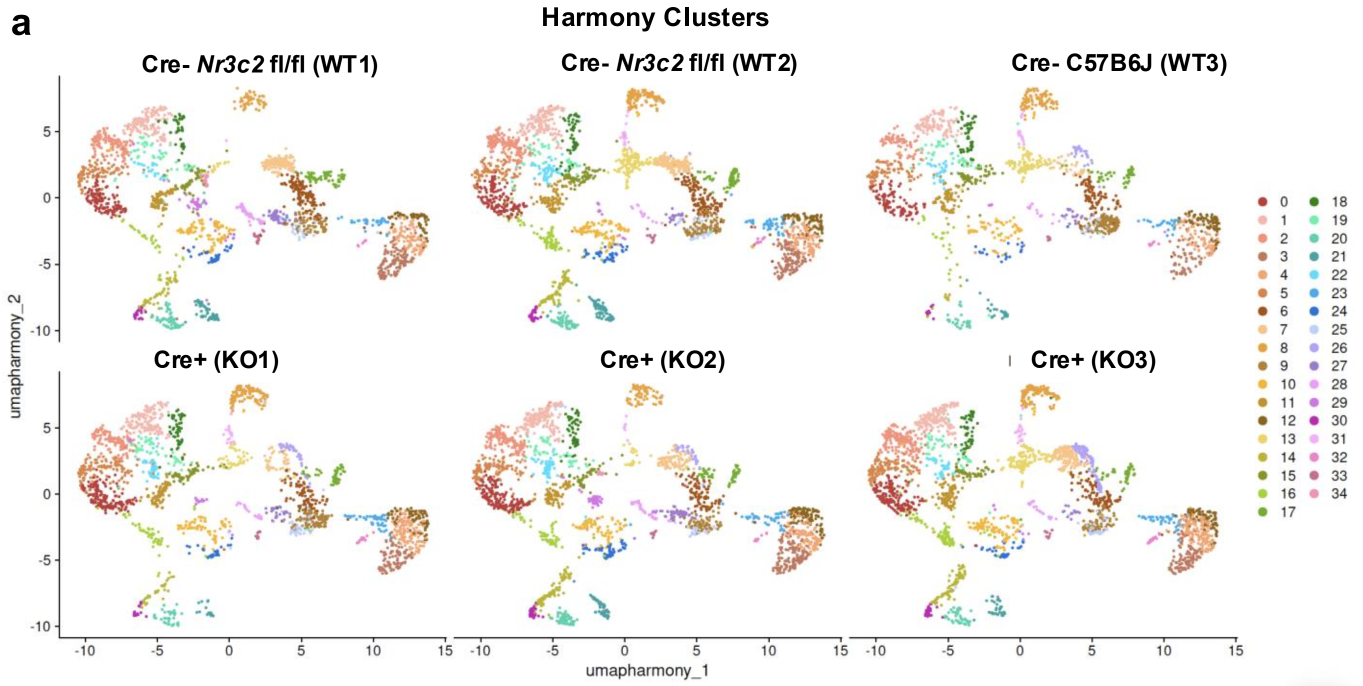

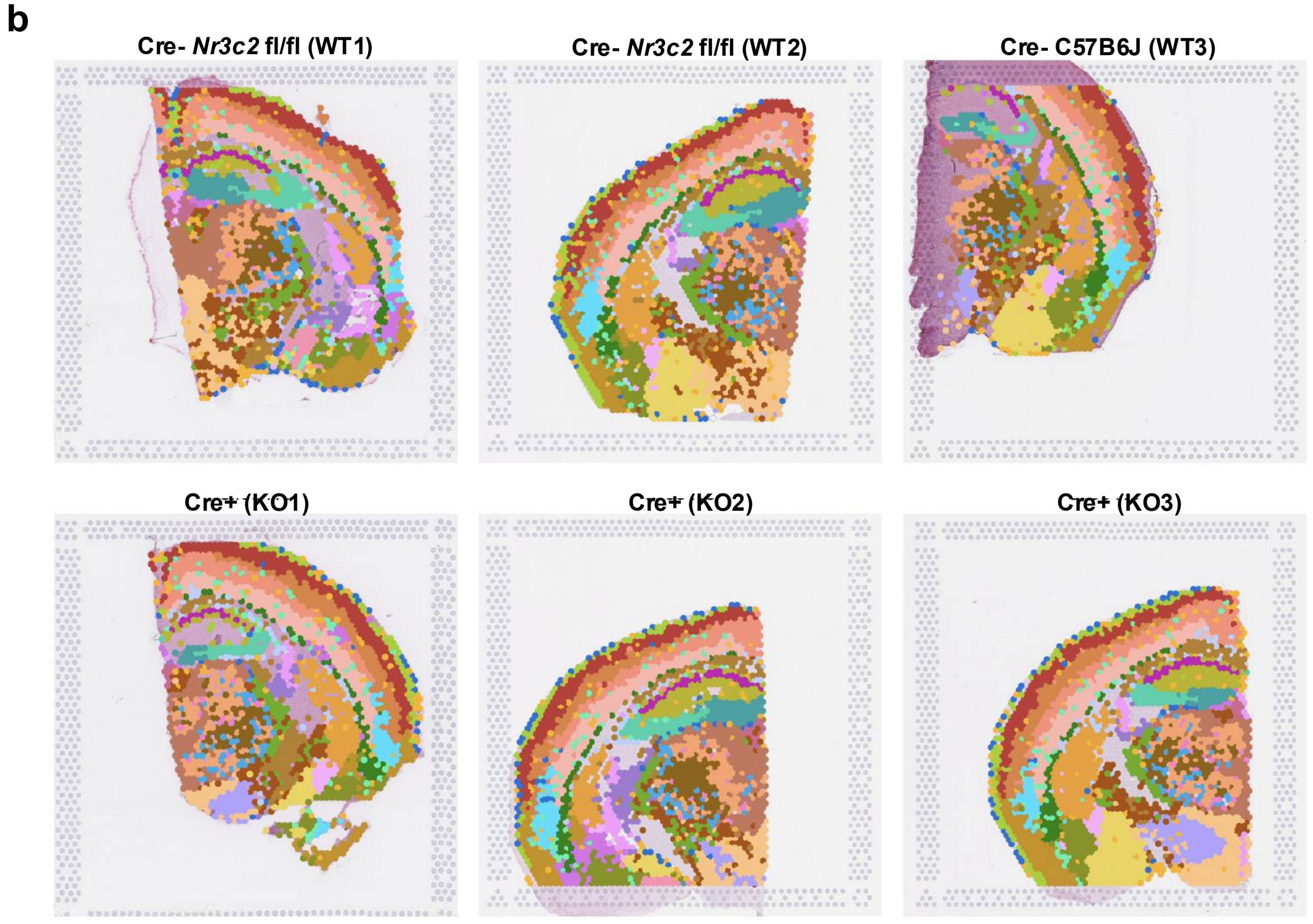

Supplementary Data Fig. 2 (continued)

c

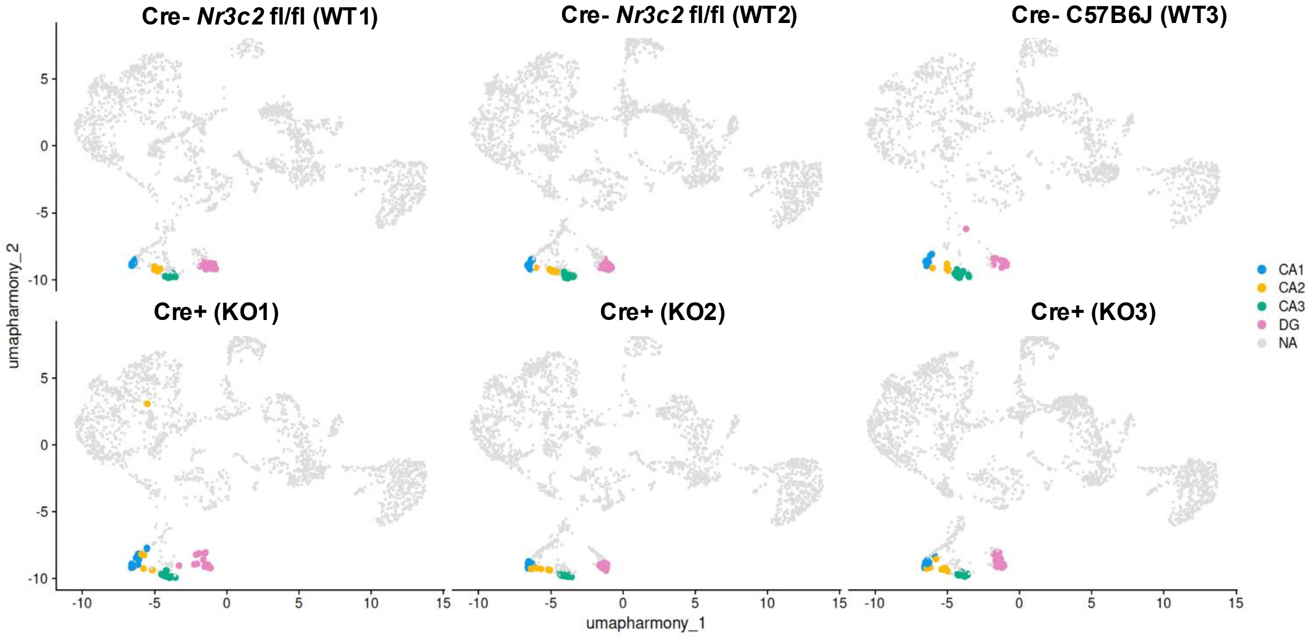

d

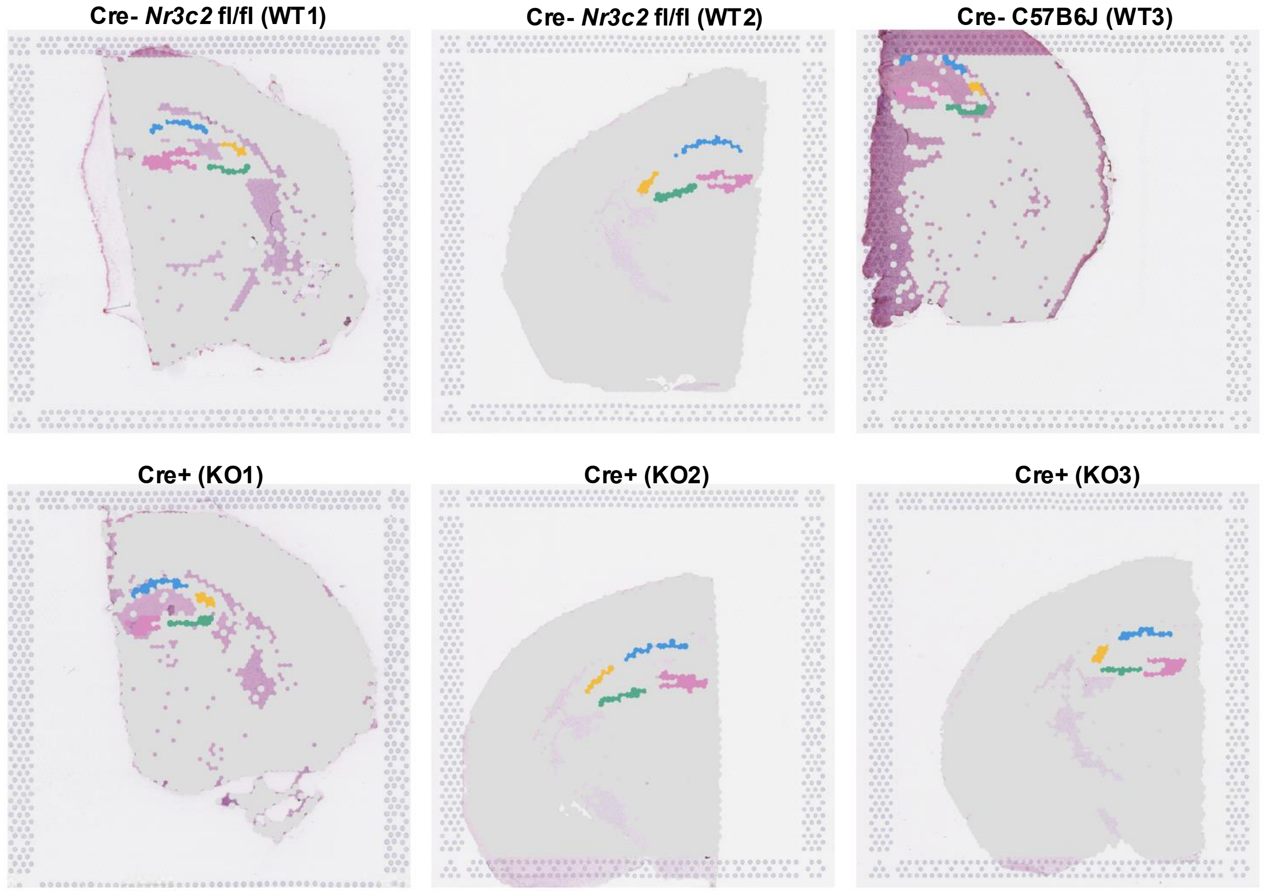

Supplementary Data Fig. 2. **a**. Projection maps were created using gene expression across six brain samples following Seurat with Harmony layer integration. Spots are colored by the 35 clusters defined using Seurat shared nearest-neighbor clustering. Wildltype (WT) samples are labeled WT1, WT2, and WT3. Mineralocorticoid receptor knockout (MR KO) samples are labeled KO1, KO2, and KO3. Each brain demonstrates consistent clustering patterns. The consistent set of clusters were represented across all WT and MR KO replicates, with no indication of batch effect, and with clear separation of hippocampus samples from other brain regions. The automated clustering also reinforced the expert annotations of hippocampus regions with the exception of CA2. **b.** Spatial maps showing six brain samples colored by cluster as (a). Spatial clusters are also consistently defined across all samples. **c.** Projection maps as in (a) colored by manually selected regions, CA1 (blue), CA2 (gold), CA3 (green), and DG (pink). **d.** Spatial maps as in (b) except colored by according to manually selected regions as in (c). The manually selected regions based on location in the stratum *pyramidale* were used for all subsequent analyses.

Supplementary Data Fig. 3

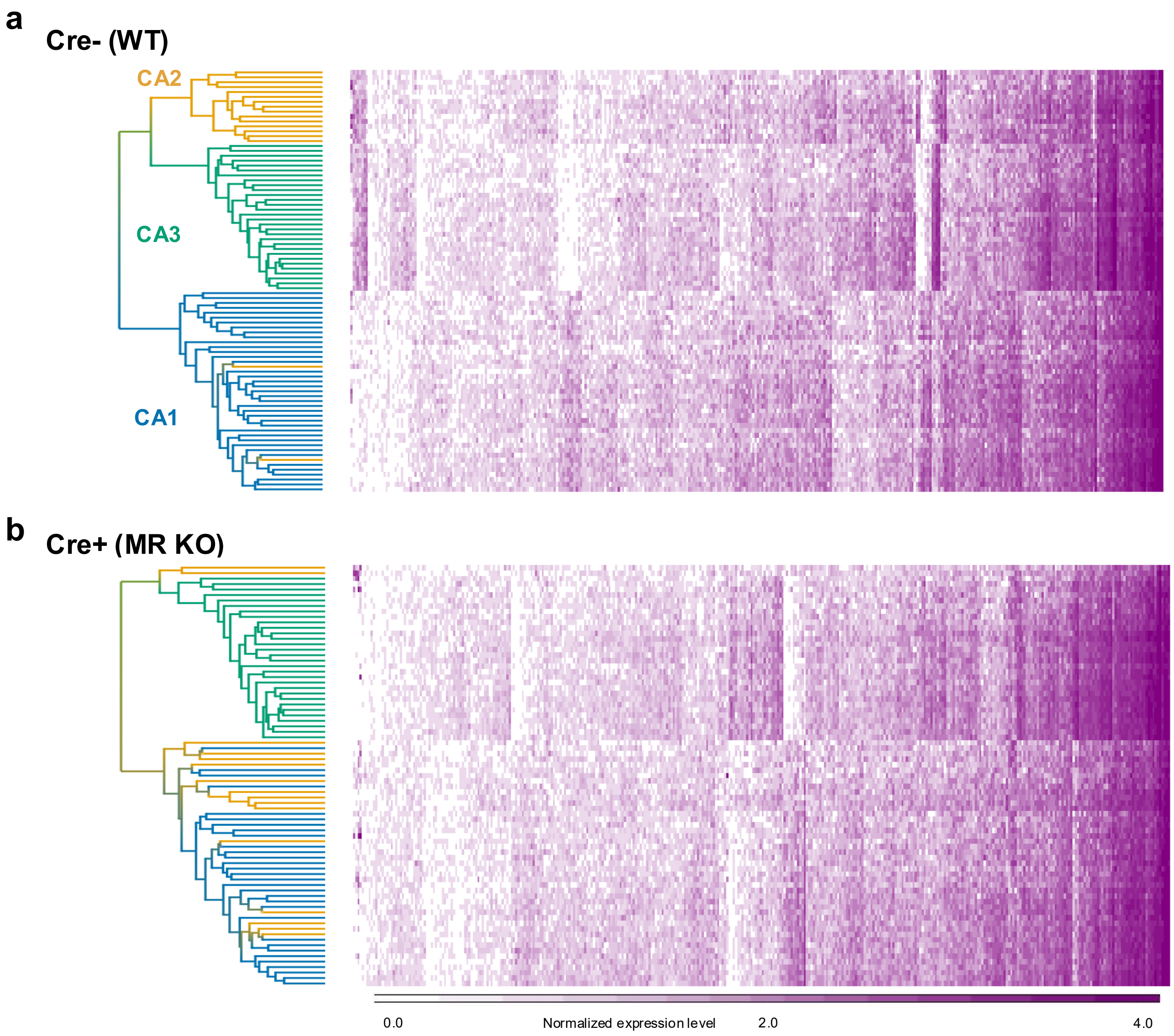

Supplementary Data Fig. 3. a. Heatmaps with normalized expression (in log10 space) with 368 hippocampal genes (columns) and spots (rows) from WT animals. Dendrograms indicate the hierarchical clustering of spots with similar expression profiles, color-coded according to the spot location. Expression levels are shown as a gradient from white to purple, where expression level zero is white, and high expression is represented by dark purple. “Hippocampal genes” are defined as overexpressed genes in the hippocampal area spots in comparison with the spots in the rest of the areas using a cutoff of FC > 1.2 and adjusted $p < 0.05$. **b.** Same as in (a) except for samples from MR KO mice.

Supplementary Data Fig. 4

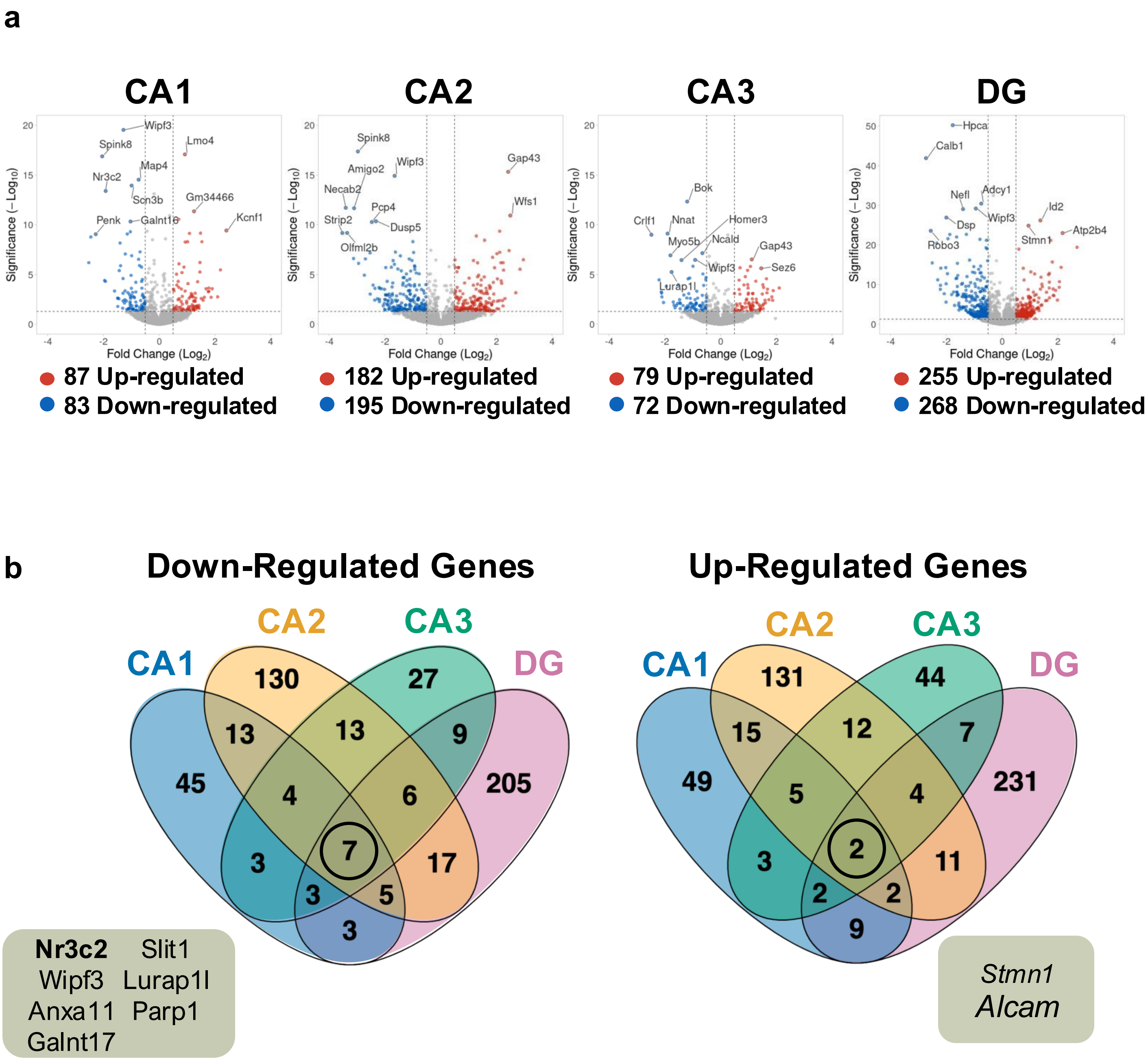

Supplementary Data Fig. 4. a. Volcano plots showing differentially expressed genes between Cre- WT and Cre+ MR KO in CA1, CA2, CA3, and DG. Differentially expressed genes with FDR <0.05 (-log10 = 1.3) and log2 fold-change values greater than 0.585 or less than -0.585 are indicated by color: significantly up-regulated in red and down-regulated in blue. The top 10 genes by Manhattan distance in each region are labelled, e.g., *Nr3c2, Wfs1, Crlf1* and *Calb1*. **b.** Venn diagrams indicate minimal overlap of DEGs between hippocampal regions. We found 130 down-regulated genes and 131 up-regulated genes that were unique to area CA2. DEGs common to all 4 regions are listed below each diagram. Notably, *Nr3c2* is represented in the down-regulated genes in all regions, consistent with the gene knockout.

Supplementary Data Fig. 5

a

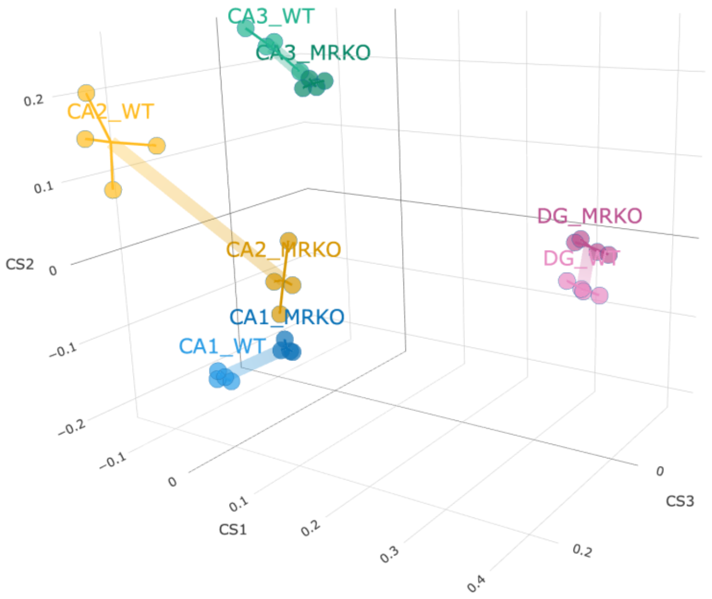

b

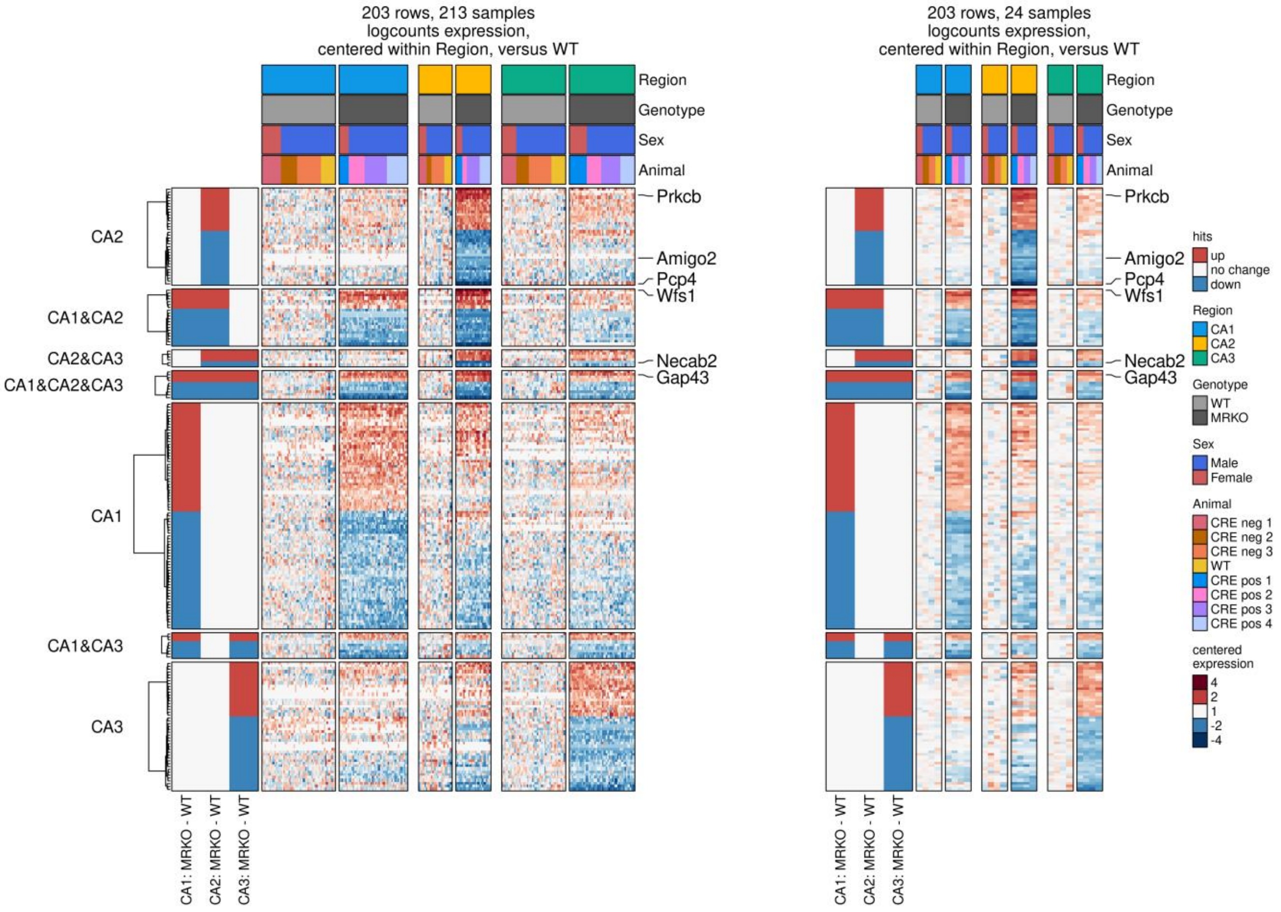

Supplementary Data Fig. 5. a. Principal component analysis (PCA) including male and female mice shows consistency across sexes within experimental groups, and a shift in transcriptional profile of CA2 Cre+ MR KO mice towards CA1, compared with Cre- mice. In MR KO, the correlation between CA1 and CA2 is enhanced, while the correlation between CA3 and CA2 remains unchanged. n = 8 (includes 3 male, 1 female WT mice and 3 male, 1 female MR KO). **b.** Heatmaps depicting the expression levels of MR KO and wildtype (WT) mice across the CA1, CA2, and CA3 hippocampal regions. Log-transformed counts were normalized as descried, then presented by both by spot (left) and by animal (right). Statistical significance for comparisons between regions is indicated ($p<0.05$, n = 4 WT, 4 MR KO). Relative expression was calculated by log-transforming the raw counts, followed by centering the data to the mean expression of wildtype samples within each region. This normalization approach isolates the differential expression of MR KO relative to wildtype in each specific hippocampal subregion. n = 8 (includes 3 male, 1 female WT mice and 3 male, 1 female MR KO).

Supplementary Data Fig. 6

a Top 20 Pathways in **CA2** impacted by MR KO

Ingenuity pathway analysis (IPA)

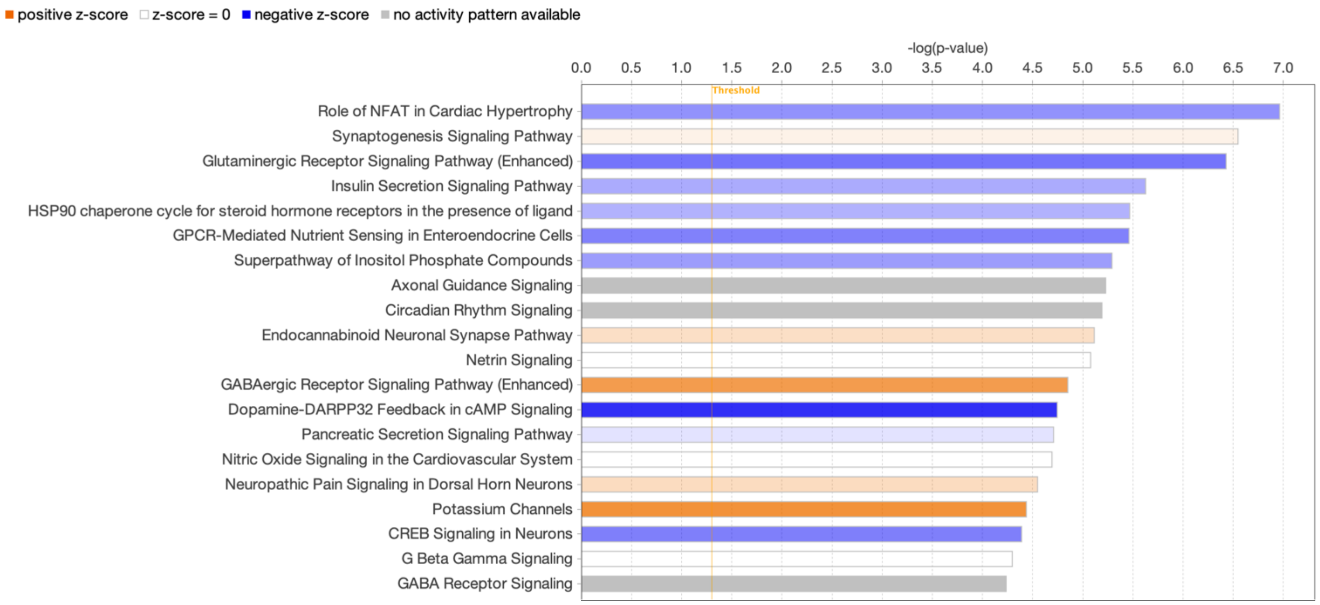

b Upstream regulators affected by MR KO in CA2

Ingenuity pathway analysis (IPA)

**Upstream Regulator**	**Molecule Type**	**Predicted Activation State**	**Activation z-score**	**p-value of overlap**	**Target Molecules in Dataset**
**IGF1R**	transmembrane receptor	Activated	2.000	0.274	CABP1,ETV1,FHL2,IGFBP4,Scd2, SCG2
**RXRA**	ligand-dependent nuclear receptor	Activated	2.000	0.561	FLRT3,PLCL1,TGFB2,TMEM215
**INSR**	kinase	Activated	2.219	0.523	ACOT13,ATP2B1,ETV1,FHL2,IGF1R, KIT,LDB2
**DNMT3B**	enzyme	Activated	2.000	0.056	ADCY5,ADORA1,CACNA1C,PRKCB
**TRAPPC1**	other	Inhibited	-2.000	0.082	HSPA1A,Hspa1b,HSPA2,LMAN2
**PRKAA1**	kinase	Inhibited	-2.828	0.001	GRIA3,Gsta4,IGFBP4,LMO4,NAP1L5, SERPINI1,SMAD3,TTYH1
**PRKAA2**	kinase	Inhibited	-2.828	0.001	GRIA3,Gsta4,IGFBP4,LMO4,NAP1L5, SERPINI1,SMAD3,TTYH1
**ESR2**	ligand-dependent nuclear receptor	Inhibited	-2.646	0.113	ARNT2,C3orf80,CABP1,CADM3, CBFA2T3,FKBP5,PTGS2
**NOTCH3**	transcription regulator	Inhibited	-2.200	0.029	CACNA1C,CCN3,ERRFI1,HSPA1A, Hspa1b
**CYP1B1**	enzyme	Inhibited	-2.000	0.131	ARNT2,C11orf87,CACHD1,FLRT3

CA2 up in MR KO/down in MR KO

Supplementary Data Fig. 6 (continued)

c Synaptic location and function of **CA2** genes impacted by MR KO

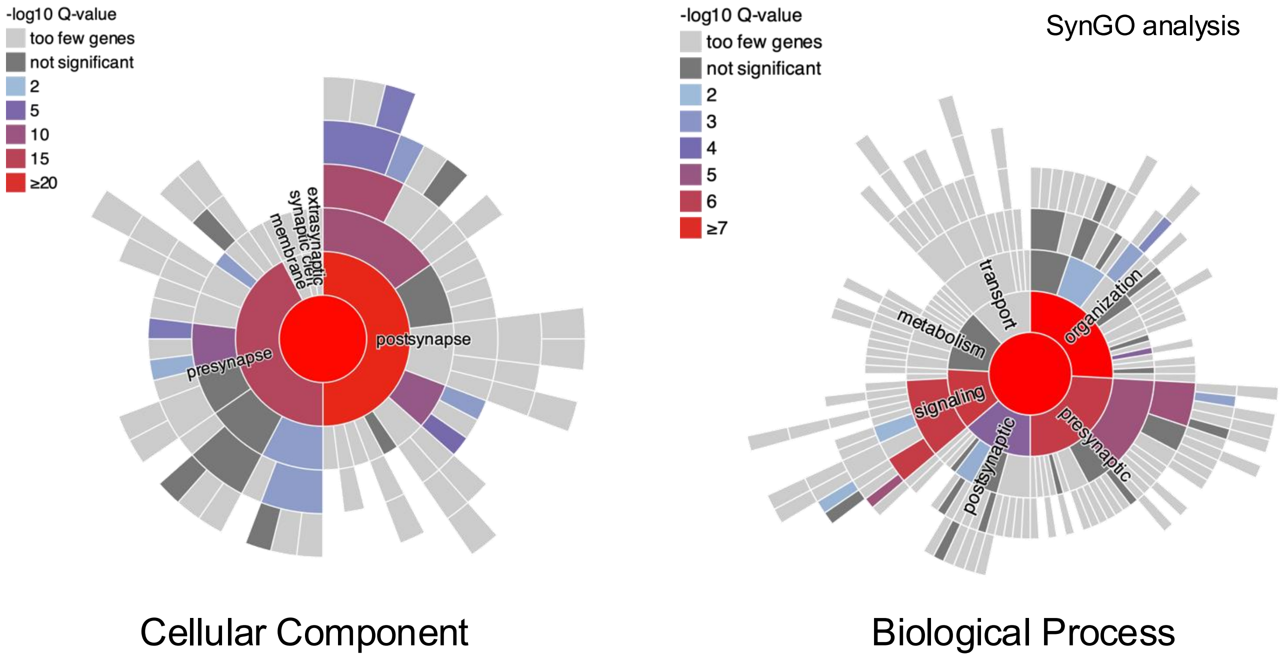

Supplementary Data Fig. 6. **a**. Bar chart showing Ingenuity Pathway Analysis (IPA) canonical pathway enrichment results using differentially expressed genes between Cre- WT and Cre+ MR KO in CA2 with log fold-change values greater than 0.5 or less than -0.5 and FDR <0.05. Among the top 20 pathways ranked by p-value, the GABAergic receptor signaling and potassium channels pathways are predicted to be activated/increased (orange), while the Dopamine-DARPP32 feedback in cAMP signaling pathway is predicted to be inactivated/decreased (blue). The white bars representing netrin, nitric oxide and G Beta Gamma Signaling pathways indicated no clear signal for prediction (z-score = 0). The gray bars representing circadian rhythm, GABA receptor signaling and axon guidance pathways indicated that there is no activity pattern available identified in IPA, despite the highly significant association of the genes within these pathways. **b.** The upstream regulator analysis predicts activation of IGF1R, RXRA, INSR, and DNMT3B (z-scores > 2.000), and inactivation of DNMT3B, TRAPPC1, PRKAA1, PRKAA2, ESR2, NOTCH3, and CYP1B1 (z-scores < 2). Although activation of these regulators is suggested, the high p-value (0.274) for the overlap indicates that this prediction may not be statistically significant. In contrast, the inactivation of PRKAA1, PRKAA2, and NOTCH3 is supported by statistically significant p-values (< 0.05).
c. Sunburst plots depict the hierarchical distribution of cellular components (left) and biological processes (right) of the DEGs at the synapse. The central circle represents the root node, with the hierarchy expanding outward. Segments of the inner circle have a direct hierarchical relationship to corresponding segments in the outer circle, which are positioned within the angular sweep of the parent segment. 135 genes were mapped to SynGO-annotated genes, including 130 genes related to various cellular components and 95 genes associated with distinct biological processes (noting that genes may be annotated to multiple categories). A total of 17 cellular component terms and 18 biological process terms exhibited significant enrichment at a 1% FDR, based on the inclusion of at least three matching input genes. Enrichment was assessed using one-sided Fisher's exact tests (with the alternative hypothesis specifying greater than), and the FDR method was applied to adjust for multiple comparisons. See Supplementary Table 3.

Supplementary Data Fig. 7

a

Top 20 Pathways affected by MR KO in all regions

Ingenuity pathway analysis (IPA)

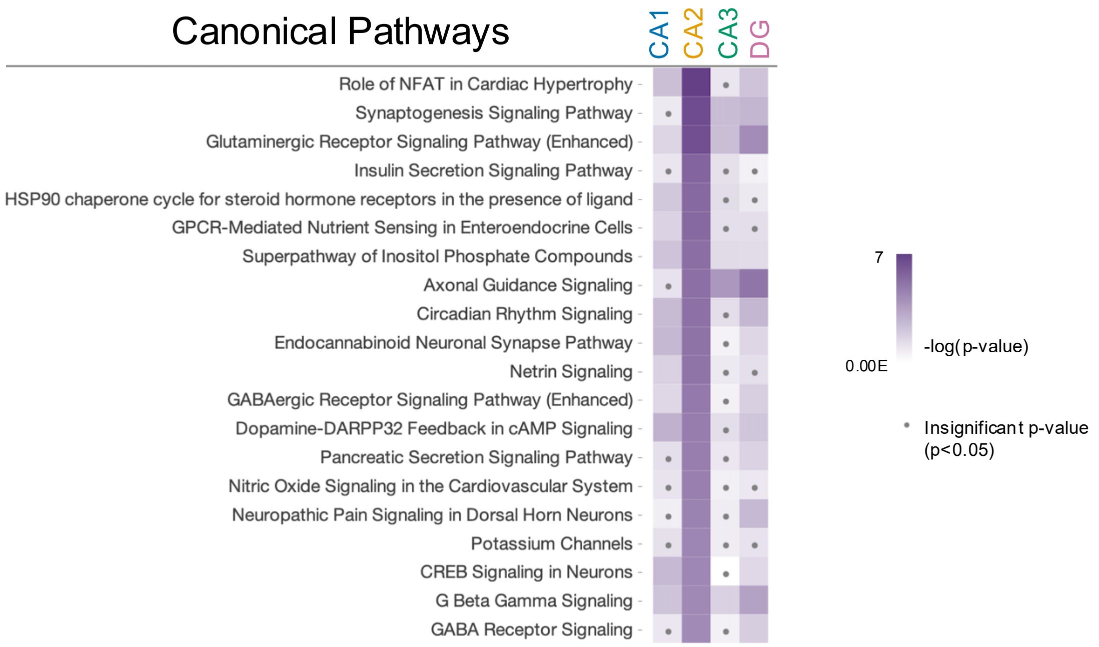

b

Upstream regulators affected by MR KO

Ingenuity pathway analysis (IPA)

Region	Upstream Regulator	Molecule Type	Predicted Activation State	Activation z-score	p-value of overlap	Target Molecules in Dataset
DG	TGFBR2	kinase	Activated	2.360	0.002	ADCY1,BHLHE40,Cd200,DDIT4,DUSP14,EPOP,GPLD1,IRS2,PGM1,SV2C
CA2	PRKAA1	kinase	Inhibited	-2.828	0.001	GRIA3,Gsta4,IGFBP4,LMO4,NAP1L5,SERPINI1,SMAD3,TTYH1
CA2	PRKAA2	kinase	Inhibited	-2.828	0.001	GRIA3,Gsta4,IGFBP4,LMO4,NAP1L5,SERPINI1,SMAD3,TTYH1
CA2	NOTCH3	transcription regulator	Inhibited	-2.200	0.029	CACNA1C,CCN3,ERRFI1,HSPA1A,Hspa1b
DG	SLCO1C1	transporter	Inhibited	-2.360	0.000	ATP1B2,ATP2B4,Cd200,CKMT1B,FXYD6,GAD1,NEFL,PITPNM1,RASAL1,SREBF2
DG	MEN1	transcription regulator	Inhibited	-2.095	0.000	ELAVL3,FABP7,FKBP5,GFAP,IRS2,NOS1,NRSN1,PTN,SLIT2,SOX11
DG	CREM	transcription regulator	Inhibited	-2.236	0.015	BCL6,BHLHE40,CEBPB,DUSP14,ERRFI1,IRS2,PDYN
DG	CREB1	transcription regulator	Inhibited	-2.236	0.035	BDNF,BHLHE40,CEBPB,DUSP14,ERRFI1,IRS2
DG	PIAS1	transcription regulator	Inhibited	-2.000	0.033	BDNF,GABRA5,GRM2,UNC13C
DG	SLC16A2	transporter	Inhibited	-2.714	0.000	ATP1B2,ATP2B4,Cd200,CKMT1B,FXYD6,GAD1,NEFL,PITPNM1,RASAL1,SREBF2
DG	DIO2	enzyme	Inhibited	-2.630	0.021	Arxes1/Arxes2,CARMIL1,CYTH3,DDIT4,MARCKSL1,NEFM,PDP1,PENK,SEMA7A

Supplementary Data Fig. 7. a. Heatmap view of the top 20 pathways affected by MR KO in CA2, showing 15 of the top 20 pathways were not significant in CA3, but were significant in CA1 and/or DG. Pathways such as glutamatergic receptor signaling, G beta gamma signaling, and the superpathway of inositol phosphate compounds were significant in all regions, with the superpathway of inositol phosphate compounds showing statistically significant inhibition specifically in CA1 and DG. **b.** Table with IPA predicted upstream regulators using DEGs from each region, sorted by activation z-score. No activators or inhibitors were predicted for the CA1 or CA3 regions. In the DG region, significant results predicted inhibition by SLCO1C1, MEN1, CREM, CREB1, PIAS1, SLC16A2, and DIO2, while TGFBR2 was predicted as an activator. See Supplementary Table 2.

Supplementary Data Fig. 8

a Top 20 Pathways in **CA1** impacted by MR KO

Ingenuity pathway analysis (IPA)

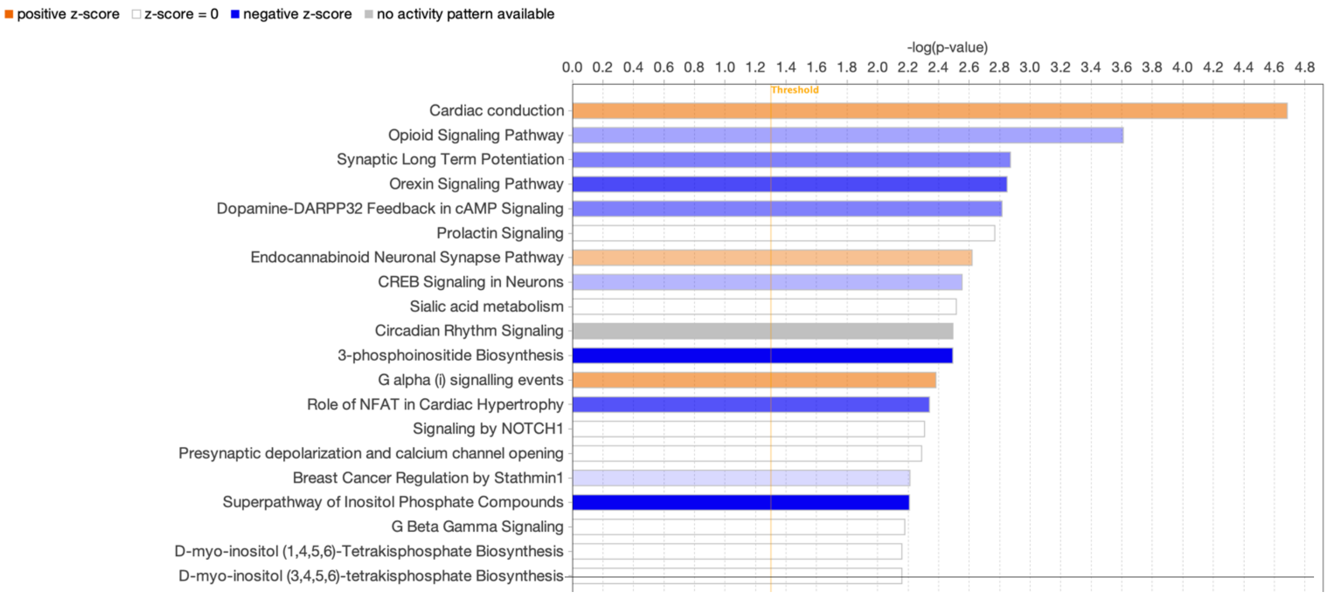

b Synaptic location and function of **CA1** genes impacted by MR KO

SynGO analysis

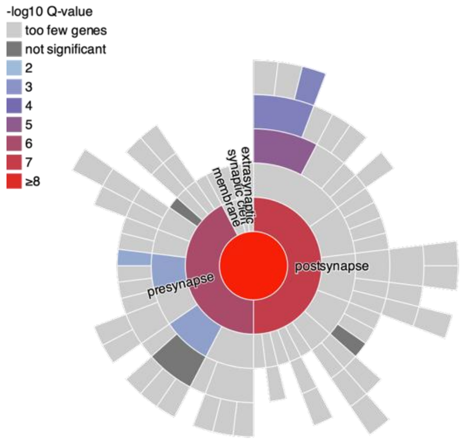

Cellular Component

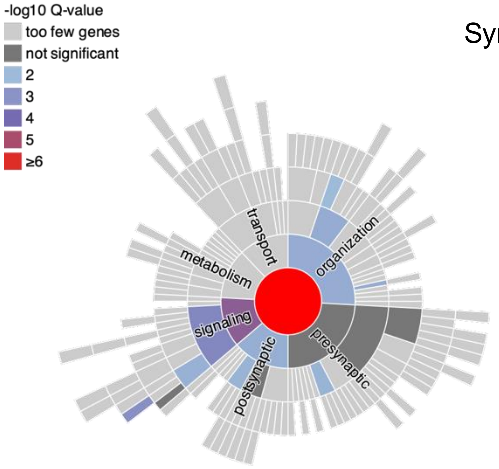

Biological Process

Supplementary Data Fig. 8 (continued)

c Top 20 Pathways in **CA3** impacted by MR KO

Ingenuity pathway analysis (IPA)

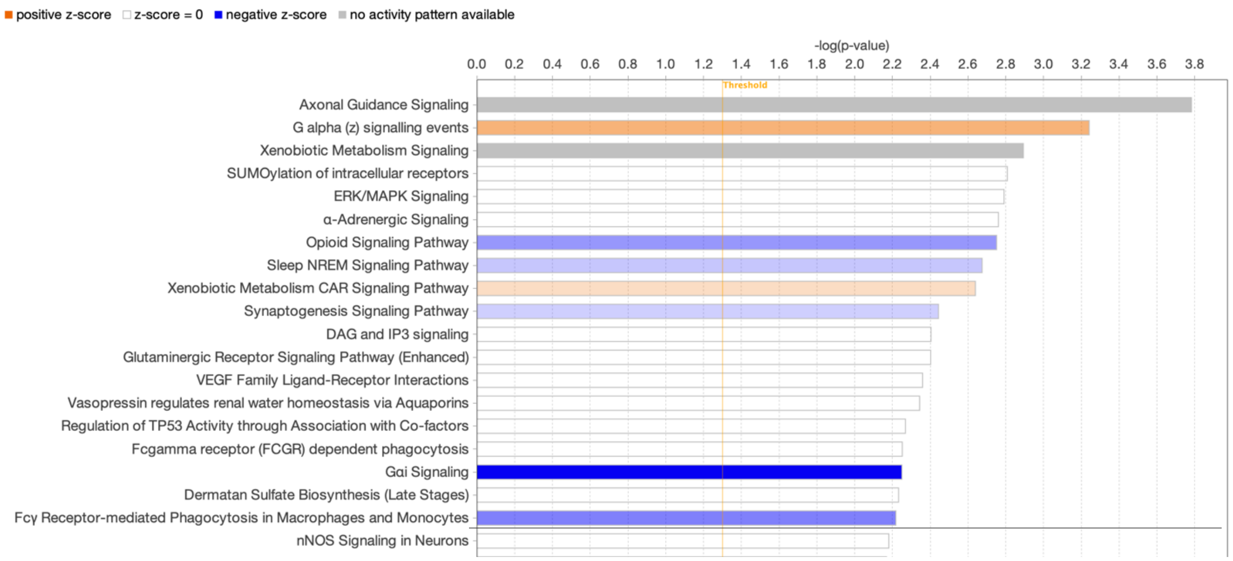

d Synaptic location and function of **CA3** genes impacted by MR KO

SynGO analysis

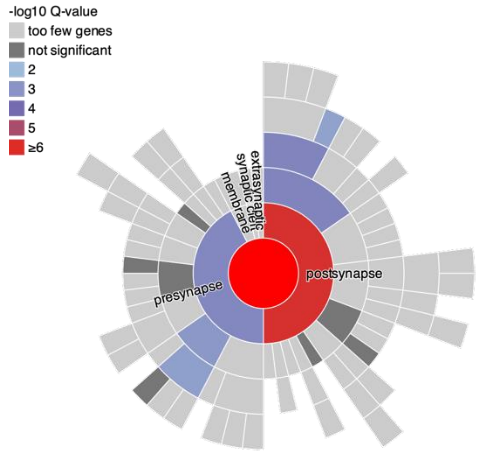

Cellular Component

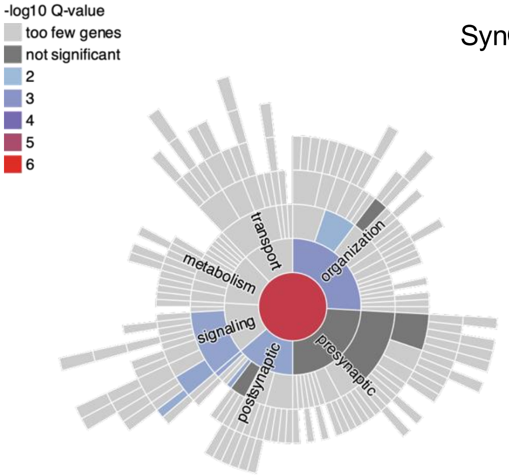

Biological Process

Supplementary Data Fig. 8 (continued)

e Top 20 Pathways in **DG** impacted by MR KO

Ingenuity pathway analysis (IPA)

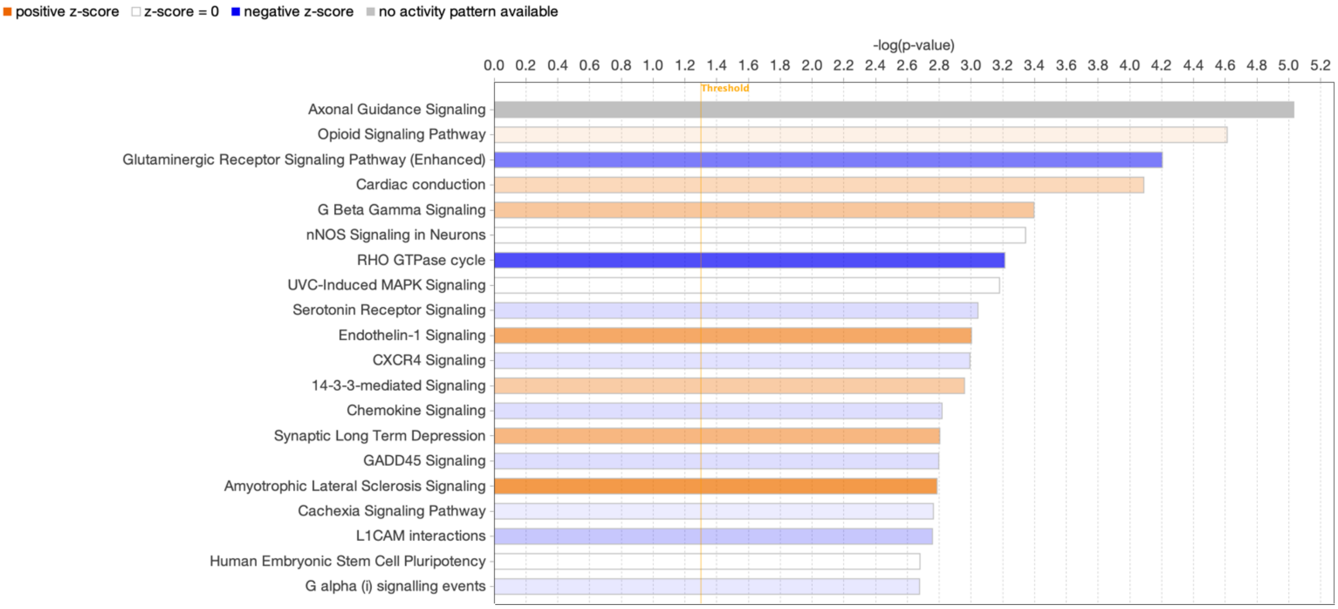

f Synaptic location and function of **DG** genes impacted by MR KO

SynGO analysis

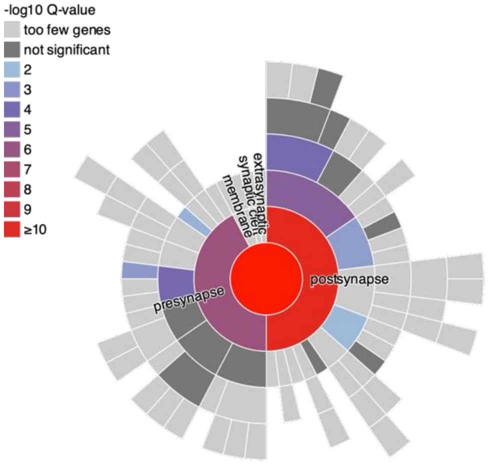

Cellular Component

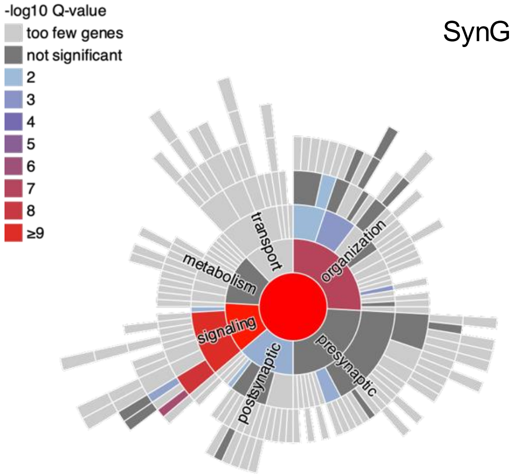

Biological Process

Supplementary Data Fig. 8. IPA canonical pathway enrichment results as shown in Supplementary Data Fig. 6 a, using differentially expressed genes between Cre- WT and Cre+ MR KO in CA1 **(a, b)**, CA3 **(c, d)** and DG **(e, f)** with log fold-change values greater than 0.5 or less than -0.5 and FDR <0.05. Bar charts (a, c, e) show the top 20 pathways ranked by p-value, where orange bars indicate predicted activation, blue bars show predicted inactivation, white indicates no clear signal (z-score 0), and gray indicates no activity pattern available in IPA. As the magnitude of the z-score (both positive and negative) increases, the colors in the plot transition from light to dark. Sunburst plots (b, d, f) as shown in Supplementary Data Fig. 6 c.

Supplementary Table 1- Hippocampal Genes

defined as genes overexpressed in the spots in the hippocampus compared with the spots in the other areas

using cutoff FC > 1.2 and adjusted p value < 0.05

**Gene**	**Log2FC**	**p_val_adj**
Hpca	1.588	1.29E-207
Neurod6	1.319	1.04E-266
Wipf3	1.066	1.90E-152
Prkcg	1.044	3.10E-151
Wfs1	1.029	6.98E-69
Cpne6	1.020	2.25E-144
Cnih2	0.985	2.86E-209
Cabp7	0.968	4.68E-299
Crym	0.966	3.26E-164
Mmd	0.943	5.70E-140
Lpl	0.937	4.88E-268
Zbtb18	0.910	3.59E-142
Chgb	0.899	1.53E-81
Gabra5	0.895	7.23E-202
Ptk2b	0.875	8.77E-143
St6galnac5	0.862	2.06E-182
Gria1	0.831	1.41E-147
Epha7	0.830	6.62E-172
Atp2b1	0.829	9.17E-115
Rasgrp1	0.827	3.12E-123
Ppp3ca	0.827	3.81E-153
Dkk3	0.826	3.33E-133
Fam131a	0.794	3.33E-109
Ddn	0.784	2.73E-156
Epha4	0.758	5.92E-118
Arhgef25	0.722	3.89E-132
Sptbn2	0.717	2.71E-150
Nr4a3	0.716	3.70E-187
Snca	0.707	7.66E-77
Wasf1	0.700	5.24E-111
Dgkz	0.699	1.95E-135
Prkca	0.698	1.47E-109
Dock4	0.696	7.60E-116
Tmsb4x	0.688	1.84E-115
Igfbp4	0.687	3.20E-56
Pou3f1	0.684	4.15E-103
Rprml	0.679	1.14E-87
Lmo3	0.678	1.29E-96
Cnksr2	0.677	1.02E-99
Grin2a	0.676	3.32E-91

Chn1	0.670	5.71E-109					
Spink8	0.669	7.23E-196					
Enc1	0.669	2.83E-94					
Arpc5	0.659	3.43E-88					
Nell2	0.659	1.27E-92					
Plppr4	0.656	2.01E-83					
Ppp3r1	0.642	7.30E-93					
Scn3b	0.642	1.18E-88					
Itpka	0.635	8.55E-57					
Neurod2	0.630	8.69E-138					
Klk8	0.629	0					
Fibcd1	0.628	1.30E-185					
Plk2	0.620	1.17E-73					
2010300C02Rik	0.620	1.02E-54					
Rgs14	0.620	8.54E-95					
Pantr1	0.617	5.80E-53					
Tcf4	0.610	1.62E-113					
Npy2r	0.592	8.86E-184					
Stim2	0.591	1.22E-109					
Nptx1	0.587	4.55E-40					
Cck	0.586	1.30E-88					
Gm34466	0.585	2.89E-264					
Cnr1	0.583	2.05E-25					
Fkbp1a	0.577	6.12E-92					
Dock9	0.574	1.17E-92					
Shisa6	0.571	7.67E-174					
Actr3b	0.569	1.76E-79					
Klhl2	0.566	6.17E-77					
Zbtb20	0.564	1.11E-139					
Epha5	0.563	6.58E-73					
Gm2115	0.562	5.33E-167					
Ak5	0.561	1.44E-48					
Lmo1	0.558	1.05E-121					
Psd	0.558	1.91E-105					
Zeb2	0.557	3.93E-96					
Foxg1	0.554	1.07E-88					
Prdm8	0.545	3.02E-73					
Camk2a	0.544	1.49E-107					
Selenow	0.542	7.33E-146					
Camkv	0.533	1.50E-75					
Kctd12	0.533	2.28E-57					
Camta2	0.532	1.42E-92					
Sema3e	0.530	5.31E-132					
Sipa1l3	0.528	1.44E-130					
Slc17a7	0.527	1.20E-70					
Pcdh20	0.527	2.40E-128					
Bcl11a	0.524	1.13E-76					

Ncdn	0.524	2.59E-84					
Tspan13	0.521	1.27E-62					
Cpne4	0.516	2.35E-21					
Dynll1	0.516	4.34E-95					
Mical2	0.515	2.03E-72					
Nrgn	0.514	1.08E-67					
Nckap1	0.506	2.92E-88					
Nr3c2	0.505	5.19E-90					
Hs3st4	0.503	2.71E-67					
Trim2	0.502	6.87E-80					
Tafa1	0.502	9.36E-102					
Prkcb	0.497	6.90E-64					
Gria2	0.497	2.77E-71					
Fkbp1b	0.495	5.86E-67					
Calm2	0.489	1.89E-75					
Arf1	0.488	7.08E-72					
Syn2	0.487	1.84E-31					
Kcnab2	0.485	8.08E-88					
Khdrbs3	0.482	1.71E-72					
Pfkl	0.479	1.56E-63					
Brinp1	0.478	1.09E-57					
Icam5	0.471	1.72E-57					
Lmo4	0.471	2.16E-33					
Prkce	0.470	6.04E-71					
Clstn2	0.469	1.05E-54					
Ppp1r1a	0.468	4.45E-58					
Sdcbp	0.466	1.42E-55					
Matn2	0.463	1.27E-117					
Adgrb2	0.463	6.21E-79					
Frrs1l	0.462	1.86E-59					
Sez6	0.460	5.00E-34					
Ppm1e	0.455	7.50E-47					
Cplx2	0.454	3.69E-59					
Faah	0.454	3.13E-74					
Camk2b	0.448	1.65E-64					
Rasgef1a	0.448	8.18E-70					
Arpc2	0.445	5.17E-34					
Man1a	0.445	3.52E-79					
Chrm1	0.444	7.84E-60					
Lrrn2	0.443	2.86E-54					
Bcl11b	0.443	1.05E-70					
Arl15	0.441	5.90E-86					
Arf3	0.440	2.16E-97					
Iqgap2	0.439	5.63E-126					
Kcnab1	0.439	7.31E-29					
Slc24a2	0.438	9.29E-60					
Olfm1	0.437	4.11E-49					

Fgf13	0.433	1.60E-48					
Mast3	0.431	4.55E-57					
Limd2	0.428	5.10E-67					
Gabrb3	0.425	2.91E-56					
Cadm2	0.421	2.99E-64					
Actr3	0.420	2.10E-56					
Shisa7	0.415	1.51E-62					
Npdc1	0.413	4.75E-62					
Syne1	0.412	1.40E-54					
Actr2	0.411	2.05E-56					
Crmp1	0.407	6.70E-46					
Plppr2	0.406	2.20E-50					
Baiap2	0.403	7.61E-43					
Rnf112	0.402	7.31E-34					
Grin2b	0.399	1.56E-44					
Calm3	0.399	6.51E-99					
Prrt1	0.396	1.84E-42					
Abracl	0.394	3.53E-65					
Camkk1	0.394	1.69E-39					
Ppp3cb	0.393	1.55E-44					
Rasgrf1	0.392	1.39E-39					
Slc25a22	0.390	4.79E-25					
Galnt17	0.388	4.99E-57					
Nbl1	0.387	1.17E-77					
Ociad2	0.386	3.77E-41					
Thra	0.385	1.03E-58					
Tomm34	0.384	1.01E-41					
Orai2	0.383	2.33E-65					
Bdnf	0.382	5.42E-73					
Itm2b	0.382	6.34E-72					
Amph	0.381	2.69E-33					
Rasd1	0.381	3.37E-63					
Med10	0.380	2.23E-56					
Pfn2	0.379	5.12E-60					
Lefty1	0.377	1.85E-254					
Dlgap1	0.377	7.81E-52					
Fhl2	0.375	7.27E-60					
Pde2a	0.374	8.12E-43					
Crls1	0.373	2.86E-44					
Prickle2	0.373	3.64E-45					
Pcdh1	0.370	6.33E-52					
Rapgefl1	0.369	1.06E-49					
Abi1	0.369	5.45E-35					
Camk1d	0.369	5.83E-29					
Kcnq2	0.369	4.61E-47					
Bok	0.368	0.000192782					
Cops9	0.368	6.31E-66					

Pfn1	0.368	1.25E-23					
Inka2	0.367	2.38E-60					
Cyfip1	0.366	2.63E-49					
Dnm1	0.365	2.96E-35					
Ccnd2	0.365	2.23E-31					
Slc8a1	0.365	3.23E-31					
Rapgef5	0.363	3.78E-31					
Smpd3	0.363	1.92E-59					
Sowaha	0.361	1.18E-51					
Serpina3n	0.359	1.61E-101					
Scn1b	0.357	4.35E-53					
Sh3bgrl3	0.356	1.08E-40					
Zdhhc23	0.356	3.31E-76					
Gmpr	0.355	6.04E-44					
Dagla	0.355	3.95E-42					
Arhgap12	0.352	2.28E-29					
Dusp6	0.352	2.69E-37					
Sh3bp5	0.352	6.34E-61					
Nsf	0.350	1.69E-35					
Tnfrsf21	0.349	4.85E-41					
Ryr2	0.349	6.52E-52					
Lsm8	0.349	1.29E-53					
Schip1	0.347	6.07E-36					
Pls3	0.347	5.55E-36					
Lrrc10b	0.346	1.21E-135					
Ctxn1	0.346	9.02E-29					
Git1	0.345	3.11E-39					
Gnaq	0.344	9.41E-43					
Gpc1	0.344	3.83E-29					
Nptxr	0.343	3.55E-16					
Msra	0.343	1.24E-56					
Gpm6a	0.342	5.07E-59					
Sh3bgrl	0.338	5.91E-40					
Tspan5	0.336	2.10E-40					
Nt5dc3	0.336	9.68E-32					
Gsta4	0.335	5.04E-31					
Tenm2	0.335	2.39E-49					
Mpped1	0.335	1.39E-13					
Numbl	0.334	2.70E-43					
Prnp	0.334	5.48E-38					
Arpc4	0.333	8.87E-41					
Slc8a2	0.332	1.55E-38					
Stum	0.331	9.13E-41					
Arhgap39	0.327	1.06E-49					
Cort	0.327	9.77E-13					
Sphkap	0.327	2.63E-23					
Ywhah	0.326	5.17E-13					

Cpe	0.325	3.04E-75					
Sec14l1	0.325	1.13E-24					
Snn	0.324	5.91E-27					
Plcb1	0.322	2.74E-31					
Cpt1c	0.322	2.23E-36					
Crhbp	0.320	1.47E-14					
Grm5	0.319	1.83E-28					
Cttn	0.317	2.30E-28					
Dbn1	0.317	5.78E-35					
Aak1	0.317	4.01E-33					
Kcnd2	0.316	3.90E-26					
Sh3gl2	0.316	1.10E-15					
Large1	0.316	2.30E-21					
Dtnbp1	0.316	2.47E-32					
Nefl	0.314	1.34E-23					
B3gat1	0.314	1.65E-33					
Kcnip2	0.313	5.74E-32					
Hivep2	0.312	3.52E-27					
Sprn	0.312	3.18E-28					
Homer3	0.312	1.36E-90					
Hapln4	0.311	4.41E-19					
Sort1	0.310	5.79E-32					
Plxna4	0.310	2.42E-43					
Lmo7	0.310	2.81E-54					
Kalrn	0.308	4.30E-28					
Abr	0.308	2.02E-32					
Slit1	0.308	9.81E-59					
Chrm3	0.306	3.42E-60					
Gpd2	0.306	4.87E-29					
Tubb2a	0.306	4.62E-33					
Synj1	0.306	3.42E-27					
Ntrk3	0.305	3.84E-35					
Stxbp5	0.302	3.11E-35					
Lats2	0.302	1.25E-48					
Cyfip2	0.301	9.67E-42					
Grin1	0.301	1.01E-34					
St8sia5	0.301	9.05E-40					
Myo5b	0.301	2.20E-51					
Nbea	0.300	6.87E-30					
Atp6v1g2	0.300	3.55E-31					
Dscaml1	0.298	8.71E-66					
B4galnt1	0.297	4.28E-30					
Map2k1	0.297	2.65E-20					
Hsp90ab1	0.296	2.04E-65					
Ptms	0.296	2.08E-38					
Plekhg5	0.296	6.56E-45					
Fam49a	0.295	2.19E-24					

Matk	0.293	4.82E-26					
Hunk	0.293	1.66E-67					
Fbxl16	0.292	1.87E-45					
Itm2c	0.291	1.48E-41					
Nmt1	0.291	3.25E-28					
Cachd1	0.291	1.20E-60					
Dgkb	0.291	3.52E-28					
Ap2a2	0.291	2.67E-22					
Gprin1	0.290	3.52E-34					
Pip4k2c	0.290	1.34E-32					
Gpr22	0.288	2.35E-39					
Sept9	0.288	2.89E-42					
Lrrtm1	0.286	8.92E-33					
Sertm1	0.286	7.61E-29					
Capza2	0.285	3.50E-26					
Trhde	0.284	1.15E-29					
Plppr5	0.284	2.48E-43					
Lct	0.284	4.28E-153					
Cdc40	0.283	6.14E-46					
Wscd2	0.282	2.73E-61					
Chst1	0.281	5.23E-24					
Rasl11b	0.281	2.55E-18					
Skil	0.280	4.09E-33					
Rimbp2	0.280	1.37E-32					
Raver2	0.279	9.61E-62					
Agap3	0.277	2.25E-26					
Cacna1e	0.277	4.95E-38					
Tmem158	0.277	2.28E-18					
Hsd11b1	0.277	6.11E-39					
Lgi1	0.276	2.02E-16					
Pkp2	0.276	3.06E-107					
Stk25	0.276	1.12E-28					
Sst	0.276	1					
Pebp1	0.275	3.56E-32					
Tanc1	0.275	4.52E-39					
Nectin3	0.275	4.28E-39					
Rogdi	0.275	3.56E-26					
Rasl10a	0.274	6.87E-43					
Epdr1	0.274	1.23E-14					
Prkag2	0.274	2.76E-28					
Gng10	0.273	1.24E-23					
Cttnbp2	0.271	2.64E-22					
Arpc3	0.271	1.55E-23					
Slc24a3	0.271	4.79E-26					
Tnfaip8l3	0.270	1.44E-85					
Creg2	0.270	1.56E-33					
Celf5	0.269	5.36E-25					

Spred2	0.269	5.49E-33					
Lsamp	0.269	1.33E-29					
Aldoa	0.268	1.56E-34					
Slit2	0.266	1.02E-13					
2900097C17Rik	0.266	3.79E-48					
Cdh11	0.266	8.42E-35					
Cpne8	0.266	1.16E-41					
Gm26644	0.266	4.67E-234					
Pclo	0.266	1.26E-21					
Lin7b	0.266	6.72E-19					
Actb	0.266	7.46E-50					
Map4k3	0.265	2.90E-26					
Phyhip	0.264	2.27E-20					
Fgfr1	0.264	7.73E-36					
Rnf182	0.264	4.02E-67					
Sorl1	0.263	2.33E-19					
Rabgap1l	0.262	1.19E-20					
Hk1	0.262	1.02E-24					
Npy	0.262	1					
Kcnn2	0.262	6.84E-35					
Zer1	0.261	2.48E-26					
Ube2ql1	0.260	9.86E-23					
Nrip3	0.259	1.67E-06					
Pwwp2b	0.258	8.59E-60					
Dab1	0.258	3.01E-32					
Tesc	0.257	3.47E-26					
Lurap1l	0.257	8.49E-39					
Dnajb5	0.255	6.93E-19					
Hebp1	0.255	2.11E-35					
Myt1l	0.255	3.58E-23					
Sept3	0.255	1.24E-29					
Yjefn3	0.255	7.16E-10					
Rbfox3	0.254	1.61E-21					
Pja2	0.254	4.26E-24					
Kcnq5	0.254	2.91E-30					
Sv2b	0.254	2.59E-15					
Slc7a14	0.253	8.07E-21					
Tbata	0.253	8.03E-137					
Cacnb1	0.252	4.26E-20					
Kbtbd11	0.252	6.08E-30					
Ksr1	0.252	5.94E-45					
Lrp11	0.252	1.49E-21					
Rapgef2	0.251	1.39E-17					
Hopx	0.251	4.09E-28					
Ywhaz	0.250	4.42E-28					
Elmod1	0.250	3.01E-19					

Supplementary Table 2- WT CA1-CA2-CA3 genes

“CA1 genes” are defined as overexpressed genes between CA1 spots and CA2 & CA3 combined spots using cutoff FC > 1.5 and adjusted p value < 0.05.
“CA2 genes” are defined as overexpressed genes between CA2 spots and CA1 & CA3 combined spots using cutoff FC > 1.5 and adjusted p value < 0.05.
“CA3 genes” are defined as overexpressed genes between CA3 spots and CA1 & CA2 combined spots using cutoff FC > 1.5 and adjusted p value < 0.05.

CA1 genes

Sample	Log2FC(KO/WT)	p_val_adj
Wfs1	2.935	0.000
Fibcd1	2.933	0.000
Calb1	2.920	0.000
Tmsb10	2.829	0.000
Arhgap12	2.817	0.000
Pou3f1	2.809	0.000
Mpped1	2.690	0.000
Ccn3	2.582	0.000
Lypd1	2.567	0.000
Kcnh7	2.564	0.000
Pantr1	2.505	0.000
Doc2b	2.502	0.000
Pex5l	2.435	0.000
Sertm1	2.433	0.000
Gpr161	2.411	0.000
Penk	2.409	0.000
Cd34	2.385	0.000
Efcab6	2.365	0.000
Ndst4	2.349	0.000
Tenm3	2.337	0.000
BC030500	2.322	0.000
Man1a	2.247	0.000
Spon1	2.238	0.000
Dipk2a	2.222	0.000
Adgrl2	2.209	0.000
6330420H09Rik	2.199	0.000
Dbpht2	2.175	0.000
Htr1a	2.169	0.000
Ndst3	2.168	0.000
Cds1	2.168	0.000
Bhlhe40	2.168	0.000
Sorcs3	2.108	0.000
Pou3f3	2.091	0.000
Wnt2	2.084	0.000
Lefty1	2.073	0.000
Bex1	2.040	0.000
Glt8d2	2.034	0.000
Ramp1	2.005	0.000
Fscn1	2.000	0.000
Htr1b	1.977	0.000
Hs6st2	1.969	0.000
B230110G15Rik	1.942	0.000
Mc4r	1.930	0.000
Igfbp4	1.929	0.000
Tyro3	1.910	0.000
Depdc7	1.900	0.000
Zdhhc2	1.897	0.000
Gm34466	1.887	0.000
Epop	1.884	0.000
Lrrn1	1.873	0.000
Cpne8	1.852	0.000
Ptpru	1.842	0.000
Bcr	1.835	0.000
AI593442	1.827	0.000
Ntm	1.821	0.000
Parm1	1.811	0.000
Tmem158	1.803	0.000
Bcl11b	1.803	0.000
Sall2	1.789	0.000
Ccnd1	1.782	0.000
Stac	1.766	0.000
Kcnj9	1.717	0.000
Cdk17	1.712	0.000
Cadps2	1.688	0.000
Gm2115	1.673	0.000
Nr3c1	1.652	0.000
Pole4	1.651	0.000
Plekhg1	1.634	0.000
Dab2ip	1.634	0.000
Rab8b	1.628	0.000
Mal2	1.628	0.000
Ngef	1.620	0.000

CA2 genes

Sample	g2FC(KO/W	p_val_adj
Amigo2	2.815	0.000
Necab2	2.668	0.000
Prss23	2.426	0.000
Arg2	2.131	0.000
Kcna6	2.030	0.000
Vit	1.997	0.000
Pcp4	1.932	0.000
Sostdc1	1.927	0.000
Syce2	1.891	0.000
Rgs4	1.891	0.000
Ntf3	1.876	0.000
Shisa2	1.843	0.000
Tafa2	1.815	0.000
Rgs14	1.813	0.000
Tmem215	1.736	0.000
Ninj1	1.701	0.000
Ptprd	1.665	0.000
Gsto1	1.636	0.000
S100b	1.630	0.000
Dock11	1.616	0.002
Strip2	1.604	0.000
Igf1r	1.571	0.000
Lhx9	1.570	0.000
Asic2	1.569	0.000
Cotl1	1.566	0.000
Ahcyl2	1.537	0.000
Dlc1	1.523	0.001
Ptpn5	1.500	0.000
Dip2a	1.496	0.001
Gpr12	1.493	0.000
Adcy5	1.485	0.001
Slc7a1	1.462	0.000
Fmnl2	1.434	0.002
Zfp804a	1.434	0.000
Phactr2	1.411	0.004
Hibadh	1.408	0.001
Stard5	1.399	0.001
Fabp7	1.399	0.004
Nptx2	1.393	0.000
Ankrd13a	1.378	0.006
Sorcs2	1.370	0.003
Ccdc3	1.365	0.000
Arhgef26	1.365	0.000
Gng4	1.352	0.004
Mgst3	1.342	0.000
E2f6	1.335	0.005
Mt2	1.319	0.000
Acyp2	1.312	0.001
Pcdh17	1.309	0.008
Aldh1a1	1.309	0.003
Plcl1	1.303	0.011
Ablim1	1.289	0.001
Kcng2	1.287	0.000
Nectin1	1.274	0.004
Zmpste24	1.274	0.004
Etfa	1.271	0.008
Lrrc8d	1.268	0.000
Klhl8	1.267	0.003
Prdm8	1.254	0.000
Asph	1.246	0.000
Sel1l3	1.230	0.015
Ankrd45	1.229	0.004
Nrsn2	1.222	0.000
Cidea	1.218	0.007
Lman2	1.217	0.000
Shisal1	1.217	0.031
Mylk	1.210	0.017
Gm16638	1.196	0.014
Mterf4	1.196	0.014
Ctsc	1.187	0.029
Mknk2	1.187	0.029
Rab3b	1.182	0.037

CA3 genes

Sample	g2FC(KO/W	p_val_adj
Rph3a	2.865	0.000
Synpr	2.666	0.000
Prkcd	2.646	0.000
Slit2	2.528	0.000
Kctd4	2.513	0.000
Crlf1	2.361	0.000
Cldn22	2.320	0.000
Trps1	2.286	0.000
Rasl11a	2.232	0.000
Rerg	2.208	0.000
Cpne9	2.130	0.000
Cdh9	2.127	0.000
Cyp26b1	2.035	0.000
Ephb1	2.015	0.000
Ccnd2	1.966	0.000
Rspo2	1.965	0.000
Otos	1.959	0.000
Cacng2	1.935	0.000
Bok	1.856	0.000
Trp53i11	1.842	0.000
Edil3	1.808	0.000
Parva	1.807	0.000
Cd109	1.781	0.000
Galnt14	1.764	0.000
Sulf2	1.744	0.000
Smoc2	1.726	0.000
Neto1	1.704	0.000
Mapk11	1.703	0.000
Mapk3	1.703	0.000
Iyd	1.699	0.000
Tagln3	1.693	0.000
Rgs12	1.677	0.000
Kcnf1	1.640	0.001
Nnat	1.634	0.000
Rcn3	1.632	0.000
Kcnk4	1.632	0.000
Islr2	1.621	0.000
Fxyd1	1.593	0.000
Enox2	1.545	0.000
Ncald	1.524	0.000
Gm26644	1.508	0.000
Camk1	1.497	0.001
Pfkfb3	1.483	0.000
Camk2n2	1.481	0.000
Fbxo2	1.471	0.000
Zfpm2	1.464	0.001
Plcxd2	1.463	0.001
Pcdh8	1.460	0.000
Gpc1	1.439	0.000
Trhde	1.422	0.000
Dbndd1	1.420	0.001
Necab3	1.391	0.003
Aifm3	1.384	0.000
D430041D	1.381	0.000
Unc119	1.366	0.003
Nectin3	1.356	0.000
Ulk1	1.352	0.000
Sh3gl3	1.350	0.002
Wscd1	1.335	0.000
Car10	1.328	0.001
Qpct	1.328	0.001
Grik4	1.324	0.000
Fstl5	1.324	0.001
Tspan17	1.323	0.000
Retreg1	1.318	0.002
Rab15	1.315	0.000
Myh14	1.309	0.000
Ptgs2	1.308	0.000
Fxyd7	1.306	0.000
Homer3	1.305	0.000
Shisa4	1.290	0.000
Tmem114	1.288	0.002

Iqgap2	1.617	0.000
Kcnh3	1.596	0.000
Nr4a1	1.583	0.000
Klhl13	1.555	0.000
Kcnab1	1.555	0.000
Fos	1.554	0.000
Asap2	1.537	0.000
Mylip	1.528	0.000
Kdm5d	1.522	0.000
Ccdc88c	1.517	0.000
Satb2	1.515	0.002
Miat	1.507	0.000
Lix1l	1.501	0.000
Tbc1d1	1.499	0.000
Far2	1.495	0.000
Brdt	1.492	0.000
Vstm2l	1.481	0.000
Lct	1.462	0.000
Syt16	1.458	0.000
Ppp4r4	1.457	0.000
Syt17	1.442	0.001
Igsf9b	1.430	0.000
Efnb3	1.428	0.000
Tacc1	1.423	0.000
Trerf1	1.413	0.000
4921539H07Rik	1.413	0.000
Itpr1	1.408	0.000
Adra2c	1.402	0.001
Cacna1i	1.387	0.001
Pcdhb17	1.375	0.002
Sez6	1.373	0.000
Ypel1	1.373	0.001
Egr1	1.373	0.000
Foxp1	1.364	0.000
Slc35g2	1.364	0.002
Usp1	1.350	0.002
Galntl6	1.350	0.001
Rmi1	1.339	0.001
Ahi1	1.337	0.000
Gm9885	1.335	0.002
Ankrd63	1.333	0.004
Nyap2	1.328	0.001
Adgrl3	1.328	0.001
Kcnb1	1.324	0.000
Tle1	1.324	0.000
Zfpm1	1.323	0.002
Ephb3	1.304	0.001
Egr4	1.294	0.000
Bcl6	1.286	0.000
Itgb1bp1	1.285	0.000
Zfp462	1.279	0.000
Gabra1	1.274	0.000
Ctsz	1.273	0.001
Hunk	1.268	0.000
Sbk1	1.256	0.000
Jakmip1	1.255	0.000
Lmo1	1.255	0.000
Rpgr	1.250	0.003
Dgkh	1.250	0.000
Grhl1	1.247	0.001
Cdc25b	1.245	0.003
Bcl9	1.240	0.002
Crip2	1.239	0.000
9330159F19Rik	1.235	0.000
Kcnq3	1.232	0.000
Abracl	1.220	0.000
Gm19410	1.219	0.005
Sap25	1.219	0.005
Inka1	1.216	0.005
Ptbp3	1.216	0.009
Mb21d2	1.216	0.009
Dab1	1.215	0.000
Klhl5	1.211	0.004
Ybx3	1.210	0.000
Rab27b	1.209	0.000
Ano4	1.202	0.003
Rnf114	1.202	0.001
Ar	1.199	0.001
Lratd1	1.194	0.000
Jph1	1.193	0.001
Itpka	1.192	0.000
Dusp6	1.189	0.000
Ier5	1.184	0.000

Sst	1.172	0.020
Spink8	1.170	0.000
Kcnv1	1.170	0.001
Sms	1.167	0.000
Plch2	1.163	0.000
Lix1	1.156	0.006
Saxo2	1.156	0.024
Pdlim5	1.149	0.030
Dusp5	1.149	0.000
Metrn	1.149	0.029
Prdx6	1.145	0.000
Efr3b	1.141	0.000
Srgap2	1.129	0.002
Ptpro	1.117	0.026
Rexo1	1.116	0.004
Clmn	1.110	0.026
Slc9a4	1.106	0.016
Nrip3	1.094	0.000
Bcar1	1.093	0.025
Cpne4	1.086	0.000
Rnf128	1.085	0.007
Egfem1	1.075	0.046
Grid1	1.074	0.043
Adamts1	1.074	0.043
Tada3	1.073	0.031
Disp3	1.072	0.049
Kcnj4	1.069	0.013
Olfml2b	1.066	0.000
Mpp3	1.063	0.005
Slc4a8	1.062	0.005
Pgd	1.049	0.043
Pcdh10	1.037	0.003
Fezf2	1.037	0.031
Mas1	1.034	0.015
Nelfe	1.029	0.037
Arap2	1.028	0.012
Il16	1.020	0.010
Fgfr3	1.020	0.050
Slc4a7	1.018	0.004
Tmem25	1.002	0.006
Adcy1	0.994	0.000
Fam3c	0.991	0.035
Desi1	0.964	0.000
2010204K	0.957	0.023
Stox2	0.955	0.040
Lynx1	0.952	0.000
Irs2	0.949	0.021
Pygo1	0.948	0.009
Brinp2	0.946	0.000
Hacd2	0.944	0.043
Cdkl2	0.941	0.036
Frzb	0.933	0.046
Coro1c	0.925	0.020
Rapgef5	0.917	0.000
Lsm11	0.916	0.033
Abcg4	0.915	0.020
Ntsr2	0.912	0.019
Man1a2	0.909	0.015
Nek6	0.905	0.032
Inpp5j	0.902	0.030
Tmem178	0.901	0.000
Slc7a4	0.900	0.003
Slc6a15	0.885	0.016
Fkbp5	0.879	0.038
Nsa2	0.872	0.010
B4galnt4	0.870	0.009
Selenom	0.859	0.000
Spock1	0.858	0.006
Hspa2	0.858	0.028
Coro7	0.850	0.008
Mapre1	0.849	0.050
Ly6e	0.830	0.000
Tbc1d14	0.821	0.034
Gabrb1	0.809	0.005
Kcnq5	0.805	0.009
Hapln4	0.804	0.000
Ehd3	0.799	0.039
Bhlhe22	0.797	0.003
Inf2	0.774	0.001
Mt1	0.772	0.000
Trnp1	0.771	0.001
Rgl1	0.749	0.010
Mrtfa	0.745	0.015

Cd200	1.277	0.000
Sema5a	1.260	0.004
Golm1	1.255	0.000
Gpr68	1.250	0.001
Robo1	1.236	0.000
Spock1	1.215	0.000
Ak5	1.212	0.000
Lrrc14	1.208	0.004
Elavl2	1.203	0.000
Tgfb2	1.193	0.000
Coprs	1.187	0.002
Kcnmb4	1.184	0.000
Ak4	1.182	0.000
Fhl1	1.182	0.001
Cpne4	1.181	0.000
Sgsm1	1.181	0.000
Cib2	1.175	0.003
Aopep	1.172	0.000
Me1	1.170	0.000
Ccbe1	1.164	0.003
Kcnc1	1.155	0.000
Nptx1	1.155	0.000
Snta1	1.152	0.001
Spock3	1.150	0.000
Sqle	1.143	0.000
Stx1a	1.133	0.000
Fbf1	1.124	0.002
Banp	1.122	0.000
Mctp1	1.113	0.001
Vamp1	1.112	0.000
Sstr3	1.109	0.002
Nrip3	1.104	0.000
Slc26a4	1.099	0.001
Cited2	1.093	0.002
Hs3st4	1.089	0.000
Gyg	1.089	0.001
Ogfrl1	1.086	0.000
Nptxr	1.083	0.000
Nwd2	1.081	0.003
Adarb1	1.072	0.000
Plrg1	1.070	0.001
Tmeff2	1.064	0.000
Ttc39c	1.064	0.003
Kcnc2	1.055	0.000
Kndc1	1.054	0.000
Cdk14	1.053	0.000
Itpk1	1.042	0.002
Myo5b	1.036	0.000
Inpp1	1.021	0.004
Glrx	1.009	0.004
Celf4	1.006	0.000
Tmbim4	1.005	0.004
Slc7a14	1.005	0.000
Pip5k1c	1.002	0.000
Nos1	0.997	0.005
Il34	0.996	0.001
Blmh	0.983	0.000
Tkt	0.979	0.001
Rims1	0.979	0.004
Cisd3	0.974	0.001
Lmo4	0.973	0.000
Bsn	0.971	0.000
Ap3b1	0.968	0.004
Ppp6r1	0.957	0.000
Mtmr12	0.955	0.004
Fam131b	0.947	0.004
Iqsec3	0.947	0.002
Smyd2	0.945	0.001
Slc35f3	0.928	0.003
Cntnap1	0.925	0.000
Cabp1	0.923	0.000
Tbc1d10b	0.896	0.004
Slc39a10	0.876	0.001
Cachd1	0.871	0.000
Neu1	0.864	0.001
Dok6	0.858	0.002
Cxxc5	0.853	0.005
Npy2r	0.849	0.000
Adora1	0.842	0.000
Adar	0.827	0.002
Rab3ip	0.805	0.002
Erc2	0.802	0.000
Hspa1b	0.790	0.004

Sh3bp4	1.180	0.005
Asap1	1.178	0.000
Flna	1.176	0.003
Tmem109	1.176	0.003
Ccdc112	1.176	0.003
Golga1	1.174	0.006
Etv1	1.171	0.000
Zhx1	1.165	0.000
Por	1.160	0.000
Ccng2	1.159	0.000
Raly	1.149	0.000
Scg2	1.148	0.000
Smad3	1.147	0.009
Ybx1	1.145	0.000
Parn	1.144	0.011
Utp11	1.144	0.011
Gm3764	1.142	0.004
Car11	1.139	0.000
Gm1976	1.131	0.007
Hcfc2	1.129	0.001
Stx6	1.126	0.000
Rasl11b	1.115	0.000
Nadk	1.115	0.005
Rasl10a	1.114	0.000
Zfp329	1.113	0.018
Ranbp10	1.112	0.009
Dapk1	1.108	0.000
Fcho2	1.106	0.011
Atp1b2	1.102	0.000
Lactb	1.100	0.006
1110032F04Rik	1.100	0.011
Ier2	1.098	0.014
Adgrb3	1.094	0.000
Arntl	1.086	0.000
Serpini1	1.084	0.000
Nr1d1	1.077	0.018
Grpel2	1.073	0.002
Spred3	1.073	0.011
Plk2	1.072	0.000
Armc10	1.064	0.013
Krit1	1.064	0.013
Ryr3	1.062	0.000
Tmem200a	1.055	0.000
Cdk19	1.050	0.005
B4galt3	1.048	0.001
Runx2	1.046	0.020
Nudcd1	1.046	0.028
Bex4	1.044	0.012
Ahctf1	1.042	0.010
Cldn10	1.039	0.008
C1qtnf4	1.039	0.000
Plekha5	1.037	0.013
Dguok	1.035	0.006
Arl4d	1.033	0.020
Scarb1	1.032	0.008
Thap12	1.032	0.000
Nmt2	1.031	0.000
Kif5b	1.030	0.000
Nrip1	1.030	0.000
Rgs2	1.030	0.002
Jcad	1.028	0.027
Mex3b	1.027	0.013
Ptn	1.026	0.000
Ep400	1.022	0.002
Midn	1.021	0.015
Usp3	1.016	0.010
Fcho1	1.016	0.027
Ajm1	1.016	0.000
Arl15	1.014	0.000
Vxn	1.012	0.000
Kcnd2	1.010	0.000
Cdk5r2	1.005	0.001
Setdb1	1.004	0.021
Pex3	1.004	0.021
Map4	1.003	0.000
Ddit3	1.002	0.016
Rasd1	1.001	0.000
Dock3	1.000	0.007
Casc4	0.998	0.000
Tmem191c	0.998	0.000
Zfp942	0.992	0.015
Vcl	0.992	0.015
Zfp518b	0.990	0.025

Large1	0.742	0.000
Aprt	0.720	0.020
Plpp6	0.704	0.017
Arhgdig	0.700	0.015
2900026A	0.688	0.015
Fabp3	0.686	0.002
Nrxn2	0.680	0.037
Drap1	0.674	0.000
Zdhhc23	0.670	0.014
Phf20	0.668	0.031
Degs1	0.656	0.024
Cacna2d3	0.651	0.008
Hs3st4	0.646	0.038
C1qtnf4	0.645	0.044
Parp1	0.612	0.003
Tafa5	0.608	0.004

Pigq	0.789	0.002
Tmem9	0.789	0.004
Bdnf	0.785	0.000
Tmem178	0.784	0.002
Trnp1	0.776	0.000
Cacnb3	0.769	0.000
Dnajc6	0.767	0.000
Sgk1	0.761	0.004
Ano3	0.745	0.000
Eno1	0.734	0.000
Cnrip1	0.734	0.000
Pygb	0.722	0.002
Ap3m2	0.720	0.000
Crls1	0.715	0.000
Sybu	0.703	0.003
Gmfb	0.700	0.000
Abhd8	0.689	0.003
Armcx2	0.689	0.002
Ppib	0.682	0.000
Syn2	0.675	0.000
Nrn1	0.675	0.000
Adam11	0.672	0.001
Sh3gl2	0.671	0.000
Tef	0.663	0.002
Tanc1	0.662	0.004
Slc25a22	0.662	0.000
Ly6e	0.661	0.000
Vbp1	0.660	0.004
Iscu	0.655	0.004
Efr3a	0.654	0.004
Madd	0.648	0.000
Camk2n1	0.643	0.000
Clstn2	0.642	0.000
Ypel5	0.641	0.001
Pea15a	0.639	0.001
Pcsk2	0.634	0.000
Chgb	0.633	0.000
Camta1	0.630	0.000
Camkk1	0.627	0.000
Lgi1	0.625	0.000
Myo5a	0.623	0.003
Hmgcs1	0.618	0.002
Sv2a	0.616	0.002
Map2k1	0.615	0.000
Ids	0.612	0.000
Tuba1b	0.605	0.000
Slc1a2	0.599	0.000
Klhl2	0.597	0.000
Hpcal4	0.595	0.000
Dmxl2	0.589	0.000

Cfap100	0.990	0.025
Nr4a2	0.990	0.004
Hnrnph3	0.987	0.011
Trank1	0.982	0.009
Gpr137c	0.975	0.032
Matn2	0.974	0.000
Junb	0.972	0.000
Mettl9	0.971	0.011
Nfyb	0.970	0.000
Zcchc14	0.965	0.003
Klf16	0.965	0.024
Adcy2	0.964	0.000
Rasal1	0.964	0.000
Btg1	0.962	0.025
Dancr	0.962	0.025
Ap1s2	0.962	0.025
Fxyd2	0.962	0.014
Ccng1	0.961	0.003
Prps2	0.961	0.028
Tada1	0.961	0.009
Marcks	0.960	0.001
Ppp1r9a	0.959	0.000
Anapc1	0.958	0.002
Ipmk	0.956	0.015
Lamp5	0.954	0.010
Fam189a1	0.953	0.000
Lmo3	0.953	0.000
Wdr33	0.951	0.012
Usp25	0.951	0.008
Cttnbp2	0.946	0.000
Tmeff1	0.944	0.000
Wsb1	0.941	0.026
Ikzf5	0.941	0.026
Inpp5f	0.939	0.000
Gtf2b	0.939	0.005
Zfp263	0.938	0.025
Vopp1	0.932	0.000
Rbmx	0.931	0.003
Nell1	0.930	0.000
Rnf185	0.930	0.029
Setbp1	0.928	0.039
Rbm7	0.927	0.025
Aldh1b1	0.926	0.014
AC149090.1	0.925	0.000
Celf1	0.922	0.000
Ppm1g	0.921	0.010
Arc	0.921	0.000
Mfsd8	0.919	0.030
Map4k5	0.919	0.008
Tnfrsf25	0.916	0.024
Ccdc88a	0.916	0.024
Ifitm10	0.913	0.002
U2surp	0.912	0.019
Scn3b	0.911	0.000
Arhgap33	0.910	0.015
Hmgcl	0.907	0.028
Gtf3c2	0.905	0.025
Stt3b	0.902	0.004
Pip5k1a	0.902	0.007
Dclk1	0.901	0.000
Mtf2	0.896	0.023
Snx11	0.896	0.044
Acot13	0.895	0.000
Galnt9	0.892	0.000
Arnt2	0.888	0.013
Prune1	0.888	0.046
Sertad2	0.888	0.046
Shfl	0.886	0.017
Cacng3	0.885	0.004
Arfrp1	0.883	0.025
Mras	0.882	0.000
Far1	0.881	0.002
Mis18a	0.881	0.010
Spred1	0.880	0.017
Dcun1d4	0.880	0.001
Sowaha	0.880	0.000
Zeb2	0.879	0.000
Ctnnbl1	0.877	0.045
Phc2	0.877	0.005
Secisbp2l	0.875	0.006
Dzip1	0.873	0.020
Hmgn1	0.873	0.002
Lmtk2	0.869	0.006

Mlip	0.868	0.036
Per1	0.865	0.007
Tial1	0.863	0.003
Pid1	0.863	0.008
Jarid2	0.863	0.037
Smarca1	0.862	0.035
Bmp1	0.862	0.019
Lrrtm2	0.859	0.015
Sec14l1	0.858	0.000
Ing1	0.856	0.037
Dyrk3	0.856	0.021
Gprin1	0.854	0.000
B3galt2	0.853	0.032
Kdm2a	0.853	0.042
Clock	0.852	0.019
Ubtf	0.850	0.000
Dicer1	0.849	0.036
Ccdc9	0.849	0.036
Guf1	0.848	0.043
Orai3	0.848	0.007
Rtkn	0.848	0.033
Rabggta	0.844	0.024
Zfp35	0.844	0.049
Tmem38b	0.844	0.049
H2afx	0.844	0.039
Rnf24	0.844	0.039
Trpc4	0.840	0.004
Txndc16	0.840	0.044
Fam193b	0.839	0.007
Minar2	0.839	0.040
Crim1	0.839	0.000
Skil	0.835	0.000
Clip4	0.835	0.003
Inafm2	0.832	0.002
Zfp948	0.832	0.041
Rnf145	0.828	0.004
Hcn1	0.828	0.002
Srpk1	0.827	0.034
B230334C09Rik	0.825	0.001
Srsf6	0.823	0.002
Kdm4b	0.822	0.011
mt-Nd4l	0.821	0.027
6330403K07Rik	0.821	0.000
Senp6	0.819	0.025
Klf10	0.818	0.021
Klf3	0.818	0.014
Pkig	0.817	0.018
Ext1	0.815	0.013
Ctcf	0.809	0.007
Pcdh19	0.808	0.009
Adam22	0.808	0.012
Ppil4	0.808	0.031
Btg3	0.807	0.021
Ncoa2	0.804	0.012
Ep300	0.804	0.036
Irak1bp1	0.803	0.030
Ugcg	0.795	0.000
Hmgn2	0.795	0.022
Shisa7	0.795	0.000
Bag5	0.793	0.003
Sh3glb1	0.793	0.040
Zc3h12b	0.793	0.050
Ten1	0.793	0.003
Zfp180	0.791	0.020
Sash1	0.791	0.044
Pcif1	0.789	0.024
Slain1	0.789	0.019
Kmt2e	0.789	0.011
Ube2i	0.788	0.024
Acap2	0.788	0.002
Nap1l5	0.788	0.002
Grasp	0.783	0.003
Sccpdh	0.780	0.018
Med10	0.779	0.000
Odc1	0.778	0.013
Car7	0.777	0.010
Ncoa1	0.774	0.004
Pcna	0.773	0.024
Rcc2	0.772	0.021
Dcaf6	0.770	0.033
Sept6	0.770	0.000
Camk2g	0.768	0.042
Fam43b	0.768	0.012

Snd1	0.767	0.020
Kalrn	0.766	0.000
Mfsd4a	0.760	0.034
Cyp46a1	0.759	0.007
Pip5k1b	0.759	0.026
Ralbp1	0.757	0.044
B3galt1	0.757	0.044
Homer2	0.756	0.035
Nudt9	0.756	0.001
Rab40b	0.753	0.003
Kras	0.752	0.011
Sptb	0.751	0.000
Sept9	0.749	0.001
Rbm22	0.747	0.027
Gm42517	0.743	0.033
Gtf3c1	0.742	0.017
Gatad1	0.740	0.023
Zfp106	0.739	0.012
Kctd1	0.737	0.003
B3gat1	0.736	0.000
Extl1	0.733	0.037
Aldh2	0.731	0.028
Wwc1	0.724	0.041
Mfsd14b	0.722	0.049
Naxe	0.722	0.014
Cbx4	0.722	0.030
Slc24a3	0.722	0.000
Ephx4	0.721	0.000
2010300C02Rik	0.720	0.000
Gmpr	0.719	0.000
Csrnp3	0.718	0.043
Egr3	0.713	0.000
Ppp1r12a	0.711	0.002
Ttc14	0.710	0.032
Pnisr	0.710	0.006
Kcnn2	0.707	0.010
Ivns1abp	0.707	0.023
Celf3	0.705	0.046
Plcl2	0.705	0.043
Lin7c	0.705	0.008
Git1	0.703	0.000
Metap2	0.701	0.018
Ndufaf3	0.699	0.017
Kcnip4	0.697	0.011
Ash1l	0.696	0.031
Churc1	0.696	0.011
Rprml	0.692	0.000
Clk1	0.689	0.016
Ppp1r21	0.687	0.011
Dnajb1	0.686	0.010
Slitrk3	0.682	0.011
Tnik	0.682	0.027
Syt5	0.682	0.005
Mib2	0.680	0.048
Lsm8	0.678	0.009
Ankrd10	0.677	0.049
Kcnj3	0.676	0.000
Sap30bp	0.676	0.037
Jph4	0.675	0.000
Ccl27a	0.675	0.035
Ifi27	0.674	0.008
Fam49b	0.671	0.000
Fdx2	0.671	0.015
Endod1	0.668	0.036
Tceal9	0.668	0.046
Abhd17c	0.668	0.013
Celf5	0.667	0.000
Shank1	0.665	0.015
Gpr27	0.663	0.043
Spink8	0.659	0.011
Fam234b	0.658	0.000
Map1s	0.658	0.020
Hp1bp3	0.655	0.000
Casd1	0.655	0.013
Arhgef3	0.655	0.038
Hnrnpdl	0.655	0.000
Ptma	0.652	0.000
Lrrfip1	0.651	0.041
Fam49a	0.650	0.000
Pde4a	0.649	0.025
Grin2a	0.642	0.000
Ergic1	0.641	0.011
Stmn4	0.641	0.000

Aebp2	0.639	0.045
Ppm1k	0.638	0.023
Gda	0.638	0.017
Ppp1r1a	0.637	0.000
Fbxl5	0.637	0.019
Adcy1	0.632	0.010
Tspan13	0.631	0.000
Brd9	0.627	0.001
Rbbp7	0.627	0.000
Cyld	0.624	0.003
Med9	0.623	0.025
Bub3	0.621	0.024
Brms1l	0.620	0.046
Agrn	0.618	0.029
L1cam	0.616	0.040
Lrp1	0.615	0.003
Egln2	0.615	0.033
Zbtb4	0.614	0.024
Tmed7	0.610	0.011
Ivd	0.609	0.039
Gpd2	0.609	0.020
Iqsec2	0.609	0.003
Lmo7	0.609	0.005
Prkcb	0.608	0.000
Mrpl58	0.605	0.044
R3hdm1	0.603	0.006
Exd2	0.602	0.011
Srrm2	0.601	0.021
Fam171b	0.599	0.000
Ppp4r2	0.596	0.009
Slc8a1	0.596	0.000
Grin2b	0.593	0.000
Rps27l	0.591	0.036
R3hdm2	0.590	0.048
Cpeb4	0.588	0.025
Washc2	0.588	0.030
Numbl	0.586	0.000
St6galnac5	0.585	0.000

Supplementary Table 3- MRKO-WT DEGs CA1

“CA1 DEGs” are defined as differentially expressed genes (DEGs) between CA1 spots in WT and CA1 spots in MR KO using cutoff FC > 1.5 and adjusted p value < 0.05.

Sample	Log2FC(KO/WT)	p_val_adj
Kcnf1	2.415	0.000
Grin3a	2.200	0.000
Fxyd6	2.100	0.002
Rgma	1.905	0.000
Rspo2	1.866	0.002
Rgcc	1.789	0.000
D430041D05Rik	1.781	0.000
Cpne5	1.761	0.001
St6gal2	1.681	0.000
Npbwr1	1.624	0.002
Stk32c	1.579	0.004
Marcksl1	1.539	0.000
Kcnt1	1.512	0.003
Ak5	1.487	0.000
Plxnd1	1.466	0.000
Dach1	1.465	0.000
Cacng2	1.432	0.013
Adgra1	1.432	0.024
Vstm2a	1.391	0.020
Rgs4	1.383	0.000
Neo1	1.373	0.014
Wls	1.371	0.022
Cntnap5a	1.334	0.016
Nwd2	1.316	0.031
Sulf2	1.315	0.016
Osbpl1a	1.311	0.002
Kcnmb4	1.298	0.005
Car12	1.296	0.001
Ighm	1.270	0.005
Gm34466	1.253	0.000
Wdr95	1.247	0.034
Slc6a7	1.247	0.034
Serf1	1.247	0.030
Gm26644	1.235	0.005
Fez2	1.234	0.015
Fhod3	1.232	0.029
Tspyl3	1.222	0.019
Prdm8	1.222	0.002
Xylt1	1.190	0.011
Selenoh	1.189	0.035
Ldb2	1.183	0.001
Dusp14	1.174	0.002
Gfra2	1.151	0.001
Pcdh17	1.127	0.018
2310011J03Rik	1.122	0.002
Gap43	1.089	0.000
Fezf2	1.088	0.044
Gm19410	1.078	0.002
Mfhas1	1.076	0.033
Hmgxb3	1.063	0.033
Pop7	1.053	0.016
Celf4	1.035	0.000
Pmepa1	1.028	0.026

Cds1	1.023	0.000								
Stim1	1.005	0.034								
Crtac1	0.991	0.008								
Satb1	0.989	0.005								
Dbp	0.958	0.043								
Fkbp1b	0.934	0.000								
Gsta4	0.931	0.000								
Lmo4	0.925	0.000								
Aph1a	0.925	0.043								
Nr3c1	0.893	0.000								
Sft2d1	0.874	0.031								
Tmem115	0.874	0.049								
Pcnx2	0.837	0.009								
Dst	0.834	0.030								
Fxyd2	0.830	0.002								
Zc2hc1a	0.829	0.045								
1110032F04Rik	0.825	0.042								
Nefm	0.823	0.000								
Ier5	0.796	0.000								
Ripor2	0.791	0.005								
Alcam	0.772	0.019								
Npy2r	0.763	0.042								
Crip2	0.724	0.003								
Dda1	0.719	0.022								
Stmn1	0.712	0.000								
Fxyd7	0.707	0.000								
Pcsk2	0.697	0.000								
Sparcl1	0.686	0.000								
Higd1a	0.679	0.003								
Hs6st2	0.659	0.040								
Dtnbp1	0.636	0.000								
Ccl27a	0.627	0.015								
Tubb3	0.625	0.000								
St8sia5	0.611	0.027								
Pkp4	-0.593	0.001								
9330159F19Rik	-0.597	0.017								
Crim1	-0.599	0.017								
Ube2e2	-0.603	0.001								
Csnk1a1	-0.604	0.000								
Add2	-0.615	0.000								
Cyld	-0.616	0.046								
Med14	-0.618	0.044								
Cabp7	-0.626	0.023								
Parp1	-0.626	0.025								
Gm2115	-0.628	0.000								
Sowaha	-0.629	0.012								
Pfn1	-0.630	0.000								
Jakmip1	-0.640	0.034								
Arel1	-0.663	0.004								
Arpc2	-0.682	0.000								
Sphkap	-0.703	0.000								
Doc2b	-0.705	0.020								
Cacnb2	-0.720	0.039								
Prkca	-0.734	0.000								
Map4	-0.736	0.000								
Susd6	-0.809	0.013								
Dhx33	-0.815	0.048								
Wnt2	-0.823	0.039								

Clk1	-0.824	0.037								
Lurap1l	-0.830	0.015								
Arhgap39	-0.833	0.001								
Sh3bp5	-0.834	0.000								
Ptpru	-0.836	0.006								
Lrrn1	-0.883	0.002								
Ptn	-0.908	0.002								
Uap1	-0.921	0.029								
Klf3	-0.936	0.028								
Dr1	-0.944	0.031								
Plpp6	-0.951	0.001								
Pdxk	-0.959	0.033								
Gpt2	-0.974	0.014								
Pcdh20	-0.974	0.001								
Adcy1	-0.984	0.000								
Scn3b	-0.988	0.000								
Pkp2	-0.999	0.000								
Shisa6	-1.006	0.000								
Srrt	-1.018	0.003								
Agpat5	-1.025	0.023								
Galnt16	-1.028	0.000								
Washc2	-1.031	0.002								
Abracl	-1.033	0.000								
Ppp1r13b	-1.038	0.012								
Dipk2a	-1.041	0.008								
Ust	-1.044	0.005								
Slit1	-1.064	0.001								
Rnf114	-1.066	0.024								
Ep300	-1.089	0.025								
Dhcr24	-1.094	0.037								
Brf2	-1.119	0.030								
Dapk1	-1.121	0.001								
Rasd1	-1.143	0.000								
Cbll1	-1.165	0.032								
Htr1a	-1.169	0.004								
Galnt17	-1.213	0.000								
Nbl1	-1.222	0.000								
Anxa11	-1.231	0.003								
Lamp5	-1.240	0.008								
Wipf3	-1.281	0.000								
Pip5k1b	-1.295	0.001								
Ksr1	-1.295	0.000								
Mdga1	-1.296	0.004								
Foxk1	-1.308	0.024								
Calb1	-1.382	0.000								
Lefty1	-1.404	0.000								
Serpina3n	-1.429	0.000								
4921539H07Rik	-1.446	0.003								
Ttr	-1.487	0.016								
Hspa1b	-1.490	0.002								
Stac	-1.490	0.002								
Dyrk3	-1.597	0.000								
Nr3c2	-1.919	0.000								
Mc4r	-1.930	0.000								
Olfml2b	-1.956	0.000								
Spink8	-2.043	0.000								
Penk	-2.276	0.000								
Dusp5	-2.449	0.000								

Cpne7	-2.524	0.000								

Supplementary Table 3- MRKO-WT DEGs CA2

“CA2 DEGs” are defined as differentially expressed genes (DEGs) between CA2 spots in WT and CA2 spots in MR KO using cutoff FC > 1.5 and adjusted p value < 0.05.

**Sample**	**Log2FC(KO/WT)**	**p_val_adj**
Npbwr1	2.969	0.000
Hs6st2	2.847	0.000
Kcnf1	2.700	0.001
Wfs1	2.502	0.000
Gm34466	2.457	0.000
Gap43	2.425	0.000
Cdk5rap3	2.313	0.003
Asap1	2.262	0.000
Nr3c1	2.240	0.000
Ldb2	2.238	0.014
Glt8d2	2.221	0.004
Efnb3	2.191	0.000
AI593442	2.173	0.000
Tmem158	2.164	0.000
Tmeff1	2.122	0.000
Tmsb10	2.122	0.000
BC030500	2.109	0.004
Bmp3	2.084	0.028
Rph3a	2.040	0.005
Cds1	2.038	0.000
Fras1	2.023	0.027
Tyro3	2.019	0.000
Ier5	1.937	0.000
Cyp26b1	1.912	0.045
B230110G15Rik	1.912	0.045
Ablim2	1.907	0.001
Slit2	1.901	0.005
Spon1	1.891	0.000
Fxyd7	1.863	0.000
Pde7a	1.843	0.043
1110032F04Rik	1.837	0.007
Kcnh3	1.832	0.011
Dach1	1.832	0.011
Dbp	1.793	0.044
Parm1	1.788	0.000
Bhlhe40	1.768	0.000
Cbfa2t3	1.756	0.031
Ccn3	1.754	0.003
Pou3f1	1.749	0.000
Syt17	1.738	0.046
Kcnq3	1.735	0.007
Ephb3	1.697	0.042
Ncam1	1.685	0.001
Ptar1	1.680	0.022
Upf2	1.672	0.007
Cadps2	1.656	0.000
Wdr44	1.638	0.042
Sult2b1	1.638	0.042
Ccdc126	1.638	0.042
Man1a	1.630	0.001
Vstm2l	1.627	0.000
Fscn1	1.624	0.005
Kcnh7	1.624	0.021

Foxp1	1.614	0.011								
Pcdh7	1.610	0.018								
Camk1	1.589	0.018								
Fgf11	1.581	0.030								
Lmo4	1.576	0.000								
Tmcc3	1.576	0.041								
Golga7	1.568	0.020								
Rasd2	1.565	0.000								
Trmt1	1.553	0.034								
Cdk18	1.553	0.034								
Tmem132a	1.552	0.001								
Fam171a2	1.538	0.018								
Pgam2	1.482	0.011								
Pantr1	1.467	0.000								
Mtcl1	1.463	0.036								
Eif2s2	1.462	0.001								
Kcnab1	1.455	0.000								
Inpp5a	1.452	0.010								
Sept6	1.450	0.002								
Gm27032	1.447	0.036								
Rspo2	1.447	0.036								
Arl15	1.446	0.000								
Pik3r4	1.426	0.043								
Dock3	1.425	0.010								
Cacng3	1.425	0.020								
Rasal1	1.425	0.006								
Tmem109	1.417	0.040								
Nmt2	1.412	0.011								
Mpped1	1.406	0.002								
Cdh9	1.395	0.048								
Gsta4	1.390	0.000								
Ttll1	1.370	0.042								
Scg2	1.366	0.000								
Cspg5	1.365	0.036								
Ttyh1	1.365	0.003								
Mylip	1.358	0.018								
Far1	1.340	0.004								
Marcks	1.333	0.004								
Gga3	1.332	0.004								
6330403K07Rik	1.329	0.000								
Fbxl5	1.327	0.003								
Lmo3	1.326	0.000								
Etv1	1.325	0.000								
Snx11	1.320	0.046								
Gpr137c	1.320	0.046								
Ttc39b	1.299	0.028								
Osbpl6	1.286	0.030								
Prkcb	1.280	0.000								
Zdhhc2	1.271	0.000								
Smad3	1.270	0.049								
Fibcd1	1.266	0.030								
Alcam	1.262	0.031								
Bysl	1.255	0.031								
Arnt2	1.248	0.004								
Itpr1	1.232	0.002								
Zc2hc1a	1.231	0.006								
Pkia	1.225	0.011								
Pole4	1.211	0.049								

Mal2	1.181	0.000								
Pcdh19	1.181	0.008								
Dennd5a	1.181	0.028								
Creg2	1.179	0.002								
Gpd2	1.175	0.000								
Bex1	1.165	0.041								
Ppdpf	1.161	0.013								
Rab8b	1.154	0.033								
Pex5l	1.152	0.026								
Cpne8	1.140	0.007								
Cadm1	1.138	0.006								
Gpr27	1.122	0.041								
Igfbp4	1.107	0.005								
Nfyb	1.091	0.030								
Lmo7	1.080	0.016								
Hcn1	1.072	0.019								
Sirpa	1.072	0.002								
Inpp5f	1.053	0.038								
Nsd3	1.046	0.032								
St8sia5	1.025	0.021								
Ets2	1.021	0.028								
Zfp365	1.020	0.000								
Pou3f3	1.017	0.046								
Tceal9	1.015	0.041								
Hp1bp3	0.973	0.008								
Tmem191c	0.968	0.039								
Pcdh17	0.968	0.022								
Dusp6	0.951	0.045								
Cdk17	0.926	0.023								
Smap1	0.902	0.004								
Chl1	0.899	0.032								
Nap1l5	0.885	0.008								
Caln1	0.878	0.041								
Inka2	0.873	0.024								
R3hdm1	0.862	0.000								
Cntnap2	0.858	0.029								
Kcnk1	0.836	0.034								
Gnb5	0.831	0.005								
Dctn6	0.830	0.042								
B230219D22Rik	0.830	0.029								
Stx7	0.829	0.017								
Cacna2d1	0.822	0.047								
Papss1	0.814	0.041								
Dtnbp1	0.808	0.010								
Mrtfb	0.799	0.009								
Gabra1	0.797	0.001								
Hnrnpdl	0.793	0.017								
Acot13	0.755	0.027								
Mturn	0.755	0.046								
Aars	0.749	0.046								
Egr1	0.745	0.044								
Ttc9b	0.736	0.037								
Fkbp1b	0.734	0.028								
Fhl2	0.732	0.041								
Egr3	0.729	0.022								
Sez6	0.728	0.002								
Hspa12a	0.712	0.033								
St6galnac5	0.693	0.000								

Fbxw7	0.667	0.041								
Jph4	0.663	0.003								
Itpka	0.659	0.004								
Pcsk2	0.644	0.000								
Plppr4	0.640	0.000								
Ephx4	0.634	0.040								
Epha7	0.629	0.007								
Celf5	0.628	0.022								
Gria3	0.617	0.045								
Vti1b	0.612	0.002								
Serpini1	0.595	0.041								
Stmn1	0.593	0.000								
Mmd	0.588	0.000								
Capzb	-0.590	0.001								
Cadm3	-0.600	0.022								
Limd2	-0.601	0.023								
Rapgef5	-0.617	0.004								
Camta2	-0.617	0.000								
Pfkp	-0.622	0.049								
Nbl1	-0.642	0.038								
Fabp3	-0.671	0.009								
Dlat	-0.676	0.021								
Kctd12	-0.679	0.036								
Plch2	-0.680	0.007								
Syt13	-0.684	0.013								
Nsmf	-0.685	0.000								
Ube2e2	-0.712	0.029								
Galnt17	-0.727	0.040								
Cabp1	-0.732	0.036								
Napg	-0.740	0.016								
Cacna2d3	-0.762	0.017								
Arhgef4	-0.764	0.000								
Ppm1e	-0.771	0.000								
Clip1	-0.774	0.037								
Slc7a4	-0.776	0.019								
Tmed9	-0.786	0.003								
Brinp2	-0.798	0.021								
Ralgapa1	-0.805	0.002								
Galnt16	-0.809	0.047								
Srgap3	-0.809	0.002								
Mgst3	-0.810	0.012								
Lgi1	-0.815	0.003								
Chst2	-0.826	0.004								
Cdipt	-0.828	0.026								
Ppp1r9b	-0.862	0.000								
Lamtor2	-0.869	0.037								
Prkca	-0.871	0.000								
Prkag2	-0.874	0.010								
Snph	-0.900	0.030								
Slc35c2	-0.905	0.018								
Prdx6	-0.906	0.036								
Cops4	-0.912	0.019								
Camta1	-0.920	0.001								
Ly6e	-0.930	0.000								
Cabp7	-0.932	0.000								
Desi1	-0.935	0.000								
Extl2	-0.938	0.030								
Trnp1	-0.939	0.000								

Mmp17	-0.945	0.000								
Tafa2	-0.946	0.029								
Errfi1	-0.946	0.034								
Ncald	-0.953	0.006								
Ppfia2	-0.957	0.002								
Pfn1	-0.967	0.000								
Sphkap	-0.986	0.003								
Arpc2	-0.999	0.000								
2900026A02Rik	-1.021	0.003								
Hapln4	-1.029	0.000								
Gpt2	-1.057	0.017								
Rgs14	-1.072	0.000								
Adora1	-1.074	0.005								
Drap1	-1.075	0.000								
B4galnt4	-1.085	0.011								
Btbd3	-1.092	0.004								
Pcdh1	-1.093	0.001								
Lman2	-1.095	0.001								
Kcnv1	-1.099	0.038								
Plpp6	-1.103	0.000								
Ptpn5	-1.106	0.000								
Slc4a7	-1.109	0.027								
Phf20	-1.117	0.015								
Nsa2	-1.119	0.010								
Selenom	-1.125	0.000								
Aprt	-1.126	0.003								
Bhlhe22	-1.129	0.000								
Prrc2a	-1.130	0.038								
Pycr2	-1.130	0.038								
Parp1	-1.142	0.000								
Inf2	-1.156	0.000								
Rnf182	-1.169	0.043								
Peak1	-1.187	0.038								
Washc2	-1.198	0.002								
Susd6	-1.202	0.033								
Ntsr2	-1.204	0.033								
Spock1	-1.207	0.000								
Lsm11	-1.213	0.038								
Adcy1	-1.231	0.000								
Ksr1	-1.234	0.002								
Tafa5	-1.237	0.000								
Pdk2	-1.249	0.011								
Mtmr9	-1.265	0.012								
Robo1	-1.282	0.037								
Tspan17	-1.283	0.042								
Cachd1	-1.286	0.028								
Anxa11	-1.288	0.026								
Cpne4	-1.309	0.000								
Lhx2	-1.313	0.031								
Wscd1	-1.316	0.027								
Ptprd	-1.333	0.001								
Gpr155	-1.335	0.033								
Zmpste24	-1.338	0.037								
Lynx1	-1.347	0.000								
Ppp1r16a	-1.350	0.047								
Efr3b	-1.350	0.000								
Plxna1	-1.358	0.000								
Tubb2b	-1.400	0.007								

Grik4	-1.401	0.019								
Negr1	-1.401	0.041								
Tmem178	-1.412	0.000								
Gpr12	-1.422	0.024								
Slit1	-1.444	0.001								
Prdm8	-1.446	0.000								
Tbl3	-1.454	0.031								
Stoml1	-1.460	0.012								
Golga7b	-1.471	0.018								
Hspa1b	-1.478	0.030								
Adcy5	-1.485	0.035								
Cables2	-1.494	0.015								
Hspa1a	-1.509	0.028								
E2f6	-1.536	0.042								
Pcyt1b	-1.536	0.043								
Nkapd1	-1.536	0.042								
Asph	-1.536	0.000								
Lurap1l	-1.539	0.048								
Cacna1c	-1.547	0.013								
Slc9a4	-1.547	0.029								
Slc7a1	-1.550	0.004								
Ahcyl2	-1.552	0.033								
Gm49980	-1.576	0.031								
Zfp804a	-1.582	0.001								
Kndc1	-1.584	0.005								
Dapk1	-1.589	0.023								
Disp3	-1.611	0.043								
Phactr2	-1.611	0.043								
Sms	-1.625	0.000								
Ostf1	-1.626	0.004								
Wipf3	-1.653	0.000								
Hs3st4	-1.660	0.000								
Slc39a14	-1.662	0.021								
Mdga1	-1.663	0.038								
Itga7	-1.683	0.042								
Kit	-1.691	0.018								
Nr3c2	-1.709	0.000								
Clmn	-1.719	0.022								
Asic2	-1.719	0.000								
Lrrc8d	-1.729	0.001								
Ablim1	-1.732	0.006								
Hspa2	-1.738	0.001								
Mpp3	-1.744	0.000								
G6pc3	-1.751	0.041								
Trhde	-1.761	0.003								
Gsto1	-1.772	0.000								
Stard5	-1.779	0.008								
Mbd2	-1.798	0.021								
Mas1	-1.825	0.002								
Rnf128	-1.850	0.001								
Slc22a23	-1.859	0.029								
Gm48992	-1.859	0.029								
Zfas1	-1.860	0.014								
Frzb	-1.883	0.004								
Nectin1	-1.883	0.004								
Ptpro	-1.904	0.009								
Tgfb2	-1.910	0.001								
Homer3	-1.918	0.011								

Erc2	-1.920	0.000								
Golm1	-1.931	0.001								
Serpina3n	-1.969	0.000								
Fam102a	-1.979	0.003								
Fkbp5	-1.979	0.001								
Flrt3	-1.979	0.001								
Dock11	-1.991	0.036								
Shisa2	-2.003	0.003								
Nebl	-2.011	0.000								
Ntf3	-2.012	0.031								
Ccbe1	-2.020	0.008								
Plcl1	-2.063	0.014								
Ccdc3	-2.119	0.000								
Syce2	-2.183	0.000								
Tmem215	-2.198	0.005								
Vit	-2.198	0.003								
Kcng2	-2.210	0.000								
Arg2	-2.267	0.003								
Dusp5	-2.322	0.000								
Igf1r	-2.355	0.000								
Ptgs2	-2.367	0.004								
Cidea	-2.423	0.000								
Pcp4	-2.469	0.000								
Rem2	-2.528	0.001								
Arhgef26	-2.535	0.000								
Cotl1	-2.704	0.000								
Bok	-2.740	0.000								
Il16	-2.945	0.000								
Spink8	-2.969	0.000								
Amigo2	-3.107	0.000								
Cpne7	-3.127	0.000								
Olfml2b	-3.357	0.000								
Necab2	-3.407	0.000								
Strip2	-3.529	0.000								

Supplementary Table 3- MRKO-WT DEGs CA3

“CA3 DEGs” are defined as differentially expressed genes (DEGs) between CA3 spots in WT and CA3 spots in MR KO using cutoff FC > 1.5 and adjusted p value < 0.05.

Sample	Log2FC(KO/WT)	p_val_adj
Gfra2	2.109	0.000
Hs6st2	1.894	0.000
Bmp3	1.867	0.004
Slc18a2	1.843	0.002
Myo1b	1.737	0.003
Nr3c1	1.688	0.000
Kcna6	1.662	0.001
Nr4a2	1.645	0.000
Fxyd6	1.611	0.007
Nrep	1.598	0.004
Rgs14	1.546	0.000
Aqp4	1.545	0.009
Mb21d2	1.536	0.028
Doc2a	1.521	0.000
Crip2	1.506	0.013
Tyro3	1.500	0.001
Sez6	1.460	0.000
Dnal1	1.437	0.027
Prss23	1.431	0.029
Mxd4	1.425	0.031
3830406C	1.425	0.017
Angptl6	1.399	0.049
Furin	1.386	0.011
Pou3f3	1.361	0.034
Mcub	1.305	0.007
Abcd3	1.292	0.006
Men1	1.285	0.003
Car10	1.268	0.000
Atp11b	1.265	0.040
Wnt7b	1.196	0.044
Fjx1	1.191	0.049
Nudt10	1.177	0.014
Rgs12	1.166	0.006
Vxn	1.160	0.001
Pcp4	1.156	0.026
3110039M	1.155	0.013
Nell1	1.150	0.001
Cyp26b1	1.143	0.003
Bcar1	1.132	0.045
Gap43	1.122	0.000
Inpp5a	1.111	0.032
Tmx1	1.107	0.010
Gm47033	1.096	0.046
Ppp2r5e	1.092	0.026
Samd5	1.088	0.033
Rgs4	1.087	0.027
Zfp365	1.070	0.000
Elavl4	1.070	0.017
Focad	1.067	0.022
Pcdh7	1.051	0.014
Mal2	1.023	0.007

Sept11	1.012	0.008								
Adcy2	1.005	0.001								
Asap1	0.995	0.001								
Rap1gap2	0.991	0.000								
Prmt2	0.975	0.000								
Prkcd	0.974	0.000								
Marcks	0.947	0.045								
Rab3c	0.946	0.003								
Naxe	0.930	0.019								
Rasal1	0.925	0.004								
Alcam	0.892	0.000								
B2m	0.890	0.008								
Cdh9	0.843	0.000								
Rspo2	0.838	0.022								
Arpp21	0.771	0.034								
Macrod2	0.765	0.016								
Gabrb1	0.754	0.017								
Tmem158	0.751	0.045								
Ahi1	0.736	0.038								
Chl1	0.703	0.009								
Spock3	0.701	0.000								
Slit2	0.693	0.001								
Stmn1	0.669	0.000								
Reps2	0.667	0.000								
Osbpl1a	0.657	0.043								
Homer1	0.634	0.049								
Fkbp1b	0.632	0.037								
Hcfc1r1	0.599	0.014								
Hs3st4	-0.606	0.000								
Camta1	-0.631	0.000								
Slit1	-0.634	0.039								
Serpina3n	-0.636	0.048								
Kctd4	-0.639	0.007								
Prickle2	-0.645	0.013								
Ncald	-0.652	0.000								
March5	-0.653	0.046								
Cabp1	-0.653	0.001								
Lynx1	-0.673	0.026								
Acsl4	-0.714	0.016								
Sdhd	-0.722	0.038								
Wscd1	-0.739	0.013								
Pygb	-0.757	0.013								
Elavl2	-0.817	0.002								
Ppfia2	-0.848	0.034								
Neto1	-0.851	0.000								
Anxa11	-0.872	0.008								
Sulf2	-0.878	0.007								
2010300C0	-0.881	0.045								
Lrrc10b	-0.882	0.009								
Wipf3	-0.911	0.000								
Tspan17	-0.916	0.003								
Parp1	-0.919	0.029								
Keap1	-0.919	0.013								
Kbtbd11	-0.924	0.027								
Ppp1r13b	-0.988	0.016								
Galnt17	-0.988	0.000								

Sgsm1	-0.990	0.010								
St3gal2	-1.015	0.017								
Banp	-1.047	0.025								
Rem2	-1.056	0.007								
Nr3c2	-1.085	0.003								
Syt12	-1.091	0.044								
Kcna2	-1.095	0.008								
Scn3b	-1.107	0.000								
Pkp2	-1.125	0.018								
Ephb1	-1.133	0.049								
Tanc1	-1.138	0.001								
Rtn4rl1	-1.168	0.000								
Snta1	-1.173	0.013								
Cebpd	-1.187	0.024								
Bok	-1.197	0.000								
Arrb2	-1.200	0.021								
Hspa1b	-1.209	0.002								
Cyp7b1	-1.244	0.022								
Fcf1	-1.256	0.049								
Selenoi	-1.256	0.049								
Itga7	-1.264	0.034								
Golga7b	-1.319	0.001								
Dhx33	-1.347	0.013								
Homer3	-1.401	0.000								
Dusp5	-1.442	0.027								
Tubb2b	-1.444	0.008								
Ttr	-1.453	0.007								
Camk2n2	-1.470	0.003								
Golm1	-1.518	0.000								
Aifm3	-1.535	0.008								
Kit	-1.577	0.013								
Robo3	-1.630	0.022								
Rcn3	-1.663	0.012								
Pla2g6	-1.696	0.006								
Lurap1l	-1.756	0.000								
Myo5b	-1.798	0.000								
Nnat	-1.900	0.000								
Smoc2	-1.946	0.000								
Necab2	-2.047	0.006								
Strip2	-2.107	0.002								
Arhgef26	-2.116	0.001								
Rasd1	-2.125	0.001								
Cpne7	-2.204	0.000								
Crlf1	-2.481	0.000								

Supplementary Table 3- MRKO-WT DEGs Dentate Gyrus

“DG DEGs” are defined as differentially expressed genes (DEGs) between DG spots in WT and DG spots in MR KO using cutoff FC > 1.5 and adjusted p value < 0.05.

**Sample**	**Log2FC(KO/WT)**	**p_val_adj**
Krt12	2.6913	0.000
Atp2b4	2.1746	0.000
A830036E02Rik	2.1239	0.000
Dpy19l1	2.0630	0.000
Osbpl1a	2.0516	0.000
Unc13c	1.9930	0.000
Epop	1.9273	0.000
Tox3	1.8626	0.000
A2ml1	1.8394	0.000
Grin3a	1.8347	0.000
Sez6	1.8072	0.000
Hpcal1	1.7508	0.000
Slit2	1.7031	0.000
Nrip3	1.7003	0.000
Tmem114	1.6995	0.000
Sema5b	1.6924	0.000
Kcnf1	1.6693	0.000
Wfs1	1.6161	0.000
Filip1	1.5996	0.000
Slc35f1	1.5652	0.000
Nos1	1.4520	0.000
Ak4	1.4325	0.000
Crtac1	1.4321	0.000
Wdr6	1.4028	0.000
Krt2	1.3987	0.000
Id2	1.3759	0.000
Mycn	1.3744	0.000
Plppr3	1.3296	0.000
Alcam	1.3206	0.000
Rasal1	1.3174	0.000
Foxo6	1.2991	0.000
Satb1	1.2914	0.000
Bcor	1.2884	0.000
Col22a1	1.2543	0.000
Cdhr1	1.2275	0.000
Aig1	1.2107	0.000
Sst	1.1905	0.037968854
Tenm2	1.1699	0.000
Tmem179	1.1699	0.000
Cdh13	1.1653	0.001
Fjx1	1.1538	0.000
Arhgap15	1.1467	0.000
H2-DMa	1.1402	0.001
Zfp467	1.1327	0.002
Ajap1	1.1281	0.000
Zfp827	1.1134	0.001
Gsta4	1.1125	0.001
Gdf10	1.1093	0.000
Gfap	1.1093	0.000
Pabpn1	1.1056	0.001
Ptn	1.0807	0.004277288
Nwd2	1.0750	0.002
Grb14	1.0719	0.000
Cd200	1.0709	0.001
Dcc	1.0635	0.002
Cdk18	1.0532	0.004366499

Arl15	1.0517	0.001								
mt-Atp8	1.0500	0.004819869								
Rab4a	1.0450	0.001								
Lasp1	1.0400	0.007077644								
Mpzl1	1.0366	0.002								
Oprl1	1.0357	0.003								
Pdyn	1.0327	0.000								
Cspg5	1.0316	0.000								
Fat3	1.0295	0.001								
St3gal1	1.0290	0.001								
Gpld1	1.0287	0.003321351								
Cacng3	1.0283	0.000								
Dner	1.0251	0.002								
Stpg1	1.0247	0.002								
Atat1	1.0247	0.002								
Khdrbs2	1.0242	0.000								
Fam124a	1.0231	0.001								
Fmnl2	1.0215	0.000								
Car12	1.0203	0.000								
Jakmip2	1.0192	0.001								
Foxp4	1.0163	0.001								
Arhgdig	1.0138	0.011444643								
Pknox2	1.0093	0.000								
Camkk2	1.0089	0.000								
Cdc42ep4	0.9997	0.000								
Polr2l	0.9968	0.000								
Nagk	0.9865	0.002								
Kif5a	0.9779	0.002								
Lsamp	0.9775	0.000								
Ccne1	0.9748	0.002								
Pgam2	0.9741	0.001								
Pak7	0.9725	0.001								
Ctnnd2	0.9631	0.000								
Rap1gap2	0.9614	0.000								
Car10	0.9550	0.003935846								
Nuak1	0.9522	0.002								
Nln	0.9515	0.009154044								
Stmn1	0.9489	0.000								
Nav1	0.9453	0.000								
Galnt14	0.9417	0.001								
Prkcb	0.9397	0.000								
Edil3	0.9388	0.000								
Fxyd2	0.9384	0.001								
Rab33a	0.9362	0.00554983								
Plppr1	0.9271	0.000								
Cask	0.9266	0.003								
Fxyd6	0.9235	0.001								
Dph6	0.9163	0.003497749								
Ash2l	0.9089	0.001								
Tubb4a	0.9070	0.002								
Rell2	0.9048	0.002								
1700037H04Rik	0.9033	0.010047321								
Stmn2	0.8999	0.024793988								
Rab3c	0.8977	0.010789897								
Sema4d	0.8966	0.003								
Pvalb	0.8864	0.042903762								
Lpl	0.8812	0.000								
Lars2	0.8754	0.003								
Lsm6	0.8744	0.000								
Mrpl17	0.8735	0.001								
Nrsn1	0.8729	0.001								

Slc16a2	0.8728	0.000									
Pde2a	0.8713	0.000									
Fam184a	0.8711	0.005821335									
E330009J07Rik	0.8663	0.009786856									
Utp18	0.8635	0.018129242									
Phlpp2	0.8629	0.009133483									
Gtf3a	0.8613	0.004682095									
Plch2	0.8547	0.01397543									
Gm42418	0.8536	0.000									
Asic4	0.8507	0.02112012									
Hecw1	0.8495	0.000									
Pwp1	0.8427	0.018661822									
Fcho1	0.8427	0.018686211									
4833422C13Rik	0.8411	0.028053682									
Armcx4	0.8306	0.003501682									
Mapre1	0.8304	0.000									
Pacs1	0.8242	0.027719616									
Lrfn4	0.8215	0.013719609									
Begain	0.8139	0.002									
Sdc3	0.8133	0.001									
Npy	0.8115	0.030931006									
Nnat	0.8104	0.027498954									
Sptlc2	0.8092	0.043506104									
Rgs17	0.8080	0.006449633									
Aida	0.8078	0.034003466									
Dcx	0.8071	0.034358856									
Chd5	0.8041	0.017890576									
Otulinl	0.8005	0.029729934									
Nkrf	0.7995	0.022144189									
Kif26b	0.7995	0.003									
Celf4	0.7933	0.000									
Tmem181a	0.7925	0.000									
2900079G21Rik	0.7915	0.001									
Pdzd4	0.7908	0.003718178									
Smim20	0.7901	0.014517648									
Nol8	0.7897	0.03706211									
Prss12	0.7861	0.026983892									
Dgcr6	0.7852	0.002									
Caln1	0.7848	0.022114045									
Acadvl	0.7826	0.047618816									
C330007P06Rik	0.7788	0.021656348									
Gstm7	0.7771	0.001									
Ndufaf2	0.7760	0.007493142									
Mapk8	0.7719	0.007879345									
Cmtm4	0.7703	0.021119565									
Kcnk2	0.7703	0.012359606									
Psme1	0.7701	0.003									
Hrk	0.7698	0.000									
Ralyl	0.7668	0.000									
Trabd	0.7661	0.016375097									
Kctd10	0.7658	0.028327787									
Gda	0.7606	0.000									
Spred1	0.7606	0.004419414									
Ccnt1	0.7560	0.037075881									
Fhl1	0.7555	0.016714256									
Capn5	0.7554	0.030638387									
Smyd2	0.7553	0.000									
Fndc10	0.7502	0.02428107									
Sobp	0.7475	0.000									
Gzf1	0.7473	0.04045818									
Poc5	0.7443	0.007139203									

Armc10	0.7439	0.020924173								
Cdh9	0.7437	0.000								
Ctxn2	0.7413	0.000								
Srcin1	0.7408	0.004090447								
Psmd1	0.7394	0.004224535								
Dyrk2	0.7351	0.028738836								
Vps37d	0.7349	0.033228963								
Fabp7	0.7334	0.042982585								
Rtl8b	0.7329	0.00599427								
Brf1	0.7329	0.041269717								
Vstm2b	0.7323	0.000								
Actn4	0.7321	0.002								
Apcdd1	0.7320	0.007719124								
Mrpl9	0.7293	0.021693206								
Lztfl1	0.7293	0.026188172								
Ahsa2	0.7234	0.022750832								
Sp3os	0.7227	0.026949988								
Necab3	0.7200	0.000								
Yaf2	0.7195	0.000								
Sox11	0.7188	0.000								
3110039M20Rik	0.7173	0.041566202								
Zbtb7a	0.7159	0.026357877								
Angel2	0.7136	0.001								
Capn3	0.7134	0.000								
Nt5dc2	0.7133	0.001								
Lypd1	0.7101	0.000								
Inpp1	0.7085	0.044496375								
Akap9	0.7081	0.014351737								
Ptprd	0.7081	0.02359307								
Rnf5	0.7068	0.004107462								
Pcnx3	0.7036	0.046697758								
Rpp14	0.6998	0.011021425								
Ssx2ip	0.6986	0.000								
Bend6	0.6974	0.032115552								
Pcdh9	0.6972	0.025537119								
Tgfbr1	0.6958	0.017574741								
Ttll7	0.6925	0.04495084								
Rusc2	0.6903	0.003								
Rap2b	0.6888	0.000								
Vipas39	0.6881	0.019851398								
6030458C11Rik	0.6790	0.021319753								
Grwd1	0.6789	0.048152426								
Lars	0.6787	0.02423961								
Flnb	0.6776	0.024314929								
Stau2	0.6743	0.004483767								
Thumpd3	0.6731	0.022986964								
Cadm1	0.6720	0.000								
Oxr1	0.6720	0.00417082								
Lrrc4c	0.6712	0.031204335								
Vezt	0.6665	0.021070104								
Csad	0.6664	0.042606982								
Cdh11	0.6635	0.027750334								
Cacnb2	0.6634	0.011230141								
B2m	0.6583	0.023218191								
B3galt1	0.6555	0.037569528								
Slc37a3	0.6543	0.028351505								
Cnot10	0.6540	0.011455278								
Pcbp4	0.6502	0.015789301								
Drosha	0.6467	0.003644621								
Larp6	0.6451	0.033329706								
Tulp4	0.6419	0.006238049								

Drg2	0.6383	0.016392549								
Tax1bp1	0.6354	0.003155237								
Spon1	0.6320	0.00352977								
Tob1	0.6312	0.011550025								
Fam92a	0.6258	0.003853558								
Syngr1	0.6236	0.002								
Rasd2	0.6229	0.002								
Rgs14	0.6214	0.001								
Larp1	0.6209	0.028331201								
Cox16	0.6196	0.038612629								
Syt16	0.6131	0.008888674								
Mycl	0.6104	0.002								
Dynlt3	0.6097	0.008309511								
D430041D05Rik	0.6063	0.000								
Nkain2	0.6054	0.009138647								
Marcksl1	0.6052	0.000								
Sh3bgrl	0.6040	0.000								
Hdac11	0.6039	0.028442958								
Pitpnm1	0.6023	0.029109052								
Tmem35a	0.6020	0.006201305								
Dock9	0.6015	0.00569013								
Usp46	0.5992	0.037671412								
Cnpy3	0.5945	0.033867878								
Ak1	0.5934	0.016799791								
Matn2	0.5891	0.011546523								
Rhbdd2	0.5869	0.046508876								
Nhp2	0.5867	0.003138693								
AW549877	0.5861	0.045801432								
Slc39a10	0.5850	0.042777369								
Pkd1	-0.5852	0.002								
Mtdh	-0.5853	0.014244317								
Dalrd3	-0.5858	0.007740902								
Gad1	-0.5875	0.001								
Uba52	-0.5885	0.014868472								
Pfkp	-0.5886	0.04587843								
Insyn1	-0.5924	0.00384908								
Prickle1	-0.5955	0.003								
Atf7ip	-0.5956	0.01889148								
Ift20	-0.5969	0.000								
B3galt5	-0.5973	0.046796052								
Baz1b	-0.5986	0.001								
Vps29	-0.6021	0.000								
Upf1	-0.6026	0.045816676								
Gabra5	-0.6034	0.000								
Fbxo33	-0.6063	0.023179827								
Slc2a13	-0.6080	0.000								
Cttnbp2	-0.6124	0.000								
Mgat3	-0.6151	0.000								
Jund	-0.6213	0.021071143								
Magi1	-0.6252	0.010366296								
Pgm1	-0.6254	0.032238888								
Prr13	-0.6268	0.041825386								
Nebl	-0.6274	0.044801955								
Camk1d	-0.6292	0.003								
Crlf1	-0.6337	0.000								
Myo5b	-0.6341	0.002								
Ube2j1	-0.6356	0.041606505								
Ppfia2	-0.6357	0.000								
Sik3	-0.6374	0.010070424								
Kcnab2	-0.6421	0.000								
Ypel4	-0.6429	0.001								

Flywch2	-0.6446	0.042623807								
Lman2	-0.6465	0.038888367								
Rbbp5	-0.6490	0.039248808								
Plxna2	-0.6503	0.027815522								
Csnk2a2	-0.6504	0.037082627								
Nedd4l	-0.6520	0.000								
Anxa11	-0.6534	0.000								
Slc35f3	-0.6559	0.009233036								
2310009A05Rik	-0.6565	0.016714745								
Acot5	-0.6610	0.040464426								
Chmp4b	-0.6623	0.000								
Atp1b2	-0.6630	0.01857407								
Ppif	-0.6690	0.029052066								
Sv2c	-0.6734	0.019685759								
Stub1	-0.6747	0.000								
Ubl3	-0.6757	0.000								
Cpeb1	-0.6773	0.000								
Prkca	-0.6794	0.006775015								
Ptprj	-0.6834	0.000								
Plekhn1	-0.6835	0.013662374								
Arpp19	-0.6856	0.014905744								
Zfp24	-0.6878	0.03664349								
Cebpb	-0.6885	0.000								
Pkp2	-0.6886	0.045880649								
Atxn1	-0.6950	0.003								
Ankrd45	-0.6976	0.001								
Ip6k2	-0.6991	0.024508174								
Map3k4	-0.6992	0.003658767								
Tceal6	-0.7007	0.000								
Cyp7b1	-0.7030	0.002								
Slit1	-0.7038	0.000								
Orai2	-0.7063	0.000								
Tacc1	-0.7136	0.000								
Elmo2	-0.7147	0.000								
Plbd2	-0.7159	0.003								
Ddit4	-0.7186	0.001								
Rapgef5	-0.7230	0.000								
Grasp	-0.7238	0.000								
Sfswap	-0.7305	0.000								
Src	-0.7384	0.001								
Srebf2	-0.7406	0.01019753								
Snhg11	-0.7410	0.022714471								
Parp1	-0.7430	0.000								
Lingo3	-0.7438	0.004730058								
Adcy1	-0.7465	0.000								
Ippk	-0.7470	0.000								
Ipo8	-0.7490	0.016416741								
Parva	-0.7500	0.04494727								
Pitpnm2	-0.7509	0.000								
Gm10419	-0.7544	0.000								
Zfp277	-0.7545	0.042409862								
Napepld	-0.7591	0.000								
Lgi3	-0.7596	0.001								
Drd5	-0.7604	0.036805415								
Arxes1	-0.7608	0.000								
Pptc7	-0.7623	0.029019334								
Rtn4rl1	-0.7630	0.000								
Rhob	-0.7638	0.000								
Zfp189	-0.7667	0.042449114								
Diaph2	-0.7691	0.044234584								
Chic1	-0.7712	0.016692528								

Itch	-0.7731	0.006812748								
Cnnm3	-0.7743	0.035370705								
Chgb	-0.7749	0.000								
Galt	-0.7753	0.040404778								
Btg3	-0.7772	0.009775512								
Btbd9	-0.7774	0.000								
Jph1	-0.7805	0.000								
Dgkb	-0.7864	0.001								
Slc24a2	-0.7867	0.000								
Errfi1	-0.7877	0.02209735								
Sez6l2	-0.7913	0.000								
Hpdl	-0.7928	0.015945011								
Trim2	-0.7946	0.000								
Cyth3	-0.7947	0.010086878								
Slc7a4	-0.7955	0.000								
Fgfr1	-0.7968	0.019199683								
Tnip2	-0.8001	0.017183536								
Mamld1	-0.8043	0.001								
Smoc2	-0.8082	0.000								
Ptpn3	-0.8131	0.012290037								
Trpm3	-0.8152	0.000								
Arhgap12	-0.8179	0.017312566								
Plekha5	-0.8187	0.005081408								
Hs6st1	-0.8189	0.005499279								
2900026A02Rik	-0.8222	0.021641583								
Glce	-0.8251	0.025497118								
Arhgef28	-0.8265	0.015330887								
Ciao2b	-0.8347	0.003601504								
Glo1	-0.8363	0.000								
Eif1b	-0.8402	0.000								
Dusp11	-0.8501	0.005943465								
Irs2	-0.8537	0.001								
Bambi	-0.8540	0.006454845								
Srxn1	-0.8569	0.008164654								
Cadm3	-0.8660	0.001								
Osbp2	-0.8670	0.000								
Plekha2	-0.8777	0.000								
Csdc2	-0.8821	0.000								
Frmd4b	-0.8869	0.007148754								
Rnf19a	-0.8871	0.000								
Scn3b	-0.8879	0.000								
Pdp1	-0.8883	0.001								
Fnip2	-0.9011	0.003641878								
1810026B05Rik	-0.9054	0.009176606								
Ccdc28b	-0.9107	0.003647545								
Fam102a	-0.9125	0.008160932								
Prkd1	-0.9171	0.010530896								
Gprc5b	-0.9174	0.000								
Foxo3	-0.9297	0.008088742								
Snhg9	-0.9299	0.005777656								
Nudt13	-0.9362	0.012747334								
Wipf3	-0.9408	0.000								
Snta1	-0.9459	0.00327063								
Bdnf	-0.9484	0.000								
Nr3c2	-0.9511	0.000								
Rimbp2	-0.9526	0.000								
Lrp12	-0.9584	0.001								
Ryr1	-0.9590	0.003								
Pls3	-0.9611	0.000								
Arhgap39	-0.9652	0.000								
Igflr1	-0.9703	0.010313367								

Rflnb	-0.9737	0.006115276								
Lratd1	-0.9750	0.000								
Mmp17	-0.9778	0.000								
Rgs4	-0.9838	0.012672317								
Carmil1	-0.9898	0.007047664								
Cemip	-0.9928	0.000								
Cabp7	-0.9984	0.000								
Rnh1	-1.0081	0.001								
Selenom	-1.0151	0.000								
Htr4	-1.0154	0.006108198								
Trpc4	-1.0279	0.010027904								
Kirrel3	-1.0374	0.000								
Gss	-1.0450	0.002								
Dtna	-1.0553	0.000								
Hist1h2bc	-1.0568	0.000								
Zfp715	-1.0671	0.002								
Npy5r	-1.0714	0.004367211								
Gpr22	-1.0717	0.000								
Efhd2	-1.0782	0.000								
Bmp1	-1.0822	0.000								
Zdhhc23	-1.0870	0.003								
Peli2	-1.0876	0.002								
Cebpd	-1.0949	0.000								
Acvr1c	-1.0990	0.002								
Htra4	-1.1008	0.000								
Cabp1	-1.1102	0.002								
Cotl1	-1.1122	0.000								
Slc2a6	-1.1128	0.002								
Rpusd3	-1.1241	0.003								
Fam183b	-1.1263	0.001								
Sesn1	-1.1268	0.000								
Pqlc1	-1.1432	0.000								
Rreb1	-1.1460	0.000								
Pcdh17	-1.1545	0.000								
Ppp1r16b	-1.1571	0.000								
Scg2	-1.1663	0.000								
Maml2	-1.1810	0.001								
Acap2	-1.1885	0.000								
Kcng2	-1.2120	0.000								
Clmp	-1.2195	0.001								
Cidea	-1.2217	0.001								
Galnt17	-1.2588	0.000								
Dusp14	-1.2741	0.000								
Ahcyl2	-1.2781	0.000								
Spata13	-1.2809	0.000								
Inpp5j	-1.2900	0.000								
Fosl2	-1.2904	0.000								
Npnt	-1.2936	0.000								
Rasal2	-1.2949	0.000								
Cyth4	-1.2951	0.000								
Slc4a4	-1.2976	0.000								
Ksr1	-1.2998	0.000								
Rprm	-1.3091	0.000								
Syndig1	-1.3454	0.000								
St18	-1.3566	0.000								
Ubash3b	-1.3856	0.000								
Net1	-1.3947	0.000								
Slc26a10	-1.3980	0.000								
Nefl	-1.3987	0.000								
Fndc5	-1.4019	0.000								
Mast4	-1.4019	0.000								

Mfsd4a	-1.4208	0.000								
Tnfrsf25	-1.4285	0.000								
Ndst4	-1.4536	0.000								
Sipa1l2	-1.4554	0.000								
Il11ra1	-1.4958	0.000								
Epha10	-1.5103	0.000								
Gm34567	-1.5462	0.000								
Nptx2	-1.5492	0.000								
Tgfa	-1.5650	0.000								
Lurap1l	-1.5652	0.000								
Dhx33	-1.5903	0.000								
Cxcl12	-1.6010	0.000								
Lratd2	-1.6041	0.000								
Npy1r	-1.6069	0.000								
Homer3	-1.6135	0.000								
Bhlhe40	-1.6144	0.000								
Spns2	-1.6229	0.000								
Nefm	-1.6238	0.000								
Inf2	-1.6292	0.000								
Pacsin2	-1.6506	0.000								
Ighm	-1.7043	0.000								
Itga7	-1.7386	0.000								
Hpca	-1.7695	0.000								
Fkbp5	-1.8069	0.000								
Tubb2b	-1.8765	0.000								
Grm2	-1.9423	0.000								
Lrrc10b	-1.9592	0.000								
Dsp	-1.9980	0.000								
Hectd2	-2.0058	0.000								
Penk	-2.0111	0.000								
Jdp2	-2.0191	0.000								
Pdzd2	-2.0784	0.000								
Olfml2b	-2.1977	0.000								
Nrip2	-2.2060	0.000								
Spsb1	-2.2166	0.000								
Il1r1	-2.3078	0.000								
Bcl6	-2.3380	0.000								
Golm1	-2.5055	0.000								
Robo3	-2.5568	0.000								
Calb1	-2.7301	0.000								

	A	B	C	D	E	F	G	H	I	J
1	**Supplementary Table 4- IPA -comparison- upstream regulator**									
2										
3	© 2000-2024 QIAGEN. All rights reserved.									
4	Analysi	Upstream Regulat	Expr False I	Expr Log Ratio	Expr p-value	Molecule Type	Predicted A	Activation z	p-value of overla	Target Molecules in Dataset
5	CA2 - r	IGF1R	0.00024	-2.355	0.00000437	transmembrane	Activated	2	0.274	CABP1,ETV1,FHL2,IGFBP4,Scd2,SCG2
6	CA2 - r	RXRA	0.328	0.864	0.0819	ligand-depender	Activated	2	0.561	FLRT3,PLCL1,TGFB2,TMEM215
7	CA2 - r	INSR				kinase	Activated	2.219	0.523	ACOT13,ATP2B1,ETV1,FHL2,IGF1R,KIT,LDB2
8	CA2 - r	DNMT3B				enzyme	Activated	2	0.0563	ADCY5,ADORA1,CACNA1C,PRKCB
9	CA3 - r	BHLHE40				transcription reg	Activated	2.236	0.0923	AK4,B2M,HOMER1,KIT,PCDH7
10	DG - ne	BHLHE40	8.14E-09	-1.614	1.46E-10	transcription reg	Activated	2.121	1	AK4,B2M,BDNF,CDH11,GDA,NTRK3,PFKP,PSME1
11	DG - ne	NR4A1				ligand-depender	Activated	2.236	0.202	CIDEA,LPL,MAPK8,MARCKSL1,NPY,PGAM2,PGM1,RAP1GAP2
12	DG - ne	TGFBR2				kinase	Activated	2.36	0.00208	ADCY1,BHLHE40,Cd200,DDIT4,DUSP14,EPOP,GPLD1,IRS2,PGM1,SV2C
13	CA2 - r	TRAPPC1	0.571	-0.42	0.258	other	Inhibited	-2	0.082	HSPA1A,Hspa1b,HSPA2,LMAN2
14	CA2 - r	PRKAA1	0.639	-0.406	0.336	kinase	Inhibited	-2.828	0.000904	GRIA3,Gsta4,IGFBP4,LMO4,NAP1L5,SERPINI1,SMAD3,TTYH1
15	CA2 - r	PRKAA2	0.332	-0.85	0.0842	kinase	Inhibited	-2.828	0.00108	GRIA3,Gsta4,IGFBP4,LMO4,NAP1L5,SERPINI1,SMAD3,TTYH1
16	CA2 - r	ESR2				ligand-depender	Inhibited	-2.646	0.113	ARNT2,C3orf80,CABP1,CADM3,CBFA2T3,FKBP5,PTGS2
17	CA2 - r	NOTCH3				transcription reg	Inhibited	-2.2	0.0286	CACNA1C,CCN3,ERRFI1,HSPA1A,Hspa1b
18	CA2 - r	CYP1B1				enzyme	Inhibited	-2	0.131	ARNT2,C11orf87,CACHD1,FLRT3
19	CA3 - r	PKD1	0.346	-0.683	0.0622	ion channel	Inhibited	-2	0.0802	MCUB,PCDH7,PCDH9,TTR
20	DG - ne	TRIM38				enzyme	Inhibited	-2	0.297	FABP7,LPL,MPZL1,SDC3
21	DG - ne	SLCO1C1				transporter	Inhibited	-2.36	5.3E-10	ATP1B2,ATP2B4,Cd200,CKMT1B,FXYD6,GAD1,NEFL,PITPNM1,RASAL1,SREBF2
22	DG - ne	HUWE1	0.798	0.059	0.621	transcription reg	Inhibited	-2	0.297	ACADVL,CIDEA,GPRC5B,Snhg11
23	DG - ne	MEN1	0.924	-0.045	0.846	transcription reg	Inhibited	-2.095	0.000018	ELAVL3,FABP7,FKBP5,GFAP,IRS2,NOS1,NRSN1,PTN,SLIT2,SOX11
24	DG - ne	ISL1				transcription reg	Inhibited	-2.2	0.0933	AJAP1,DCC,LHX2,NPY,NTRK3
25	DG - ne	GNAS	0.745	0.02	0.544	enzyme	Inhibited	-2.121	0.0529	ALCAM,BCL6,Cd200,FOSL2,GFOD1,PPP1R16B,PRKCB,Tubb2b
26	DG - ne	CREM				transcription reg	Inhibited	-2.236	0.0153	BCL6,BHLHE40,CEBPB,DUSP14,ERRFI1,IRS2,PDYN
27	DG - ne	ELAVL1	0.414	-0.268	0.189	other	Inhibited	-2.236	0.414	COX16,DNMT3A,FABP7,IL1R1,LPL
28	DG - ne	CREB1	0.988	-0.009	0.973	transcription reg	Inhibited	-2.236	0.0351	BDNF,BHLHE40,CEBPB,DUSP14,ERRFI1,IRS2
29	DG - ne	PIAS1	0.218	0.411	0.0685	transcription reg	Inhibited	-2	0.0329	BDNF,GABRA5,GRM2,UNC13C
30	DG - ne	BMAL1				transcription reg	Inhibited	-2	0.0989	DGAT2,LPL,NPY,PDP1
31	DG - ne	SLC16A2	0.000131	0.873	0.00000671	transporter	Inhibited	-2.714	1.62E-10	ATP1B2,ATP2B4,Cd200,CKMT1B,FXYD6,GAD1,NEFL,PITPNM1,RASAL1,SREBF2
32	DG - ne	DIO2				enzyme	Inhibited	-2.63	0.0209	Arxes1/Arxes2,CARMIL1,CYTH3,DDIT4,MARCKSL1,NEFM,PDP1,PENK,SEMA7A
33	CA2 - r	FFAR3				G-protein couple			0.0132	NECAB2,SPOCK1
34	CA2 - r	JINK1/2 (family)				group			0.0255	PARP1,PTGS2
35	CA2 - r	CREB (family)				group		-0.447	0.0269	CACNA1C,CLMN,FOXP1,LMO4,PRKCA,PTGS2,SLIT2
36	CA2 - r	ERK1/2 (family)				group		-0.179	0.0326	CACNA1C,DAPK1,EGR1,PTGS2
37	CA2 - r	DUB (family)				group			0.0357	MYLIP
38	CA2 - r	CALMODULIN (fa				group			0.0396	RPH3A,SEZ6,TSPAN17
39	CA2 - r	INTEGRIN (comp				complex			0.018	TGFB2
40	CA2 - r	TMED10	0.227	0.781	0.0416	other			0.018	TMED9
41	CA2 - r	HSPA1A	0.0283	-1.509	0.0017	other			0.0357	Hspa1b
42	CA2 - r	AGTR1				G-protein couple			0.018	PTGS2
43	CA2 - r	LGALS7				other			0.0357	EGR1
44	CA2 - r	Nme2	0.991	0.004	0.982	kinase			0.018	PTGS2
45	CA2 - r	Sult1e1				enzyme			0.0132	IGFBP4,PTGS2
46	CA2 - r	PSMB11				peptidase		1.791	0.000416	HSPA1A,Hspa1b,KIT,NCAM1,OLFML2B,PRKCB,PTGS2,TGFB2,VIT
47	CA2 - r	MCPH1				other			0.0189	INKA2,PLK2
48	CA2 - r	EOMES				transcription reg		1	0.0254	BHLHE22,KCNQ3,MMP17,PCDH19
49	CA2 - r	BDNF	0.967	0.027	0.928	growth factor			0.0124	BHLHE40,DUSP6,EGR1,FSCN1,LOC728392,TMSB10
50	CA2 - r	TLX3				transcription reg			0.0189	GABRA1,GRIA3
51	CA2 - r	NANOG				transcription reg			0.0179	CBFA2T3,POLE4,VIT
52	CA2 - r	MRTFB	0.00853	0.799	0.000368	transcription reg		0.816	0.0425	ABLIM1,ALCAM,CACNA1C,EGR1,EPHA7,SLIT2
53	CA2 - r	PDZD11	0.267	0.917	0.0556	other			0.0357	NECTIN1
54	CA2 - r	TSPAN33				other			0.018	PTGS2
55	CA2 - r	TTL				enzyme			0.018	CLIP1
56	CA2 - r	NIPBL				transcription reg			0.0357	POLE4
57	CA2 - r	EBF2				transcription reg			0.00691	CIDEA,LGI1,SHISA6
58	CA2 - r	TFCP2L1				transcription reg			0.0357	POLE4
59	CA2 - r	HMG20A	0.682	0.363	0.393	transcription reg		-1.342	0.0442	CHST2,ERRFI1,IGFBP4,LY6E,SLC7A1
60	CA2 - r	LBX1				transcription reg			0.0357	FHL2
61	CA2 - r	ESYT3				other			0.0357	PCSK2
62	CA2 - r	UBR2				enzyme			0.0496	IGFBP4,LY6E
63	CA2 - r	STRA8				other			0.0267	CNTNAP2,SYCE2,ZNF804A
64	CA2 - r	SLCO1C1				transporter		-1.342	0.0162	CIDEA,CNTN1,PCP4,RASAL1,SIRPA
65	CA2 - r	KDM4C				enzyme		-1	0.00427	EGR1,PRDX6,PRKCB,TTYH1
66	CA2 - r	TMEM184B	0.305	0.707	0.0717	other			0.00992	GABRA1,PPP1R9B,RGS14
67	CA2 - r	ZFP91	0.686	-0.316	0.399	transcription reg			0.0255	Scd2,STMN1
68	CA2 - r	SLC30A7				transporter			0.0189	EPHX4,FABP3
69	CA2 - r	LINC01159				other			0.018	POU3F3
70	CA2 - r	TSHZ3				transcription reg		-1.698	0.000368	ADCY1,BHLHE22,HS3ST4,KCTD12,LDB2,RSPO2
71	CA2 - r	TRIM32	0.957	-0.016	0.901	transcription reg			0.018	DTNBP1
72	CA2 - r	NEK7				kinase			0.0107	LMO4,PTGS2
73	CA2 - r	ASCL1				transcription reg		0.447	0.0114	CACNA2D3,EPHB3,POU3F1,PTPRD,SEZ6
74	CA2 - r	N6AMT1				enzyme		-0.128	0.0476	DACH1,EPHB3,MPP3,PTPRO,SHISA2,TMEM132A
75	CA2 - r	PTF1A				transcription reg		0	0.0151	CACNA2D3,CADPS2,POU3F1,SEZ6
76	CA2 - r	PLEKHA7				other			0.0357	NECTIN1
77	CA2 - r	RREB1				transcription reg			0.018	TUBB3
78	CA2 - r	FAR1	0.00351	1.34	0.000119	enzyme			0.0496	KCNH3,Serpina3n (includes others)
79	CA2 - r	TCF7L1				transcription reg		-0.447	0.00807	ARHGEF26,CCDC3,KIT,POU3F1,PYCR2
80	CA2 - r	FITM2				enzyme			0.0368	CACNA1C,CACNA2D1
81	CA2 - r	MEN1	0.988	0.014	0.971	transcription reg		-1.912	0.0113	ETV1,FKBP5,GABRB3,NCAM1,NEGR1,SLIT2,TUBB3
82	CA2 - r	STAG1				other			0.00842	PCDH17,PCDH7
83	CA2 - r	EIF2AK3				kinase		0.453	0.0304	EGR1,ETS2,LMO4,SLC7A1,TMED9,WFS1
84	CA2 - r	NFATC3				transcription reg			0.0409	EGR3,GAP43
85	CA2 - r	ATF (family)				group			0.018	PTGS2
86	CA2 - r	FEV				transcription reg		1.89	0.000745	CNTN1,EPHA7,GRIA3,NCALD,NR3C1,SCG2,SLIT2
87	CA2 - r	PGF				growth factor			0.0291	EGR1,ETS2
88	CA2 - r	PTEN	0.941	-0.069	0.853	phosphatase		1.77	0.121	CADM3,DLAT,ETS2,GPR137C,KIT,LY6E,MMP17,NCAM1,NTSR2,PTGS2
89	CA2 - r	ZFHX3				transcription reg			0.0286	HCN1,NTSR2,SLIT2,Tubb2b,TUBB3
90	CA2 - r	MRTFA	0.071	-0.633	0.00679	transcription reg		1.134	0.0217	ABLIM1,ALCAM,CACNA1C,EGR1,EPHA7,FABP3,SLIT2
91	CA2 - r	CNGA2				ion channel			0.0357	CYP26B1
92	CA2 - r	MKNK1				kinase			0.0365	CPLX2,EGR1,GAP43,HS6ST2,HSPA2,KCTD12,Scd2
93	CA2 - r	DNMT1				enzyme			0.00119	ADCY5,ARG2,Hspa1b,IGF1R,INPP5F,NCAM1,PRKCA,PTGS2,Tubb2b,TUBB3
94	CA2 - r	RGS9				enzyme			0.018	GNB5
95	CA2 - r	CAMKK1	0.856	0.112	0.675	kinase			0.018	FBXW7
96	CA2 - r	MAT1A				enzyme			0.0452	EGR1,PRDX6
97	CA2 - r	SMARCA5	0.545	0.588	0.233	transcription reg		-0.378	0.00755	ETS2,FKBP5,GRIA3,IGFBP4,KIT,PLK2,TUBB3
98	CA2 - r	CTNNBIP1				other			0.018	TUBB3

	A	B	C	D	E	F	G	H	I	J
99	CA2 - r	HOXA10				transcription reg		-1.633	0.00819	DBP,KCNAB1,NBL1,NCAM1,PCP4,Scd2
100	CA2 - r	RGS6				enzyme			0.0357	GNB5
101	CA2 - r	TCOF1				transcription reg			0.0391	ASAP1,FABP3,HNRNPDL,UPF2
102	CA2 - r	ADAM17				peptidase		-1.342	0.0298	ERRFI1,Hspa1b,MARCKS,NBL1,PEA15
103	CA2 - r	NPTN	0.4	0.07	0.121	other			0.018	ATP2B1
104	CA2 - r	RCAN1				other			0.0452	CBFA2T3,PTGS2
105	CA2 - r	GNAS	0.912	-0.009	0.783	enzyme		-1.134	0.0197	ALCAM,ETS2,KCTD12,PLK2,PRDX6,PRKCB,Tubb2b
106	CA2 - r	CREM				transcription reg			0.0298	BHLHE40,EGR1,ERRFI1,GPD2,MARCKS
107	CA2 - r	LEP				growth factor		-1.98	0.407	DUSP6,FABP3,FKBP5,GAP43,PTGS2,Scd2
108	CA2 - r	SLC32A1	0.59	0.499	0.282	transporter			0.0357	GABRA1
109	CA2 - r	NGFR				transmembrane			0.0107	GRIA3,NCAM1
110	CA2 - r	mir-19 (includes c				microRNA			0.016	ADCY1,CIDEA
111	CA2 - r	mir-204 (includes				microRNA			0.035	ITPR1,NBL1,SMAD3
112	CA2 - r	RAB1A	0.345	0.195	0.0915	enzyme			0.0357	PRKCA
113	CA2 - r	ADCYAP1				other		0.378	0.0008	CACNA2D3,CCBE1,CNTNAP2,EGR1,GAP43,MAN1A1,TTYH1
114	CA2 - r	MRAS	0.117	0.931	0.0142	enzyme			0.00462	DUSP6,ETV1
115	CA2 - r	KIF5B	0.0927	0.917	0.00991	other			0.018	CACNA1C
116	CA2 - r	FST				other			0.035	CIDEA,FABP3,SMAD3
117	CA2 - r	ID3				transcription reg		0.816	0.0375	CBFA2T3,ETS2,FOXP1,G6PC3,IGFBP4,LMO4,PTPN5
118	CA2 - r	WIF1				other			0.0357	FRZB
119	CA2 - r	PLA2G6				enzyme			0.0452	ARG2,PTGS2
120	CA2 - r	SPRY2	0.688	0.406	0.4	other		-1.276	0.0008	CIDEA,DUSP6,ETV1,LMO4,MMD,PTGS2,SPON1
121	CA2 - r	APP	0.923	0.01	0.806	other			0.00011	ATP2B1,FKBP1B,NSMF,NTF3,PLPPR4,POU3F3,PTGS2
122	CA2 - r	LTK				kinase			0.018	IGF1R
123	CA2 - r	ADORA2A				G-protein couple		0.555	0.0413	ATP2B1,EIF2S2,GABRB3,PEA15,TUBB3
124	CA2 - r	ELK4				transcription reg			0.0107	EGR1,EGR3
125	CA2 - r	GNB3				other			0.016	GNB5,PRKCA
126	CA2 - r	CLOCK	0.99	-0.013	0.977	transcription reg			0.0401	ARNT2,BHLHE40,DBP,FKBP5,Hspa1b,NTSR2
127	CA2 - r	ALK				kinase			0.018	IGF1R
128	CA2 - r	RGS11				enzyme			0.018	GNB5
129	CA2 - r	MECP2				transcription reg		0.552	0.0000239	BEX2,FKBP5,FRZB,GABRB3,GSTO1,INPP5A,KCNF1,MMD,NCALD,RSPO2
130	CA2 - r	DNMT3A				enzyme		1.89	0.0269	ADCY5,ADORA1,CACNA1C,CPLX2,PLK2,PRKCB,SLIT2
131	CA2 - r	KCNE3				ion channel			0.0358	CHL1,DUSP6,HSPA1A,PTGS2
132	CA2 - r	NFE2L2				transcription reg		0.758	0.00969	FKBP5,Gsta4,GSTO1,LY6E,MAS1,PCDH7,PFKP,PKIA,PTPRO,TGFB2
133	CA2 - r	TCF20	0.703	0.401	0.426	transcription reg		0	0.000937	BEX2,FRZB,INPP5A,MMD,NCALD,RSPO2
134	CA2 - r	CTCF	0.889	-0.138	0.736	transcription reg		1.633	0.00486	EGR1,ERRFI1,HSPA1A,Hspa1b,NCAM1,PTGS2
135	CA2 - r	SLC2A1				transporter			0.0255	Gsta4,PTGS2
136	CA2 - r	TP63				transcription reg		-0.392	0.00325	BMP3,EGR1,FGF11,FSCN1,HSPA1A,Hspa1b,PRKAG2,SLIT2,SPON1,Tubb2b
137	CA2 - r	KIF3B	0.821	-0.221	0.611	transporter			0.0357	PYCR2
138	CA2 - r	LMNA	0.629	-0.441	0.324	other			0.0114	ADORA1,FHL2,Serpina3n (includes others),TGFB2,TUBB3
139	CA2 - r	BACE1	0.469	0.6	0.17	peptidase		0	0.00567	IGFBP4,ITM2B,KCTD12,Serpina3n (includes others)
140	CA2 - r	STK11	0.686	-0.331	0.397	kinase		-0.707	0.0156	CABP1,GRIA3,NAP1L5,PAPSS1,PTGS2,PYCR2,SERPINI1,TTYH1
141	CA2 - r	RTN4R	0.951	-0.039	0.886	transmembrane			0.0357	PTGS2
142	CA2 - r	SLC7A11				transporter			0.0107	GPT2,PTGS2
143	CA2 - r	IL1A				cytokine			0.0497	NR3C1,PTGS2,Serpina3n (includes others)
144	CA2 - r	USP19	0.833	-0.103	0.64	peptidase			0.0496	FKBP5,NR3C1
145	CA2 - r	MED13				transcription reg			0.0328	CIDEA,GPD2,PGAM2
146	CA2 - r	RB1	0.409	0.576	0.127	transcription reg		0.707	0.0234	DLAT,GPR12,GPR137C,GPT2,GSTO1,IGF1R,IL16,NTSR2,PARP1,PGAM2
147	CA2 - r	MAP3K8				kinase		0.05	0.00437	CHST2,DUSP6,EGR1,EGR3,FKBP5,IER5,IGF1R,PTGS2,SLC39A14,Tubb2b
148	CA2 - r	DIO3				enzyme		1.134	0.0385	CIDEA,FGF11,IER5,ITGA7,PEX5L,SYCE2,TYRO3
149	CA2 - r	HADH				enzyme		-0.333	0.000000554	CABP1,CACNG3,FABP3,HSPA1A,NECAB2,PCP4,PRKCA,RASD2,SCG2
150	CA2 - r	MEF2A	0.111	-0.454	0.013	transcription reg		1.432	0.00331	BEX2,Gsta4,ITGA7,PDE7A,SHISA6
151	CA2 - r	TXNDC5				enzyme			0.0291	PRDX6,Serpina3n (includes others)
152	CA2 - r	IL4R				transmembrane		0.447	0.014	ASIC2,ITPKA,SEZ6,ZDHHC2,ZNF804A
153	CA2 - r	TFF1				other			0.018	PTGS2
154	CA2 - r	YAP1				transcription reg		0.447	0.0378	EGR1,IGF1R,IGFBP4,PARP1,PHACTR2,PTGS2
155	CA2 - r	CHL1	0.0316	0.899	0.00208	other			0.018	NCAM1
156	CA2 - r	SHCBP1				other			0.000632	Hspa1b,HSPA2,KIT
157	CA2 - r	PRDM14				transcription reg			0.018	POLE4
158	CA2 - r	NAA10	0.928	0.108	0.817	enzyme			0.016	CIDEA,FABP3
159	CA2 - r	HTT	0.78	-0.2	0.544	transcription reg			0.000016	CACNA2D3,DBP,GNB5,ITPR1,MAN1A1,PCP4,PRKCB,PTPN5,RGS14,SEZ6
160	CA2 - r	APEX1	0.581	-0.433	0.269	enzyme			0.00326	EGR1,EGR3,PTGS2
161	CA2 - r	TAFAZZIN				enzyme		0.711	0.0304	ANXA11,ARNT2,FOXP1,IGFBP4,IL16,LY6E,PHACTR2,PLK2,PRKCB,PTGS2
162	CA2 - r	ARID1A	0.532	0.49	0.221	transcription reg		-0.905	0.00231	CBFA2T3,Gsta4,HS3ST4,ITGA7,KIT,NCAM1,PARP1,PLCL1,SPOCK1,TUBB3
163	CA2 - r	SCN9A				ion channel			0.0171	C3orf80,OSBPL6,PPM1E,STRIP2
164	CA2 - r	THEM4	0.951	0.066	0.883	enzyme			0.018	PTGS2
165	CA2 - r	SLC16A2				transporter		-0.816	0.0159	CIDEA,CNTN1,IER5,PCP4,RASAL1,SIRPA
166	CA2 - r	GATA1				transcription reg		0.896	0.00358	CBFA2T3,EGR1,ETS2,FOXP1,KIT,LMO4,PLXNA1,PRDM8
167	CA2 - r	NRIP1	0.642	0.391	0.342	transcription reg			0.0093	CIDEA,FABP3,PDK2,PTGS2
168	CA2 - r	IFNG				cytokine		0.488	0.039	ARG2,ARNT2,DAPK1,GNB5,HSPA1A,KCTD12,LY6E,NCALD,PTGS2,SMAD3
169	CA2 - r	HNRNPU	0.56	0.234	0.248	transporter		0.277	0.00714	CYP26B1,DACH1,KCNQ3,TGFB2,TYRO3
170	CA2 - r	DUSP5	4.38E-11	-2.322	5.32E-14	phosphatase			0.0452	EGR1,EGR3
171	CA2 - r	CNR1	0.905	0.07	0.767	G-protein couple		-0.378	0.0126	ADORA1,CDK5RAP3,ERRFI1,IGF1R,NR3C1,PTGS2,SLIT2
172	CA2 - r	AR	0.304	0.756	0.0719	ligand-dependen		-1.311	0.00247	CACNA1C,CADM3,CPNE8,FKBP5,IGF1R,NR3C1,SLC7A4,SMAD3,SYCE2,TUBB3
173	CA2 - r	PAX7				transcription reg		1.067	0.0391	MARCKS,PARM1,PKIA,RNF128
174	CA2 - r	GPX4	0.69	-0.054	0.404	enzyme			0.0496	PTGS2,SLC39A14
175	CA2 - r	CITED2				transcription reg		-1.982	0.554	POU3F1,TMCC3,TMSB10,TTC39B
176	CA2 - r	DDB1	0.814	-0.114	0.603	other			0.0496	EGR1,EGR3
177	CA2 - r	ERBB2				kinase			0.0126	ETV1,HSPA1A,KIT,MAN1A1,SERPINI1
178	CA2 - r	STUB1	0.842	0.108	0.651	enzyme			0.0409	Gsta4,TGFB2
179	CA2 - r	SCLY				enzyme			0.0357	GRIK4
180	CA2 - r	SPIB				transcription reg		-1.342	0.0243	EGR1,HSPA1A,Hspa1b,KIT,MBD2
181	CA2 - r	ELAVL3	0.374	0.432	0.107	other			0.000958	GAP43,TUBB3
182	CA2 - r	DIO2				enzyme			0.0191	EGR1,HSPA1A,Hspa1b,IER5,REM2,SYCE2,ZNF804A
183	CA2 - r	NMI				transcription reg			0.0357	PTGS2
184	CA2 - r	RET				kinase			0.0497	ETV1,NTF3,TGFB2
185	CA2 - r	TRIM54				other			0.0357	FHL2
186	CA2 - r	CHRNA9				transmembrane			0.000632	GABRA1,GABRB3,KCNAB1
187	CA2 - r	IL1B				cytokine		-1.902	0.125	EGR1,ERRFI1,FKBP5,IGFBP4,PTGS2,Serpina3n (includes others),SLC39A14
188	CA2 - r	DYSF				other			0.0233	ARPC2,ERRFI1,HSPA1A,Serpina3n (includes others),TCEAL9
189	CA2 - r	ELK1	0.663	0.345	0.367	transcription reg			0.0291	EGR1,EGR3
190	CA2 - r	DCN				other			0.0368	IGF1R,SMAD3
191	CA2 - r	BAG1	0.305	0.658	0.0724	other			0.00189	FKBP5,Hspa1b
192	CA2 - r	FOXP1	0.0112	1.614	0.00052	transcription reg		0.447	0.000722	CDH9,CDS1,HCN1,MTCL1,SPHKAP
193	CA2 - r	NDP				growth factor			0.042	FABP3,FRZB,SLC7A1
194	CA2 - r	PLN				transporter			0.016	PRKCA,PRKCB
195	CA2 - r	TCF7				transcription reg		0.781	0.0236	ARHGEF26,CCDC3,EGR3,IGF1R,INPP5F,KIT,PARP1,POU3F1,PTPRO,TMEM158
196	CA2 - r	FUS	0.457	0.426	0.163	transcription reg			0.00807	CHL1,EPHB3,GABRA1,GRIA3,SPOCK1

	A	B	C	D	E	F	G	H	I	J
197	CA2 - r	EMD	0.806	-0.255	0.59	other			0.0357	EGR1
198	CA2 - r	FMR1	0.443	0.479	0.148	translation regul		0.992	0.00275	DUSP6,EGR3,GABRA1,GAP43,IGFBP4,MRPL20,PFKP,PFN1,SPON1,SYCE2
199	CA2 - r	TP53				transcription reg		0.639	0.00739	DLAT,FABP3,FKBP5,IGFBP4,IL16,LHX2,PFKP,PFN1,PRDX6,PRKAG2
200	CA2 - r	PDX1				transcription reg			0.0275	GAP43,MAN1A1,PCSK2,SCG2,WFS1
201	CA2 - r	PEX14	0.974	0.033	0.94	transcription reg			0.018	PTGS2
202	CA1 - r	CHD8	0.983	-0.017	0.948	enzyme		-1.131	0.00266	ARPC2,FEZF2,NEFM,TUBB3
203	CA1 - r	CDC14B				phosphatase			0.0417	TUBB3
204	CA1 - r	BECN2				other			0.00847	CNR1
205	CA1 - r	CALMODULIN (fa				group			0.0493	CPNE5,DOC2B
206	CA1 - r	IGLL5				other			0.0498	IGHM
207	CA1 - r	HSPA1A				other			0.0169	Hspa1b
208	CA1 - r	TRPV5				ion channel			0.0335	CALB1
209	CA1 - r	Samsn1				other			0.0389	LMO4,RGCC,SH3BP5
210	CA1 - r	Nme1	0.941	0.021	0.824	kinase			0.0417	PTN
211	CA1 - r	MAFA				transcription reg			0.0466	ADCY1,IGHM,PCSK2,TTR
212	CA1 - r	FICD				enzyme			0.00847	IGHM
213	CA1 - r	Snhg20	0.771	-0.193	0.465	other			0.0417	PARP1
214	CA1 - r	CDHR1				other			0.0498	Serpina3n (includes others)
215	CA1 - r	CC2D1A				transcription reg			0.0498	HTR1A
216	CA1 - r	Mir124a-1hg	0.951	0.033	0.853	other			0.0252	NR3C1
217	CA1 - r	ZNF503				other			0.0111	PENK,PLXND1
218	CA1 - r	ESYT3				other			0.0169	PCSK2
219	CA1 - r	MESP2				transcription reg			0.0335	LMO4
220	CA1 - r	TSHZ3				transcription reg		-1.982	0.00106	ADCY1,FEZF2,LDB2,RSPO2
221	CA1 - r	USP36				peptidase			0.00847	DHX33
222	CA1 - r	TRIM32	0.772	-0.077	0.467	transcription reg			0.00847	DTNBP1
223	CA1 - r	PDE11A				enzyme			0.0169	CACNG2
224	CA1 - r	NEK7				kinase			0.00247	LMO4,RGCC
225	CA1 - r	RREB1				transcription reg			0.00847	TUBB3
226	CA1 - r	RBM47				other			0.0119	CNR1,Gsta4,PENK
227	CA1 - r	TBR1				transcription reg			0.0335	FEZF2
228	CA1 - r	FEV				transcription reg		0	0.00545	HTR1A,NR3C1,SATB1,SCN3B
229	CA1 - r	CHFR	0.887	0.128	0.691	enzyme			0.0252	PARP1
230	CA1 - r	IGF1				growth factor			0.00876	Hspa1b,NEFM,PENK
231	CA1 - r	PRKAA1	0.538	0.427	0.18	kinase		-1	0.0148	DOCK4,Gsta4,HIGD1A,LMO4
232	CA1 - r	PRKAA2				kinase		-1	0.0162	DOCK4,Gsta4,HIGD1A,LMO4
233	CA1 - r	OPRK1				G-protein couple			0.0335	PLXND1
234	CA1 - r	DNMT1	0.359	0.67	0.0719	enzyme			0.0228	CACNG2,CNR1,GRIN3A,Hspa1b,KCNMB4,NEFM,PRKCA,TUBB3
235	CA1 - r	CAMKK1	0.676	-0.131	0.33	kinase			0.00847	FBXW7
236	CA1 - r	CTNNBIP1				other			0.00847	TUBB3
237	CA1 - r	HES1				transcription reg			0.0373	HTR1A,TUBB3
238	CA1 - r	ADAM17				peptidase			0.0483	CRIP2,Hspa1b,NBL1
239	CA1 - r	RUNX1				transcription reg			0.0423	ADD2,ALCAM,LMO4,PARP1
240	CA1 - r	CACNB4	0.113	0.665	0.00882	ion channel			0.0335	CACNB2
241	CA1 - r	DSCAM				other			0.0252	PCDH17
242	CA1 - r	RAB1A	0.757	-0.07	0.446	enzyme			0.0169	PRKCA
243	CA1 - r	STRN	0.73	-0.283	0.406	other			0.0335	NR3C2
244	CA1 - r	MARK4	0.787	-0.125	0.502	kinase			0.00847	MAP4
245	CA1 - r	LRPAP1	0.693	0.155	0.351	other			0.0252	Serpina3n (includes others)
246	CA1 - r	CDC14A				phosphatase			0.0417	TUBB3
247	CA1 - r	CDKN2B				transcription reg			0.0335	ENO1
248	CA1 - r	GPRASP1	0.523	0.128	0.171	transporter			0.00847	CNR1
249	CA1 - r	RNF111	0.522	0.424	0.17	enzyme			0.0335	PMEPA1
250	CA1 - r	DEAF1	0.938	0.082	0.81	transcription reg			0.00847	HTR1A
251	CA1 - r	KLF1				transcription reg			0.0317	ADD2,KLF3
252	CA1 - r	IGFBP2				other			0.0417	PTN
253	CA1 - r	FOLR1				transporter			0.0434	OSBPL1A,PFN1,PIP5K1B
254	CA1 - r	TAC1				other			0.0498	HTR1A
255	CA1 - r	BACE1	0.803	0.18	0.53	peptidase			0.0451	Serpina3n (includes others),TTR
256	CA1 - r	ATF7				transcription reg			0.0498	DUSP5
257	CA1 - r	DISC1				other			0.0252	DTNBP1
258	CA1 - r	TRIM33	0.366	0.449	0.0751	transcription reg			0.0169	Lefty1
259	CA1 - r	MMP8				peptidase			0.0335	NR3C1
260	CA1 - r	PCSK6				peptidase			0.0169	Lefty1
261	CA1 - r	DIO3				enzyme		0.447	0.0165	FXYD6,IER5,JAKMIP1,MC4R,STK32C
262	CA1 - r	TEK				kinase			0.0252	TUBB3
263	CA1 - r	GC				transporter			0.0252	CALB1
264	CA1 - r	CPE	0.332	-0.033	0.0631	peptidase			0.0472	PCSK2,PENK
265	CA1 - r	SACS	0.738	-0.206	0.417	other			0.0417	CALB1
266	CA1 - r	RPTOR	0.9	-0.108	0.718	other		0	0.039	DHCR24,ENO1,HIGD1A,NR3C2
267	CA1 - r	HTT	0.533	-0.334	0.177	transcription reg			0.000699	CA12,CNR1,DBP,FXYD6,PENK,RGS4,Serpina3n (includes others)
268	CA1 - r	PTPN6				phosphatase			0.0472	IGHM,MED14
269	CA1 - r	ATRX	0.908	-0.059	0.74	transcription reg			0.0417	PPP1CC
270	CA1 - r	ARID1A	0.984	-0.016	0.951	transcription reg		-1.633	0.0115	CACNB2,Gsta4,PARP1,SPARCL1,Tle1,TUBB3
271	CA1 - r	SIN3A				transcription reg			0.0283	MAP4,NEFM
272	CA1 - r	SCN9A				ion channel			0.0114	C3orf80,DIRAS2,OSBPL1A
273	CA1 - r	SLC16A2				transporter		0.152	0.0153	FXYD6,IER5,MARCKSL1,NEFM
274	CA1 - r	PRDM1				transcription reg		-1.091	0.00696	Gsta4,IGHM,LRRN1,TTR
275	CA1 - r	GATA1				transcription reg			0.0301	LMO4,PRDM8,SATB1,UAP1
276	CA1 - r	HNRNPU	0.775	-0.078	0.472	transporter			0.0193	CACNG2,DACH1,WNT2
277	CA1 - r	CNR1	0.0495	0.946	0.00248	G-protein couple		1	0.0301	CNR1,NR3C1,PENK,RGMA
278	CA1 - r	FOS	0.227	-0.63	0.0296	transcription reg			0.0211	ADD2,PFN1,PIP5K1B,PMEPA1,RGMA,STMN1
279	CA1 - r	DRD2				G-protein couple			0.0336	PCSK2,PENK,PMEPA1
280	CA1 - r	POU2AF1				transcription reg			0.0259	DAPK1,IGHM,RIPOR2
281	CA1 - r	ITK				kinase			0.0356	CPNE7,CRIM1,SULF2
282	CA1 - r	FOXA1				transcription reg			0.0144	GRIN3A,TTR,UST
283	CA1 - r	ELAVL3	0.753	0.158	0.44	other			0.000000599	GAP43,NEFM,TUBB3
284	CA1 - r	DIO2				enzyme			0.00912	Hspa1b,IER5,MARCKSL1,NEFM,PENK
285	CA1 - r	CDON				other			0.00247	STIM1,TUBB3
286	CA1 - r	VEZF1				transcription reg			0.0252	STMN1
287	CA1 - r	DYSF				other			0.0411	ABRACL,ARPC2,Serpina3n (includes others)
288	CA1 - r	CHD4	0.616	-0.256	0.262	enzyme			0.0114	CPNE7,DAPK1,SCN3B
289	CA1 - r	TXN	0.405	0.314	0.0936	enzyme			0.0335	PPP1R13B
290	CA1 - r	BAG1	0.125	0.554	0.0104	other			0.0335	Hspa1b
291	CA1 - r	FOXP1	0.29	0.451	0.0486	transcription reg		1.342	0.0000214	CDS1,CNR1,KCNT1,RASD1,SPHKAP
292	CA1 - r	TCF7				transcription reg		1.414	0.00954	AGPAT5,DHCR24,DST,PARP1,PENK,PIP5K1B,VSTM2A,Wdr95
293	CA1 - r	FMR1	0.223	0.634	0.0288	translation regul		-1.98	0.0437	CELF4,ENO1,GAP43,PFN1,SPARCL1
294	CA1 - r	TP53				transcription reg		0.366	0.0394	ARPC2,CRIP2,DAPK1,DHCR24,IER5,NEO1,PARP1,PFN1,PTN,STMN1

	A	B	C	D	E	F	G	H	I	J
295	CA3 - r	ADCY (family)				group			0.0147	NR4A2
296	CA3 - r	PKC (family)				group			0.026	NR4A2,PRKCA
297	CA3 - r	CALMODULIN (fa				group			0.0384	SEZ6,TSPAN17
298	CA3 - r	CYP3A (family)				group			0.0434	CYP7B1
299	CA3 - r	HSPA1A	0.125	-0.927	0.00889	other			0.0147	Hspa1b
300	CA3 - r	PSMB11				peptidase		0	0.0135	ADCY2,Hspa1b,KIT,NR4A2
301	CA3 - r	MAFA				transcription reg			0.0303	ADCY2,AQP4,PRKCD,TTR
302	CA3 - r	BDNF	0.813	-0.103	0.508	growth factor			0.0438	ARPP21,LOC728392,RGS4
303	CA3 - r	Snhg20	0.435	-0.546	0.0998	other			0.0363	PARP1
304	CA3 - r	CCAR1	0.475	-0.628	0.121	transcription reg			0.0434	CEBPD
305	CA3 - r	FOXN4				transcription reg			0.0292	NR4A2
306	CA3 - r	CDHR1				other			0.000795	CEBPD,Serpina3n (includes others)
307	CA3 - r	METTL3				enzyme			0.0408	ROBO3,SCN3B,ZNF365
308	CA3 - r	ZBTB20	0.91	-0.056	0.749	transcription reg			0.018	AQP4,NNAT,NR3C1
309	CA3 - r	HRH3				G-protein couple			0.0401	NNAT,Serpina3n (includes others)
310	CA3 - r	CDCA8				other		1	0.00279	DUSP5,MYO1B,MYO5B,SYT12
311	CA3 - r	ISL2				transcription reg			0.0147	EPHB1
312	CA3 - r	Mir124a-1hg	0.9	0.092	0.716	other			0.022	NR3C1
313	CA3 - r	SLCO1C1				transporter			0.0228	FXYD6,PCP4,RASAL1
314	CA3 - r	SOX11				transcription reg			0.0289	MARCKS,NREP
315	CA3 - r	LINC01159				other			0.00737	POU3F3
316	CA3 - r	TSHZ3				transcription reg			0.00704	ADCY2,HS3ST4,RSPO2
317	CA3 - r	USP36				peptidase			0.00737	DHX33
318	CA3 - r	VANGL2				other			0.00737	PRICKLE2
319	CA3 - r	MIAT	0.776	0.231	0.45	other			0.0434	FURIN
320	CA3 - r	TCF7L1				transcription reg			0.0145	ARHGEF26,KIT,NNAT
321	CA3 - r	MEN1	0.00297	1.285	0.0000405	transcription reg		-0.97	0.000503	AHI1,AQP4,ELAVL4,GFRA2,MEN1,SLIT2
322	CA3 - r	EIF2B5	0.847	0.195	0.563	translation regul			0.0156	AQP4,Serpina3n (includes others)
323	CA3 - r	LGI1	0.229	-0.289	0.0251	other			0.0292	KCNA2
324	CA3 - r	TBR1	0.578	0.31	0.199	transcription reg			0.0292	NR4A2
325	CA3 - r	FEV				transcription reg		0.478	0.0000027	NCALD,NR3C1,PCDH9,PYGB,SCN3B,SLC18A2,SLIT2
326	CA3 - r	CHFR				enzyme			0.022	PARP1
327	CA3 - r	BMP10				growth factor			0.025	KIT,NR4A2,Serpina3n (includes others)
328	CA3 - r	ZFHX3				transcription reg			0.0333	BANP,SLIT2,Tubb2b
329	CA3 - r	LEF1				transcription reg		-0.447	0.0323	ARHGEF26,ARRB2,CEBPD,NNAT,TMEM158
330	CA3 - r	CNGA2				ion channel			0.0147	CYP26B1
331	CA3 - r	MKNK1				kinase			0.0387	GAP43,HS6ST2,NREP,RAB15
332	CA3 - r	DAG1	0.512	-0.655	0.143	transmembrane			0.00147	ITGA7,SNTA1
333	CA3 - r	SIM2				transcription reg			0.0292	ROBO3
334	CA3 - r	MSX2				transcription reg			0.0168	AQP4,WNT7B
335	CA3 - r	DLX1				transcription reg			0.0434	POU3F3
336	CA3 - r	ADAM17				peptidase		1	0.00559	CRIP2,FURIN,Hspa1b,MARCKS
337	CA3 - r	DMD	0.895	-0.148	0.704	other			0.000207	AQP4,ARPP21,CAMTA1,GALNT17,MARCKS,RAP1GAP2,SCN3B,SNTA1
338	CA3 - r	GNAS	0.24	0.043	0.0281	enzyme		-0.762	0.0257	ALCAM,NR4A2,RASD1,Tubb2b
339	CA3 - r	RASSF1				other			0.0363	Tubb2b
340	CA3 - r	CTSS				peptidase			0.0351	ITGA7,PRKCD
341	CA3 - r	GATA4				transcription reg			0.0369	EPHB1,KIT,Serpina3n (includes others)
342	CA3 - r	VSX1				transcription reg			0.0434	NETO1
343	CA3 - r	TRIM28	0.287	0.39	0.0418	transcription reg			0.0458	RSPO2,SAMD5,WNT7B
344	CA3 - r	RAB1A	0.958	-0.015	0.894	enzyme			0.000054	PRKCA,PRKCD
345	CA3 - r	STRN	0.731	0.336	0.384	other			0.0292	NR3C2
346	CA3 - r	LRPAP1	0.0815	0.49	0.00449	other			0.022	Serpina3n (includes others)
347	CA3 - r	RCE1				peptidase			0.0367	B2M,HS6ST2
348	CA3 - r	NKX2-1				transcription reg			0.0289	EPHB1,WNT7B
349	CA3 - r	Silc1				other			0.0367	MARCKS,NREP
350	CA3 - r	INHA				growth factor			0.0324	KIT,PCDH7,PRSS23
351	CA3 - r	APP	0.495	0.032	0.132	other			0.00107	FKBP1B,NSMF,POU3F3,SDHD
352	CA3 - r	PPM1B	0.45	-0.384	0.108	phosphatase			0.0434	CEBPD
353	CA3 - r	SUB1	0.907	-0.021	0.736	transcription reg			0.000475	FJX1,KCNA6,KCTD4
354	CA3 - r	CLOCK	0.482	0.569	0.124	transcription reg			0.0197	ACSL4,AK4,Hspa1b,MEN1
355	CA3 - r	TOB1	0.824	-0.24	0.528	transcription reg			0.0335	BANP,SCN3B
356	CA3 - r	TP73				transcription reg		0.788	0.00134	AHI1,B2M,BCAR1,FOCAD,KIT,PKP2,Serpina3n (includes others),TTR
357	CA3 - r	MECP2	0.351	-0.645	0.0644	transcription reg		0	0.0158	INPP5A,NCALD,RSPO2,WNT7B
358	CA3 - r	SLC12A2				transporter			0.0363	LYNX1
359	CA3 - r	THRA	0.693	-0.04	0.327	ligand-depender			0.0303	FKBP1B,PKP2,RGS4,SYT12
360	CA3 - r	RGS4	0.0273	1.087	0.000885	enzyme			0.0315	NELL1,NR4A2,PCDH9
361	CA3 - r	GFER				enzyme			0.0193	ABCD3,SDHD
362	CA3 - r	TCF20	0.937	0.079	0.828	transcription reg		0.447	0.000102	FXYD6,INPP5A,NCALD,RSPO2,WNT7B
363	CA3 - r	ITGA6				transmembrane			0.0363	ITGA7
364	CA3 - r	TP63				transcription reg		0.555	0.0158	BMP3,Hspa1b,PKP2,SLIT2,Tubb2b
365	CA3 - r	BACE1	0.598	0.356	0.221	peptidase			0.0351	Serpina3n (includes others),TTR
366	CA3 - r	ATF7				transcription reg			0.0434	DUSP5
367	CA3 - r	STK11	0.774	-0.306	0.449	kinase		0	0.043	CABP1,KCNA2,NR4A2,SDHD
368	CA3 - r	IL1A				cytokine			0.0453	NR3C1,Serpina3n (includes others)
369	CA3 - r	CARD14				other			0.0434	AQP4
370	CA3 - r	MMP8				peptidase			0.0292	NR3C1
371	CA3 - r	PTPN11	0.943	-0.062	0.852	phosphatase			0.00378	ACSL4,KIT,MARCKS,MXD4
372	CA3 - r	GPD1	0.848	0.204	0.565	enzyme			0.0324	CAMK2N2,CYP7B1,Serpina3n (includes others)
373	CA3 - r	HADH				enzyme		0	0.000725	CABP1,NECAB2,PCP4,PRKCA
374	CA3 - r	IKZF1				transcription reg		-0.152	0.0188	CYP7B1,KIT,MYO1B,SULF2
375	CA3 - r	SHCBP1				other			0.00234	Hspa1b,KIT
376	CA3 - r	PAGR1				other			0.0147	CEBPD
377	CA3 - r	UACA				other			0.0292	CEBPD
378	CA3 - r	SLC25A12	0.907	0.052	0.739	transporter			0.022	SLC18A2
379	CA3 - r	LMNB1				other			0.0351	B2M,HS6ST2
380	CA3 - r	HTT	0.336	-0.5	0.0568	transcription reg			0.00000569	ARPP21,ELAVL4,FXYD6,HOMER1,PCP4,RGS14,RGS4,Serpina3n (includes others),S
381	CA3 - r	ARID1A	0.763	-0.211	0.431	transcription reg		-1.342	0.0242	ARRB2,HS3ST4,ITGA7,KIT,PARP1
382	CA3 - r	RHO				G-protein couple			0.00843	B2M,CEBPD,NCALD,STMN1
383	CA3 - r	SCN9A				ion channel			0.0078	OSBPL1A,RAB15,STRIP2
384	CA3 - r	SCTR				G-protein couple			0.022	AQP4
385	CA3 - r	HNRNPU	0.994	-0.002	0.985	transporter			0.0134	ARPP21,CYP26B1,TYRO3
386	CA3 - r	HLF				transcription reg			0.0363	AQP4
387	CA3 - r	AR				ligand-depender		-1.342	0.0285	GOLM1,HS6ST2,NR3C1,RAB3C,Serpina3n (includes others),SULF2,TMEM158
388	CA3 - r	BRCA1				transcription reg			0.0351	KIT,PARP1
389	CA3 - r	PAX7				transcription reg			0.0156	CRLF1,MARCKS,PRSS23
390	CA3 - r	WRN				enzyme			0.022	PRKCD
391	CA3 - r	NR5A2				ligand-depender			0.00927	CABP1,CEBPD,CYP7B1,Hspa1b
392	CA3 - r	GPX4	0.522	0.061	0.154	enzyme			0.00941	ACSL4,TMX1

	A	B	C	D	E	F	G	H	I	J
393	CA3 - r	FOXA1				transcription reg			0.00991	NR4A2,TTR,WNT7B
394	CA3 - r	CGA				other		1	0.00279	KIT,MXD4,NNAT,PPIB
395	CA3 - r	ELAVL3	0.696	0.241	0.334	other			0.022	GAP43
396	CA3 - r	VEZF1				transcription reg			0.022	STMN1
397	CA3 - r	DYSF				other			0.00445	ARPC2,B2M,PRSS23,Serpina3n (includes others)
398	CA3 - r	CHD4	0.927	-0.06	0.801	enzyme			0.0078	CPNE7,ELAVL2,SCN3B
399	CA3 - r	TXN	0.959	0.021	0.902	enzyme			0.0292	PPP1R13B
400	CA3 - r	BAG1	0.928	0.057	0.805	other			0.0292	Hspa1b
401	CA3 - r	NEDD9				other			0.00633	MACROD2,MAL2,NELL1
402	CA3 - r	CTNNB1	0.958	0.015	0.896	transcription reg		0.243	0.0158	ALCAM,AQP4,CYP7B1,GAP43,KIT,RAB18,SDHD,Tubb2b,WNT7B
403	CA3 - r	SLC25A13				transporter			0.0342	CAMK2N2,CYP7B1,Serpina3n (includes others)
404	CA3 - r	FOXP1				transcription reg			0.00318	CDH9,ELAVL4,RASD1
405	CA3 - r	CDC42	0.793	0.059	0.476	enzyme		0	0.00363	ASAP1,SLIT2,STMN1,SULF2
406	CA3 - r	TCF7				transcription reg		0	0.0145	ARHGEF26,CEBPD,FURIN,KIT,NNAT,PARP1,TMEM158
407	CA3 - r	TP53				transcription reg		0	0.0459	ACSL4,AK4,ARPC2,ARRB2,CRIP2,PARP1,PCDH7,RGS12,SDHD,STMN1
408	CA3 - r	PDX1				transcription reg			0.0324	GAP43,NNAT,PRSS23
409	CA3 - r	LAMB2				enzyme			0.0292	AQP4
410	CA3 - r	LAMC3				other			0.0147	AQP4
411	CA3 - r	ADD2	0.859	-0.119	0.602	other			0.0434	PRKCD
412	CA3 - r	Pick1				other			0.00392	FURIN,SCN3B,Serpina3n (includes others),SYT12,TSPAN17,TYRO3
413	DG - ne	CHD8	0.924	-0.043	0.844	enzyme		0.271	0.0367	DCX,FOXO6,LHX2,NEFM,SEMA7A
414	DG - ne	G PROTEIN ALPH				group		0.378	0.0145	Cd200,GNAZ,GPRC5B,MATN2,NPY,NRSN1,PLS3
415	DG - ne	VEGF (family)				group		-0.64	0.0149	CALB1,CIDEA,CRTAC1,MFSD4A,MYCN,PABPN1
416	DG - ne	CYP3A (family)				group			0.0102	CYP7B1,SREBF2
417	DG - ne	H2bw2				other			0.027	DNMT3A
418	DG - ne	Zfp157				other			0.027	ID2
419	DG - ne	MAFA				transcription reg			0.0114	ADCY1,CHGB,CXCL12,EFNA3,ID2,IGHM,LARS2,LGI3,NOS1,ZNF189
420	DG - ne	IGF2-AS				other			0.00215	BDNF,NTF3
421	DG - ne	HES3				transcription reg		1	0.0284	MPZL1,NEFL,NRSN1,SST
422	DG - ne	CADPS	0.148	0.338	0.0389	other			0.00215	BDNF,SCG2
423	DG - ne	FICD				enzyme			0.027	IGHM
424	DG - ne	EOMES				transcription reg		-0.396	0.0000409	BCL6,CEMIP,CRYM,DUSP14,FAT3,MMP17,NEFL,NEFM,PCDH19
425	DG - ne	BDNF	9.05E-07	-0.948	2.78E-08	growth factor			0.0000296	BDNF,BHLHE40,CEBPB,LYPD1,NECAB3,PENK,PRSS12,RGS4,RPRM,SST
426	DG - ne	ULK4				kinase			0.0182	KIF5A,POC5,TUBB4A
427	DG - ne	TLX3				transcription reg			0.0036	GRIN3A,NPY1R,SST
428	DG - ne	GPAT3				enzyme			0.0102	DGAT2,LPL
429	DG - ne	CDHR1	3.02E-08	1.228	5.98E-10	other			0.0102	CEBPD,GFAP
430	DG - ne	FBXO11	0.94	-0.019	0.88	enzyme			0.027	BCL6
431	DG - ne	PABPC1L				other			0.0102	FGFR1,MAP3K4
432	DG - ne	NOC3L				other			0.027	CCNE1
433	DG - ne	TRIB2	0.0707	-0.681	0.0137	kinase			0.027	CEBPB
434	DG - ne	SIRT6				enzyme		-0.498	0.0262	CIDEA,FOXO3,NAPEPLD,NPNT,SREBF2,TGFBR1
435	DG - ne	ARID5A				transcription reg			0.0402	CEBPB,CEBPD
436	DG - ne	GSDMD				transporter		-1.342	0.0276	CEBPB,ERRFI1,FOSL2,GABRA2,GFAP
437	DG - ne	TSHZ3				transcription reg		0.396	0.0000111	ACVR1C,ADCY1,CRYM,FOSL2,GDF10,NPY,NTRK3,PDE2A,SPSB1
438	DG - ne	UBE3D				enzyme			0.0183	CEBPD,LPL
439	DG - ne	USP36				peptidase			0.027	DHX33
440	DG - ne	CP				enzyme			0.027	BDNF
441	DG - ne	SKIC2				enzyme		1	0.0352	AK4,BHLHE40,DDIT4,PGM1
442	DG - ne	NTNG1	0.0338	-0.544	0.00501	other			0.027	LRRC4C
443	DG - ne	ASCL1				transcription reg		1.195	0.000933	DCC,GAD1,NPY,PDYN,PLXNA2,PTPRD,SEMA7A,SEZ6
444	DG - ne	Sox2ot				other			0.0231	DCX,FABP7
445	DG - ne	FNDC5	0.0000561	-1.402	0.00000259	other			0.0486	CAPN3,RYR1,TGFBR1,WFS1
446	DG - ne	RBM47				other		-0.707	0.000000253	A2ml1,CCNE1,CDK18,CXCL12,EPOP,GSS,Gsta4,NPY1R,PENK,SMOC2
447	DG - ne	NKX2-2-AS1				other		0	0.000568	CHGB,GNAO1,NNAT,SST
448	DG - ne	AMPA RECEPTO				complex			0.027	BDNF
449	DG - ne	HSF1	0.597	-0.246	0.36	transcription reg		1.165	0.0129	B2M,CCNE1,CEBPB,FOXP4,LPL,PDZD2,SLC44A5,TUBB4A
450	DG - ne	FEV				transcription reg		1.155	0.00000229	CHGB,KCTD10,PCDH9,PDYN,PYGB,SATB1,SCG2,SCN3B,SLIT2,SST
451	DG - ne	RGS2	0.454	-0.289	0.222	enzyme		0.378	0.017	CEBPD,CHMP4B,CXCL12,ERRFI1,LPL,RFLNB,SLC35F1
452	DG - ne	IGF1				growth factor		-0.2	0.0458	IRS2,NEFM,PDYN,PENK
453	DG - ne	CCK	0.237	-0.216	0.0775	other			0.027	GFAP
454	DG - ne	MYO5A	0.358	0.208	0.152	enzyme			0.027	NEFL
455	DG - ne	DHX36	0.661	-0.137	0.436	enzyme			0.0322	DSP,DTNA,FHL1
456	DG - ne	BSN	0.0539	-0.424	0.00946	other			0.027	BDNF
457	DG - ne	PRKAA1	0.481	-0.32	0.247	kinase		0.113	0.0105	CHGB,CXCL12,FOXO3,Gsta4,IL1R1,PDYN,RTN4RL1,SPRED1
458	DG - ne	S100B				other			0.014	GFAP,NEFL
459	DG - ne	LEF1				transcription reg		0.832	0.0135	CAPN3,Cd200,CEBPD,DGKH,FGFR1,GNAO1,ID2,KCNJ3,PENK,ZNF467
460	DG - ne	SRD5A2				enzyme			0.0284	IRS2,LPL
461	DG - ne	FGF20				growth factor			0.027	MAP3K4
462	DG - ne	PRKAA2				kinase		0.308	0.0038	CHGB,CXCL12,FOXO3,Gsta4,IL1R1,NOS1,PDYN,RTN4RL1,SPRED1
463	DG - ne	MKNK1	0.185	-0.461	0.0527	kinase		1.414	0.0445	APC,ATAT1,CELF4,FLNB,GNAO1,ID2,KIF5A,NELL2,RNH1
464	DG - ne	TLX1				transcription reg			0.000993	GRIN3A,NPY1R,SST
465	DG - ne	COL6A3				other			0.027	WWC1
466	DG - ne	SNTA1	0.00327	-0.946	0.000289	other			0.014	DTNA,NOS1
467	DG - ne	DNMT1	0.407	-0.295	0.184	enzyme			0.0000489	ASH2L,DCC,KIF5A,NEFL,NOS1,PDYN,RBBP5,SCN3A,TNR,Tubb2b
468	DG - ne	KLF12				transcription reg			0.027	BTG3
469	DG - ne	NEFH				other			0.027	NEFL
470	DG - ne	EFNA5				kinase			0.0183	BDNF,NOS1
471	DG - ne	SMARCA5	0.906	0.047	0.813	transcription reg		-1.414	0.0203	ATP2B4,B2M,BCL6,FKBP5,GPRC5B,MAST4,NLN,SYNGR1
472	DG - ne	GCK				kinase			0.014	IRS2,NPY
473	DG - ne	DAG1	0.319	0.294	0.123	transmembrane			0.000993	ITGA7,NOS1,SNTA1
474	DG - ne	TAF4				transcription reg			0.00323	BCL6,BDNF,CARMIL1,CDH11,ID2,PENK,PTN,WWC1
475	DG - ne	ITGB1				transmembrane		0.239	0.0261	BMP1,GFAP,IGHM,ITGA7,MATN2,SEMA5B,SPON1
476	DG - ne	KCNK9	0.182	-0.538	0.0515	ion channel		1.982	0.0224	ACVR1C,CALB1,NEFL,SV2C
477	DG - ne	Ctcflos				other			0.0341	CEBPB,CIDEA
478	DG - ne	LRAT				enzyme			0.0467	CCNE1,TGFA
479	DG - ne	ZFP36L2				transcription reg			0.014	DDIT4,NEFM
480	DG - ne	ADAM10	0.698	-0.115	0.483	peptidase			0.00844	CDHR1,CXCL12,DTNA,FABP7,GSS,NOS1,PVALB,RGS4,Tubb2b
481	DG - ne	DMD	0.0882	0.461	0.0183	other		1.067	0.00233	ARHGAP20,ATP1B2,CAPN3,CDH13,DCX,GALNT17,NOS1,RAP1GAP2,TTLL7,WWC1
482	DG - ne	REST				transcription reg		1	0.0187	BDNF,CXCL12,NEFM,PENK,SOX11
483	DG - ne	CNBP	0.681	0.045	0.46	transcription reg			0.0231	AK1,PGM1
484	DG - ne	IL4				cytokine		-1.334	0.0103	ADCY1,BHLHE40,EPOP,FKBP5,FLNB,GPLD1,HLA-DMA,INPP1,PFKP,THOP1
485	DG - ne	SIM1				transcription reg			0.0467	ROBO3,SST
486	DG - ne	TJP1	0.262	0.343	0.091	other		1	0.00565	DDIT4,PRICKLE1,RHOB,STMN1
487	DG - ne	ESR2				ligand-depender		-1.342	0.0428	CABP1,CADM3,CDH13,EPHA5,FKBP5,FOXO3,KCNJ3,MYCL,SRXN1,TGFBR1
488	DG - ne	ACSS2	0.537	-0.264	0.3	enzyme			0.029	BDNF,CTDSP2,DGAT2
489	DG - ne	mir-223				microRNA		1.48	0.017	ALCAM,CCNE1,FNIP2,FOXO3,ID2,INPP5J,LPL,TOB1
490	DG - ne	mir-19 (includes c				microRNA			0.0341	ADCY1,CIDEA

	A	B	C	D	E	F	G	H	I	J
491	DG - ne	mir-802				microRNA		0	0.0481	ARL15,ARPC4,CDH11,CXCL12,GNAO1,MAPK8,RHOB,SCG2
492	DG - ne	MXI1	0.395	-0.306	0.177	transcription reg			0.027	IRS2
493	DG - ne	HTR1A				G-protein couple			0.027	NOS1
494	DG - ne	ADCYAP1				other		-1.134	0.00765	ARHGDIG,CRTAC1,EDIL3,NPY1R,NPY5R,ST18,TENM2
495	DG - ne	RTN4	7.25E-08	0.242	1.68E-09	other			0.00853	GFAP,GNAO1,NEFL,RHOB,TUBB4A
496	DG - ne	POU4F1				transcription reg		-1.673	0.00683	AJAP1,DCC,LHX2,NPY,NTRK3,NWD2,PLCH2,PVALB
497	DG - ne	POR	0.578	0.165	0.34	enzyme		-0.378	0.0179	ACADVL,APCDD1,CSAD,CYP7B1,HLA-DMA,ITGA7,JDP2,LHX2,LPL,Tubb2b
498	DG - ne	CUL3	0.384	0.197	0.169	enzyme		0.555	0.00876	CCNE1,FGFR1,IRS2,PLS3,RHOB,SPON1
499	DG - ne	STAT6				transcription reg		-1.781	1	BCL6,CAMK1D,EFHD2,HLA-DMA,INPP1,IRS2,KSR1,PRKCA,TNIP2,UBL3
500	DG - ne	FOXO4				transcription reg		-1.342	0.0475	FABP5,FLNB,IL1R1,SESN1,SPTLC2
501	DG - ne	SPRY2				other		-1.698	0.0262	CIDEA,FABP7,ID2,MYCN,SATB1,SPON1
502	DG - ne	PRDM8	0.0557	0.229	0.00989	transcription reg			0.0402	CDH11,GRIN3A
503	DG - ne	ADORA2A				G-protein couple		0.388	0.0238	DGAT2,GPLD1,HPCA,KIF5A,PDYN,PENK,TUBB4A
504	DG - ne	SRPK3				kinase			0.027	ATXN1
505	DG - ne	PKD1	0.002	-0.585	0.000157	ion channel		-0.282	0.0169	ATP1B2,EZH1,FGFR1,JAKMIP2,NAPEPLD,PCDH9,PKNOX2,PRKCB,TENM2,TGFBR1
506	DG - ne	PHLDA1				other			0.0261	DGAT2,GDF10,SREBF2
507	DG - ne	LIPE				enzyme			0.0428	BCL6,CEBPB,FKBP5,ID2,NET1,NPY,PVALB
508	DG - ne	GNB3				other			0.0341	GNAO1,PRKCA
509	DG - ne	MAPK9	0.338	0.232	0.136	kinase		-1.98	0.177	BMP1,BRF1,CEBPB,FOSL2,JUND
510	DG - ne	CLOCK	0.751	0.13	0.552	transcription reg			0.00456	AK4,ARHGEF28,BHLHE40,CCNT1,FKBP5,GNAO1,MAPK8,PPIF,RAP2B,SRR
511	DG - ne	PPARGC1A				transcription reg		0.667	0.0144	ACADVL,CEBPB,CEBPD,GNAO1,INPP5J,IRS2,LPL,PTN,SLC24A2,WDR77
512	DG - ne	GHR				transmembrane		1.969	0.13	BDNF,NPY,SRXN1,SST
513	DG - ne	ST13	0.701	-0.1	0.487	other			0.014	CEBPB,CEBPD
514	DG - ne	LDB1	0.079	0.587	0.0159	transcription reg		0.087	0.029	CDH13,CPEB1,GPRC5B,IRS2,PDZD2,PTPN3,PTPRJ,SOBP,SPRED1,SST
515	DG - ne	ERN1				kinase		1.973	0.232	CEBPB,CEBPD,DGAT2,WFS1
516	DG - ne	MECP2	0.947	-0.024	0.895	transcription reg		-0.082	0.025	B3GALT5,BDNF,FKBP5,GABRA5,KCNF1,NEFM,PACSIN2,PLS3
517	DG - ne	PSEN1	0.33	0.343	0.131	peptidase		-1.755	0.0217	ARHGDIG,BDNF,DCX,FOXO3,GFAP,KCNAB2,NNAT,NOS1,SRC
518	DG - ne	NFE2L2				transcription reg		0.687	0.00645	COPS5,FABP5,FKBP5,FRMD4B,Gsta4,HLA-DMA,NRSN1,PFKP,PRKCB,SATB1
519	DG - ne	HNF4A				transcription reg		0.45	0.0176	ALCAM,B2M,CHIC1,CYP7B1,FHL1,FLNB,GLCE,GPLD1,PARVA,PKP2
520	DG - ne	FOXO1	0.971	-0.011	0.94	transcription reg		-0.711	0.0195	CYP7B1,FABP5,FLNB,IL1R1,IRS2,MYCN,NPY,SESN1,SPTLC2,SREBF2
521	DG - ne	STK16	0.274	0.344	0.0975	kinase			0.027	ATXN1
522	DG - ne	GATAD2B	0.611	0.211	0.375	transcription reg		1.633	0.000106	Gm10076,JUND,LSAMP,PTN,PTPRD,UBA52
523	DG - ne	NTRK2	0.124	0.439	0.0298	kinase		0.447	0.000747	CCNE1,CEBPB,CYTH4,GAD1,PVALB,TGFBR1,TRANK1
524	DG - ne	RGS4	0.0127	-0.984	0.00148	enzyme		-0.302	0.000042	CALN1,DNER,GFOD1,INPP5J,NDST4,NRIP3,NTNG1,PCDH9,PDE2A,PRSS12
525	DG - ne	TCF20	0.761	0.136	0.57	transcription reg		0.447	0.0276	B3GALT5,FXYD6,GABRA5,PACSIN2,PLS3
526	DG - ne	NRAS	0.748	0.156	0.548	enzyme		-0.378	0.0412	AK1,Arxes1/Arxes2,B2M,CRIP2,FHL1,PENK,TULP4
527	DG - ne	PGR				ligand-dependen		-1.508	0.0344	BMP1,CADM1,CADM3,CDH13,FHL1,NPNT,SLIT2,STMN1,STMN2,TENM2
528	DG - ne	TP63				transcription reg		0.653	0.0192	BCL6,DDIT4,MAMLD1,PCBP4,PKP2,RASGRF1,SLIT2,SPON1,Tubb2b,VMP1
529	DG - ne	CSF3R				transmembrane			0.027	CXCL12
530	DG - ne	EP300	0.624	-0.202	0.389	transcription reg			0.0282	BCL6,FMNL2,FOSL2,FRMD4B,HDAC11,ID2,NEBL,NTRK3,SPSB1,WFS1
531	DG - ne	NCOA2	0.383	-0.303	0.168	transcription reg		-1.982	0.425	CEBPB,GLO1,IP6K2,LPL,SRXN1
532	DG - ne	TAC1				other			0.0102	NOS1,PDYN
533	DG - ne	DYNLL1	0.648	-0.022	0.421	other			0.027	CCNE1
534	DG - ne	GHSR				G-protein couple			0.0402	IRS2,NPY
535	DG - ne	NKX3-1				transcription reg			0.00931	CA12,EZH1,FKBP5,FOXO3,PRKCB
536	DG - ne	USP19	0.626	-0.136	0.392	peptidase			0.0159	DDIT4,FKBP5,FOXO3
537	DG - ne	MAP3K8				kinase		0.277	0.0371	CEBPD,CPEB1,FKBP5,FLNB,GFAP,IL1R1,RASGRF1,SESN1,SYNGR1,Tubb2b
538	DG - ne	RPS6KA2	0.142	0.477	0.0363	kinase			0.027	ATXN1
539	DG - ne	APC	0.0113	0.55	0.00129	enzyme			0.00765	CHGB,CXCL12,DDIT4,FOXO3,ID2,LPL,PRKCA,PRKCB,SST
540	DG - ne	RELN				peptidase			0.00146	BDNF,FABP7,GAD1
541	DG - ne	DIO3				enzyme		1	0.0473	CIDEA,CTTNBP2,FLYWCH2,FXYD6,GDA,HRK,ITGA7,KCNJ3,SEMA7A
542	DG - ne	TBC1D1				other			0.0341	CIDEA,FABP5
543	DG - ne	HADH				enzyme		-0.378	0.000669	ATP2B4,CABP1,CACNG3,LARS2,PRKCA,RASD2,SCG2
544	DG - ne	IL4R				transmembrane		0	0.02	CACNB2,CEBPB,CTTNBP2,PTN,SEZ6,SEZ6L2
545	DG - ne	MXD4	0.758	0.112	0.562	transcription reg			0.027	ID2
546	DG - ne	ANK2	0.192	-0.179	0.0563	other			0.00572	KALRN,NEFL,NEFM
547	DG - ne	AK1	0.0168	0.593	0.00207	kinase			0.0467	AK1,CKMT1B
548	DG - ne	CPE	0.0096	0.141	0.00105	peptidase			0.00475	CHGB,PDYN,PENK,SCG2,SREBF2
549	DG - ne	HNRNPH1	0.983	0.003	0.962	other			0.000763	CARMIL1,FHL1,GDA,LASP1,NDUFAF2,TMEM70
550	DG - ne	PAGR1				other			0.000729	CEBPB,CEBPD
551	DG - ne	HTT	0.539	-0.201	0.302	transcription reg			0.0000198	CA12,FXYD6,GLO1,GNAO1,GSS,KCNC4,KCNK2,PENK,SEMA4D,SEZ6
552	DG - ne	MYCN	1.33E-09	1.374	1.97E-11	transcription reg		-1.342	0.0149	BCL11A,DNMT3A,EPHA5,EZH1,HDAC11,PTPRD
553	DG - ne	YY1	0.841	-0.057	0.696	transcription reg		-1	0.00682	ALCAM,CEBPB,DDIT4,DNMT3A,DROSHA,ID2,NNAT,NTF3,SRC,TGFBR1
554	DG - ne	ARID1A	0.754	0.097	0.556	transcription reg		-1.897	0.0782	ATP1B2,BAMBI,CACNB2,DSP,Gsta4,ITGA7,MYCN,NPNT,PARP1,ZBTB7A
555	DG - ne	RHO				G-protein couple		1	0.0273	B2M,CACNB2,CEBPB,CEBPD,FABP7,PSME1,STMN1
556	DG - ne	ITPR1	0.927	0.023	0.856	ion channel			0.0467	BDNF,NOS1
557	DG - ne	VEGFD				growth factor			0.00422	BDNF,DNMT3A
558	DG - ne	JAM2				other			0.027	CXCL12
559	DG - ne	BAG3				other			0.0248	ACADVL,CEBPB,EFHD2,FHL1,FNIP2,FOSL2,FOXO3,Gstm7,NEBL,STUB1
560	DG - ne	CNR1	0.449	0.396	0.219	G-protein couple		0.469	0.00133	APCDD1,CYP7B1,DRD5,DYRK2,ERRFI1,GAD1,GLO1,PDYN,PENK,SLIT2
561	DG - ne	KAT2A	0.924	0.037	0.845	enzyme		0.378	0.0217	ACTN4,BCL11A,EPHA5,FABP5,HDAC11,PTPRD,THOP1
562	DG - ne	FOS				transcription reg			0.043	ATF7IP,BDNF,CADM1,CCNE1,CMTM4,IRS2,KRT2,LPL,NET1,SEMA4D
563	DG - ne	PAX3				transcription reg			0.0402	FABP7,ID2
564	DG - ne	PAX7				transcription reg		0.152	0.000646	CAMK1D,CDH11,CRLF1,ID2,KCNK2,NPNT,RNH1,TOB1
565	DG - ne	ATOH1				transcription reg			0.029	NOS1,RASD2,SST
566	DG - ne	CYP27A1				enzyme			0.0467	CYP7B1,SREBF2
567	DG - ne	NEU1	0.877	-0.07	0.76	enzyme			0.014	DGAT2,SREBF2
568	DG - ne	HRAS	0.963	0.01	0.924	enzyme			0.0347	FHL1,GRWD1,NLN,NNAT,ST18
569	DG - ne	NDUFS4	0.523	-0.101	0.287	enzyme			0.0284	CIDEA,FGFR1
570	DG - ne	MAFB				transcription reg		0.447	0.0174	EFNA3,IGHM,LARS2,LGI3,ZNF189
571	DG - ne	FOXA1				transcription reg		0.555	0.0229	DCX,GRIN3A,NPTX2,NPY,SST
572	DG - ne	DDB1	0.249	0.197	0.0838	other			0.0159	CEBPB,CEBPD,ID2
573	DG - ne	TJP2				kinase		1	0.00565	DDIT4,PRICKLE1,RHOB,STMN1
574	DG - ne	SSTR2				G-protein couple			0.0402	LPL,SST
575	DG - ne	KLF15				transcription reg		-1	0.000532	ACADVL,CEBPB,CEBPD,DDIT4,DGAT2,FABP5,FKBP5,NET1
576	DG - ne	RET				kinase			0.0352	CALB1,NOS1,NTF3,SST
577	DG - ne	CHD4	0.231	0.195	0.0747	enzyme		0	0.00358	BCL11A,DAPK1,ID2,NT5DC2,OPRL1,SCN3B
578	DG - ne	RELB				transcription reg		-1.342	0.0351	AHCYL2,CARMIL1,IGHM,JPH1,NEBL,TGFA
579	DG - ne	EFNA2				kinase			0.000729	BDNF,NOS1
580	DG - ne	CREBBP	0.134	-0.32	0.0331	transcription reg			0.0165	BCL6,CXCL12,DCX,FMNL2,FOSL2,JUND,NEBL,PITPNM1,SPSB1,TULP4
581	DG - ne	MXD3				transcription reg			0.000729	ID2,MYCN
582	DG - ne	CTNNB1	0.123	0.102	0.0295	transcription reg		0.169	0.0011	APC,BHLHE40,CXCL12,CYP7B1,GNAO1,LHX2,MYCN,PDYN,SESN1,Tubb2b
583	DG - ne	RCOR2	0.928	-0.045	0.859	transcription reg			0.0284	BDNF,NEFM
584	DG - ne	FOXP1				transcription reg		0	0.00427	CDH9,DNER,MYCN,RYR1,SPHKAP
585	DG - ne	NCOA6	0.484	0.231	0.25	transcription reg			0.0341	LPL,TGFA
586	DG - ne	MT3	2.23E-08	-0.107	4.26E-10	other			0.027	GFAP
587	DG - ne	Cxcl3				cytokine			0.0341	CEBPB,CEBPD
588	DG - ne	PLN				transporter			0.0341	PRKCA,PRKCB

	A	B	C	D	E	F	G	H	I	J
589	DG - ne	TCF7				transcription reg		1.107	0.0135	Cd200,CEBPD,FGFR1,GNAO1,ID2,PARP1,PENK,RAB4A,SREBF2,ZNF467
590	DG - ne	TWSG1	0.931	-0.042	0.864	other			0.00422	BCL6,ID2
591	DG - ne	TGM5				enzyme			0.027	ATXN1
592	DG - ne	FMR1	0.583	-0.17	0.344	translation regul		0.749	0.044	CELF4,GABRA5,GRM2,PFKP,RASGRF1,RPRM,RTN4RL1,SLC24A2,SPON1,SYNGR1
593	DG - ne	TP53				transcription reg		-0.676	0.00162	ACTN4,COPS5,FABP5,FGFR1,LHX2,PFKP,PRKCB,TGFBR1,TOB1,VMP1
594	DG - ne	PDX1				transcription reg			0.0421	CHGB,FGFR1,IRS2,NNAT,SCG2,WFS1
595	DG - ne	COLQ				other		0.152	0.0387	BDNF,BMP1,DTNA,ITGA7,SNTA1
596	DG - ne	EEF2	0.248	-0.072	0.083	translation regul			0.027	BDNF
597	DG - ne	SLC30A3	0.988	0.002	0.973	transporter			0.0183	BDNF,DCX
598	DG - ne	VGF	0.44	0.454	0.211	growth factor			0.0102	CHGB,NPY
599										
600										
601										
602										

	A	B	C	D	E
1	**Supplementary Table 5- IPA pathways CA1**				
2					
3	¬© 2000-2024 QIAGEN. All rights reserved.				
4					
5	**Ingenuity Canonical Pathways**	**-log(p-value**	**Ratio**	**z-score**	**Molecules**
6	Cardiac conduction	4.68E+00	6.35E-02	1.134	CACNB2,FKBP1B,FXYD2,FXYD6,FXYD7,KCNK1,SCN3B,STIM1
7	Opioid Signaling Pathway	3.61E+00	3.60E-02	-0.707	ADCY1,CACNB2,CACNG2,EP300,GRIN3A,NPBWR1,PENK,PRKCA,RASD1,RGS4
8	Synaptic Long Term Potentiation	2.87E+00	4.55E-02	-1	ADCY1,EP300,GRIN3A,PPP1CC,PRKCA,RASD1
9	Orexin Signaling Pathway	2.85E+00	3.42E-02	-1.414	ADCY1,CACNB2,CACNG2,CNR1,DAGLA,KCNK1,PIP5K1B,PRKCA
10	Dopamine-DARPP32 Feedback in cAMP Signaling	2.82E+00	3.80E-02	-1	ADCY1,CACNB2,CACNG2,CSNK1A1,GRIN3A,PPP1CC,PRKCA
11	Prolactin Signaling	2.77E+00	5.32E-02	NaN	EP300,KCNMB4,NR3C1,PRKCA,RASD1
12	Endocannabinoid Neuronal Synapse Pathway	2.62E+00	4.05E-02	0.816	ADCY1,CACNB2,CACNG2,CNR1,DAGLA,GRIN3A
13	CREB Signaling in Neurons	2.55E+00	2.23E-02	-0.577	ADCY1,ADGRA1,CACNB2,CACNG2,CNR1,EP300,GPR161,HTR1A,MC4R,NPBWR1,NPY2R,PRKCA,RASD1
14	Sialic acid metabolism	2.52E+00	9.38E-02	NaN	ST3GAL5,ST6GAL2,ST8SIA5
15	Circadian Rhythm Signaling	2.49E+00	3.00E-02	NaN	ADCY1,CACNB2,CACNG2,DBP,GRIN3A,NR3C1,PRKCA,RASD1
16	3-phosphoinositide Biosynthesis	2.49E+00	3.33E-02	-2	DUSP14,DUSP5,PIP5K1B,PLPP6,PPP1CC,PPP1R13B,PTPRU
17	G alpha (i) signalling events	2.38E+00	3.18E-02	1.134	ADCY1,CCL27,CNR1,NPBWR1,NPY2R,PENK,RGS4
18	Role of NFAT in Cardiac Hypertrophy	2.34E+00	3.12E-02	-1.342	ADCY1,CACNB2,CACNG2,CSNK1A1,EP300,PRKCA,RASD1
19	Signaling by NOTCH1	2.31E+00	5.33E-02	0	APH1A,EP300,FBXW7,Tle1
20	Presynaptic depolarization and calcium channel opening	2.29E+00	1.67E-01	NaN	CACNB2,CACNG2
21	Breast Cancer Regulation by Stathmin1	2.21E+00	2.11E-02	-0.302	ADGRA1,CNR1,GPR161,HTR1A,MC4R,NPBWR1,NPY2R,PPP1CC,PRKCA,RASD1,STMN1,TUBB3
22	Superpathway of Inositol Phosphate Compounds	2.21E+00	2.95E-02	-2	DUSP14,DUSP5,PIP5K1B,PLPP6,PPP1CC,PPP1R13B,PTPRU
23	G Beta Gamma Signaling	2.18E+00	3.88E-02	0	ADCY1,CACNB2,CACNG2,PRKCA,RASD1
24	D-myo-inositol (1,4,5,6)-Tetrakisphosphate Biosynthesis	2.16E+00	3.26E-02	NaN	DUSP14,DUSP5,PLPP6,PPP1CC,PPP1R13B,PTPRU
25	D-myo-inositol (3,4,5,6)-tetrakisphosphate Biosynthesis	2.16E+00	3.26E-02	NaN	DUSP14,DUSP5,PLPP6,PPP1CC,PPP1R13B,PTPRU
26	Gustation Pathway	2.16E+00	3.26E-02	-0.816	ADCY1,CACNB2,CACNG2,HTR1A,SCN3B,STIM1
27	Netrin-1 signaling	2.12E+00	6.82E-02	NaN	NEO1,RGMA,SLIT1
28	HSP90 chaperone cycle for steroid hormone receptors in the presence	2.04E+00	6.38E-02	NaN	NR3C1,NR3C2,TUBB3
29	3-phosphoinositide Degradation	2.04E+00	3.08E-02	NaN	DUSP14,DUSP5,PLPP6,PPP1CC,PPP1R13B,PTPRU
30	G alpha (z) signalling events	2.02E+00	6.25E-02	NaN	ADCY1,PRKCA,RGS4
31	nNOS Signaling in Neurons	2.02E+00	6.25E-02	NaN	GRIN3A,PRKCA,RASD1
32	GŒ±i Signaling	2.01E+00	3.52E-02	0	ADCY1,CNR1,HTR1A,RASD1,RGS4
33	D-myo-inositol-5-phosphate Metabolism	1.99E+00	3.00E-02	NaN	DUSP14,DUSP5,PLPP6,PPP1CC,PPP1R13B,PTPRU
34	Signaling by NOTCH3	1.97E+00	6.00E-02	NaN	APH1A,EP300,PLXND1
35	Corticotropin Releasing Hormone Signaling	1.90E+00	3.31E-02	-0.447	ADCY1,CACNB2,CACNG2,CNR1,PRKCA
36	S100 Family Signaling Pathway	1.86E+00	1.82E-02	0.277	ADGRA1,CACNB2,CACNG2,CNR1,GPR161,HTR1A,IGHM,MC4R,NPBWR1,NPY2R,NR3C1,PRKCA,WNT2
37	Circadian Clock	1.82E+00	5.26E-02	NaN	EP300,NR3C1,PPP1CC
38	Chondroitin Sulfate Biosynthesis	1.82E+00	5.26E-02	NaN	HS6ST2,UST,XYLT1
39	Neurovascular Coupling Signaling Pathway	1.81E+00	2.74E-02	0.816	CACNB2,CACNG2,DAGLA,GRIN3A,KCNMB4,STIM1
40	Phagosome Formation	1.80E+00	1.86E-02	-0.577	ADGRA1,ARPC2,CNR1,GPR161,HTR1A,IGHM,MC4R,NPBWR1,NPY2R,PIP5K1B,PRKCA,RASD1
41	Dermatan Sulfate Biosynthesis	1.78E+00	5.08E-02	NaN	HS6ST2,UST,XYLT1
42	Hedgehog 'off' state	1.75E+00	3.67E-02	NaN	ADCY1,CSNK1A1,GPR161,TUBB3
43	Class A/1 (Rhodopsin-like receptors)	1.75E+00	2.40E-02	0.378	CCL27,CNR1,HTR1A,MC4R,NPBWR1,NPY2R,PENK
44	Alanine metabolism	1.74E+00	5.00E-01	NaN	GPT2
45	Alanine Degradation III	1.74E+00	5.00E-01	NaN	GPT2
46	Alanine Biosynthesis II	1.74E+00	5.00E-01	NaN	GPT2
47	Xenobiotic Metabolism PXR Signaling Pathway	1.73E+00	2.99E-02	0	EP300,HS6ST2,PPP1CC,PRKCA,UST
48	Amyotrophic Lateral Sclerosis Signaling	1.71E+00	3.57E-02	NaN	CACNB2,CACNG2,GRIN3A,NEFM
49	Remodeling of Epithelial Adherens Junctions	1.70E+00	4.76E-02	NaN	ARPC2,CBLL1,TUBB3
50	L1CAM interactions	1.70E+00	3.54E-02	1	ALCAM,GAP43,SCN3B,TUBB3
51	Sleep NREM Signaling Pathway	1.70E+00	3.54E-02	0	ADCY1,PARP1,PRKCA,RASD1
52	Amyloid fiber formation	1.68E+00	4.69E-02	NaN	APH1A,CALB1,TTR
53	Netrin Signaling	1.68E+00	2.91E-02	-0.447	ARPC2,CACNB2,CACNG2,NEO1,PRKCA
54	Pyridoxal 5'-phosphate Salvage Pathway	1.67E+00	4.62E-02	NaN	CSNK1A1,DAPK1,PDXK
55	Cholesterol biosynthesis	1.66E+00	8.00E-02	NaN	DHCR24,PLPP6
56	GPCR-Mediated Nutrient Sensing in Enteroendocrine Cells	1.65E+00	3.42E-02	-1	ADCY1,CACNB2,CACNG2,PRKCA
57	WNT ligand biogenesis and trafficking	1.63E+00	7.69E-02	NaN	WLS,WNT2
58	ERBB4 Signaling	1.63E+00	4.48E-02	NaN	APH1A,PRKCA,RASD1
59	TCF dependent signaling in response to WNT	1.60E+00	2.78E-02	-1.342	CSNK1A1,EP300,PIP5K1B,RSPO2,Tle1
60	CDP-diacylglycerol Biosynthesis I	1.60E+00	7.41E-02	NaN	AGPAT5,CDS1
61	Glutaminergic Receptor Signaling Pathway (Enhanced)	1.57E+00	2.21E-02	-0.378	ADCY1,CACNB2,CACNG2,GRIN3A,NR3C1,PRKCA,SCN3B
62	GŒ±s Signaling	1.57E+00	3.23E-02	0	ADCY1,ADD2,CNR1,MC4R
63	Lactose Degradation III	1.57E+00	3.33E-01	NaN	LCT
64	SUMOylation of intracellular receptors	1.54E+00	6.90E-02	NaN	NR3C1,NR3C2
65	Phosphatidylglycerol Biosynthesis II (Non-plastidic)	1.54E+00	6.90E-02	NaN	AGPAT5,CDS1
66	Maturity Onset Diabetes of Young (MODY) Signaling	1.54E+00	4.11E-02	NaN	CACNB2,CACNG2,EP300
67	Acetylcholine Receptor Signaling Pathway	1.53E+00	2.65E-02	-1.342	ADCY1,APH1A,CACNB2,CACNG2,PRKCA
68	GNRH Signaling	1.53E+00	2.65E-02	NaN	ADCY1,CACNB2,CACNG2,PRKCA,RASD1
69	VDR/RXR Activation	1.52E+00	4.05E-02	NaN	CALB1,EP300,PRKCA
70	Signaling by ALK	1.52E+00	6.67E-02	NaN	EP300,PTN
71	Estrogen Receptor Signaling	1.50E+00	1.99E-02	-1.633	ADCY1,CACNB2,CACNG2,EP300,MED14,NR3C1,PRKCA,RASD1
72	Glutamate binding, activation of AMPA receptors and synaptic plasticit	1.49E+00	6.45E-02	NaN	CACNG2,PRKCA
73	DHCR24 Signaling Pathway	1.47E+00	3.01E-02	-2	DHCR24,PRKCA,RASD1,TTR
74	GABAergic Receptor Signaling Pathway (Enhanced)	1.46E+00	2.99E-02	0	ADCY1,CACNB2,CACNG2,GRIN3A
75	Lung Ionic Balance Signaling Pathway	1.46E+00	1.85E-02	0.333	ADGRA1,CNR1,GPR161,HTR1A,KCNMB4,MC4R,NPBWR1,NPY2R,PRKCA
76	Signaling by NOTCH4	1.42E+00	2.90E-02	1	APH1A,EP300,FBXW7,STK32C
77	Signaling by NOTCH2	1.42E+00	5.88E-02	NaN	APH1A,EP300
78	Cellular Effects of Sildenafil (Viagra)	1.41E+00	1.68E-02	-0.632	ADCY1,ADGRA1,CACNB2,CACNG2,CNR1,GPR161,HTR1A,MC4R,NPBWR1,NPY2R,PPP1CC
79	Assembly and cell surface presentation of NMDA receptors	1.37E+00	5.56E-02	NaN	GRIN3A,TUBB3
80	Heparan Sulfate Biosynthesis	1.37E+00	3.53E-02	NaN	HS6ST2,UST,XYLT1
81	NFE2L2 regulating inflammation associated genes	1.35E+00	2.00E-01	NaN	EP300
82	NFE2L2 regulating ER-stress associated genes	1.35E+00	2.00E-01	NaN	EP300
83	G-Protein Coupled Receptor Signaling	1.34E+00	1.63E-02	-0.905	ADCY1,ADGRA1,CNR1,GPR161,HTR1A,MC4R,NPBWR1,NPY2R,PRKCA,RASD1,RGS4
84	Notch Signaling	1.33E+00	5.26E-02	NaN	APH1A,NUMBL
85	Senescence Pathway	1.31E+00	2.08E-02	-2	CACNB2,CACNG2,DHCR24,EP300,PARP1,RASD1
86	DAG and IP3 signaling	1.29E+00	5.00E-02	NaN	ADCY1,PRKCA
87	RET signaling	1.29E+00	5.00E-02	NaN	GFRA2,PRKCA
88	Salvage Pathways of Pyrimidine Ribonucleotides	1.28E+00	3.26E-02	NaN	AK5,CSNK1A1,DAPK1
89	Autism Signaling Pathway	1.28E+00	2.05E-02	-0.816	CACNB2,CACNG2,EP300,GRIN3A,RASD1,WNT2
90	UDP-N-acetyl-D-glucosamine Biosynthesis II	1.27E+00	1.67E-01	NaN	UAP1
91	Signaling by SCF-KIT	1.25E+00	4.76E-02	NaN	PRKCA,PTPRU
92	Serotonin Receptor Signaling	1.24E+00	1.76E-02	-0.707	ADCY1,CACNB2,CACNG2,GAP43,HTR1A,PRKCA,RASD1,STIM1
93	Melanocyte Development and Pigmentation Signaling	1.23E+00	3.09E-02	NaN	ADCY1,EP300,RASD1
94	UVA-Induced MAPK Signaling	1.23E+00	3.09E-02	NaN	PARP1,PRKCA,RASD1
95	TGF-Œ≤ Signaling	1.23E+00	3.09E-02	NaN	EP300,PMEPA1,RASD1
96	Glycoaminoglycan-protein Linkage Region Biosynthesis	1.21E+00	1.43E-01	NaN	XYLT1

	A	B	C	D	E
97	RHO GTPases Activate WASPs and WAVEs	1.20E+00	4.44E-02	NaN	ARPC2,WIPF3
98	cAMP-mediated signaling	1.20E+00	2.14E-02	-1.342	ADCY1,CNR1,HTR1A,MC4R,RGS4
99	Apoptosis Signaling	1.19E+00	2.97E-02	NaN	PARP1,PRKCA,RASD1
100	Pancreatic Secretion Signaling Pathway	1.18E+00	2.12E-02	0	ADCY1,CA12,PPP1CC,PRKCA,STIM1
101	Dermatan Sulfate Biosynthesis (Late Stages)	1.18E+00	4.35E-02	NaN	HS6ST2,UST
102	Androgen Signaling	1.17E+00	2.40E-02	-1	CACNB2,CACNG2,EP300,PRKCA
103	Aldosterone Signaling in Epithelial Cells	1.17E+00	2.38E-02	NaN	KCNMB4,NR3C2,PIP5K1B,PRKCA
104	Xenobiotic Metabolism CAR Signaling Pathway	1.17E+00	2.38E-02	0	EP300,HS6ST2,PRKCA,UST
105	Potassium Channels	1.17E+00	2.91E-02	NaN	KCNF1,KCNK1,KCNMB4
106	WNT/Œ≤-catenin Signaling	1.15E+00	2.35E-02	NaN	CSNK1A1,EP300,Tle1,WNT2
107	Creatine metabolism	1.15E+00	1.25E-01	NaN	SLC6A7
108	NFE2L2 regulates pentose phosphate pathway genes	1.15E+00	1.25E-01	NaN	EP300
109	Regulation of NFE2L2 gene expression	1.15E+00	1.25E-01	NaN	EP300
110	NFE2L2 regulating MDR associated enzymes	1.15E+00	1.25E-01	NaN	EP300
111	Gap Junction Signaling	1.15E+00	1.89E-02	-0.447	ADCY1,CSNK1A1,DAGLA,PRKCA,RASD1,TUBB3
112	Molecular Mechanisms of Cancer	1.13E+00	1.46E-02	-0.577	ADCY1,ADGRA1,APH1A,CNR1,EP300,GPR161,HTR1A,MC4R,NPBWR1,NPY2R,PRKCA,RASD1
113	TP53 Regulates Transcription of Cell Cycle Genes	1.13E+00	4.08E-02	NaN	EP300,RGCC
114	nNOS Signaling in Skeletal Muscle Cells	1.13E+00	4.08E-02	NaN	CACNB2,CACNG2
115	Chondroitin Sulfate Biosynthesis (Late Stages)	1.13E+00	4.08E-02	NaN	HS6ST2,UST
116	Integration of energy metabolism	1.13E+00	2.80E-02	NaN	ADCY1,CACNB2,PRKCA
117	Regulation of Actin-based Motility by Rho	1.13E+00	2.80E-02	NaN	ARPC2,PFN1,PIP5K1B
118	Ion channel transport	1.12E+00	2.30E-02	2	FKBP1B,FXYD2,FXYD6,FXYD7
119	Œ±-Adrenergic Signaling	1.12E+00	2.78E-02	NaN	ADCY1,PRKCA,RASD1
120	Signaling by TGFBR3	1.12E+00	4.00E-02	NaN	APH1A,EP300
121	Amyloid Processing	1.12E+00	4.00E-02	NaN	APH1A,CSNK1A1
122	Hepatic Fibrosis Signaling Pathway	1.11E+00	1.73E-02	-1	CACNB2,CACNG2,CNR1,CSNK1A1,PRKCA,RASD1,WNT2
123	Transcriptional activity of SMAD2/SMAD3:SMAD4 heterotrimer	1.10E+00	3.92E-02	NaN	EP300,PARP1
124	UVC-Induced MAPK Signaling	1.10E+00	3.92E-02	NaN	PRKCA,RASD1
125	Cardiac Œ≤-adrenergic Signaling	1.10E+00	2.26E-02	NaN	ADCY1,CACNB2,CACNG2,PPP1CC
126	Caspase activation via Dependence Receptors in the absence of ligand	1.10E+00	1.11E-01	NaN	DAPK1
127	Zygotic genome activation (ZGA)	1.10E+00	1.11E-01	NaN	EP300
128	Cardiac Hypertrophy Signaling	1.09E+00	1.98E-02	NaN	ADCY1,CACNB2,CACNG2,EP300,RASD1
129	Axonal Guidance Signaling	1.08E+00	1.62E-02	NaN	ARPC2,PFN1,PLXND1,PRKCA,RASD1,SLIT1,TUBB3,WNT2
130	CDK5 Signaling	1.07E+00	2.65E-02	NaN	ADCY1,PPP1CC,RASD1
131	Signaling by TGF-beta Receptor Complex	1.06E+00	3.70E-02	NaN	PMEPA1,PPP1CC
132	Oxytocin in Brain Signaling Pathway	1.05E+00	2.17E-02	-1	CACNB2,CACNG2,PRKCA,RASD1
133	Triacylglycerol Biosynthesis	1.05E+00	3.64E-02	NaN	AGPAT5,PLPP6
134	Nitric Oxide Signaling in the Cardiovascular System	1.04E+00	2.56E-02	NaN	CACNB2,CACNG2,PRKCA
135	Xenobiotic Metabolism Signaling	1.02E+00	1.89E-02	NaN	EP300,HS6ST2,PRKCA,RASD1,UST
136	PPARŒ±/RXRŒ± Activation	1.02E+00	2.12E-02	NaN	ADCY1,EP300,PRKCA,RASD1
137	NFE2L2 regulating tumorigenic genes	1.02E+00	9.09E-02	NaN	EP300
138	Renin-Angiotensin Signaling	1.01E+00	2.50E-02	NaN	ADCY1,PRKCA,RASD1
139	RHOA Signaling	1.01E+00	2.50E-02	NaN	ARPC2,PFN1,PIP5K1B
140	Visual phototransduction	1.01E+00	3.45E-02	NaN	PRKCA,TTR
141	Synaptic Long Term Depression	1.00E+00	2.08E-02	NaN	CACNB2,CACNG2,PRKCA,RASD1
142	Adrenergic Receptor Signaling Pathway (Enhanced)	1.00E+00	2.08E-02	-1	ADCY1,CACNB2,CACNG2,PRKCA
143	Glycerophospholipid biosynthesis	9.96E-01	2.46E-02	NaN	AGPAT5,CDS1,CPNE7
144	Signaling by ERBB4	9.95E-01	3.39E-02	NaN	APH1A,STMN1
145	Pulmonary Healing Signaling Pathway	9.91E-01	2.06E-02	-2	EP300,PRKCA,RASD1,WNT2
146	PCP (Planar Cell Polarity) Pathway	9.83E-01	3.33E-02	NaN	PFN1,WNT2
147	Reversible hydration of carbon dioxide	9.82E-01	8.33E-02	NaN	CA12
148	HDR through MMEJ (alt-NHEJ)	9.82E-01	8.33E-02	NaN	PARP1
149	UDP-N-acetyl-D-galactosamine Biosynthesis II	9.82E-01	8.33E-02	NaN	UAP1
150	Glycosaminoglycan metabolism	9.80E-01	2.42E-02	NaN	HS6ST2,UST,XYLT1
151	Cachexia Signaling Pathway	9.79E-01	1.70E-02	-1.633	ADCY1,EP300,Hspa1b,MC4R,NR3C1,PRKCA
152	14-3-3-mediated Signaling	9.73E-01	2.40E-02	NaN	PRKCA,RASD1,TUBB3
153	Insulin Secretion Signaling Pathway	9.73E-01	1.82E-02	NaN	ADCY1,CACNB2,CACNG2,PCSK2,PRKCA
154	Endocannabinoid Developing Neuron Pathway	9.65E-01	2.38E-02	NaN	ADCY1,CNR1,RASD1
155	Cholesterol Biosynthesis I	9.49E-01	7.69E-02	NaN	DHCR24
156	Cholesterol Biosynthesis II (via 24,25-dihydrolanosterol)	9.49E-01	7.69E-02	NaN	DHCR24
157	Cholesterol Biosynthesis III (via Desmosterol)	9.49E-01	7.69E-02	NaN	DHCR24
158	NCAM signaling for neurite out-growth	9.48E-01	3.17E-02	NaN	CACNB2,GFRA2
159	Thrombopoietin Signaling	9.48E-01	3.17E-02	NaN	PRKCA,RASD1
160	HIF1Œ± Signaling	9.44E-01	1.98E-02	-1	EP300,Hspa1b,PRKCA,RASD1
161	Xenobiotic Metabolism General Signaling Pathway	9.35E-01	2.31E-02	NaN	EP300,PRKCA,RASD1
162	RHO GTPases Activate Formins	9.28E-01	2.29E-02	NaN	PFN1,PPP1CC,TUBB3
163	fMLP Signaling in Neutrophils	9.28E-01	2.29E-02	NaN	ARPC2,PRKCA,RASD1
164	P2Y Purinergic Receptor Signaling Pathway	9.28E-01	2.29E-02	NaN	ADCY1,PRKCA,RASD1
165	GABA Receptor Signaling	9.28E-01	2.29E-02	NaN	ADCY1,CACNB2,CACNG2
166	Role of WNT/GSK-3Œ≤ Signaling in the Pathogenesis of Influenza	9.25E-01	3.08E-02	NaN	CSNK1A1,WNT2
167	LGI-ADAM interactions	9.19E-01	7.14E-02	NaN	CACNG2
168	Regulation of TP53 Activity through Association with Co-factors	9.19E-01	7.14E-02	NaN	PPP1R13B
169	Assembly of RNA Polymerase III Complex	9.19E-01	7.14E-02	NaN	BRF2
170	DNA Double-Strand Break Repair by Non-Homologous End Joining	9.19E-01	7.14E-02	NaN	PARP1
171	DDX58/IFIH1-mediated induction of interferon-alpha/beta	9.14E-01	3.03E-02	NaN	CYLD,EP300
172	Calcium Signaling	8.99E-01	1.90E-02	0	CACNB2,CACNG2,EP300,GRIN3A
173	RAC Signaling	8.85E-01	2.19E-02	NaN	ARPC2,PIP5K1B,RASD1
174	White Adipose Tissue Browning Pathway	8.85E-01	2.19E-02	NaN	ADCY1,CACNB2,CACNG2
175	BBSome Signaling Pathway	8.83E-01	1.52E-02	0.378	ADGRA1,CNR1,GPR161,HTR1A,MC4R,NPBWR1,NPY2R
176	Sensory processing of sound by inner hair cells of the cochlea	8.83E-01	2.90E-02	NaN	CACNB2,RIPOR2
177	ERK/MAPK Signaling	8.83E-01	1.88E-02	NaN	KSR1,PPP1CC,PRKCA,RASD1
178	RHOGDI Signaling	8.72E-01	1.86E-02	1	ARPC2,EP300,PIP5K1B,PRKCA
179	Reelin Signaling in Neurons	8.65E-01	2.14E-02	NaN	ARPC2,CNR1,GRIN3A
180	FOXO-mediated transcription of cell death genes	8.65E-01	6.25E-02	NaN	EP300
181	Granzyme B Signaling	8.65E-01	6.25E-02	NaN	PARP1
182	Hedgehog 'on' state	8.59E-01	2.13E-02	NaN	CSNK1A1,GPR161,STK32C
183	Apelin Endothelial Signaling Pathway	8.52E-01	2.11E-02	NaN	ADCY1,PRKCA,RASD1
184	RORA activates gene expression	8.41E-01	5.88E-02	NaN	EP300
185	Gastrin-CREB signalling pathway via PKC and MAPK	8.41E-01	5.88E-02	NaN	PRKCA
186	Digestion	8.41E-01	5.88E-02	NaN	LCT
187	Caveolar-mediated Endocytosis Signaling	8.34E-01	2.70E-02	NaN	DYRK3,PRKCA
188	GPCR-Mediated Integration of Enteroendocrine Signaling Exemplified	8.34E-01	2.70E-02	NaN	ADCY1,NPY2R
189	Chemokine Signaling	8.25E-01	2.67E-02	NaN	PRKCA,RASD1
190	Dilated Cardiomyopathy Signaling Pathway	8.20E-01	2.04E-02	NaN	ADCY1,CACNB2,CACNG2
191	NFE2L2 regulating anti-oxidant/detoxification enzymes	8.18E-01	5.56E-02	NaN	EP300
192	Signaling by ROBO receptors	8.17E-01	1.77E-02	-2	ARHGAP39,PFN1,PRKCA,SLIT1

	A	B	C	D	E
193	Cellular response to hypoxia	8.16E-01	2.63E-02	NaN	EP300,HIGD1A
194	Activation of NMDA receptors and postsynaptic events	8.16E-01	2.63E-02	NaN	ADCY1,TUBB3
195	GDNF Family Ligand-Receptor Interactions	8.16E-01	2.63E-02	NaN	GFRA2,RASD1
196	Hypoxia Signaling in the Cardiovascular System	8.16E-01	2.63E-02	NaN	EP300,UBE2E2
197	Macropinocytosis Signaling	8.16E-01	2.63E-02	NaN	PRKCA,RASD1
198	Protein Kinase A Signaling	8.10E-01	1.52E-02	1.342	ADCY1,ADD2,DUSP5,PPP1CC,PRKCA,PTPRU
199	eNOS Signaling	8.08E-01	2.01E-02	NaN	ADCY1,Hspa1b,PRKCA
200	NF-ŒºB Activation by Viruses	8.07E-01	2.60E-02	NaN	PRKCA,RASD1
201	Dopamine Receptor Signaling	8.07E-01	2.60E-02	NaN	ADCY1,PPP1CC
202	Synaptogenesis Signaling Pathway	8.03E-01	1.60E-02	NaN	ADCY1,ARPC2,CACNB2,GRIN3A,RASD1
203	WNT/SHH Axonal Guidance Signaling Pathway	8.02E-01	2.00E-02	NaN	ADCY1,PRKCA,WNT2
204	Role of Macrophages, Fibroblasts and Endothelial Cells in Rheumatoid	7.99E-01	1.60E-02	-0.447	CSNK1A1,IGHM,PRKCA,RASD1,WNT2
205	NAP1L1 Transcription Regulation Signaling Pathway	7.98E-01	2.56E-02	NaN	PRDM8,TUBB3
206	Renal Cell Carcinoma Signaling	7.98E-01	2.56E-02	NaN	EP300,RASD1
207	Role of MAPK Signaling in the Pathogenesis of Influenza	7.98E-01	2.56E-02	NaN	PRKCA,RASD1
208	Heparan Sulfate Biosynthesis (Late Stages)	7.98E-01	2.56E-02	NaN	HS6ST2,UST
209	Regulation of TP53 Activity through Methylation	7.96E-01	5.26E-02	NaN	EP300
210	FOXO-mediated transcription	7.96E-01	5.26E-02	NaN	EP300
211	Type II Diabetes Mellitus Signaling	7.90E-01	1.97E-02	NaN	CACNB2,CACNG2,PRKCA
212	IL-3 Signaling	7.81E-01	2.50E-02	NaN	PRKCA,RASD1
213	Receptor-type tyrosine-protein phosphatases	7.76E-01	5.00E-02	NaN	SLITRK1
214	Actin Cytoskeleton Signaling	7.66E-01	1.69E-02	NaN	ARPC2,PFN1,PIP5K1B,RASD1
215	Immunogenic Cell Death Signaling Pathway	7.64E-01	2.44E-02	NaN	DAPK1,Hspa1b
216	Transcriptional regulation of white adipocyte differentiation	7.64E-01	2.44E-02	NaN	EP300,MED14
217	VEGF Family Ligand-Receptor Interactions	7.56E-01	2.41E-02	NaN	PRKCA,RASD1
218	Platelet homeostasis	7.40E-01	2.35E-02	NaN	KCNMB4,STIM1
219	LPS-stimulated MAPK Signaling	7.40E-01	2.35E-02	NaN	PRKCA,RASD1
220	Regulation of the Epithelial Mesenchymal Transition in Development P	7.40E-01	2.35E-02	NaN	APH1A,WNT2
221	Formation of paraxial mesoderm	7.38E-01	4.55E-02	NaN	EP300
222	Pyrimidine Deoxyribonucleotides De Novo Biosynthesis I	7.38E-01	4.55E-02	NaN	AK5
223	PDGF Signaling	7.32E-01	2.33E-02	NaN	PRKCA,RASD1
224	CXCR4 Signaling	7.29E-01	1.84E-02	NaN	ADCY1,PRKCA,RASD1
225	Mitochondrial Division Signaling Pathway	7.23E-01	1.83E-02	NaN	CACNB2,CACNG2,RASD1
226	Transcriptional Regulatory Network in Embryonic Stem Cells	7.23E-01	1.83E-02	NaN	CALB1,RASD1,WNT2
227	RAF-independent MAPK1/3 activation	7.21E-01	4.35E-02	NaN	DUSP5
228	Opioid Signalling	7.10E-01	2.25E-02	NaN	ADCY1,PRKCA
229	Fcgamma receptor (FCGR) dependent phagocytosis	7.10E-01	2.25E-02	NaN	ARPC2,WIPF3
230	Hepatitis B Chronic Liver Pathogenesis Signaling Pathway	7.08E-01	1.80E-02	NaN	PRKCA,RASD1,SATB1
231	RHO GTPases activate IQGAPs	7.04E-01	4.17E-02	NaN	TUBB3
232	RHO GTPases Activate NADPH Oxidases	7.04E-01	4.17E-02	NaN	PRKCA
233	Degradation of beta-catenin by the destruction complex	7.02E-01	2.22E-02	NaN	CSNK1A1,Tle1
234	Keratinization	7.02E-01	2.22E-02	NaN	PKP2,PKP4
235	Ceramide Signaling	7.02E-01	2.22E-02	NaN	KSR1,RASD1
236	Actin Nucleation by ARP-WASP Complex	7.02E-01	2.22E-02	NaN	ARPC2,RASD1
237	EPH-Ephrin signaling	6.95E-01	2.20E-02	NaN	APH1A,ARPC2
238	Protein folding	6.95E-01	2.20E-02	NaN	FBXW7,TUBB3
239	FcŒ≥ Receptor-mediated Phagocytosis in Macrophages and Monocyte	6.95E-01	2.20E-02	NaN	ARPC2,PRKCA
240	Insulin processing	6.88E-01	4.00E-02	NaN	PCSK2
241	Miscellaneous transport and binding events	6.88E-01	4.00E-02	NaN	ADD2
242	Cardiac Hypertrophy Signaling (Enhanced)	6.83E-01	1.33E-02	NaN	ADCY1,CACNB2,CACNG2,EP300,PRKCA,RASD1,WNT2
243	COPI-mediated anterograde transport	6.81E-01	2.15E-02	NaN	TMEM115,TUBB3
244	ERBB Signaling	6.81E-01	2.15E-02	NaN	PRKCA,RASD1
245	Non-Small Cell Lung Cancer Signaling	6.74E-01	2.13E-02	NaN	PRKCA,RASD1
246	BMAL1:CLOCK,NPAS2 activates circadian gene expression	6.73E-01	3.85E-02	NaN	DBP
247	D-myo-inositol (1,4,5)-Trisphosphate Biosynthesis	6.73E-01	3.85E-02	NaN	PIP5K1B
248	Glycolysis I	6.73E-01	3.85E-02	NaN	ENO1
249	Gluconeogenesis I	6.73E-01	3.85E-02	NaN	ENO1
250	RAF/MAP kinase cascade	6.70E-01	1.54E-02	-1	DUSP5,GFRA2,KSR1,PPP1CC
251	FAK Signaling	6.68E-01	1.22E-02	0	ADGRA1,ARPC2,CNR1,GPR161,HTR1A,MC4R,NPBWR1,NPY2R,RASD1,SPARCL1
252	p53 Signaling	6.67E-01	2.11E-02	NaN	EP300,PPP1R13B
253	Acute Phase Response Signaling	6.63E-01	1.70E-02	NaN	NR3C1,RASD1,TTR
254	RHO GTPase cycle	6.62E-01	1.36E-02	-0.816	ARHGAP39,DOCK4,DST,PKP4,PLXND1,WIPF3
255	VEGF Signaling	6.61E-01	2.08E-02	NaN	PRKCA,RASD1
256	Effects of PIP2 hydrolysis	6.59E-01	3.70E-02	NaN	DAGLA
257	Syndecan interactions	6.59E-01	3.70E-02	NaN	PRKCA
258	Interconversion of nucleotide di- and triphosphates	6.59E-01	3.70E-02	NaN	AK5
259	Sumoylation Pathway	6.48E-01	2.04E-02	NaN	EP300,NR3C1
260	Superpathway of Cholesterol Biosynthesis	6.45E-01	3.57E-02	NaN	DHCR24
261	Interferon Signaling	6.45E-01	3.57E-02	NaN	MED14
262	GPER1 signaling	6.41E-01	2.02E-02	NaN	ADCY1,STK32C
263	Neuropathic Pain Signaling in Dorsal Horn Neurons	6.35E-01	2.00E-02	NaN	GRIN3A,PRKCA
264	Myogenesis	6.32E-01	3.45E-02	NaN	NEO1
265	FOXO-mediated transcription of oxidative stress, metabolic and neuro	6.32E-01	3.45E-02	NaN	NR3C1
266	Endothelin-1 Signaling	6.31E-01	1.64E-02	NaN	ADCY1,PRKCA,RASD1
267	Eicosanoid Signaling	6.25E-01	1.47E-02	-1	ADCY1,KCNMB4,PRKCA,RASD1
268	Regulation of TP53 Activity through Acetylation	6.19E-01	3.33E-02	NaN	EP300
269	Regulation of CDH11 Expression and Function	6.19E-01	3.33E-02	NaN	PRDM8
270	Sphingolipid metabolism	6.16E-01	1.94E-02	NaN	ST3GAL5,ST8SIA5
271	Post-translational protein phosphorylation	6.16E-01	1.94E-02	NaN	PENK,SPARCL1
272	Huntington's Disease Signaling	6.14E-01	1.45E-02	NaN	EP300,Hspa1b,PENK,PRKCA
273	WNK Renal Signaling Pathway	6.10E-01	1.92E-02	NaN	NR3C2,PRKCA
274	Oxytocin Signaling Pathway	6.07E-01	1.44E-02	-1	CACNB2,CACNG2,PRKCA,RASD1
275	PPAR Signaling	6.04E-01	1.90E-02	NaN	EP300,RASD1
276	Generic Transcription Pathway	6.00E-01	1.58E-02	NaN	MED14,NR3C1,NR3C2
277	Glutathione-mediated Detoxification	5.95E-01	3.12E-02	NaN	Gsta4
278	MHC class II antigen presentation	5.87E-01	1.85E-02	NaN	OSBPL1A,TUBB3
279	Regulation of the Epithelial-Mesenchymal Transition Pathway	5.84E-01	1.55E-02	NaN	APH1A,RASD1,WNT2
280	Triglyceride metabolism	5.83E-01	3.03E-02	NaN	PPP1CC
281	O-linked glycosylation	5.81E-01	1.83E-02	NaN	GALNT16,GALNT17
282	Role of MAPK Signaling in Promoting the Pathogenesis of Influenza	5.81E-01	1.83E-02	NaN	PRKCA,RASD1
283	mRNA 3 Prime End Processing Signaling Pathway	5.76E-01	1.82E-02	NaN	IGHM,PPP1CC
284	Activin Inhibin Signaling Pathway	5.76E-01	1.53E-02	NaN	EP300,IGHM,PMEPA1
285	Transcriptional Regulation by NPAS4	5.72E-01	2.94E-02	NaN	NR3C1
286	Signaling by the B Cell Receptor (BCR)	5.70E-01	1.80E-02	NaN	IGHM,STIM1
287	Parkinson's Signaling Pathway	5.70E-01	1.39E-02	0	CACNB2,CACNG2,GRIN3A,TUBB3
288	Human Embryonic Stem Cell Pluripotency	5.68E-01	1.52E-02	NaN	PRKCA,RASD1,WNT2

	A	B	C	D	E
289	Transcriptional regulation by the AP-2 (TFAP2) family of transcription f	5.61E-01	2.86E-02	NaN	EP300
290	Role of RIG1-like Receptors in Antiviral Innate Immunity	5.61E-01	2.86E-02	NaN	EP300
291	MIF-mediated Glucocorticoid Regulation	5.61E-01	2.86E-02	NaN	NR3C1
292	Cholecystokinin/Gastrin-mediated Signaling	5.60E-01	1.77E-02	NaN	PRKCA,RASD1
293	Bladder Cancer Signaling	5.60E-01	1.77E-02	NaN	DAPK1,RASD1
294	Virus Entry via Endocytic Pathways	5.54E-01	1.75E-02	NaN	PRKCA,RASD1
295	Beta-catenin independent WNT signaling	5.52E-01	1.49E-02	NaN	PFN1,PRKCA,STK32C
296	Ephrin Receptor Signaling	5.52E-01	1.49E-02	NaN	ARPC2,GRIN3A,RASD1
297	Transcriptional regulation of granulopoiesis	5.51E-01	2.78E-02	NaN	EP300
298	Aggrephagy	5.51E-01	2.78E-02	NaN	TUBB3
299	Fc Epsilon RI Signaling	5.49E-01	1.74E-02	NaN	PRKCA,RASD1
300	Regulation of lipid metabolism by PPARalpha	5.49E-01	1.74E-02	NaN	EP300,MED14
301	Integrin Signaling	5.48E-01	1.48E-02	NaN	ARPC2,PFN1,RASD1
302	B-WICH complex positively regulates rRNA expression	5.41E-01	2.70E-02	NaN	EP300
303	Neuregulin Signaling	5.39E-01	1.71E-02	NaN	PRKCA,RASD1
304	Regulation of Insulin-like Growth Factor (IGF) transport and uptake by	5.39E-01	1.71E-02	NaN	PENK,SPARCL1
305	IL-12 Signaling and Production in Macrophages	5.37E-01	1.46E-02	NaN	EP300,IGHM,PRKCA
306	IL-15 Production	5.34E-01	1.69E-02	NaN	CLK1,DYRK3
307	TP53 Regulates Transcription of Cell Death Genes	5.31E-01	2.63E-02	NaN	PPP1R13B
308	Resolution of Abasic Sites (AP sites)	5.31E-01	2.63E-02	NaN	PARP1
309	Carboxyterminal post-translational modifications of tubulin	5.31E-01	2.63E-02	NaN	TUBB3
310	NGF-stimulated transcription	5.22E-01	2.56E-02	NaN	EP300
311	Pyrimidine Ribonucleotides Interconversion	5.22E-01	2.56E-02	NaN	AK5
312	Cohesin Chromatin Regulation Pathway	5.19E-01	1.42E-02	NaN	MED14,PCDH17,PCDH20
313	Role of NANOG in Mammalian Embryonic Stem Cell Pluripotency	5.14E-01	1.64E-02	NaN	RASD1,WNT2
314	Gap junction trafficking and regulation	5.13E-01	2.50E-02	NaN	TUBB3
315	Bile acid and bile salt metabolism	5.13E-01	2.50E-02	NaN	OSBPL1A
316	Retinoid metabolism and transport	5.13E-01	2.50E-02	NaN	TTR
317	Mechanisms of Viral Exit from Host Cells	5.13E-01	2.50E-02	NaN	PRKCA
318	Response to elevated platelet cytosolic Ca2+	5.05E-01	1.61E-02	NaN	PFN1,PRKCA
319	ROBO SLIT Signaling Pathway	5.05E-01	1.61E-02	NaN	ARPC2,SLIT1
320	RNA Polymerase III Transcription	5.04E-01	2.44E-02	NaN	BRF2
321	Oncostatin M Signaling	5.04E-01	2.44E-02	NaN	RASD1
322	Glioma Signaling	5.00E-01	1.60E-02	NaN	PRKCA,RASD1
323	TR/RXR Activation	4.95E-01	1.59E-02	NaN	ENO1,EP300
324	Regulation of beta-cell development	4.95E-01	2.38E-02	NaN	EP300
325	SUMOylation of transcription cofactors	4.95E-01	2.38E-02	NaN	EP300
326	Vasopressin regulates renal water homeostasis via Aquaporins	4.95E-01	2.38E-02	NaN	ADCY1
327	Formation of WDR5-containing histone-modifying complexes	4.95E-01	2.38E-02	NaN	DR1
328	Pyrimidine Ribonucleotides De Novo Biosynthesis	4.95E-01	2.38E-02	NaN	AK5
329	Thrombin Signaling	4.94E-01	1.38E-02	NaN	ADCY1,PRKCA,RASD1
330	RUNX1 regulates megakaryocyte differentiation and platelet function	4.87E-01	2.33E-02	NaN	EP300
331	BER (Base Excision Repair) Pathway	4.87E-01	2.33E-02	NaN	PARP1
332	Clathrin-mediated endocytosis	4.86E-01	1.56E-02	NaN	ARPC2,EPS15
333	Glycation Signaling Pathway	4.84E-01	1.36E-02	NaN	PRKCA,RASD1,TTR
334	Retinoic acid Mediated Apoptosis Signaling	4.79E-01	2.27E-02	NaN	PARP1
335	HGF Signaling	4.78E-01	1.54E-02	NaN	PRKCA,RASD1
336	Glucocorticoid Receptor Signaling	4.75E-01	1.16E-02	NaN	EP300,Hspa1b,MED14,NR3C1,NR3C2,RASD1
337	CCR3 Signaling in Eosinophils	4.73E-01	1.53E-02	NaN	PRKCA,RASD1
338	NRF2-mediated Oxidative Stress Response	4.71E-01	1.33E-02	NaN	EP300,PRKCA,RASD1
339	Heme signaling	4.71E-01	2.22E-02	NaN	EP300
340	SNARE Signaling Pathway	4.69E-01	1.52E-02	NaN	ADCY1,CACNB2
341	Myelination Signaling Pathway	4.68E-01	1.24E-02	-1	APH1A,EP300,RASD1,WNT2
342	Role of OCT4 in Mammalian Embryonic Stem Cell Pluripotency	4.63E-01	2.17E-02	NaN	PARP1
343	Primary Immunodeficiency Signaling	4.63E-01	2.17E-02	NaN	IGHM
344	Complex IV assembly	4.56E-01	2.13E-02	NaN	HIGD1A
345	Hereditary Breast Cancer Signaling	4.52E-01	1.47E-02	NaN	EP300,RASD1
346	Adipogenesis pathway	4.48E-01	1.46E-02	NaN	FBXW7,KLF3
347	NR1H2 and NR1H3-mediated signaling	4.48E-01	2.08E-02	NaN	EP300
348	Assembly of RNA Polymerase II Complex	4.48E-01	2.08E-02	NaN	DR1
349	G alpha (s) signalling events	4.44E-01	1.45E-02	NaN	ADCY1,MC4R
350	Insulin Receptor Signaling	4.44E-01	1.45E-02	NaN	PPP1CC,RASD1
351	PXR/RXR Activation	4.41E-01	2.04E-02	NaN	NR3C1
352	Melanoma Signaling	4.41E-01	2.04E-02	NaN	RASD1
353	Mitochondrial Dysfunction	4.37E-01	1.19E-02	1	APH1A,CACNB2,CACNG2,FBXW7
354	Signaling by ERBB2	4.34E-01	2.00E-02	NaN	PRKCA
355	Apoptotic execution phase	4.34E-01	2.00E-02	NaN	SATB1
356	Cell Cycle: G2/M DNA Damage Checkpoint Regulation	4.34E-01	2.00E-02	NaN	EP300
357	Endocannabinoid Cancer Inhibition Pathway	4.33E-01	1.42E-02	NaN	ADCY1,CNR1
358	Docosahexaenoic Acid (DHA) Signaling	4.29E-01	1.26E-02	NaN	ADCY1,APH1A,PRKCA
359	Kinesins	4.27E-01	1.96E-02	NaN	TUBB3
360	Transcriptional and post-translational regulation of MITF-M expression	4.27E-01	1.96E-02	NaN	MC4R
361	AMPK Signaling	4.26E-01	1.25E-02	NaN	AK5,EP300,STIM1
362	Deubiquitination	4.23E-01	1.24E-02	NaN	CYLD,EP300,FOXK1
363	Necroptosis Signaling Pathway	4.21E-01	1.39E-02	NaN	CYLD,DAPK1
364	NLR signaling pathways	4.21E-01	1.92E-02	NaN	CYLD
365	UVB-Induced MAPK Signaling	4.21E-01	1.92E-02	NaN	PRKCA
366	FcŒ≥RIIB Signaling in B Lymphocytes	4.18E-01	1.17E-02	NaN	CACNB2,CACNG2,IGHM,RASD1
367	Intrinsic Pathway for Apoptosis	4.14E-01	1.89E-02	NaN	PPP1R13B
368	Signaling by EGFR	4.14E-01	1.89E-02	NaN	EPS15
369	KEAP1-NFE2L2 pathway	4.10E-01	1.36E-02	NaN	EP300,STK32C
370	Signaling by PTK6	4.08E-01	1.85E-02	NaN	NR3C1
371	Lymphotoxin Œ≤ Receptor Signaling	4.08E-01	1.85E-02	NaN	EP300
372	Sensory processing of sound by outer hair cells of the cochlea	4.02E-01	1.82E-02	NaN	RIPOR2
373	Triacylglycerol Degradation	4.02E-01	1.82E-02	NaN	DAGLA
374	EGF Signaling	4.02E-01	1.82E-02	NaN	PRKCA
375	SPINK1 General Cancer Pathway	4.02E-01	1.82E-02	NaN	RASD1
376	Irritable Bowel Syndrome Signaling Pathway	4.01E-01	1.20E-02	NaN	ARPC2,CYLD,WNT2
377	Sperm Motility	4.01E-01	1.20E-02	NaN	CLK1,DYRK3,PRKCA
378	Factors Promoting Cardiogenesis in Vertebrates	4.00E-01	1.33E-02	NaN	PRKCA,WNT2
379	TNF signaling	3.96E-01	1.79E-02	NaN	CYLD
380	CNTF Signaling	3.96E-01	1.79E-02	NaN	RASD1
381	p75 NTR receptor-mediated signalling	3.93E-01	1.32E-02	NaN	APH1A,STK32C
382	Polyamine Regulation in Colon Cancer	3.90E-01	1.75E-02	NaN	STIM1
383	Assembly of collagen fibrils and other multimeric structures	3.84E-01	1.72E-02	NaN	DST
384	SUMOylation of chromatin organization proteins	3.84E-01	1.72E-02	NaN	SATB1

	A	B	C	D	E
385	Cancer Drug Resistance by Drug Efflux	3.84E-01	1.72E-02	NaN	RASD1
386	Ovarian Cancer Signaling	3.83E-01	1.29E-02	NaN	RASD1,WNT2
387	Epithelial Adherens Junction Signaling	3.83E-01	1.29E-02	NaN	ARPC2,RASD1
388	GABA receptor activation	3.73E-01	1.67E-02	NaN	ADCY1
389	Endometrial Cancer Signaling	3.73E-01	1.67E-02	NaN	RASD1
390	HOTAIR Regulatory Pathway	3.73E-01	1.27E-02	NaN	EP300,WNT2
391	UFMylation Signaling Pathway	3.68E-01	1.64E-02	NaN	EP300
392	IL-2 Signaling	3.68E-01	1.64E-02	NaN	RASD1
393	Class I MHC mediated antigen processing and presentation	3.67E-01	1.09E-02	-1	AREL1,FBXW7,RNF114,UBE2E2
394	Signaling by Rho Family GTPases	3.65E-01	1.14E-02	NaN	ARPC2,PIP5K1B,STMN1
395	Colorectal Cancer Metastasis Signaling	3.63E-01	1.14E-02	NaN	ADCY1,RASD1,WNT2
396	Cytosolic sensors of pathogen-associated DNA	3.62E-01	1.61E-02	NaN	EP300
397	Pre-NOTCH Expression and Processing	3.62E-01	1.61E-02	NaN	EP300
398	Abacavir ADME	3.62E-01	1.61E-02	NaN	STK32C
399	Germ Cell-Sertoli Cell Junction Signaling	3.60E-01	1.23E-02	NaN	RASD1,TUBB3
400	Ribavirin ADME	3.57E-01	1.59E-02	NaN	STK32C
401	Stearate Biosynthesis I (Animals)	3.57E-01	1.59E-02	NaN	DHCR24
402	Macrophage Alternative Activation Signaling Pathway	3.57E-01	1.23E-02	NaN	IGHM,NR3C1
403	Translocation of SLC2A4 (GLUT4) to the plasma membrane	3.52E-01	1.56E-02	NaN	TUBB3
404	Semaphorin interactions	3.52E-01	1.56E-02	NaN	PLXND1
405	ERB2-ERBB3 Signaling	3.52E-01	1.56E-02	NaN	RASD1
406	Natural Killer Cell Signaling	3.48E-01	1.20E-02	NaN	Hspa1b,RASD1
407	GŒ±q Signaling	3.45E-01	1.20E-02	NaN	PRKCA,RGS4
408	PKR-mediated signaling	3.43E-01	1.52E-02	NaN	TUBB3
409	WNT/Ca+ pathway	3.43E-01	1.52E-02	NaN	PRKCA
410	Erythropoietin Signaling Pathway	3.42E-01	1.19E-02	NaN	PRKCA,RASD1
411	Glioblastoma Multiforme Signaling	3.39E-01	1.18E-02	NaN	RASD1,WNT2
412	Glutamate Receptor Signaling	3.38E-01	1.49E-02	NaN	GRIN3A
413	Agrin Interactions at Neuromuscular Junction	3.33E-01	1.47E-02	NaN	RASD1
414	Role of JAK1 and JAK3 in Œ≥c Cytokine Signaling	3.33E-01	1.47E-02	NaN	RASD1
415	GM-CSF Signaling	3.33E-01	1.47E-02	NaN	RASD1
416	Activation of anterior HOX genes in hindbrain during early embryogen	3.29E-01	1.45E-02	NaN	EP300
417	Growth Hormone Signaling	3.29E-01	1.45E-02	NaN	PRKCA
418	Basal Cell Carcinoma Signaling	3.29E-01	1.45E-02	NaN	WNT2
419	CLEAR Signaling Pathway	3.24E-01	1.07E-02	NaN	EP300,PRKCA,RASD1
420	Metabolism of water-soluble vitamins and cofactors	3.24E-01	1.43E-02	NaN	PDXK
421	Glioma Invasiveness Signaling	3.24E-01	1.43E-02	NaN	RASD1
422	Granzyme A Signaling	3.24E-01	1.43E-02	NaN	PARP1
423	Melatonin Signaling	3.20E-01	1.41E-02	NaN	PRKCA
424	Protein Sorting Signaling Pathway	3.17E-01	1.13E-02	NaN	ADCY1,WASHC2C
425	Regulation of RUNX2 expression and activity	3.15E-01	1.39E-02	NaN	NR3C1
426	Neuroinflammation Signaling Pathway	3.12E-01	1.05E-02	NaN	APH1A,CALB1,GRIN3A
427	ERK5 Signaling	3.11E-01	1.37E-02	NaN	RASD1
428	IL-17A Signaling in Fibroblasts	3.11E-01	1.37E-02	NaN	PRKCA
429	SUMOylation of DNA damage response and repair proteins	3.07E-01	1.35E-02	NaN	PARP1
430	trans-Golgi Network Vesicle Budding	3.03E-01	1.33E-02	NaN	DTNBP1
431	Leptin Signaling in Obesity	3.03E-01	1.33E-02	NaN	ADCY1
432	Production of Nitric Oxide and Reactive Oxygen Species in Macrophage	2.99E-01	1.09E-02	NaN	PPP1CC,PRKCA
433	Angiopoietin Signaling	2.99E-01	1.32E-02	NaN	RASD1
434	Factors involved in megakaryocyte development and platelet producti	2.95E-01	1.30E-02	NaN	DOCK4
435	Antiproliferative Role of Somatostatin Receptor 2	2.95E-01	1.30E-02	NaN	RASD1
436	Thyroid Cancer Signaling	2.95E-01	1.30E-02	NaN	RASD1
437	IL-7 Signaling Pathway	2.95E-01	1.30E-02	NaN	IGHM
438	Transport of bile salts and organic acids, metal ions and amine compou	2.91E-01	1.28E-02	NaN	SLC6A7
439	RNA polymerase II transcribes snRNA genes	2.91E-01	1.28E-02	NaN	SRRT
440	Neurotrophin/TRK Signaling	2.91E-01	1.28E-02	NaN	RASD1
441	Mitotic G2-G2/M phases	2.89E-01	1.06E-02	NaN	EP300,TUBB3
442	Signaling by MET	2.87E-01	1.27E-02	NaN	EPS15
443	Xenobiotic Metabolism AHR Signaling Pathway	2.87E-01	1.27E-02	NaN	EP300
444	Post-translational modification: synthesis of GPI-anchored proteins	2.84E-01	1.25E-02	NaN	MDGA1
445	Estrogen-Dependent Breast Cancer Signaling	2.84E-01	1.25E-02	NaN	RASD1
446	Multiple Sclerosis Signaling Pathway	2.82E-01	1.05E-02	NaN	GRIN3A,PARP1
447	PI Metabolism	2.80E-01	1.23E-02	NaN	PIP5K1B
448	Glucose metabolism	2.80E-01	1.23E-02	NaN	ENO1
449	FLT3 Signaling in Hematopoietic Progenitor Cells	2.80E-01	1.23E-02	NaN	RASD1
450	BAG2 Signaling Pathway	2.80E-01	1.23E-02	NaN	Hspa1b
451	CDX Gastrointestinal Cancer Signaling Pathway	2.78E-01	1.04E-02	NaN	LCT,WNT2
452	Mitotic Prometaphase	2.78E-01	1.04E-02	NaN	PPP1CC,TUBB3
453	JAK/STAT Signaling	2.76E-01	1.22E-02	NaN	RASD1
454	CCR5 Signaling in Macrophages	2.70E-01	9.71E-03	NaN	CACNB2,CACNG2,PRKCA
455	PEDF Signaling	2.69E-01	1.19E-02	NaN	RASD1
456	Adrenomedullin signaling pathway	2.69E-01	1.02E-02	NaN	ADCY1,RASD1
457	ID1 Signaling Pathway	2.64E-01	1.01E-02	NaN	EP300,RASD1
458	HIPPO signaling	2.63E-01	1.16E-02	NaN	PPP1CC
459	FGF Signaling	2.63E-01	1.16E-02	NaN	PRKCA
460	Role of Tissue Factor in Cancer	2.62E-01	1.00E-02	NaN	PRKCA,RASD1
461	Clathrin-mediated Endocytosis Signaling	2.60E-01	9.95E-03	NaN	ARPC2,EPS15
462	Mitotic Prophase	2.59E-01	1.15E-02	NaN	PRKCA
463	BMP signaling pathway	2.59E-01	1.15E-02	NaN	RASD1
464	Apelin Adipocyte Signaling Pathway	2.59E-01	1.15E-02	NaN	ADCY1
465	IL-8 Signaling	2.58E-01	9.90E-03	NaN	PRKCA,RASD1
466	Regulation of Cellular Mechanics by Calpain Protease	2.56E-01	1.14E-02	NaN	RASD1
467	NER (Nucleotide Excision Repair, Enhanced Pathway)	2.56E-01	1.14E-02	NaN	EP300
468	ISGylation Signaling Pathway	2.53E-01	1.12E-02	NaN	EP300
469	Regulation of mRNA stability by proteins that bind AU-rich elements	2.53E-01	1.12E-02	NaN	PRKCA
470	Unfolded protein response	2.53E-01	1.12E-02	NaN	Hspa1b
471	mTOR Signaling	2.52E-01	9.76E-03	NaN	PRKCA,RASD1
472	Cell junction organization	2.50E-01	1.11E-02	NaN	DST
473	Pulmonary Fibrosis Idiopathic Signaling Pathway	2.49E-01	9.35E-03	NaN	EP300,RASD1,WNT2
474	Acute Myeloid Leukemia Signaling	2.47E-01	1.10E-02	NaN	RASD1
475	Hepatic Cholestasis	2.42E-01	9.52E-03	NaN	ADCY1,PRKCA
476	Death Receptor Signaling	2.41E-01	1.08E-02	NaN	PARP1
477	Class B/2 (Secretin family receptors)	2.38E-01	1.06E-02	NaN	WNT2
478	Cellular response to heat stress	2.35E-01	1.05E-02	NaN	EP300
479	Transcriptional regulation by RUNX3	2.35E-01	1.05E-02	NaN	EP300
480	IL-1 Signaling	2.32E-01	1.04E-02	NaN	ADCY1

	A	B	C	D	E
481	Chromatin organization	2.32E-01	9.30E-03	NaN	DR1,EP300
482	ATM Signaling	2.29E-01	1.03E-02	NaN	PPP1CC
483	Apelin Cardiomyocyte Signaling Pathway	2.29E-01	1.03E-02	NaN	PRKCA
484	EIF2 Signaling	2.28E-01	9.22E-03	NaN	PPP1CC,RASD1
485	ESR-mediated signaling	2.13E-01	9.71E-03	NaN	EP300
486	Mouse Embryonic Stem Cell Pluripotency	2.13E-01	9.71E-03	NaN	RASD1
487	IGF-1 Signaling	2.13E-01	9.71E-03	NaN	RASD1
488	Extracellular matrix organization	2.11E-01	9.62E-03	NaN	TTR
489	Cargo recognition for clathrin-mediated endocytosis	2.11E-01	9.62E-03	NaN	EPS15
490	MSP-RON Signaling in Macrophages Pathway	2.11E-01	9.62E-03	NaN	RASD1
491	Paxillin Signaling	2.06E-01	9.43E-03	NaN	RASD1
492	Telomerase Signaling	2.06E-01	9.43E-03	NaN	RASD1
493	Signaling by VEGF	2.03E-01	9.35E-03	NaN	PRKCA
494	Nucleotide Excision Repair	2.01E-01	9.26E-03	NaN	PARP1
495	Th1 Pathway	2.01E-01	9.26E-03	NaN	APH1A
496	Kinetochore Metaphase Signaling Pathway	2.01E-01	9.26E-03	NaN	PPP1CC
497	NAD Signaling Pathway	0.00E+00	7.09E-03	NaN	PARP1
498	Wound Healing Signaling Pathway	0.00E+00	8.20E-03	NaN	PRKCA,RASD1
499	MicroRNA Biogenesis Signaling Pathway	0.00E+00	5.56E-03	NaN	RASD1
500	Pathogen Induced Cytokine Storm Signaling Pathway	0.00E+00	3.16E-03	NaN	CCL27
501	Role of Osteoblasts in Rheumatoid Arthritis Signaling Pathway	0.00E+00	8.62E-03	NaN	CSNK1A1,WNT2
502	Role of Osteoclasts in Rheumatoid Arthritis Signaling Pathway	0.00E+00	3.31E-03	NaN	RASD1
503	Ribonucleotide Reductase Signaling Pathway	0.00E+00	6.17E-03	NaN	PARP1
504	Neutrophil Extracellular Trap Signaling Pathway	0.00E+00	8.47E-03	NaN	IGHM,PRKCA,STIM1
505	Leukocyte Extravasation Signaling	0.00E+00	5.35E-03	NaN	PRKCA
506	Chaperone Mediated Autophagy Signaling Pathway	0.00E+00	4.60E-03	NaN	EP300,Hspa1b
507	NOD1/2 Signaling Pathway	0.00E+00	6.10E-03	NaN	Hspa1b
508	Sertoli Cell-Germ Cell Junction Signaling Pathway (Enhanced)	0.00E+00	8.66E-03	NaN	ARPC2,RASD1
509	Aryl Hydrocarbon Receptor Signaling	0.00E+00	6.76E-03	NaN	EP300
510	LPS/IL-1 Mediated Inhibition of RXR Function	0.00E+00	8.47E-03	NaN	HS6ST2,UST
511	PIP3 activates AKT signaling	0.00E+00	6.85E-03	NaN	PIP5K1B
512	Cell surface interactions at the vascular wall	0.00E+00	8.70E-03	NaN	IGHM
513	Asparagine N-linked glycosylation	0.00E+00	8.20E-03	NaN	UAP1
514	Mitotic Metaphase and Anaphase	0.00E+00	8.93E-03	NaN	PPP1CC,TUBB3
515	Costimulation by the CD28 family	0.00E+00	8.93E-03	NaN	STK32C
516	G alpha (q) signalling events	0.00E+00	6.10E-03	NaN	RGS4
517	Cilium Assembly	0.00E+00	5.10E-03	NaN	TUBB3
518	C-type lectin receptors (CLRs)	0.00E+00	7.46E-03	NaN	EP300
519	Neutrophil degranulation	0.00E+00	4.81E-03	NaN	PDXK,TTR
520	Intra-Golgi and retrograde Golgi-to-ER traffic	0.00E+00	5.26E-03	NaN	TUBB3
521	Cell Cycle Checkpoints	0.00E+00	3.88E-03	NaN	PPP1CC
522	Processing of Capped Intron-Containing Pre-mRNA	0.00E+00	3.56E-03	NaN	SRRT
523	Transcriptional regulation by RUNX1	0.00E+00	7.35E-03	NaN	EP300
524	Neddylation	0.00E+00	8.26E-03	NaN	DDA1,FBXW7
525	LXR/RXR Activation	0.00E+00	8.77E-03	NaN	TTR
526	HEY1 Signaling Pathway	0.00E+00	6.37E-03	NaN	APH1A
527	Histone Modification Signaling Pathway	0.00E+00	7.33E-03	NaN	EP300,PRDM8
528	Nuclear Cytoskeleton Signaling Pathway	0.00E+00	4.76E-03	NaN	TUBB3
529	Hematoma Resolution Signaling Pathway	0.00E+00	8.16E-03	NaN	GRIN3A,MC4R
530	Cyclophilin Signaling Pathway	0.00E+00	8.47E-03	NaN	EP300,WNT2
531	TRIM21 Intracellular Antibody Signaling Pathway	0.00E+00	2.67E-03	NaN	IGHM
532	RAR Activation	0.00E+00	7.26E-03	NaN	ADCY1,EP300,PARP1
533	Role of Pattern Recognition Receptors in Recognition of Bacteria and V	0.00E+00	7.04E-03	NaN	PRKCA
534	Role of PKR in Interferon Induction and Antiviral Response	0.00E+00	8.33E-03	NaN	Hspa1b
535	Role of NFAT in Regulation of the Immune Response	0.00E+00	4.63E-03	NaN	CSNK1A1,IGHM,RASD1
536	Calcium-induced T Lymphocyte Apoptosis	0.00E+00	7.66E-03	NaN	EP300,PRKCA
537	IL-17 Signaling	0.00E+00	6.25E-03	NaN	RASD1
538	CTLA4 Signaling in Cytotoxic T Lymphocytes	0.00E+00	2.53E-03	NaN	RASD1
539	CD28 Signaling in T Helper Cells	0.00E+00	3.13E-03	NaN	ARPC2
540	IL-15 Signaling	0.00E+00	5.90E-03	NaN	IGHM,RASD1
541	Dendritic Cell Maturation	0.00E+00	2.66E-03	NaN	IGHM
542	Relaxin Signaling	0.00E+00	6.54E-03	NaN	ADCY1
543	HMGB1 Signaling	0.00E+00	6.41E-03	NaN	RASD1
544	Prostate Cancer Signaling	0.00E+00	8.85E-03	NaN	RASD1
545	Allograft Rejection Signaling	0.00E+00	3.73E-03	NaN	IGHM
546	Autoimmune Thyroid Disease Signaling	0.00E+00	4.05E-03	NaN	IGHM
547	Chronic Myeloid Leukemia Signaling	0.00E+00	7.27E-03	NaN	RASD1,WNT2
548	GŒ±12/13 Signaling	0.00E+00	7.69E-03	NaN	RASD1
549	p70S6K Signaling	0.00E+00	7.67E-03	NaN	IGHM,PRKCA,RASD1
550	G Protein Signaling Mediated by Tubby	0.00E+00	3.56E-03	NaN	NPY2R
551	Communication between Innate and Adaptive Immune Cells	0.00E+00	1.85E-03	NaN	IGHM
552	Sphingosine-1-phosphate Signaling	0.00E+00	8.93E-03	NaN	ADCY1
553	Systemic Lupus Erythematosus Signaling	0.00E+00	3.12E-03	NaN	IGHM,RASD1
554	CDC42 Signaling	0.00E+00	2.77E-03	NaN	ARPC2
555	PAK Signaling	0.00E+00	8.70E-03	NaN	RASD1
556	Role of Osteoblasts, Osteoclasts and Chondrocytes in Rheumatoid Arth	0.00E+00	9.05E-03	NaN	CSNK1A1,WNT2
557	Phospholipase C Signaling	0.00E+00	6.83E-03	NaN	ADCY1,EP300,IGHM,PRKCA,RASD1
558	HER-2 Signaling in Breast Cancer	0.00E+00	8.85E-03	NaN	PRKCA,RASD1
559	Altered T Cell and B Cell Signaling in Rheumatoid Arthritis	0.00E+00	1.82E-03	NaN	IGHM
560	Regulation of eIF4 and p70S6K Signaling	0.00E+00	5.62E-03	NaN	RASD1
561	B Cell Development	0.00E+00	3.46E-03	NaN	IGHM
562	Regulation of IL-2 Expression in Activated and Anergic T Lymphocytes	0.00E+00	3.33E-03	NaN	RASD1
563	NUR77 Signaling in T Lymphocytes	0.00E+00	6.62E-03	NaN	EP300,PRKCA
564	PKCŒ∏ Signaling in T Lymphocytes	0.00E+00	8.36E-03	NaN	CACNB2,CACNG2,RASD1
565	PI3K Signaling in B Lymphocytes	0.00E+00	4.99E-03	NaN	IGHM,RASD1
566	NGF Signaling	0.00E+00	8.26E-03	NaN	RASD1
567	Sertoli Cell-Sertoli Cell Junction Signaling	0.00E+00	8.44E-03	NaN	RASD1,TUBB3
568	Hematopoiesis from Pluripotent Stem Cells	0.00E+00	4.10E-03	NaN	IGHM
569	Ephrin A Signaling	0.00E+00	7.30E-03	NaN	APH1A
570	Agranulocyte Adhesion and Diapedesis	0.00E+00	5.43E-03	NaN	CCL27
571	Granulocyte Adhesion and Diapedesis	0.00E+00	6.06E-03	NaN	CCL27
572	TEC Kinase Signaling	0.00E+00	2.60E-03	NaN	PRKCA
573	STAT3 Pathway	0.00E+00	7.30E-03	NaN	RASD1
574	SAPK/JNK Signaling	0.00E+00	3.18E-03	NaN	RASD1
575	PI3K/AKT Signaling	0.00E+00	5.08E-03	NaN	RASD1
576	PTEN Signaling	0.00E+00	6.67E-03	NaN	RASD1

	A	B	C	D	E
577	Protein Ubiquitination Pathway	0.00E+00	7.55E-03	NaN	FBXW7,UBE2E2
578	IL-4 Signaling	0.00E+00	5.39E-03	NaN	NR3C1,RASD1
579	B Cell Receptor Signaling	0.00E+00	4.51E-03	NaN	IGHM,RASD1
580	NF-ŒJB Signaling	0.00E+00	5.31E-03	NaN	EP300,RASD1
581	T Cell Receptor Signaling	0.00E+00	4.91E-03	NaN	DUSP5,RASD1
582	IL-6 Signaling	0.00E+00	8.06E-03	NaN	RASD1
583	Phagosome Maturation	0.00E+00	6.62E-03	NaN	TUBB3
584	Autophagy	0.00E+00	4.67E-03	NaN	DAPK1
585	Th1 and Th2 Activation Pathway	0.00E+00	6.41E-03	NaN	APH1A
586	Th2 Pathway	0.00E+00	8.06E-03	NaN	APH1A
587	GP6 Signaling Pathway	0.00E+00	8.00E-03	NaN	PRKCA
588	Sirtuin Signaling Pathway	0.00E+00	3.61E-03	NaN	PARP1
589	T Cell Exhaustion Signaling Pathway	0.00E+00	2.87E-03	NaN	RASD1
590	Systemic Lupus Erythematosus in T Cell Signaling Pathway	0.00E+00	4.72E-03	NaN	RASD1,STIM1
591	Systemic Lupus Erythematosus in B Cell Signaling Pathway	0.00E+00	5.85E-03	NaN	IGHM,PRKCA,RASD1
592	Semaphorin Neuronal Repulsive Signaling Pathway	0.00E+00	6.99E-03	NaN	PLXND1
593	Regulation of the Epithelial Mesenchymal Transition by Growth Factor	0.00E+00	5.21E-03	NaN	RASD1
594	Coronavirus Pathogenesis Pathway	0.00E+00	5.32E-03	NaN	EP300
595	Coronavirus Replication Pathway	0.00E+00	7.35E-03	NaN	TUBB3
596	Tumor Microenvironment Pathway	0.00E+00	5.85E-03	NaN	RASD1
597	MSP-RON Signaling in Cancer Cells Pathway	0.00E+00	7.25E-03	NaN	RASD1
598	Ferroptosis Signaling Pathway	0.00E+00	8.13E-03	NaN	RASD1
599					
600					

	A	B	C	D	E
1	**Supplementary Table 2- IPA pathways CA2**				
2					
3	¬© 2000-2024 QIAGEN. All rights reserved.				
4					
5	**Ingenuity Canonical Pathways**	**-log(p-value**	**Ratio**	**z-score**	**Molecules**
6	Cardiac Hypertrophy Signaling	3.30E+00	5.56E-02	-2.333	ADCY1,ADCY5,CACNA1C,CACNA2D1,CACNA2D3,CACNG3,GNB5,IGF1R,PIK3R4,PLCH2,PLCL1,PRKAG2,RASD2,TGFB2
7	RHO GTPase cycle	1.52E+00	3.39E-02	-2.324	AMIGO2,ARHGEF26,ARHGEF4,CAPZB,COPS4,CPNE8,DOCK11,DOCK3,PCDH7,PEAK1,PLXNA1,RALGAPA1,SLC4A7,SRGAP3,WIPF3
8	Role of Osteoblasts in Rheumatoid Arthritis Signaling Pathway	1.05E+00	3.45E-02	-2.121	BMP3,CSNK1A1,FRZB,IGF1R,MMP17,PIK3R4,PTGS2,TGFB2
9	Transport of inorganic cations/anions and amino acids/oligopeptid	8.04E-01	3.77E-02	-2	AHCYL2,SLC4A7,SLC7A1,SLC9A4
10	Dopamine-DARPP32 Feedback in cAMP Signaling	4.74E+00	7.61E-02	-1.897	ADCY1,ADCY5,CACNA1C,CACNA2D1,CACNA2D3,CACNG3,CSNK1A1,ITPR1,PLCH2,PLCL1,PPP1CC,PRKAG2,PRKCA,PRKCB
11	D-myo-inositol-5-phosphate Metabolism	3.75E+00	6.50E-02	-1.89	DUSP5,DUSP6,G6PC3,INPP5F,MTMR9,PLCH2,PLPP6,PLPPR4,PPFIA2,PPP1CC,PTPRD,PTPRO,SIRPA
12	D-myo-inositol (1,4,5,6)-Tetrakisphosphate Biosynthesis	3.52E+00	6.52E-02	-1.633	DUSP5,DUSP6,G6PC3,INPP5F,MTMR9,PLPP6,PLPPR4,PPFIA2,PPP1CC,PTPRD,PTPRO,SIRPA
13	D-myo-inositol (3,4,5,6)-tetrakisphosphate Biosynthesis	3.52E+00	6.52E-02	-1.633	DUSP5,DUSP6,G6PC3,INPP5F,MTMR9,PLPP6,PLPPR4,PPFIA2,PPP1CC,PTPRD,PTPRO,SIRPA
14	3-phosphoinositide Degradation	3.29E+00	6.15E-02	-1.633	DUSP5,DUSP6,G6PC3,INPP5F,MTMR9,PLPP6,PLPPR4,PPFIA2,PPP1CC,PTPRD,PTPRO,SIRPA
15	Cardiac Œ≤-adrenergic Signaling	3.10E+00	6.21E-02	-1.633	ADCY1,ADCY5,CACNA1C,CACNA2D1,CACNA2D3,CACNG3,GNB5,PDE7A,PKIA,PPP1CC,PRKAG2
16	Colorectal Cancer Metastasis Signaling	1.43E+00	3.79E-02	-1.633	ADCY1,ADCY5,GNB5,MMP17,PIK3R4,PRKAG2,PTGS2,RASD2,SMAD3,TGFB2
17	Hepatic Cholestasis	6.29E-01	2.86E-02	-1.633	ADCY1,ADCY5,PRKAG2,PRKCA,PRKCB,TGFB2
18	3-phosphoinositide Biosynthesis	4.11E+00	6.67E-02	-1.414	CDIPT,DUSP5,DUSP6,G6PC3,INPP5F,MTMR9,PIK3R4,PLPP6,PLPPR4,PPFIA2,PPP1CC,PTPRD,PTPRO,SIRPA
19	P2Y Purinergic Receptor Signaling Pathway	3.56E+00	7.63E-02	-1.414	ADCY1,ADCY5,GNB5,PIK3R4,PLCH2,PLCL1,PRKAG2,PRKCA,PRKCB,RASD2
20	PPARŒ±/RXRŒ± Activation	2.87E+00	5.82E-02	-1.414	ADCY1,ADCY5,GPD2,PLCH2,PLCL1,PRKAG2,PRKCA,PRKCB,RASD2,SMAD3,TGFB2
21	Neutrophil degranulation	0.00E+00	1.92E-02	-1.414	APRT,COTL1,GOLGA7,HSPA1A,LAMTOR2,OSTF1,PRDX6,SIRPA
22	Cachexia Signaling Pathway	1.97E+00	3.97E-02	-1.387	ADCY1,ADCY5,EIF2S2,HSPA1A,Hspa1b,HSPA2,IGF1R,NR3C1,PIK3R4,PRKAG2,PRKCA,PRKCB,SMAD3,TGFB2
23	Serotonin Receptor Signaling	3.13E+00	4.41E-02	-1.342	ADCY1,ADCY5,CACNA1C,CACNA2D1,CACNA2D3,CACNG3,G6PC3,GAP43,GNB5,KCNQ3,PIK3R4,PLCH2,PLCL1,PRDX6,
24					PRKAG2,PRKCA,PRKCB,PTGS2,RASD2,TGFB2
25	Melanocyte Development and Pigmentation Signaling	1.91E+00	6.19E-02	-1.342	ADCY1,ADCY5,KIT,PIK3R4,PRKAG2,RASD2
26	Melatonin Signaling	1.88E+00	7.04E-02	-1.342	PLCH2,PLCL1,PRKAG2,PRKCA,PRKCB
27	NAFLD Signaling Pathway	3.75E-01	2.31E-02	-1.342	G6PC3,PIK3R4,PRKAG2,PTGS2,TGFB2
28	Docosahexaenoic Acid (DHA) Signaling	4.07E+00	6.28E-02	-1.291	ADCY1,ADCY5,FABP3,ITPR1,PIK3R4,PLCH2,PLCL1,PRDX6,PRKAG2,PRKCA,PRKCB,PTGS2,SYT13,SYT17,TGFB2
29	Estrogen Receptor Signaling	3.37E+00	4.73E-02	-1.291	ADCY1,ADCY5,ARG2,CACNA1C,CACNA2D1,CACNA2D3,CACNG3,GNB5,IGF1R,MED10,MMP17,NR3C1,PIK3R4,PLCH2,PLCL1,
30					PRKAG2,PRKCA,PRKCB,RASD2
31	Lung Ionic Balance Signaling Pathway	1.23E+00	3.09E-02	-1.291	ADORA1,ASIC2,GPR12,GPR137C,GPR155,GPR27,HCN1,MAS1,NPBWR1,NTSR2,PLCH2,PLCL1,PRKAG2,PRKCA,PRKCB
32	Glutaminergic Receptor Signaling Pathway (Enhanced)	6.43E+00	6.94E-02	-1.279	ADCY1,ADCY5,CACNA1C,CACNA2D1,CACNA2D3,CACNG3,GABRA1,GABRB3,GRIA3,GRIK4,ITPR1,NAPG,NR3C1,PIK3R4,
33					PLCH2,PLCL1,PRDX6,PRKAG2,PRKCA,PRKCB,TGFB2,VTI1B
34	BBSome Signaling Pathway	3.70E-01	2.16E-02	-1.265	ADORA1,GPR12,GPR137C,GPR155,GPR27,IGF1R,MAS1,NPBWR1,NTSR2,PRKAG2
35	CREB Signaling in Neurons	4.39E+00	4.62E-02	-1.177	ADCY1,ADCY5,ADORA1,CACNA1C,CACNA2D1,CACNA2D3,CACNG3,GNB5,GPR12,GPR137C,GPR155,GPR27,GRIA3,GRIK4,
36					IGF1R,ITPR1,MAS1,NPBWR1,NTSR2,PIK3R4,PLCH2,PLCL1,PRKAG2,PRKCA,PRKCB,RASD2,TGFB2
37	GPCR-Mediated Nutrient Sensing in Enteroendocrine Cells	5.46E+00	1.03E-01	-1.155	ADCY1,ADCY5,CACNA1C,CACNA2D1,CACNA2D3,CACNG3,ITPR1,PLCH2,PLCL1,PRKAG2,PRKCA,PRKCB
38	White Adipose Tissue Browning Pathway	2.23E+00	5.84E-02	-1.134	ADCY1,ADCY5,CACNA1C,CACNA2D1,CACNA2D3,CACNG3,ITPR1,PRKAG2
39	Signaling by ROBO receptors	1.45E+00	3.98E-02	-1.134	FLRT3,LHX2,PFN1,PRKCA,ROBO1,SLIT1,SLIT2,SRGAP3,UPF2
40	Sheddase Signaling Pathway	1.13E+00	3.78E-02	-1.134	CCN3,E2F6,ETS2,IGFBP4,MMP17,PIK3R4,TGFB2
41	G-Protein Coupled Receptor Signaling	1.56E+00	3.11E-02	-1.091	ADCY1,ADCY5,ADORA1,DUSP6,GNB5,GPR12,GPR137C,GPR155,GPR27,HCN1,KCNQ3,MAS1,NPBWR1,NTSR2,PDE7A,PIK3R4,
42					PRKAG2,PRKCA,PRKCB,RASD2,RGS14
43	Molecular Mechanisms of Cancer	1.24E+00	2.80E-02	-1.043	ADCY1,ADCY5,ADORA1,ARHGEF4,BMP3,E2F6,GNB5,GPR12,GPR137C,GPR155,GPR27,ITGA7,MAS1,MMP17,NPBWR1,NTSR2,
44					PIK3R4,PRKAG2,PRKCA,PRKCB,RASD2,SMAD3,TGFB2
45	Role of NFAT in Cardiac Hypertrophy	6.96E+00	8.48E-02	-1	ADCY1,ADCY5,CACNA1C,CACNA2D1,CACNA2D3,CACNG3,CAMK1,CSNK1A1,GNB5,IGF1R,ITPR1,PIK3R4,PLCH2,PLCL1,PRKAG2,
46					PRKCA,PRKCB,RASD2,TGFB2
47	Corticotropin Releasing Hormone Signaling	3.68E+00	7.28E-02	-1	ADCY1,ADCY5,CACNA1C,CACNA2D1,CACNA2D3,CACNG3,ITPR1,PRKAG2,PRKCA,PRKCB,PTGS2
48	Integration of energy metabolism	3.57E+00	8.41E-02	-1	ADCY1,ADCY5,CACNA1C,GNB5,ITPR1,KCNG2,MARCKS,PRKAG2,PRKCA
49	Xenobiotic Metabolism PXR Signaling Pathway	3.31E+00	6.59E-02	-1	CHST2,GSTO1,HS3ST4,HS6ST2,MGST3,PPP1CC,PRKAG2,PRKCA,PRKCB,SULT2B1,WSCD1
50	Cardiac Hypertrophy Signaling (Enhanced)	3.08E+00	4.17E-02	-1	ADCY1,ADCY5,CACNA1C,CACNA2D1,CACNA2D3,CACNG3,FGF11,GNB5,IGF1R,INPP5F,ITGA7,ITPR1,PDE7A,PIK3R4,PLCH2,PLCL1,
51					PRKAG2,PRKCA,PRKCB,PTGS2,RASD2,TGFB2
52	SNARE Signaling Pathway	2.91E+00	6.82E-02	-1	ADCY1,ADCY5,CACNA2D1,CPLX2,NAPG,PRKAG2,SYT13,SYT17,VTI1B
53	Dilated Cardiomyopathy Signaling Pathway	2.59E+00	6.12E-02	-1	ADCY1,ADCY5,CACNA1C,CACNA2D1,CACNA2D3,CACNG3,CAMK1,ITPR1,PRKAG2
54	DAG and IP3 signaling	2.11E+00	1.00E-01	-1	ADCY1,ADCY5,ITPR1,PRKCA
55	UVA-Induced MAPK Signaling	1.91E+00	6.19E-02	-1	PARP1,PIK3R4,PLCH2,PLCL1,PRKCA,RASD2
56	CDK5 Signaling	1.61E+00	5.31E-02	-1	ADCY1,ADCY5,EGR1,PPP1CC,PRKAG2,RASD2
57	Factors involved in megakaryocyte development and platelet produ	1.18E+00	5.19E-02	-1	CABLES2,CAPZB,DOCK11,DOCK3
58	Relaxin Signaling	1.08E+00	3.92E-02	-1	ADCY1,ADCY5,GNB5,PDE7A,PIK3R4,PRKAG2
59	Reelin Signaling in Neurons	8.43E-01	3.57E-02	-1	ARHGEF26,ARHGEF4,ARPC2,PDK2,PIK3R4
60	Actin Cytoskeleton Signaling	7.24E-01	2.95E-02	-1	ARHGEF4,ARPC2,FGF11,ITGA7,PFN1,PIK3R4,RASD2
61	Ribonucleotide Reductase Signaling Pathway	6.65E-01	3.09E-02	-1	E2F6,FBXL5,NFYB,PARP1,PIK3R4
62	RHO GTPases Activate Formins	5.87E-01	3.05E-02	-1	CLIP1,PFN1,PPP1CC,TUBB3
63	Role of Chondrocytes in Rheumatoid Arthritis Signaling Pathway	5.52E-01	2.94E-02	-1	EGR1,MMP17,PRKAG2,PTGS2
64	NAD Signaling Pathway	5.19E-01	2.84E-02	-1	PARP1,PIK3R4,PRKAG2,TGFB2
65	IL-33 Signaling Pathway	4.12E-01	2.50E-02	-1	KIT,PIK3R4,PRKAG2,PTGS2
66	RAR Activation	3.69E-01	2.18E-02	-1	ADCY1,ADCY5,KIT,PARP1,PDE7A,PIK3R4,PRKAG2,SMAD3,TGFB2
67	Irritable Bowel Syndrome Signaling Pathway	0.00E+00	1.61E-02	-1	ARPC2,MMP17,PIK3R4,TGFB2
68	Phagosome Formation	1.05E+00	2.79E-02	-0.943	ADORA1,ARPC2,CLIP1,GPR12,GPR137C,GPR155,GPR27,ITGA7,ITPR1,MARCKS,MAS1,NPBWR1,NTSR2,PIK3R4,PRDX6,
69					PRKCA,PRKCB,RASD2
70	Superpathway of Inositol Phosphate Compounds	5.29E+00	7.17E-02	-0.905	CDIPT,DUSP5,DUSP6,G6PC3,INPP5A,INPP5F,ITPKA,MTMR9,PIK3R4,PLCH2,PLPP6,PLPPR4,PPFIA2,PPP1CC,PTPRD,PTPRO,SIRPA
71	Gustation Pathway	4.11E+00	7.07E-02	-0.832	ADCY1,ADCY5,ASIC2,CACNA1C,CACNA2D1,CACNA2D3,CACNG3,GABRA1,GABRB3,ITPR1,KCNQ3,LRRC8D,PRKAG2
72	Acetylcholine Receptor Signaling Pathway	4.00E+00	6.88E-02	-0.832	ADCY1,ADCY5,CACNA1C,CACNA2D1,CACNA2D3,CACNG3,ITPR1,PIK3R4,PLCH2,PLCL1,PRKAG2,PRKCA,PRKCB
73	Adrenergic Receptor Signaling Pathway (Enhanced)	3.93E+00	6.77E-02	-0.832	ADCY1,ADCY5,CACNA1C,CACNA2D1,CACNA2D3,CACNG3,ITPR1,PLCH2,PLCL1,PRKAG2,PRKCA,PRKCB,TGFB2
74	Eicosanoid Signaling	2.52E+00	4.78E-02	-0.832	ADCY1,ADCY5,EGR1,GNB5,PIK3R4,PLCH2,PLCL1,PRDX6,PRKAG2,PRKCA,PRKCB,PTGS2,RASD2
75	Heparan Sulfate Biosynthesis (Late Stages)	3.06E+00	8.97E-02	-0.816	CHST2,EXTL2,HS3ST4,HS6ST2,PRDX6,SULT2B1,WSCD1
76	Sperm Motility	2.85E+00	5.22E-02	-0.816	EPHB3,GNB5,IGF1R,ITPR1,KIT,PEAK1,PLCH2,PLCL1,PRDX6,PRKAG2,PRKCA,PRKCB,TYRO3
77	Heparan Sulfate Biosynthesis	2.84E+00	8.24E-02	-0.816	CHST2,EXTL2,HS3ST4,HS6ST2,PRDX6,SULT2B1,WSCD1
78	S100 Family Signaling Pathway	2.10E+00	3.37E-02	-0.816	ADORA1,CACNA1C,CACNA2D1,CACNA2D3,CACNG3,GPR12,GPR137C,GPR155,GPR27,ITPR1,MAS1,MMP17,NPBWR1,NR3C1,
79					NTF3,NTSR2,PIK3R4,PLCH2,PLCL1,PRKAG2,PRKCA,PRKCB,SMAD3,TGFB2
80	WNK Renal Signaling Pathway	1.77E+00	5.77E-02	-0.816	ASIC2,LRRC8D,NR3C2,PIK3R4,PRKCA,PRKCB
81	Protein Sorting Signaling Pathway	1.21E+00	3.95E-02	-0.816	ADCY1,ADCY5,GGA3,PRKAG2,SNX11,TMED9,WASHC2C
82	Factors Promoting Cardiogenesis in Vertebrates	1.11E+00	4.00E-02	-0.816	BMP3,PLCH2,PLCL1,PRKCA,PRKCB,TGFB2
83	Epithelial Adherens Junction Signaling	1.06E+00	3.87E-02	-0.816	ARPC2,IGF1R,NECTIN1,PRKAG2,RASD2,TGFB2
84	G alpha (i) signalling events	5.72E-01	2.73E-02	-0.816	ADCY1,ADCY5,ADORA1,GNB5,NPBWR1,RGS14
85	Insulin Secretion Signaling Pathway	5.63E+00	6.93E-02	-0.775	ADCY1,ADCY5,ASIC2,CACNA1C,CACNA2D1,CACNA2D3,CACNG3,DLAT,EIF2S2,ITPR1,NAPG,PCSK2,PIK3R4,PLCH2,PLCL1,
86					PRKAG2,PRKCA,PRKCB,VTI1B
87	Orexin Signaling Pathway	4.17E+00	6.41E-02	-0.775	ADCY1,ADCY5,CACNA1C,CACNA2D1,CACNA2D3,CACNG3,GNB5,ITPR1,KCNK1,PIK3R4,PLCH2,PLCL1,PRKAG2,PRKCA,PRKCB
88	HSP90 chaperone cycle for steroid hormone receptors in the prese	5.47E+00	1.70E-01	-0.707	CAPZB,DCTN6,FKBP5,HSPA1A,HSPA2,NR3C1,NR3C2,TUBB3
89	Synaptic Long Term Potentiation	4.21E+00	8.33E-02	-0.707	ADCY1,CACNA1C,GRIA3,ITPR1,PLCH2,PLCL1,PPP1CC,PRKAG2,PRKCA,PRKCB,RASD2
90	Aldosterone Signaling in Epithelial Cells	3.29E+00	6.55E-02	-0.707	ASIC2,HSPA12A,HSPA1A,HSPA2,ITPR1,NR3C2,PIK3R4,PLCH2,PLCL1,PRKCA,PRKCB
91	Androgen Signaling	2.74E+00	5.99E-02	-0.707	CACNA1C,CACNA2D1,CACNA2D3,CACNG3,GNB5,ITPR1,PRKAG2,PRKCA,PRKCB,SMAD3
92	Xenobiotic Metabolism CAR Signaling Pathway	2.21E+00	5.36E-02	-0.707	CHST2,GSTO1,HS3ST4,HS6ST2,MGST3,PRKCA,PRKCB,SULT2B1,WSCD1
93	HEY1 Signaling Pathway	1.89E+00	5.10E-02	-0.707	ADORA1,BMP3,E2F6,MMP17,PIK3R4,PRKAG2,SMAD3,TGFB2
94	Adrenomedullin signaling pathway	1.78E+00	4.57E-02	-0.707	ADCY1,ADCY5,ITPR1,KCNQ3,PIK3R4,PLCH2,PLCL1,PRKAG2,RASD2
95	cAMP-mediated signaling	1.03E+00	3.42E-02	-0.707	ADCY1,ADCY5,ADORA1,CAMK1,DUSP6,PDE7A,PKIA,RGS14
96	Myelination Signaling Pathway	5.14E-01	2.48E-02	-0.707	BMP3,IGF1R,NTF3,PIK3R4,POU3F1,PRKAG2,RASD2,SMAD3
97	Neutrophil Extracellular Trap Signaling Pathway	4.00E-01	2.26E-02	-0.707	ARG2,ITPR1,PIK3R4,PLCH2,PLCL1,PRDX6,PRKCA,PRKCB
98	Breast Cancer Regulation by Stathmin1	2.66E+00	3.87E-02	-0.688	ADORA1,ARHGEF4,CAMK1,E2F6,GNB5,GPR12,GPR137C,GPR155,GPR27,MAS1,NPBWR1,NTSR2,PIK3R4,PPP1CC,PRKAG2,
99					PRKCA,PRKCB,RASD2,STMN1,TGFB2,Tubb2b,TUBB3
100	Neurovascular Coupling Signaling Pathway	2.85E+00	5.48E-02	-0.577	ADORA1,CACNA1C,CACNA2D1,CACNA2D3,CACNG3,GABRA1,GABRB3,GRIA3,ITPR1,PRDX6,PRKAG2,PTGS2
101	FAK Signaling	4.56E-01	2.20E-02	-0.471	ADORA1,ARPC2,ASAP1,EFNB3,ETS2,GPR12,GPR137C,GPR155,GPR27,IGF1R,ITGA7,KIT,MAS1,NPBWR1,NTSR2,
102					PIK3R4,RASD2,TGFB2

	A	B	C	D	E
103	Netrin-1 signaling	2.78E+00	1.14E-01	-0.447	ABLIM1,ABLIM2,ROBO1,SLIT1,SLIT2
104	G alpha (z) signalling events	2.61E+00	1.04E-01	-0.447	ADCY1,ADCY5,GNB5,PRKCA,PRKCB
105	14-3-3-mediated Signaling	2.47E+00	6.40E-02	-0.447	PIK3R4,PLCH2,PLCL1,PRKCA,PRKCB,RASD2,Tubb2b,TUBB3
106	Apelin Adipocyte Signaling Pathway	1.54E+00	5.75E-02	-0.447	ADCY1,ADCY5,MGST3,PRDX6,PRKAG2
107	Opioid Signalling	1.50E+00	5.62E-02	-0.447	ADCY1,ADCY5,GNB5,ITPR1,PRKCA
108	COPI-mediated anterograde transport	1.43E+00	5.38E-02	-0.447	CAPZB,DCTN6,NAPG,TMED9,TUBB3
109	Glioblastoma Multiforme Signaling	1.30E+00	4.14E-02	-0.447	E2F6,IGF1R,ITPR1,PIK3R4,PLCH2,PLCL1,RASD2
110	IL-15 Production	1.07E+00	4.24E-02	-0.447	EPHB3,IGF1R,KIT,PEAK1,TYRO3
111	Glycerophospholipid biosynthesis	1.03E+00	4.10E-02	-0.447	CDIPT,CDS1,CPNE7,GPD2,PCYT1B
112	Signaling by Rho Family GTPases	8.24E-01	3.04E-02	-0.447	ARHGEF4,ARPC2,CDH9,CLIP1,GNB5,ITGA7,PIK3R4,STMN1
113	Integrin Signaling	6.72E-01	2.96E-02	-0.447	ARPC2,ASAP1,ITGA7,PFN1,PIK3R4,RASD2
114	p70S6K Signaling	0.00E+00	1.53E-02	-0.447	PIK3R4,PLCH2,PLCL1,PRKCA,PRKCB,RASD2
115	GNRH Signaling	4.00E+00	6.88E-02	-0.378	ADCY1,ADCY5,CACNA1C,CACNA2D1,CACNA2D3,CACNG3,EGR1,GNB5,ITPR1,PRKAG2,PRKCA,PRKCB,RASD2
116	ERK/MAPK Signaling	2.00E+00	4.69E-02	-0.378	DUSP6,ETS2,ITGA7,KSR1,PIK3R4,PPP1CC,PRKAG2,PRKCA,PRKCB,RASD2
117	Response to elevated platelet cytosolic Ca2+	1.94E+00	5.65E-02	-0.378	PCDH7,PFN1,PHACTR2,PRKCA,PRKCB,TGFB2,VTI1B
118	ROBO SLIT Signaling Pathway	1.94E+00	5.65E-02	-0.378	ARPC2,CNTNAP2,PLXNA1,PRKAG2,ROBO1,SLIT1,SLIT2
119	Chaperone Mediated Autophagy Signaling Pathway	0.00E+00	1.84E-02	-0.378	HSPA1A,Hspa1b,HSPA2,IGF1R,MMP17,PEA15,PIK3R4,PRKAG2
120	Synaptic Long Term Depression	3.93E+00	6.77E-02	-0.302	CACNA1C,CACNA2D1,CACNA2D3,CACNG3,GRIA3,IGF1R,ITPR1,PLCH2,PLCL1,PRDX6,PRKCA,PRKCB,RASD2
121	Pancreatic Secretion Signaling Pathway	4.71E+00	6.78E-02	-0.258	ADCY1,ADCY5,ARHGEF4,ATP2B1,CADPS2,ITPR1,NAPG,PIK3R4,PLCH2,PLCL1,PPP1CC,PRKAG2,PRKCA,PRKCB,SLC4A7,VTI1B
122	Netrin Signaling	5.08E+00	8.14E-02	0	ABLIM1,ABLIM2,ARPC2,CACNA1C,CACNA2D1,CACNA2D3,CACNG3,ITPR1,NAPG,PIK3R4,PRKAG2,PRKCA,PRKCB,VTI1B
123	Nitric Oxide Signaling in the Cardiovascular System	4.69E+00	9.40E-02	0	ARG2,CACNA1C,CACNA2D1,CACNA2D3,CACNG3,ITPR1,PIK3R4,PRKAG2,PRKCA,PRKCB,SLC7A1
124	G Beta Gamma Signaling	4.30E+00	8.53E-02	0	ADCY1,CACNA1C,CACNA2D1,CACNA2D3,CACNG3,GNB5,ITPR1,PRKAG2,PRKCA,PRKCB,RASD2
125	eNOS Signaling	3.73E+00	7.38E-02	0	ADCY1,ADCY5,HSPA1A,Hspa1b,HSPA2,ITPR1,PIK3R4,PRKAG2,PRKCA,PRKCB,SLC7A1
126	Gap Junction Signaling	3.70E+00	5.36E-02	0	ADCY1,ADCY5,CCN3,CSNK1A1,GRIA3,ITPR1,KCNQ3,PIK3R4,PLCH2,PLCL1,PRKAG2,PRKCA,PRKCB,RASD2,SMAD3,Tubb2b,TUBB3
127	Senescence Pathway	2.74E+00	4.86E-02	0	BHLHE40,CACNA1C,CACNA2D1,CACNA2D3,CACNG3,DLAT,E2F6,ETS2,PARP1,PDK2,PIK3R4,RASD2,SMAD3,TGFB2
128	Dermatan Sulfate Biosynthesis (Late Stages)	2.69E+00	1.09E-01	0	CHST2,HS3ST4,HS6ST2,SULT2B1,WSCD1
129	Chondroitin Sulfate Biosynthesis (Late Stages)	2.57E+00	1.02E-01	0	CHST2,HS3ST4,HS6ST2,SULT2B1,WSCD1
130	GPCR-Mediated Integration of Enteroendocrine Signaling Exemplifi	2.47E+00	8.11E-02	0	ADCY1,ADCY5,ITPR1,PLCH2,PLCL1,PRKAG2
131	Activation of NMDA receptors and postsynaptic events	2.41E+00	7.89E-02	0	ADCY1,CAMK1,GRIA3,PPM1E,PRKAG2,TUBB3
132	Chondroitin Sulfate Biosynthesis	2.28E+00	8.77E-02	0	CHST2,HS3ST4,HS6ST2,SULT2B1,WSCD1
133	Dermatan Sulfate Biosynthesis	2.22E+00	8.47E-02	0	CHST2,HS3ST4,HS6ST2,SULT2B1,WSCD1
134	Apelin Cardiomyocyte Signaling Pathway	1.91E+00	6.19E-02	0	ITPR1,PIK3R4,PLCH2,PLCL1,PRKCA,PRKCB
135	Inositol phosphate metabolism	1.87E+00	8.51E-02	0	INPP5A,ITPKA,MTMR9,PLCH2
136	RAF/MAP kinase cascade	1.47E+00	3.85E-02	0	CNKSR2,DUSP5,DUSP6,KIT,KSR1,LAMTOR2,NCAM1,PEA15,PPP1CC,RASAL1
137	Phase II - Conjugation of compounds	1.41E+00	6.15E-02	0	GSTO1,MGST3,PAPSS1,SULT2B1
138	Macroautophagy	1.41E+00	6.15E-02	0	GABARAPL2,LAMTOR2,PIK3R4,PRKAG2
139	PKR-mediated signaling	1.38E+00	6.06E-02	0	EIF2S2,HSPA1A,HSPA2,TUBB3
140	Xenobiotic Metabolism General Signaling Pathway	1.36E+00	4.62E-02	0	GSTO1,MGST3,PIK3R4,PRKCA,PRKCB,RASD2
141	Insulin Receptor Signaling	1.25E+00	4.35E-02	0	ASIC2,INPP5F,PIK3R4,PPP1CC,PRKAG2,RASD2
142	Cholecystokinin/Gastrin-mediated Signaling	1.14E+00	4.42E-02	0	ITPR1,PRKCA,PRKCB,PTGS2,RASD2
143	Fc Epsilon RI Signaling	1.11E+00	4.35E-02	0	INPP5F,PIK3R4,PRKCA,PRKCB,RASD2
144	Cell junction organization	9.91E-01	4.44E-02	0	CADM1,CADM3,CDH9,NECTIN1
145	DNA Methylation and Transcriptional Repression Signaling	9.27E-01	4.21E-02	0	CDK17,CDK18,E2F6,MBD2
146	FXR/RXR Activation	9.11E-01	3.51E-02	0	G6PC3,GSTO1,MGST3,PRKAG2,PTGS2,TGFB2
147	Tumor Microenvironment Pathway	9.11E-01	3.51E-02	0	FGF11,MMP17,PIK3R4,PTGS2,RASD2,TGFB2
148	GPER1 signaling	8.80E-01	4.04E-02	0	ADCY1,ADCY5,GNB5,PIK3R4
149	Semaphorin Neuronal Repulsive Signaling Pathway	8.16E-01	3.50E-02	0	CSPG5,ITGA7,PIK3R4,PLXNA1,PRKAG2
150	Intra-Golgi and retrograde Golgi-to-ER traffic	7.60E-01	3.16E-02	0	CAPZB,DCTN6,MAN1A1,NAPG,TMED9,TUBB3
151	Chronic Myeloid Leukemia Signaling	7.51E-01	2.91E-02	0	E2F6,PIK3R4,PLCH2,PLCL1,PRKAG2,RASD2,SMAD3,TGFB2
152	Role of Tissue Factor in Cancer	6.92E-01	3.00E-02	0	EGR1,PIK3R4,PRKCA,PTGS2,RASD2,TGFB2
153	IL-8 Signaling	6.79E-01	2.97E-02	0	GNB5,PIK3R4,PRKCA,PRKCB,PTGS2,RASD2
154	Parkinson's Signaling Pathway	6.78E-01	2.78E-02	0	CACNA1C,CACNA2D1,CACNA2D3,CACNG3,GRIA3,ITPR1,TGFB2,TUBB3
155	Natural Killer Cell Signaling	6.37E-01	3.01E-02	0	HSPA1A,Hspa1b,HSPA2,PIK3R4,RASD2
156	Endocannabinoid Cancer Inhibition Pathway	5.19E-01	2.84E-02	0	ADCY1,ADCY5,PIK3R4,PRKAG2
157	IL-17 Signaling	4.12E-01	2.50E-02	0	PIK3R4,PTGS2,RASD2,TGFB2
158	Leukocyte Extravasation Signaling	2.97E-01	2.14E-02	0	MMP17,PIK3R4,PRKCA,PRKCB
159	Activin Inhibin Signaling Pathway	2.66E-01	2.04E-02	0	PIK3R4,PTGS2,SMAD3,TGFB2
160	Class A/1 (Rhodopsin-like receptors)	0.00E+00	1.37E-02	0	ADORA1,NPBWR1,NTSR2,PLPPR4
161	Dendritic Cell Maturation	0.00E+00	1.06E-02	0	FSCN1,PIK3R4,PLCH2,PLCL1
162	Phospholipase C Signaling	0.00E+00	1.37E-02	0	ADCY1,ADCY5,ARHGEF4,GNB5,ITGA7,ITPR1,MARCKS,PRKCA,PRKCB,RASD2
163	PI3K Signaling in B Lymphocytes	0.00E+00	1.25E-02	0	ITPR1,PLCH2,PLCL1,PRKCB,RASD2
164	Protein Ubiquitination Pathway	0.00E+00	1.89E-02	0	FBXW7,HSPA12A,HSPA1A,HSPA2,UBE2E2
165	IL-4 Signaling	0.00E+00	1.08E-02	0	NR3C1,PIK3R4,RASD2,TGFB2
166	Sirtuin Signaling Pathway	0.00E+00	1.81E-02	0	ARG2,DUSP6,GABARAPL2,PARP1,PGAM2
167	Systemic Lupus Erythematosus in B Cell Signaling Pathway	0.00E+00	1.17E-02	0	INPP5F,PIK3R4,PRKCA,PRKCB,RASD2,TGFB2
168	Synaptogenesis Signaling Pathway	6.55E+00	7.05E-02	0.229	ADCY1,ADCY5,ARPC2,CACNA2D1,CADM1,CDH9,CNTNAP2,CPLX2,EFNB3,EPHA7,EPHB3,GRIA3,ITPR1,MARCKS,NAPG,
169					NECTIN1,PIK3R4,PRKAG2,RASD2,SYT13,SYT17,VTI1B
170	Oxytocin Signaling Pathway	2.45E+00	4.69E-02	0.277	ATP2B1,CACNA1C,CACNA2D1,CACNA2D3,CACNG3,GNB5,ITPR1,PIK3R4,PRKAG2,PRKCA,PRKCB,PTGS2,RASD2
171	Sleep NREM Signaling Pathway	3.40E+00	7.96E-02	0.333	ADCY1,ADCY5,ADORA1,GABRA1,GABRB3,PARP1,PRKAG2,PRKCA,RASD2
172	Endothelin-1 Signaling	2.98E+00	6.01E-02	0.333	ADCY1,ADCY5,ITPR1,PIK3R4,PLCH2,PLCL1,PRDX6,PRKCA,PRKCB,PTGS2,RASD2
173	Role of Macrophages, Fibroblasts and Endothelial Cells in Rheumat	7.77E-01	2.88E-02	0.333	CSNK1A1,FRZB,IL16,PIK3R4,PLCH2,PLCL1,PRKCA,PRKCB,RASD2
174	Glycation Signaling Pathway	8.30E-01	3.17E-02	0.378	EGR1,PFKP,PIK3R4,PRKCA,PRKCB,RASD2,TGFB2
175	Sertoli Cell-Germ Cell Junction Signaling Pathway (Enhanced)	7.62E-01	3.03E-02	0.378	ARPC2,NECTIN1,PIK3R4,PRKAG2,RASD2,SMAD3,TGFB2
176	Renin-Angiotensin Signaling	2.58E+00	6.67E-02	0.447	ADCY1,ADCY5,ITPR1,PIK3R4,PRKAG2,PRKCA,PRKCB,RASD2
177	nNOS Signaling in Skeletal Muscle Cells	2.57E+00	1.02E-01	0.447	CACNA1C,CACNA2D1,CACNA2D3,CACNG3,ITPR1
178	Huntington's Disease Signaling	2.48E+00	4.73E-02	0.447	CPLX2,GNB5,HSPA1A,Hspa1b,HSPA2,IGF1R,ITPR1,NAPG,PIK3R4,PRKCA,PRKCB,RPH3A,VTI1B
179	GABA receptor activation	2.19E+00	8.33E-02	0.447	ADCY1,ADCY5,GABRA1,GABRB3,GNB5
180	Glioma Signaling	1.92E+00	5.60E-02	0.447	CAMK1,E2F6,IGF1R,PIK3R4,PRKCA,PRKCB,RASD2
181	fMLP Signaling in Neutrophils	1.82E+00	5.34E-02	0.447	ARPC2,GNB5,ITPR1,PIK3R4,PRKCA,PRKCB,RASD2
182	Sphingosine-1-phosphate Signaling	1.15E+00	4.46E-02	0.447	ADCY1,ADCY5,PIK3R4,PLCH2,PLCL1
183	Glycosaminoglycan metabolism	1.01E+00	4.03E-02	0.447	CHST2,CSPG5,HS3ST4,HS6ST2,PAPSS1
184	G alpha (s) signalling events	8.62E-01	3.62E-02	0.447	ADCY1,ADCY5,GNB5,GPR27,PDE7A
185	Transcriptional Regulatory Network in Embryonic Stem Cells	6.51E-01	3.05E-02	0.447	IGF1R,PIK3R4,RASD2,SMAD3,TGFB2
186	Autophagy	3.83E-01	2.34E-02	0.447	DAPK1,GABARAPL2,PIK3R4,PRKAG2,TGFB2
187	Histone Modification Signaling Pathway	0.00E+00	1.83E-02	0.447	HP1BP3,NSD3,PHF20,PRDM8,PRKCB
188	Endocannabinoid Neuronal Synapse Pathway	5.11E+00	8.78E-02	0.577	ADCY1,ADCY5,CACNA1C,CACNA2D1,CACNA2D3,CACNG3,GNB5,GRIA3,ITPR1,PLCH2,PLCL1,PRKAG2,PTGS2
189	Oxytocin in Brain Signaling Pathway	3.52E+00	6.52E-02	0.577	CACNA1C,CACNA2D1,CACNA2D3,CACNG3,CAMK1,GNB5,ITPR1,PIK3R4,PRDX6,PRKCA,PRKCB,PTGS2,RASD2
190	Neuropathic Pain Signaling in Dorsal Horn Neurons	4.55E+00	1.00E-01	0.632	CAMK1,GRIA3,ITPR1,KCNQ3,PIK3R4,PLCH2,PLCL1,PRKAG2,PRKCA,PRKCB
191	Thrombin Signaling	3.39E+00	5.96E-02	0.632	ADCY1,ADCY5,ARHGEF26,ARHGEF4,CAMK1,GNB5,ITPR1,PIK3R4,PLCH2,PLCL1,PRKCA,PRKCB,RASD2
192	Mitochondrial Division Signaling Pathway	2.80E+00	6.10E-02	0.632	CACNA1C,CACNA2D1,CACNA2D3,CACNG3,CAMK1,DUSP6,INF2,ITPR1,PRKAG2,RASD2
193	Autism Signaling Pathway	1.18E+00	3.41E-02	0.632	CACNA1C,CACNA2D1,CACNA2D3,CACNG3,CNTNAP2,IGF1R,PIK3R4,PRKAG2,RASD2,TGFB2
194	Cellular Effects of Sildenafil (Viagra)	1.95E+00	3.35E-02	0.655	ADCY1,ADCY5,ADORA1,CACNA1C,CACNA2D1,CACNA2D3,CACNG3,GNB5,GPR12,GPR137C,GPR155,GPR27,HCN1,KCNQ3,
195					MAS1,NPBWR1,NTSR2,PIK3R4,PPP1CC,PRKAG2,SMAD3,TGFB2
196	ID1 Signaling Pathway	1.36E+00	4.02E-02	0.707	BHLHE22,BHLHE40,EGR1,IGF1R,PIK3R4,RASD2,SMAD3,TGFB2
197	Hepatic Fibrosis Signaling Pathway	1.23E+00	3.22E-02	0.707	CACNA1C,CACNA2D1,CACNA2D3,CACNG3,CSNK1A1,ITGA7,PIK3R4,PRKAG2,PRKCA,PRKCB,RASD2,SMAD3,TGFB2
198	CLEAR Signaling Pathway	7.16E-01	2.85E-02	0.707	IGF1R,ITPR1,LAMTOR2,PRKAG2,PRKCA,PRKCB,RASD2,TGFB2
199	Protein Kinase A Signaling	3.90E+00	5.05E-02	0.728	ADCY1,ADCY5,DUSP5,DUSP6,GNB5,ITPR1,PDE7A,PLCH2,PLCL1,PPP1CC,PRKAG2,PRKCA,PRKCB,PTGS2,PTPN5,PTPRD,
200					PTPRO,SIRPA,SMAD3,TGFB2
201	Hepatitis B Chronic Liver Pathogenesis Signaling Pathway	9.46E-01	3.59E-02	0.816	EGR1,PIK3R4,PRKCA,PRKCB,RASD2,TGFB2
202	Ion channel transport	8.85E-01	3.45E-02	0.816	ASIC2,ASPH,ATP2B1,FKBP1B,FXYD7,TTYH1
203	Pulmonary Healing Signaling Pathway	7.32E-01	3.09E-02	0.816	MMP17,PRKAG2,PRKCA,PRKCB,RASD2,TGFB2
204	Beta-catenin independent WNT signaling	6.79E-01	2.97E-02	0.816	GNB5,ITPR1,PFN1,PIK3R4,PRKCA,PRKCB

	A	B	C	D	E
205	IL-12 Signaling and Production in Macrophages	6.53E-01	2.91E-02	0.816	ETS2,PIK3R4,PRKAG2,PRKCA,PRKCB,TGFB2
206	Glutamate binding, activation of AMPA receptors and synaptic plas	2.51E+00	1.29E-01	1	CACNG3,GRIA3,PRKCA,PRKCB
207	Type II Diabetes Mellitus Signaling	2.49E+00	5.92E-02	1	CACNA1C,CACNA2D1,CACNA2D3,CACNG3,ITPR1,PIK3R4,PRKAG2,PRKCA,PRKCB
208	HIF1Œ± Signaling	2.16E+00	4.95E-02	1	CAMK1,HSPA1A,Hspa1b,HSPA2,MMP17,PIK3R4,PRKCA,PRKCB,RASD2,TGFB2
209	Calcium Signaling	2.04E+00	4.76E-02	1	ASPH,ATP2B1,CACNA1C,CACNA2D1,CACNA2D3,CACNG3,CAMK1,GRIA3,ITPR1,PRKAG2
210	Human Embryonic Stem Cell Pluripotency	1.77E+00	4.55E-02	1	BMP3,NFYB,NTF3,PIK3R4,PRKCA,PRKCB,RASD2,SMAD3,TGFB2
211	PDGF Signaling	1.56E+00	5.81E-02	1	INPP5F,PIK3R4,PRKCA,PRKCB,RASD2
212	FGF Signaling	1.05E+00	4.65E-02	1	FGF11,ITPR1,PIK3R4,PRKCA
213	EPH-Ephrin signaling	9.78E-01	4.40E-02	1	ARPC2,EFNB3,EPHA7,EPHB3
214	Production of Nitric Oxide and Reactive Oxygen Species in Macroph	8.05E-01	3.26E-02	1	ARG2,PIK3R4,PPP1CC,PRKCA,PRKCB,SIRPA
215	Virus Entry via Endocytic Pathways	7.26E-01	3.51E-02	1	PIK3R4,PRKCA,PRKCB,RASD2
216	Regulation of lipid metabolism by PPARalpha	7.17E-01	3.48E-02	1	ARNT2,FHL2,MED10,NFYB
217	GP6 Signaling Pathway	6.32E-01	3.20E-02	1	ITPR1,PIK3R4,PRKCA,PRKCB
218	GŒ±q Signaling	6.31E-01	2.99E-02	1	GNB5,ITPR1,PIK3R4,PRKCA,PRKCB
219	RHOGDI Signaling	6.00E-01	2.79E-02	1	ARHGEF4,ARPC2,CDH9,GNB5,ITGA7,PRKCA
220	mTOR Signaling	4.21E-01	2.44E-02	1	PIK3R4,PRKAG2,PRKCA,PRKCB,RASD2
221	Osteoarthritis Pathway	3.37E-01	2.21E-02	1	FRZB,ITGA7,PRKAG2,PTGS2,SMAD3
222	CDX Gastrointestinal Cancer Signaling Pathway	2.76E-01	2.07E-02	1	BMP3,PIK3R4,PTGS2,TGFB2
223	Role of Osteoclasts in Rheumatoid Arthritis Signaling Pathway	0.00E+00	1.32E-02	1	FRZB,MMP17,PIK3R4,RASD2
224	Neddylation	0.00E+00	1.65E-02	1	COPS4,FBXL5,FBXW7,LMO7
225	B Cell Receptor Signaling	0.00E+00	1.13E-02	1	EGR1,INPP5F,PIK3R4,PRKCB,RASD2
226	T Cell Receptor Signaling	0.00E+00	9.83E-03	1	DUSP5,DUSP6,PIK3R4,RASD2
227	Apelin Endothelial Signaling Pathway	2.69E+00	6.34E-02	1.134	ADCY1,ADCY5,GNB5,PIK3R4,PRKAG2,PRKCA,PRKCB,RASD2,SMAD3
228	HER-2 Signaling in Breast Cancer	1.10E+00	3.54E-02	1.134	ETS2,MTCL1,PIK3R4,PRKCA,PRKCB,PTGS2,RASD2,SMAD3
229	Nuclear Cytoskeleton Signaling Pathway	9.12E-01	3.33E-02	1.134	CAPZB,CDH9,DCTN6,EGR1,ITGA7,MTCL1,TUBB3
230	Pulmonary Fibrosis Idiopathic Signaling Pathway	3.50E-01	2.18E-02	1.134	EGR1,EPHB3,MMP17,PIK3R4,RASD2,SMAD3,TGFB2
231	Class I MHC mediated antigen processing and presentation	0.00E+00	1.91E-02	1.134	FBXL5,FBXW7,LMO7,MYLIP,PIK3R4,RNF182,UBE2E2
232	Opioid Signaling Pathway	3.36E+00	5.40E-02	1.155	ADCY1,ADCY5,CACNA1C,CACNA2D1,CACNA2D3,CACNG3,CAMK1,GNB5,ITPR1,NPBWR1,PRKAG2,PRKCA,PRKCB,RASD2,RGS14
233	Cardiac conduction	2.45E+00	6.35E-02	1.342	ASPH,ATP2B1,CACNA1C,FGF11,FKBP1B,FXYD7,ITPR1,KCNK1
234	GŒ±i Signaling	1.65E+00	4.93E-02	1.342	ADCY1,ADCY5,ADORA1,GNB5,PRKAG2,RASD2,RGS14
235	Post-translational protein phosphorylation	1.27E+00	4.85E-02	1.342	GOLM1,IGFBP4,SCG2,TMEM132A,WFS1
236	Regulation of Insulin-like Growth Factor (IGF) transport and uptake	1.09E+00	4.27E-02	1.342	GOLM1,IGFBP4,SCG2,TMEM132A,WFS1
237	DHCR24 Signaling Pathway	9.10E-01	3.76E-02	1.342	GGA3,PIK3R4,PRKCA,PRKCB,RASD2
238	Regulation of the Epithelial Mesenchymal Transition by Growth Fac	7.46E-01	3.12E-02	1.342	EGR1,FGF11,PIK3R4,RASD2,SMAD3,TGFB2
239	MicroRNA Biogenesis Signaling Pathway	5.49E-01	2.78E-02	1.342	MTCL1,PIK3R4,RASD2,SMAD3,TGFB2
240	Mitochondrial Dysfunction	1.79E+00	3.88E-02	1.387	ARG2,CACNA1C,CACNA2D1,CACNA2D3,CACNG3,DLAT,FBXW7,GPD2,ITPR1,MGST3,PIK3R4,PRDX6,PRKAG2
241	WNT/SHH Axonal Guidance Signaling Pathway	2.00E+00	5.33E-02	1.414	ADCY1,ADCY5,ITPR1,PIK3R4,PLXNA1,PRKAG2,PRKCA,SHISA2
242	Erythropoietin Signaling Pathway	9.37E-01	3.57E-02	1.633	ITPR1,PIK3R4,PRKCA,PRKCB,RASD2,TGFB2
243	Sertoli Cell-Sertoli Cell Junction Signaling	7.24E-01	2.95E-02	1.633	CDH9,NECTIN1,PIK3R4,RAB8B,RASD2,Tubb2b,TUBB3
244	Cohesin Chromatin Regulation Pathway	6.23E-01	2.84E-02	1.633	HP1BP3,MED10,PCDH1,PCDH17,PCDH19,PCDH7
245	GABAergic Receptor Signaling Pathway (Enhanced)	4.85E+00	8.96E-02	1.732	ADCY1,ADCY5,CACNA1C,CACNA2D1,CACNA2D3,CACNG3,CNTNAP2,GABRA1,GABRB3,GNB5,ITPR1,PRKAG2
246	CXCR4 Signaling	2.29E+00	5.52E-02	1.89	ADCY1,ADCY5,EGR1,GNB5,ITPR1,PIK3R4,PRKCA,PRKCB,RASD2
247	Potassium Channels	4.44E+00	9.71E-02	1.897	GNB5,HCN1,KCNAB1,KCNF1,KCNG2,KCNH3,KCNH7,KCNK1,KCNQ3,KCNV1
248	NAP1L1 Transcription Regulation Signaling Pathway	1.17E+00	5.13E-02	2	DLAT,PIK3R4,PRDM8,TUBB3
249	Protein folding	9.78E-01	4.40E-02	2	FBXL5,FBXW7,GNB5,TUBB3
250	Ephrin A Signaling	5.45E-01	2.92E-02	2	EPHA7,PIK3R4,PRKAG2,TGFB2
251	L1CAM interactions	2.15E+00	6.19E-02	2.646	ALCAM,CHL1,CNTN1,GAP43,KCNQ3,NCAM1,TUBB3
252	Axonal Guidance Signaling	5.23E+00	5.27E-02	NaN	ABLIM1,ABLIM2,ARPC2,BMP3,EFNB3,EPHA7,EPHB3,GNB5,ITGA7,MMP17,NTF3,PFN1,PIK3R4,PLCH2,PLCL1,PLXNA1,PRKAG2,
253					PRKCA,PRKCB,RASD2,ROBO1,SLIT1,SLIT2,SRGAP3,Tubb2b,TUBB3
254	Circadian Rhythm Signaling	5.19E+00	6.74E-02	NaN	ADCY1,ADCY5,BHLHE40,CACNA1C,CACNA2D1,CACNA2D3,CACNG3,DBP,GNB5,GRIA3,ITPR1,NR3C1,PLCH2,PLCL1,PRKAG2,
255					PRKCA,PRKCB,RASD2
256	GABA Receptor Signaling	4.24E+00	8.40E-02	NaN	ADCY1,ADCY5,CACNA1C,CACNA2D1,CACNA2D3,CACNG3,GABRA1,GABRB3,GNB5,ITPR1,KCNQ3
257	Œ±-Adrenergic Signaling	2.87E+00	7.41E-02	NaN	ADCY1,ADCY5,GNB5,ITPR1,PRKAG2,PRKCA,PRKCB,RASD2
258	Leptin Signaling in Obesity	2.44E+00	8.00E-02	NaN	ADCY1,ADCY5,PIK3R4,PLCH2,PLCL1,PRKAG2
259	1D-myo-inositol Hexakisphosphate Biosynthesis II (Mammalian)	2.38E+00	1.76E-01	NaN	INPP5A,INPP5F,ITPKA
260	D-myo-inositol (1,3,4)-trisphosphate Biosynthesis	2.38E+00	1.76E-01	NaN	INPP5A,INPP5F,ITPKA
261	Xenobiotic Metabolism Signaling	2.19E+00	4.55E-02	NaN	CAMK1,CHST2,GSTO1,HS3ST4,HS6ST2,MGST3,PIK3R4,PRKCA,PRKCB,RASD2,SULT2B1,WSCD1
262	Amyotrophic Lateral Sclerosis Signaling	2.17E+00	6.25E-02	NaN	CACNA1C,CACNA2D1,CACNA2D3,CACNG3,GRIA3,GRIK4,PIK3R4
263	Arginine Degradation VI (Arginase 2 Pathway)	2.12E+00	2.86E-01	NaN	ARG2,PYCR2
264	Superpathway of D-myo-inositol (1,4,5)-trisphosphate Metabolism	2.05E+00	1.36E-01	NaN	INPP5A,INPP5F,ITPKA
265	RAF-independent MAPK1/3 activation	2.00E+00	1.30E-01	NaN	DUSP5,DUSP6,PEA15
266	RHO GTPases Activate NADPH Oxidases	1.95E+00	1.25E-01	NaN	PIK3R4,PRKCA,PRKCB
267	HGF Signaling	1.83E+00	5.38E-02	NaN	ETS2,ITGA7,PIK3R4,PRKCA,PRKCB,PTGS2,RASD2
268	IGF-1 Signaling	1.79E+00	5.83E-02	NaN	CCN3,IGF1R,IGFBP4,PIK3R4,PRKAG2,RASD2
269	Activation of kainate receptors upon glutamate binding	1.68E+00	1.00E-01	NaN	GNB5,GRIK4,NCALD
270	Presynaptic depolarization and calcium channel opening	1.65E+00	1.67E-01	NaN	CACNA2D1,CACNA2D3
271	Glutathione-mediated Detoxification	1.61E+00	9.38E-02	NaN	Gsta4,GSTO1,MGST3
272	Triglyceride metabolism	1.57E+00	9.09E-02	NaN	FABP3,GPD2,PPP1CC
273	Transcriptional Regulation by NPAS4	1.54E+00	8.82E-02	NaN	ARNT2,NR3C1,PLK2
274	LGI-ADAM interactions	1.52E+00	1.43E-01	NaN	CACNG3,LGI1
275	Thrombopoietin Signaling	1.45E+00	6.35E-02	NaN	PIK3R4,PRKCA,PRKCB,RASD2
276	Remodeling of Epithelial Adherens Junctions	1.45E+00	6.35E-02	NaN	ARPC2,CLIP1,Tubb2b,TUBB3
277	Prolactin Signaling	1.42E+00	5.32E-02	NaN	NR3C1,PIK3R4,PRKCA,PRKCB,RASD2
278	Non-Small Cell Lung Cancer Signaling	1.42E+00	5.32E-02	NaN	E2F6,ITPR1,PIK3R4,PRKCA,RASD2
279	D-myo-inositol (1,4,5)-trisphosphate Degradation	1.41E+00	1.25E-01	NaN	INPP5A,INPP5F
280	Endocannabinoid Developing Neuron Pathway	1.41E+00	4.76E-02	NaN	ADCY1,ADCY5,GNB5,PIK3R4,PRKAG2,RASD2
281	Alanine metabolism	1.41E+00	5.00E-01	NaN	GPT2
282	Alanine Degradation III	1.41E+00	5.00E-01	NaN	GPT2
283	Alanine Biosynthesis II	1.41E+00	5.00E-01	NaN	GPT2
284	Sulfate Activation for Sulfonation	1.41E+00	5.00E-01	NaN	PAPSS1
285	Glycerol-3-phosphate Shuttle	1.41E+00	5.00E-01	NaN	GPD2
286	Signaling by Retinoic Acid	1.39E+00	7.69E-02	NaN	CYP26B1,DLAT,PDK2
287	VEGF Signaling	1.38E+00	5.21E-02	NaN	EIF2S2,PIK3R4,PRKCA,PRKCB,RASD2
288	ERBB4 Signaling	1.36E+00	5.97E-02	NaN	PIK3R4,PRKCA,PRKCB,RASD2
289	Cell Cycle: G1/S Checkpoint Regulation	1.36E+00	5.97E-02	NaN	E2F6,FBXL5,SMAD3,TGFB2
290	Glutamate Receptor Signaling	1.36E+00	5.97E-02	NaN	GNB5,GRIA3,GRIK4,HOMER3
291	CCR3 Signaling in Eosinophils	1.34E+00	4.58E-02	NaN	GNB5,ITPR1,PIK3R4,PRKCA,PRKCB,RASD2
292	Growth Hormone Signaling	1.33E+00	5.80E-02	NaN	IGF1R,PIK3R4,PRKCA,PRKCB
293	Vasopressin regulates renal water homeostasis via Aquaporins	1.30E+00	7.14E-02	NaN	ADCY1,ADCY5,GNB5
294	TBC/RABGAPs	1.28E+00	6.98E-02	NaN	GABARAPL2,GGA3,RAB8B
295	Signaling by Activin	1.27E+00	1.05E-01	NaN	DRAP1,SMAD3
296	Maturity Onset Diabetes of Young (MODY) Signaling	1.25E+00	5.48E-02	NaN	CACNA1C,CACNA2D1,CACNA2D3,CACNG3
297	Spermine Biosynthesis	1.24E+00	3.33E-01	NaN	SMS
298	Ascorbate Recycling (Cytosolic)	1.24E+00	3.33E-01	NaN	GSTO1
299	Signaling by NODAL	1.23E+00	1.00E-01	NaN	DRAP1,SMAD3
300	Receptor-type tyrosine-protein phosphatases	1.23E+00	1.00E-01	NaN	PPFIA2,PTPRD
301	Chemokine Signaling	1.22E+00	5.33E-02	NaN	CAMK1,PRKCA,PRKCB,RASD2
302	Macropinocytosis Signaling	1.20E+00	5.26E-02	NaN	PIK3R4,PRKCA,PRKCB,RASD2
303	Synaptic adhesion-like molecules	1.20E+00	9.52E-02	NaN	GRIA3,PTPRD
304	NF-Œ∫B Activation by Viruses	1.18E+00	5.19E-02	NaN	PIK3R4,PRKCA,PRKCB,RASD2

	A	B	C	D	E
305	Dopamine Receptor Signaling	1.18E+00	5.19E-02	NaN	ADCY1,ADCY5,PPP1CC,PRKAG2
306	Role of MAPK Signaling in the Pathogenesis of Influenza	1.17E+00	5.13E-02	NaN	PRDX6,PRKCA,PTGS2,RASD2
307	Vitamin-C Transport	1.16E+00	9.09E-02	NaN	GSTO1,LRRC8D
308	Bladder Cancer Signaling	1.14E+00	4.42E-02	NaN	DAPK1,E2F6,FGF11,MMP17,RASD2
309	IL-3 Signaling	1.13E+00	5.00E-02	NaN	PIK3R4,PRKCA,PRKCB,RASD2
310	Apelin Cardiac Fibroblast Signaling Pathway	1.13E+00	8.70E-02	NaN	PRKAG2,TGFB2
311	Proline Biosynthesis I	1.12E+00	2.50E-01	NaN	PYCR2
312	Adenine and Adenosine Salvage I	1.12E+00	2.50E-01	NaN	APRT
313	Rapoport-Luebering Glycolytic Shunt	1.12E+00	2.50E-01	NaN	PGAM2
314	Immunogenic Cell Death Signaling Pathway	1.10E+00	4.88E-02	NaN	DAPK1,HSPA1A,Hspa1b,HSPA2
315	UVC-Induced MAPK Signaling	1.10E+00	5.88E-02	NaN	PRKCA,PRKCB,RASD2
316	RHO GTPases activate IQGAPs	1.09E+00	8.33E-02	NaN	CLIP1,TUBB3
317	VEGF Family Ligand-Receptor Interactions	1.09E+00	4.82E-02	NaN	PIK3R4,PRKCA,PRKCB,RASD2
318	Neuregulin Signaling	1.09E+00	4.27E-02	NaN	ERRFI1,ITGA7,PRKCA,PRKCB,RASD2
319	UVB-Induced MAPK Signaling	1.08E+00	5.77E-02	NaN	PIK3R4,PRKCA,PRKCB
320	Signaling by NTRK2 (TRKB)	1.06E+00	8.00E-02	NaN	DOCK3,NTF3
321	Glutathione Redox Reactions I	1.06E+00	8.00E-02	NaN	MGST3,PRDX6
322	LPS-stimulated MAPK Signaling	1.06E+00	4.71E-02	NaN	PIK3R4,PRKCA,PRKCB,RASD2
323	Signaling by TGF-beta Receptor Complex	1.04E+00	5.56E-02	NaN	PPP1CC,SMAD3,TGFB2
324	BMAL1:CLOCK,NPAS2 activates circadian gene expression	1.03E+00	7.69E-02	NaN	BHLHE40,DBP
325	Signaling by NTRK3 (TRKC)	1.03E+00	7.69E-02	NaN	NTF3,PTPRO
326	D-myo-inositol (1,4,5)-Trisphosphate Biosynthesis	1.03E+00	7.69E-02	NaN	CDIPT,PLCH2
327	Glycolysis I	1.03E+00	7.69E-02	NaN	PFKP,PGAM2
328	EGF Signaling	1.03E+00	5.45E-02	NaN	ITPR1,PIK3R4,PRKCA
329	Proline Biosynthesis II (from Arginine)	1.02E+00	2.00E-01	NaN	PYCR2
330	Myo-inositol Biosynthesis	1.02E+00	2.00E-01	NaN	INPP5F
331	Arginine Degradation I (Arginase Pathway)	1.02E+00	2.00E-01	NaN	ARG2
332	LPS/IL-1 Mediated Inhibition of RXR Function	1.02E+00	3.39E-02	NaN	CHST2,FABP3,GSTO1,HS3ST4,HS6ST2,MGST3,SULT2B1,WSCD1
333	Pancreatic Adenocarcinoma Signaling	1.02E+00	4.07E-02	NaN	E2F6,PIK3R4,PTGS2,SMAD3,TGFB2
334	Germ Cell-Sertoli Cell Junction Signaling	9.93E-01	3.70E-02	NaN	PIK3R4,RAB8B,RASD2,TGFB2,Tubb2b,TUBB3
335	Ephrin Receptor Signaling	9.76E-01	3.47E-02	NaN	ARPC2,EFNB3,EPHA7,EPHB3,GNB5,ITGA7,RASD2
336	Visual phototransduction	9.73E-01	5.17E-02	NaN	GNB5,NMT2,PRKCA
337	Signaling by ERBB4	9.57E-01	5.08E-02	NaN	GABRA1,GABRB3,STMN1
338	Energy dependent regulation of mTOR by LKB1-AMPK	9.54E-01	6.90E-02	NaN	LAMTOR2,PRKAG2
339	SUMOylation of intracellular receptors	9.54E-01	6.90E-02	NaN	NR3C1,NR3C2
340	FOXO-mediated transcription of oxidative stress, metabolic and ne	9.54E-01	6.90E-02	NaN	NR3C1,SMAD3
341	ERBB Signaling	9.52E-01	4.30E-02	NaN	PIK3R4,PRKCA,PRKCB,RASD2
342	Lipid particle organization	9.48E-01	1.67E-01	NaN	CIDEA
343	Glycerol Degradation I	9.48E-01	1.67E-01	NaN	GPD2
344	Acetyl-CoA Biosynthesis I (Pyruvate Dehydrogenase Complex)	9.48E-01	1.67E-01	NaN	DLAT
345	Regulation of CDH11 Expression and Function	9.30E-01	6.67E-02	NaN	BHLHE22,PRDM8
346	IL-1 Signaling	9.15E-01	4.17E-02	NaN	ADCY1,ADCY5,GNB5,PRKAG2
347	Phospholipases	8.94E-01	4.76E-02	NaN	PLCH2,PLCL1,PRDX6
348	Wax and plasmalogen biosynthesis	8.86E-01	1.43E-01	NaN	FAR1
349	Aryl hydrocarbon receptor signalling	8.86E-01	1.43E-01	NaN	ARNT2
350	Arsenate Detoxification I (Glutaredoxin)	8.86E-01	1.43E-01	NaN	GSTO1
351	Urea Cycle	8.86E-01	1.43E-01	NaN	ARG2
352	Phosphatidylcholine Biosynthesis I	8.86E-01	1.43E-01	NaN	PCYT1B
353	Sialic acid metabolism	8.84E-01	6.25E-02	NaN	ST6GALNAC5,ST8SIA5
354	Pyridoxal 5'-phosphate Salvage Pathway	8.64E-01	4.62E-02	NaN	CDK18,CSNK1A1,DAPK1
355	WNT/Ca+ pathway	8.50E-01	4.55E-02	NaN	PLCH2,PLCL1,PRKCA
356	GM-CSF Signaling	8.23E-01	4.41E-02	NaN	PIK3R4,PRKCB,RASD2
357	Gene and protein expression by JAK-STAT signaling after IL-12 stim	8.21E-01	5.71E-02	NaN	GSTO1,HNRNPDL
358	MIF-mediated Glucocorticoid Regulation	8.21E-01	5.71E-02	NaN	NR3C1,PTGS2
359	Sensory processing of sound by inner hair cells of the cochlea	8.09E-01	4.35E-02	NaN	ATP2B1,CABP1,CAPZB
360	NRF2-mediated Oxidative Stress Response	8.02E-01	3.11E-02	NaN	FKBP5,GSTO1,MGST3,PIK3R4,PRKCA,PRKCB,RASD2
361	Caspase activation via Dependence Receptors in the absence of liga	7.85E-01	1.11E-01	NaN	DAPK1
362	Urea cycle	7.85E-01	1.11E-01	NaN	ARG2
363	Lipophagy	7.85E-01	1.11E-01	NaN	PRKAG2
364	Cellular response to mitochondrial stress	7.85E-01	1.11E-01	NaN	EIF2S2
365	Antioxidant Action of Vitamin C	7.83E-01	3.70E-02	NaN	GSTO1,PLCH2,PLCL1,PRDX6
366	Hedgehog 'off' state	7.73E-01	3.67E-02	NaN	ADCY1,ADCY5,CSNK1A1,TUBB3
367	Role of MAPK Signaling in Promoting the Pathogenesis of Influenza	7.73E-01	3.67E-02	NaN	PRDX6,PRKCA,PTGS2,RASD2
368	Resolution of Abasic Sites (AP sites)	7.64E-01	5.26E-02	NaN	PARP1,POLE4
369	Carboxyterminal post-translational modifications of tubulin	7.64E-01	5.26E-02	NaN	TTLL1,TUBB3
370	Ephrin B Signaling	7.59E-01	4.11E-02	NaN	EFNB3,EPHB3,GNB5
371	NGF-stimulated transcription	7.47E-01	5.13E-02	NaN	EGR1,EGR3
372	VDR/RXR Activation	7.47E-01	4.05E-02	NaN	PRKCA,PRKCB,TGFB2
373	ATF6 (ATF6-alpha) activates chaperone genes	7.43E-01	1.00E-01	NaN	NFYB
374	Formation of the posterior neural plate	7.43E-01	1.00E-01	NaN	POU3F1
375	Protein lipoylation	7.43E-01	1.00E-01	NaN	DLAT
376	Oleate Biosynthesis II (Animals)	7.43E-01	1.00E-01	NaN	Scd2
377	Calcium Transport I	7.43E-01	1.00E-01	NaN	ATP2B1
378	Citrulline Biosynthesis	7.43E-01	1.00E-01	NaN	ARG2
379	Regulation of the Epithelial-Mesenchymal Transition Pathway	7.32E-01	3.09E-02	NaN	EGR1,FGF11,PIK3R4,RASD2,SMAD3,TGFB2
380	Meiotic synapsis	7.30E-01	5.00E-02	NaN	HSPA2,SYCE2
381	Bile acid and bile salt metabolism	7.30E-01	5.00E-02	NaN	OSBPL6,STARD5
382	Mechanisms of Viral Exit from Host Cells	7.30E-01	5.00E-02	NaN	PRKCA,PRKCB
383	GDNF Family Ligand-Receptor Interactions	7.23E-01	3.95E-02	NaN	ITPR1,PIK3R4,RASD2
384	Ovarian Cancer Signaling	7.17E-01	3.23E-02	NaN	E2F6,PIK3R4,PRKAG2,PTGS2,RASD2
385	PAK Signaling	7.17E-01	3.48E-02	NaN	EPHB3,ITGA7,PIK3R4,RASD2
386	Antiproliferative Role of Somatostatin Receptor 2	7.12E-01	3.90E-02	NaN	GNB5,PIK3R4,RASD2
387	Thyroid Cancer Signaling	7.12E-01	3.90E-02	NaN	NTF3,PIK3R4,RASD2
388	Formation of the anterior neural plate	7.06E-01	9.09E-02	NaN	POU3F1
389	Prostanoid Biosynthesis	7.06E-01	9.09E-02	NaN	PTGS2
390	Neurotrophin/TRK Signaling	7.01E-01	3.85E-02	NaN	NTF3,PIK3R4,RASD2
391	Signaling by SCF-KIT	6.98E-01	4.76E-02	NaN	KIT,PRKCA
392	G alpha (12/13) signalling events	6.90E-01	3.80E-02	NaN	ARHGEF26,ARHGEF4,GNB5
393	Post-translational modification: synthesis of GPI-anchored proteins	6.79E-01	3.75E-02	NaN	LY6E,MDGA1,NEGR1
394	Estrogen-Dependent Breast Cancer Signaling	6.79E-01	3.75E-02	NaN	IGF1R,PIK3R4,RASD2
395	RHOA Signaling	6.73E-01	3.33E-02	NaN	ARPC2,IGF1R,PFN1,PLXNA1
396	Signal regulatory protein family interactions	6.73E-01	8.33E-02	NaN	SIRPA
397	HDR through MMEJ (alt-NHEJ)	6.73E-01	8.33E-02	NaN	PARP1
398	PI Metabolism	6.69E-01	3.70E-02	NaN	INPP5F,MTMR9,PIK3R4
399	Glucose metabolism	6.69E-01	3.70E-02	NaN	G6PC3,PFKP,PGAM2
400	BAG2 Signaling Pathway	6.69E-01	3.70E-02	NaN	HSPA1A,Hspa1b,HSPA2
401	RHO GTPases Activate WASPs and WAVEs	6.53E-01	4.44E-02	NaN	ARPC2,WIPF3
402	Apelin Pancreas Signaling Pathway	6.53E-01	4.44E-02	NaN	PIK3R4,PRKAG2
403	Glucocorticoid Receptor Signaling	6.49E-01	2.51E-02	NaN	ARG2,FKBP5,HSPA1A,Hspa1b,HSPA2,NR3C1,NR3C2,PIK3R4,PRKAG2,PTGS2,RASD2,SMAD3,TGFB2
404	Cyclins and Cell Cycle Regulation	6.48E-01	3.61E-02	NaN	E2F6,FBXL5,TGFB2
405	Glutamate and glutamine metabolism	6.42E-01	7.69E-02	NaN	PYCR2

	A	B	C	D	E
406	Nephron development	6.42E-01	7.69E-02	NaN	POU3F3
407	GŒ±s Signaling	6.40E-01	3.23E-02	NaN	ADCY1,ADCY5,GNB5,PRKAG2
408	Role of OCT4 in Mammalian Embryonic Stem Cell Pluripotency	6.39E-01	4.35E-02	NaN	ETS2,PARP1
409	PEDF Signaling	6.38E-01	3.57E-02	NaN	DOCK3,PIK3R4,RASD2
410	Platelet homeostasis	6.28E-01	3.53E-02	NaN	ATP2B1,GNB5,ITPR1
411	XBP1(S) activates chaperone genes	6.25E-01	4.26E-02	NaN	EXTL2,WFS1
412	Carnitine metabolism	6.14E-01	7.14E-02	NaN	PRKAG2
413	Synthesis of Prostaglandins (PG) and Thromboxanes (TX)	6.14E-01	7.14E-02	NaN	PTGS2
414	Regulation of TP53 Activity through Association with Co-factors	6.14E-01	7.14E-02	NaN	PHF20
415	Formation of axial mesoderm	6.14E-01	7.14E-02	NaN	SMAD3
416	DNA Double-Strand Break Repair by Non-Homologous End Joining	6.14E-01	7.14E-02	NaN	PARP1
417	Choline Biosynthesis III	6.14E-01	7.14E-02	NaN	PCYT1B
418	Leukotriene Biosynthesis	6.14E-01	7.14E-02	NaN	MGST3
419	Pyruvate metabolism	6.12E-01	4.17E-02	NaN	DLAT,PDK2
420	nNOS Signaling in Neurons	6.12E-01	4.17E-02	NaN	PRKCA,PRKCB
421	PFKFB4 Signaling Pathway	6.12E-01	4.17E-02	NaN	PRKAG2,TGFB2
422	BMP signaling pathway	6.10E-01	3.45E-02	NaN	BMP3,PRKAG2,RASD2
423	PXR/RXR Activation	5.99E-01	4.08E-02	NaN	NR3C1,PRKAG2
424	Melanoma Signaling	5.99E-01	4.08E-02	NaN	PIK3R4,RASD2
425	Apelin Muscle Signaling Pathway	5.99E-01	4.08E-02	NaN	GNB5,PRKAG2
426	Fcgamma receptor (FCGR) dependent phagocytosis	5.91E-01	3.37E-02	NaN	ARPC2,ITPR1,WIPF3
427	Unfolded protein response	5.91E-01	3.37E-02	NaN	HSPA1A,Hspa1b,HSPA2
428	Response of EIF2AK1 (HRI) to heme deficiency	5.88E-01	6.67E-02	NaN	EIF2S2
429	Formation of definitive endoderm	5.88E-01	6.67E-02	NaN	SMAD3
430	Signaling by TGFBR3	5.87E-01	4.00E-02	NaN	SMAD3,TGFB2
431	Amyloid Processing	5.87E-01	4.00E-02	NaN	CSNK1A1,PRKAG2
432	Tight Junction Signaling	5.85E-01	2.87E-02	NaN	NAPG,NECTIN1,PRKAG2,TGFB2,VTI1B
433	Ceramide Signaling	5.82E-01	3.33E-02	NaN	KSR1,PIK3R4,RASD2
434	Actin Nucleation by ARP-WASP Complex	5.82E-01	3.33E-02	NaN	ARPC2,ITGA7,RASD2
435	Transcriptional activity of SMAD2/SMAD3:SMAD4 heterotrimer	5.75E-01	3.92E-02	NaN	PARP1,SMAD3
436	Role of IL-17A in Arthritis	5.75E-01	3.92E-02	NaN	PIK3R4,PTGS2
437	Phototransduction Pathway	5.75E-01	3.92E-02	NaN	GNB5,PRKAG2
438	FAT10 Cancer Signaling Pathway	5.75E-01	3.92E-02	NaN	SMAD3,TGFB2
439	CCR5 Signaling in Macrophages	5.75E-01	2.59E-02	NaN	CACNA1C,CACNA2D1,CACNA2D3,CACNG3,GNB5,ITPR1,PRKCA,PRKCB
440	FcŒ≥ Receptor-mediated Phagocytosis in Macrophages and Monoc	5.74E-01	3.30E-02	NaN	ARPC2,PRKCA,PRKCB
441	Acute Myeloid Leukemia Signaling	5.74E-01	3.30E-02	NaN	KIT,PIK3R4,RASD2
442	Iron homeostasis signaling pathway	5.66E-01	2.99E-02	NaN	BMP3,FBXL5,SLC39A14,SMAD3
443	Salvage Pathways of Pyrimidine Ribonucleotides	5.65E-01	3.26E-02	NaN	CDK18,CSNK1A1,DAPK1
444	FOXO-mediated transcription of cell death genes	5.64E-01	6.25E-02	NaN	NFYB
445	Granzyme B Signaling	5.64E-01	6.25E-02	NaN	PARP1
446	Superpathway of Citrulline Metabolism	5.64E-01	6.25E-02	NaN	ARG2
447	RAC Signaling	5.45E-01	2.92E-02	NaN	ARPC2,ITGA7,PIK3R4,RASD2
448	Signaling by Type 1 Insulin-like Growth Factor 1 Receptor (IGF1R)	5.42E-01	5.88E-02	NaN	IGF1R
449	Transcriptional regulation of pluripotent stem cells	5.42E-01	5.88E-02	NaN	FOXP1
450	Gastrin-CREB signalling pathway via PKC and MAPK	5.42E-01	5.88E-02	NaN	PRKCA
451	FOXO-mediated transcription of cell cycle genes	5.42E-01	5.88E-02	NaN	SMAD3
452	Germ layer formation at gastrulation	5.42E-01	5.88E-02	NaN	SMAD3
453	Cell Cycle Control of Chromosomal Replication	5.40E-01	3.70E-02	NaN	CDK17,CDK18
454	Cellular response to heat stress	5.40E-01	3.16E-02	NaN	HSPA12A,HSPA1A,HSPA2
455	PD-1, PD-L1 cancer immunotherapy pathway	5.40E-01	3.16E-02	NaN	PIK3R4,SMAD3,TGFB2
456	Triacylglycerol Biosynthesis	5.29E-01	3.64E-02	NaN	PLPP6,PLPPR4
457	SPINK1 General Cancer Pathway	5.29E-01	3.64E-02	NaN	PIK3R4,RASD2
458	Hepatic Fibrosis / Hepatic Stellate Cell Activation	5.26E-01	2.72E-02	NaN	IGF1R,IGFBP4,LHX2,SMAD3,TGFB2
459	Small Cell Lung Cancer Signaling	5.24E-01	3.09E-02	NaN	E2F6,PIK3R4,PTGS2
460	TGF-Œ≤ Signaling	5.24E-01	3.09E-02	NaN	RASD2,SMAD3,TGFB2
461	Formation of the nephric duct	5.21E-01	5.56E-02	NaN	PCDH19
462	CNTF Signaling	5.19E-01	3.57E-02	NaN	PIK3R4,RASD2
463	Role of Pattern Recognition Receptors in Recognition of Bacteria ar	5.13E-01	2.82E-02	NaN	PIK3R4,PRKCA,PRKCB,TGFB2
464	Circadian Clock	5.09E-01	3.51E-02	NaN	NR3C1,PPP1CC
465	Unfolded Protein Response (UPR)	5.02E-01	5.26E-02	NaN	EIF2S2
466	Fatty Acid Œ±-oxidation	5.02E-01	5.26E-02	NaN	PTGS2
467	Cancer Drug Resistance by Drug Efflux	4.98E-01	3.45E-02	NaN	PTGS2,RASD2
468	Apoptosis Signaling	4.94E-01	2.97E-02	NaN	PARP1,PRKCA,RASD2
469	GADD45 Signaling	4.89E-01	3.39E-02	NaN	SMAD3,TGFB2
470	Sleep REM Signaling Pathway	4.87E-01	2.94E-02	NaN	EGR1,PRKAG2,TGFB2
471	Endometrial Cancer Signaling	4.79E-01	3.33E-02	NaN	PIK3R4,RASD2
472	Extracellular matrix organization	4.72E-01	2.88E-02	NaN	ITGA7,NCAM1,TGFB2
473	MSP-RON Signaling in Macrophages Pathway	4.72E-01	2.88E-02	NaN	PIK3R4,PTGS2,RASD2
474	IL-2 Signaling	4.70E-01	3.28E-02	NaN	PIK3R4,RASD2
475	Formation of the ureteric bud	4.66E-01	4.76E-02	NaN	SLIT2
476	Pregnenolone Biosynthesis	4.66E-01	4.76E-02	NaN	CYP26B1
477	Methionine Degradation I (to Homocysteine)	4.66E-01	4.76E-02	NaN	AHCYL2
478	PTEN Signaling	4.65E-01	2.67E-02	NaN	IGF1R,INPP5F,ITGA7,RASD2
479	Phagosome Maturation	4.59E-01	2.65E-02	NaN	NAPG,PRDX6,TUBB3,VTI1B
480	Paxillin Signaling	4.59E-01	2.83E-02	NaN	ITGA7,PIK3R4,RASD2
481	Telomerase Signaling	4.59E-01	2.83E-02	NaN	ETS2,PIK3R4,RASD2
482	Signaling by VEGF	4.52E-01	2.80E-02	NaN	ITPR1,PRKCA,PRKCB
483	Regulation of Actin-based Motility by Rho	4.52E-01	2.80E-02	NaN	ARPC2,ITGA7,PFN1
484	NCAM signaling for neurite out-growth	4.52E-01	3.17E-02	NaN	CACNA1C,NCAM1
485	Other interleukin signaling	4.50E-01	4.55E-02	NaN	IL16
486	Histidine Degradation VI	4.50E-01	4.55E-02	NaN	CYP26B1
487	MHC class II antigen presentation	4.45E-01	2.78E-02	NaN	CAPZB,DCTN6,TUBB3
488	Nucleotide Excision Repair	4.45E-01	2.78E-02	NaN	COPS4,PARP1,POLE4
489	Translocation of SLC2A4 (GLUT4) to the plasma membrane	4.43E-01	3.12E-02	NaN	PRKAG2,TUBB3
490	Amyloid fiber formation	4.43E-01	3.12E-02	NaN	GGA3,ITM2B
491	ERB2-ERBB3 Signaling	4.43E-01	3.12E-02	NaN	PIK3R4,RASD2
492	O-linked glycosylation	4.39E-01	2.75E-02	NaN	GALNT16,GALNT17,SPON1
493	Surfactant metabolism	4.35E-01	4.35E-02	NaN	ZDHHC2
494	Nucleotide salvage	4.35E-01	4.35E-02	NaN	APRT
495	RAS processing	4.35E-01	4.35E-02	NaN	GOLGA7
496	Cysteine Biosynthesis III (mammalia)	4.35E-01	4.35E-02	NaN	AHCYL2
497	CD40 Signaling	4.18E-01	2.99E-02	NaN	PIK3R4,PTGS2
498	Prostate Cancer Signaling	4.14E-01	2.65E-02	NaN	E2F6,PIK3R4,RASD2
499	Role of JAK1 and JAK3 in Œ≥c Cytokine Signaling	4.10E-01	2.94E-02	NaN	PIK3R4,RASD2
500	LXR/RXR Activation	4.08E-01	2.63E-02	NaN	ARG2,MYLIP,PTGS2
501	Activation of Matrix Metalloproteinases	4.06E-01	4.00E-02	NaN	MMP17
502	Cholesterol biosynthesis	4.06E-01	4.00E-02	NaN	PLPP6
503	Insulin processing	4.06E-01	4.00E-02	NaN	PCSK2
504	Miscellaneous transport and binding events	4.06E-01	4.00E-02	NaN	LRRC8D
505	Ubiquinol-10 Biosynthesis (Eukaryotic)	4.06E-01	4.00E-02	NaN	CYP26B1
506	Glioma Invasiveness Signaling	3.95E-01	2.86E-02	NaN	PIK3R4,RASD2

	A	B	C	D	E
507	ATF4 activates genes in response to endoplasmic reticulum stress	3.93E-01	3.85E-02	NaN	NFYB
508	Estrogen-mediated S-phase Entry	3.93E-01	3.85E-02	NaN	E2F6
509	Gluconeogenesis I	3.93E-01	3.85E-02	NaN	PGAM2
510	NOD1/2 Signaling Pathway	3.92E-01	2.44E-02	NaN	HSPA1A,Hspa1b,HSPA2,TGFB2
511	COPII-mediated vesicle transport	3.88E-01	2.82E-02	NaN	LMAN2,NAPG
512	Effects of PIP2 hydrolysis	3.81E-01	3.70E-02	NaN	ITPR1
513	Syndecan interactions	3.81E-01	3.70E-02	NaN	PRKCA
514	CDP-diacylglycerol Biosynthesis I	3.81E-01	3.70E-02	NaN	CDS1
515	Role of MAPK Signaling in Inhibiting the Pathogenesis of Influenza	3.80E-01	2.78E-02	NaN	PRDX6,PTGS2
516	Role of PKR in Interferon Induction and Antiviral Response	3.74E-01	2.50E-02	NaN	HSPA1A,Hspa1b,HSPA2
517	IL-17A Signaling in Fibroblasts	3.73E-01	2.74E-02	NaN	PRKCA,PRKCB
518	EIF2 Signaling	3.71E-01	2.30E-02	NaN	EIF2S2,IGF1R,PIK3R4,PPP1CC,RASD2
519	MTOR signalling	3.69E-01	3.57E-02	NaN	LAMTOR2
520	Role of p14/p19ARF in Tumor Suppression	3.69E-01	3.57E-02	NaN	PIK3R4
521	Extra-nuclear estrogen signaling	3.66E-01	2.70E-02	NaN	GNB5,IGF1R
522	Caveolar-mediated Endocytosis Signaling	3.66E-01	2.70E-02	NaN	ITGA7,PRKCA
523	Role of NANOG in Mammalian Embryonic Stem Cell Pluripotency	3.64E-01	2.46E-02	NaN	BMP3,PIK3R4,RASD2
524	Signaling by NOTCH1	3.60E-01	2.67E-02	NaN	CNTN1,FBXW7
525	trans-Golgi Network Vesicle Budding	3.60E-01	2.67E-02	NaN	DTNBP1,NECAP1
526	IL-27 Signaling Pathway	3.58E-01	2.44E-02	NaN	PIK3R4,PTGS2,SMAD3
527	Ferroptosis Signaling Pathway	3.58E-01	2.44E-02	NaN	PRKAG2,RASD2,SLC39A14
528	Toll-like Receptor Cascades	3.58E-01	3.45E-02	NaN	PIK3R4
529	Processing of Capped Intronless Pre-mRNA	3.58E-01	3.45E-02	NaN	LSM11
530	EGR2 and SOX10-mediated initiation of Schwann cell myelination	3.58E-01	3.45E-02	NaN	POU3F1
531	Airway Inflammation in Asthma	3.58E-01	3.45E-02	NaN	TGFB2
532	Phosphatidylglycerol Biosynthesis II (Non-plastidic)	3.58E-01	3.45E-02	NaN	CDS1
533	Sonic Hedgehog Signaling	3.58E-01	3.45E-02	NaN	PRKAG2
534	Angiopoietin Signaling	3.53E-01	2.63E-02	NaN	PIK3R4,RASD2
535	Nicotinate metabolism	3.47E-01	3.33E-02	NaN	PTGS2
536	Renal Cell Carcinoma Signaling	3.40E-01	2.56E-02	NaN	PIK3R4,RASD2
537	Transcriptional regulation of brown and beige adipocyte differentia	3.37E-01	3.23E-02	NaN	CIDEA
538	Phase I - Functionalization of compounds	3.34E-01	2.53E-02	NaN	ARNT2,CYP26B1
539	Xenobiotic Metabolism AHR Signaling Pathway	3.34E-01	2.53E-02	NaN	GSTO1,MGST3
540	Regulation of eIF4 and p70S6K Signaling	3.31E-01	2.25E-02	NaN	EIF2S2,ITGA7,PIK3R4,RASD2
541	G-protein beta:gamma signalling	3.27E-01	3.12E-02	NaN	GNB5
542	Thrombin signalling through proteinase activated receptors (PARs)	3.27E-01	3.12E-02	NaN	GNB5
543	Cargo concentration in the ER	3.27E-01	3.12E-02	NaN	LMAN2
544	GŒ±12/13 Signaling	3.24E-01	2.31E-02	NaN	CDH9,PIK3R4,RASD2
545	FLT3 Signaling in Hematopoietic Progenitor Cells	3.22E-01	2.47E-02	NaN	PIK3R4,RASD2
546	IL-10 Signaling	3.20E-01	2.29E-02	NaN	ARG2,BHLHE40,PRKAG2
547	Arachidonic acid metabolism	3.17E-01	3.03E-02	NaN	PTGS2
548	Signal amplification	3.17E-01	3.03E-02	NaN	GNB5
549	MAPK targets/ Nuclear events mediated by MAP kinases	3.17E-01	3.03E-02	NaN	DUSP6
550	JAK/STAT Signaling	3.16E-01	2.44E-02	NaN	PIK3R4,RASD2
551	Signaling by NOTCH2	3.08E-01	2.94E-02	NaN	CNTN1
552	Oncogene Induced Senescence	3.08E-01	2.94E-02	NaN	ETS2
553	Activation of the pre-replicative complex	3.08E-01	2.94E-02	NaN	POLE4
554	Transcriptional Regulation by E2F6	3.08E-01	2.94E-02	NaN	E2F6
555	Inhibition of Matrix Metalloproteases	3.08E-01	2.94E-02	NaN	MMP17
556	Neuroinflammation Signaling Pathway	3.01E-01	2.09E-02	NaN	GABRA1,GABRB3,NTF3,PIK3R4,PTGS2,TGFB2
557	TP53 Regulates Metabolic Genes	3.00E-01	2.35E-02	NaN	LAMTOR2,PRKAG2
558	Detoxification of Reactive Oxygen Species	2.99E-01	2.86E-02	NaN	PRDX6
559	Mitochondrial Fatty Acid Beta-Oxidation	2.99E-01	2.86E-02	NaN	ACOT13
560	Transcriptional regulation by the AP-2 (TFAP2) family of transcriptio	2.99E-01	2.86E-02	NaN	KIT
561	IL-9 Signaling	2.99E-01	2.86E-02	NaN	PIK3R4
562	Hereditary Breast Cancer Signaling	2.98E-01	2.21E-02	NaN	PIK3R4,RASD2,TGFB2
563	HIPPO signaling	2.94E-01	2.33E-02	NaN	PPP1CC,SMAD3
564	STAT3 Pathway	2.93E-01	2.19E-02	NaN	IGF1R,RASD2,TGFB2
565	Assembly and cell surface presentation of NMDA receptors	2.91E-01	2.78E-02	NaN	TUBB3
566	Aggrephagy	2.91E-01	2.78E-02	NaN	TUBB3
567	Superpathway of Methionine Degradation	2.91E-01	2.78E-02	NaN	AHCYL2
568	AMPK Signaling	2.90E-01	2.08E-02	NaN	GNB5,PFKP,PIK3R4,PPM1E,PRKAG2
569	Signaling by NOTCH4	2.89E-01	2.17E-02	NaN	FBXW7,PIK3R4,SMAD3
570	MSP-RON Signaling in Cancer Cells Pathway	2.89E-01	2.17E-02	NaN	ETS2,PIK3R4,RASD2
571	ABRA Signaling Pathway	2.89E-01	2.30E-02	NaN	ABLIM2,EGR1
572	Mitotic Prophase	2.89E-01	2.30E-02	NaN	PRKCA,PRKCB
573	FcŒ≥RIIB Signaling in B Lymphocytes	2.87E-01	2.04E-02	NaN	CACNA1C,CACNA2D1,CACNA2D3,CACNG3,ITPR1,PIK3R4,RASD2
574	Regulation of Cellular Mechanics by Calpain Protease	2.84E-01	2.27E-02	NaN	ITGA7,RASD2
575	NER (Nucleotide Excision Repair, Enhanced Pathway)	2.84E-01	2.27E-02	NaN	COPS4,POLE4
576	Interleukin-10 signaling	2.83E-01	2.70E-02	NaN	PTGS2
577	Cell Cycle Regulation by BTG Family Proteins	2.83E-01	2.70E-02	NaN	E2F6
578	Regulation of mRNA stability by proteins that bind AU-rich element	2.79E-01	2.25E-02	NaN	HSPA1A,PRKCA
579	RAB GEFs exchange GTP for GDP on RABs	2.79E-01	2.25E-02	NaN	DENND5A,RAB8B
580	Regulation of TP53 Expression and Degradation	2.75E-01	2.63E-02	NaN	PHF20
581	Notch Signaling	2.75E-01	2.63E-02	NaN	CNTN1
582	Neurotransmitter release cycle	2.67E-01	2.56E-02	NaN	PPFIA2
583	PI3K/AKT Signaling	2.63E-01	2.03E-02	NaN	INPP5F,ITGA7,PTGS2,RASD2
584	Gap junction trafficking and regulation	2.60E-01	2.50E-02	NaN	TUBB3
585	Activation of gene expression by SREBF (SREBP)	2.60E-01	2.50E-02	NaN	NFYB
586	RET signaling	2.60E-01	2.50E-02	NaN	PRKCA
587	Oncostatin M Signaling	2.53E-01	2.44E-02	NaN	RASD2
588	Aryl Hydrocarbon Receptor Signaling	2.51E-01	2.03E-02	NaN	GSTO1,MGST3,TGFB2
589	Elastic fibre formation	2.46E-01	2.38E-02	NaN	TGFB2
590	Formation of WDR5-containing histone-modifying complexes	2.46E-01	2.38E-02	NaN	PHF20
591	ATM Signaling	2.42E-01	2.06E-02	NaN	HP1BP3,PPP1CC
592	MIF Regulation of Innate Immunity	2.40E-01	2.33E-02	NaN	PTGS2
593	BER (Base Excision Repair) Pathway	2.40E-01	2.33E-02	NaN	PARP1
594	p75 NTR receptor-mediated signalling	2.37E-01	1.97E-02	NaN	ARHGEF26,ARHGEF4,PIK3R4
595	PI3K Cascade	2.34E-01	2.27E-02	NaN	PIK3R4
596	Interleukin-2 family signaling	2.34E-01	2.27E-02	NaN	INPP5F
597	Retinoic acid Mediated Apoptosis Signaling	2.34E-01	2.27E-02	NaN	PARP1
598	IL-13 Signaling Pathway	2.30E-01	2.00E-02	NaN	PIK3R4,TGFB2
599	tRNA Splicing	2.28E-01	2.22E-02	NaN	PDE7A
600	IL-23 Signaling Pathway	2.28E-01	2.22E-02	NaN	PIK3R4
601	DNA Damage Bypass	2.11E-01	2.08E-02	NaN	POLE4
602	NR1H2 and NR1H3-mediated signaling	2.11E-01	2.08E-02	NaN	MYLIP
603	Interferon alpha/beta signaling	2.11E-01	2.08E-02	NaN	EGR1
604	MYC Mediated Apoptosis Signaling	2.11E-01	2.08E-02	NaN	PRKAG2
605	TP53 Regulates Transcription of Cell Cycle Genes	2.05E-01	2.04E-02	NaN	PLK2
606	Signaling by ERBB2	2.00E-01	2.00E-02	NaN	PRKCA
607	Cell Cycle: G2/M DNA Damage Checkpoint Regulation	2.00E-01	2.00E-02	NaN	FBXL5

	A	B	C	D	E
608	Pyroptosis Signaling Pathway	0.00E+00	1.25E-02	NaN	PRKAG2
609	Wound Healing Signaling Pathway	0.00E+00	1.23E-02	NaN	PRKCA,RASD2,TGFB2
610	Macrophage Classical Activation Signaling Pathway	0.00E+00	1.28E-02	NaN	DLAT,TGFB2
611	Multiple Sclerosis Signaling Pathway	0.00E+00	1.57E-02	NaN	DUSP6,PARP1,TGFB2
612	Pathogen Induced Cytokine Storm Signaling Pathway	0.00E+00	6.33E-03	NaN	BHLHE40,TGFB2
613	Macrophage Alternative Activation Signaling Pathway	0.00E+00	1.84E-02	NaN	NR3C1,PIK3R4,TGFB2
614	Microautophagy Signaling Pathway	0.00E+00	6.49E-03	NaN	PRKAG2
615	p53 Signaling	0.00E+00	1.05E-02	NaN	PIK3R4
616	PIP3 activates AKT signaling	0.00E+00	1.37E-02	NaN	KIT,NTF3
617	Mitochondrial biogenesis	0.00E+00	1.64E-02	NaN	PRKAG2
618	Gamma carboxylation, hypusinylation, hydroxylation, and arylsulfat	0.00E+00	1.75E-02	NaN	ASPH
619	Plasma lipoprotein assembly, remodeling, and clearance	0.00E+00	1.37E-02	NaN	MYLIP
620	Signaling by NTRK1 (TRKA)	0.00E+00	1.23E-02	NaN	DUSP6
621	Degradation of beta-catenin by the destruction complex	0.00E+00	1.11E-02	NaN	CSNK1A1
622	Metabolism of water-soluble vitamins and cofactors	0.00E+00	1.43E-02	NaN	GSTO1
623	TCF dependent signaling in response to WNT	0.00E+00	1.11E-02	NaN	CSNK1A1,RSPO2
624	Cell surface interactions at the vascular wall	0.00E+00	8.70E-03	NaN	SIRPA
625	Generic Transcription Pathway	0.00E+00	1.58E-02	NaN	MED10,NR3C1,NR3C2
626	RNA Polymerase II Transcription	0.00E+00	6.85E-03	NaN	LSM11
627	Asparagine N-linked glycosylation	0.00E+00	8.20E-03	NaN	MAN1A1
628	Integrin cell surface interactions	0.00E+00	1.19E-02	NaN	ITGA7
629	Abacavir ADME	0.00E+00	1.61E-02	NaN	PIK3R4
630	Selenoamino acid metabolism	0.00E+00	7.81E-03	NaN	PAPSS1
631	Fc epsilon receptor (FCERI) signaling	0.00E+00	7.52E-03	NaN	ITPR1
632	Mitotic Metaphase and Anaphase	0.00E+00	1.34E-02	NaN	CLIP1,PPP1CC,TUBB3
633	SUMOylation of DNA damage response and repair proteins	0.00E+00	1.35E-02	NaN	PARP1
634	Metabolism of polyamines	0.00E+00	1.69E-02	NaN	SMS
635	Class B/2 (Secretin family receptors)	0.00E+00	1.06E-02	NaN	GNB5
636	Semaphorin interactions	0.00E+00	1.56E-02	NaN	PLXNA1
637	Transcriptional regulation of white adipocyte differentiation	0.00E+00	1.22E-02	NaN	MED10
638	ABC-family proteins mediated transport	0.00E+00	1.04E-02	NaN	EIF2S2
639	Costimulation by the CD28 family	0.00E+00	8.93E-03	NaN	PIK3R4
640	G alpha (q) signalling events	0.00E+00	1.22E-02	NaN	GNB5,NTSR2
641	Transport of bile salts and organic acids, metal ions and amine com	0.00E+00	1.28E-02	NaN	SLC39A14
642	NoRC negatively regulates rRNA expression	0.00E+00	1.96E-02	NaN	MBD2
643	Sphingolipid metabolism	0.00E+00	1.94E-02	NaN	ST6GALNAC5,ST8SIA5
644	Interleukin-1 family signaling	0.00E+00	1.59E-02	NaN	PTPN5,SMAD3
645	Mitotic G2-G2/M phases	0.00E+00	5.32E-03	NaN	TUBB3
646	Mitotic G1 phase and G1/S transition	0.00E+00	7.69E-03	NaN	E2F6
647	Interleukin-3, Interleukin-5 and GM-CSF signaling	0.00E+00	1.89E-02	NaN	INPP5F
648	Mitochondrial translation	0.00E+00	1.03E-02	NaN	MRPL20
649	Cilium Assembly	0.00E+00	1.02E-02	NaN	ASAP1,TUBB3
650	C-type lectin receptors (CLRs)	0.00E+00	7.46E-03	NaN	ITPR1
651	Hedgehog 'on' state	0.00E+00	1.42E-02	NaN	CSNK1A1,PIK3R4
652	Signaling by FGFR1	0.00E+00	1.96E-02	NaN	FLRT3
653	Deubiquitination	0.00E+00	8.30E-03	NaN	RNF128,SMAD3
654	HDR through Homologous Recombination (HRR) or Single Strand A	0.00E+00	9.80E-03	NaN	POLE4
655	Interleukin-4 and Interleukin-13 signaling	0.00E+00	1.94E-02	NaN	FSCN1,PTGS2
656	Major pathway of rRNA processing in the nucleolus and cytosol	0.00E+00	1.12E-02	NaN	BYSL,TBL3
657	Neurexins and neuroligins	0.00E+00	1.82E-02	NaN	HOMER3
658	Regulation of TP53 Activity through Phosphorylation	0.00E+00	1.10E-02	NaN	PRKAG2
659	Signaling by MET	0.00E+00	1.27E-02	NaN	GGA3
660	PTEN Regulation	0.00E+00	1.35E-02	NaN	EGR1,LAMTOR2
661	Mitotic Prometaphase	0.00E+00	1.55E-02	NaN	CLIP1,PPP1CC,TUBB3
662	Synthesis of DNA	0.00E+00	8.26E-03	NaN	POLE4
663	Cell Cycle Checkpoints	0.00E+00	1.16E-02	NaN	CLIP1,PHF20,PPP1CC
664	Eukaryotic Translation Initiation	0.00E+00	7.63E-03	NaN	EIF2S2
665	RNA Polymerase I Transcription	0.00E+00	1.75E-02	NaN	MBD2
666	Interferon gamma signaling	0.00E+00	1.49E-02	NaN	NCAM1
667	Signaling by PTK6	0.00E+00	1.85E-02	NaN	NR3C1
668	Cargo recognition for clathrin-mediated endocytosis	0.00E+00	1.92E-02	NaN	COPS4,NECAP1
669	Clathrin-mediated endocytosis	0.00E+00	1.56E-02	NaN	ARPC2,NECAP1
670	RAB geranylgeranylation	0.00E+00	1.59E-02	NaN	RAB8B
671	Transcriptional regulation by RUNX1	0.00E+00	7.35E-03	NaN	PRKCB
672	Transcriptional regulation by RUNX3	0.00E+00	1.05E-02	NaN	SMAD3
673	ESR-mediated signaling	0.00E+00	9.71E-03	NaN	FKBP5
674	Regulation of RUNX2 expression and activity	0.00E+00	1.39E-02	NaN	NR3C1
675	Transcriptional Regulation by MECP2	0.00E+00	1.61E-02	NaN	FKBP5
676	Iron uptake and transport	0.00E+00	1.69E-02	NaN	FBXL5
677	Nonsense-Mediated Decay (NMD)	0.00E+00	8.93E-03	NaN	UPF2
678	Response of EIF2AK4 (GCN2) to amino acid deficiency	0.00E+00	1.01E-02	NaN	EIF2S2
679	Amino acids regulate mTORC1	0.00E+00	1.82E-02	NaN	LAMTOR2
680	Expression and translocation of olfactory receptors	0.00E+00	1.18E-02	NaN	LHX2
681	KEAP1-NFE2L2 pathway	0.00E+00	6.80E-03	NaN	PIK3R4
682	Kinesins	0.00E+00	1.96E-02	NaN	TUBB3
683	Signaling by the B Cell Receptor (BCR)	0.00E+00	1.80E-02	NaN	ITPR1,PRKCB
684	Acute Phase Response Signaling	0.00E+00	1.14E-02	NaN	NR3C1,RASD2
685	CGAS-STING Signaling Pathway	0.00E+00	1.59E-02	NaN	PCDH7,TGFB2
686	UFMylation Signaling Pathway	0.00E+00	1.64E-02	NaN	CDK5RAP3
687	Hematoma Resolution Signaling Pathway	0.00E+00	1.63E-02	NaN	MMP17,PRKAG2,SIRPA,TGFB2
688	mRNA 3 Prime End Processing Signaling Pathway	0.00E+00	1.82E-02	NaN	E2F6,PPP1CC
689	Chromatin organization	0.00E+00	9.30E-03	NaN	NSD3,PHF20
690	rRNA modification in the nucleus and cytosol	0.00E+00	1.64E-02	NaN	TBL3
691	Ribavirin ADME	0.00E+00	1.59E-02	NaN	PIK3R4
692	Mitochondrial protein degradation	0.00E+00	1.04E-02	NaN	ARG2
693	Transcriptional and post-translational regulation of MITF-M expres	0.00E+00	1.96E-02	NaN	KIT
694	Cyclophilin Signaling Pathway	0.00E+00	4.24E-03	NaN	PIK3R4
695	TR/RXR Activation	0.00E+00	1.59E-02	NaN	PFKP,PIK3R4
696	Role of BRCA1 in DNA Damage Response	0.00E+00	1.25E-02	NaN	E2F6
697	Clathrin-mediated Endocytosis Signaling	0.00E+00	1.49E-02	NaN	ARPC2,FGF11,PIK3R4
698	Role of NFAT in Regulation of the Immune Response	0.00E+00	7.72E-03	NaN	CSNK1A1,GNB5,ITPR1,PIK3R4,RASD2
699	Calcium-induced T Lymphocyte Apoptosis	0.00E+00	1.15E-02	NaN	ITPR1,PRKCA,PRKCB
700	Lymphotoxin Œ≤ Receptor Signaling	0.00E+00	1.85E-02	NaN	PIK3R4
701	Airway Pathology in Chronic Obstructive Pulmonary Disease	0.00E+00	1.92E-02	NaN	FGF11,TGFB2
702	CTLA4 Signaling in Cytotoxic T Lymphocytes	0.00E+00	7.58E-03	NaN	PIK3R4,RASD2,TGFB2
703	CD28 Signaling in T Helper Cells	0.00E+00	9.37E-03	NaN	ARPC2,ITPR1,PIK3R4
704	IL-15 Signaling	0.00E+00	5.90E-03	NaN	PIK3R4,RASD2
705	Agrin Interactions at Neuromuscular Junction	0.00E+00	1.47E-02	NaN	RASD2
706	Semaphorin Signaling in Neurons	0.00E+00	1.72E-02	NaN	PLXNA1
707	ICOS-ICOSL Signaling in T Helper Cells	0.00E+00	6.45E-03	NaN	ITPR1,PIK3R4

	A	B	C	D	E
708	Mitotic Roles of Polo-Like Kinase	0.00E+00	1.52E-02	NaN	PLK2
709	HMGB1 Signaling	0.00E+00	1.92E-02	NaN	PIK3R4,RASD2,TGFB2
710	Role of CHK Proteins in Cell Cycle Checkpoint Control	0.00E+00	1.75E-02	NaN	E2F6
711	Polyamine Regulation in Colon Cancer	0.00E+00	1.75E-02	NaN	SMS
712	Basal Cell Carcinoma Signaling	0.00E+00	1.45E-02	NaN	BMP3
713	ERK5 Signaling	0.00E+00	1.37E-02	NaN	RASD2
714	G Protein Signaling Mediated by Tubby	0.00E+00	3.56E-03	NaN	GNB5
715	Crosstalk between Dendritic Cells and Natural Killer Cells	0.00E+00	1.47E-02	NaN	FSCN1
716	Systemic Lupus Erythematosus Signaling	0.00E+00	4.67E-03	NaN	LSM11,PIK3R4,RASD2
717	CDC42 Signaling	0.00E+00	8.31E-03	NaN	ARPC2,CLIP1,ITGA7
718	ILK Signaling	0.00E+00	1.04E-02	NaN	PIK3R4,PTGS2
719	Role of Osteoblasts, Osteoclasts and Chondrocytes in Rheumatoid A	0.00E+00	1.81E-02	NaN	BMP3,CSNK1A1,FRZB,PIK3R4
720	Atherosclerosis Signaling	0.00E+00	8.55E-03	NaN	PRDX6
721	RANK Signaling in Osteoclasts	0.00E+00	1.10E-02	NaN	PIK3R4
722	Neuroprotective Role of THOP1 in Alzheimer's Disease	0.00E+00	9.90E-03	NaN	PRKAG2
723	Regulation of IL-2 Expression in Activated and Anergic T Lymphocyt	0.00E+00	1.00E-02	NaN	RASD2,SMAD3,TGFB2
724	Granzyme A Signaling	0.00E+00	1.43E-02	NaN	PARP1
725	Role of WNT/GSK-3Œ≤ Signaling in the Pathogenesis of Influenza	0.00E+00	1.54E-02	NaN	CSNK1A1
726	NUR77 Signaling in T Lymphocytes	0.00E+00	6.62E-03	NaN	PRKCA,PRKCB
727	PKCŒ∏ Signaling in T Lymphocytes	0.00E+00	1.95E-02	NaN	CACNA1C,CACNA2D1,CACNA2D3,CACNG3,ITPR1,PIK3R4,RASD2
728	Role of PI3K/AKT Signaling in the Pathogenesis of Influenza	0.00E+00	1.89E-02	NaN	PIK3R4
729	Antiproliferative Role of TOB in T Cell Signaling	0.00E+00	8.40E-03	NaN	SMAD3,TGFB2
730	MSP-RON Signaling Pathway	0.00E+00	1.75E-02	NaN	PIK3R4
731	IL-17A Signaling in Airway Cells	0.00E+00	1.61E-02	NaN	PIK3R4
732	Role of JAK2 in Hormone-like Cytokine Signaling	0.00E+00	1.82E-02	NaN	SIRPA
733	Role of JAK family kinases in IL-6-type Cytokine Signaling	0.00E+00	1.32E-02	NaN	TGFB2
734	NGF Signaling	0.00E+00	1.65E-02	NaN	PIK3R4,RASD2
735	Mouse Embryonic Stem Cell Pluripotency	0.00E+00	1.94E-02	NaN	PIK3R4,RASD2
736	Triacylglycerol Degradation	0.00E+00	1.82E-02	NaN	PRDX6
737	Agranulocyte Adhesion and Diapedesis	0.00E+00	5.43E-03	NaN	MMP17
738	Granulocyte Adhesion and Diapedesis	0.00E+00	6.06E-03	NaN	MMP17
739	TEC Kinase Signaling	0.00E+00	1.30E-02	NaN	GNB5,ITGA7,PIK3R4,PRKCA,PRKCB
740	Adipogenesis pathway	0.00E+00	1.46E-02	NaN	FBXW7,SMAD3
741	PCP (Planar Cell Polarity) Pathway	0.00E+00	1.67E-02	NaN	PFN1
742	SAPK/JNK Signaling	0.00E+00	9.55E-03	NaN	GNB5,PIK3R4,RASD2
743	WNT/Œ≤-catenin Signaling	0.00E+00	1.76E-02	NaN	CSNK1A1,FRZB,TGFB2
744	Death Receptor Signaling	0.00E+00	1.08E-02	NaN	PARP1
745	PPAR Signaling	0.00E+00	1.90E-02	NaN	PTGS2,RASD2
746	p38 MAPK Signaling	0.00E+00	8.62E-03	NaN	TGFB2
747	NF-Œ∫B Signaling	0.00E+00	1.06E-02	NaN	IGF1R,PIK3R4,PRKCB,RASD2
748	Hypoxia Signaling in the Cardiovascular System	0.00E+00	1.32E-02	NaN	UBE2E2
749	IL-6 Signaling	0.00E+00	1.61E-02	NaN	PIK3R4,RASD2
750	Sumoylation Pathway	0.00E+00	1.02E-02	NaN	NR3C1
751	Th1 and Th2 Activation Pathway	0.00E+00	6.41E-03	NaN	PIK3R4
752	Th1 Pathway	0.00E+00	9.26E-03	NaN	PIK3R4
753	Th2 Pathway	0.00E+00	8.06E-03	NaN	PIK3R4
754	IL-7 Signaling Pathway	0.00E+00	1.30E-02	NaN	PIK3R4
755	SPINK1 Pancreatic Cancer Pathway	0.00E+00	1.96E-02	NaN	SMAD3
756	T Cell Exhaustion Signaling Pathway	0.00E+00	1.15E-02	NaN	FOXP1,PIK3R4,RASD2,SMAD3
757	Systemic Lupus Erythematosus in T Cell Signaling Pathway	0.00E+00	7.08E-03	NaN	ITPR1,PIK3R4,RASD2
758	Inhibition of ARE-Mediated mRNA Degradation Pathway	0.00E+00	6.37E-03	NaN	PRKAG2
759	HOTAIR Regulatory Pathway	0.00E+00	1.27E-02	NaN	MMP17,PIK3R4
760	BEX2 Signaling Pathway	0.00E+00	1.25E-02	NaN	BEX2
761	Necroptosis Signaling Pathway	0.00E+00	1.39E-02	NaN	DAPK1,TYRO3
762	Kinetochore Metaphase Signaling Pathway	0.00E+00	9.26E-03	NaN	PPP1CC
763	Coronavirus Pathogenesis Pathway	0.00E+00	1.60E-02	NaN	E2F6,PTGS2,SMAD3
764	Coronavirus Replication Pathway	0.00E+00	1.47E-02	NaN	Tubb2b,TUBB3
765	Ingenuity Canonical Pathways	-log(p-value)	Ratio	z-score	Molecules
766	¬© 2000-2024 QIAGEN. All rights reserved.				
767					

	A	B	C	D	E	F
1	**Supplementary Table 2- IPA pathways CA3**					
2						
3	¬© 2000-2024 QIAGEN. All rights reserved.					
4						
5	**Ingenuity Canonical Pathways**	**-log(p-value**	**Ratio**	**z-score**	**Molecules**	
6	Axonal Guidance Signaling	3.78E+00	2.64E-02	NaN	ARPC2,BCAR1,BMP3,EPHB1,ITGA7,PRKCA,PRKCD,RASD1,ROBO3,SLIT1,SLIT2,Tubb2b,WNT7B	
7	G alpha (z) signalling events	3.24E+00	8.33E-02	1	ADCY2,PRKCA,PRKCD,RGS4	
8	Xenobiotic Metabolism Signaling	2.89E+00	3.03E-02	NaN	HS3ST4,HS6ST2,KEAP1,PPP2R5E,PRKCA,PRKCD,RASD1,WSCD1	
9	SUMOylation of intracellular receptors	2.81E+00	1.03E-01	NaN	NR3C1,NR3C2,NR4A2	
10	ERK/MAPK Signaling	2.79E+00	3.29E-02	0	BCAR1,ITGA7,PLA2G6,PPP2R5E,PRKCA,PRKCD,RASD1	
11	Œ±-Adrenergic Signaling	2.76E+00	4.63E-02	NaN	ADCY2,PRKCA,PRKCD,PYGB,RASD1	
12	Opioid Signaling Pathway	2.75E+00	2.88E-02	-0.816	ADCY2,ARRB2,PRKCA,PRKCD,RASD1,RGS12,RGS14,RGS4	
13	Sleep NREM Signaling Pathway	2.67E+00	4.42E-02	-0.447	ADCY2,GABRB1,PARP1,PRKCA,RASD1	
14	Xenobiotic Metabolism CAR Signaling Pathway	2.64E+00	3.57E-02	0.447	HS3ST4,HS6ST2,PPP2R5E,PRKCA,PRKCD,WSCD1	
15	Synaptogenesis Signaling Pathway	2.44E+00	2.56E-02	-0.378	ADCY2,ARPC2,CDH9,EPHB1,MARCKS,PRKCD,RASD1,SYT12	
16	DAG and IP3 signaling	2.40E+00	7.50E-02	NaN	ADCY2,PRKCA,PRKCD	
17	Glutaminergic Receptor Signaling Pathway (Enhanced)	2.40E+00	2.52E-02	0	ADCY2,GABRB1,HOMER1,NR3C1,PLA2G6,PRKCA,PRKCD,SCN3B	
18	VEGF Family Ligand-Receptor Interactions	2.36E+00	4.82E-02	NaN	PLA2G6,PRKCA,PRKCD,RASD1	
19	Vasopressin regulates renal water homeostasis via Aquaporins	2.34E+00	7.14E-02	NaN	ADCY2,AQP4,MYO5B	
20	Regulation of TP53 Activity through Association with Co-factors	2.27E+00	1.43E-01	NaN	BANP,PPP1R13B	
21	Fcgamma receptor (FCGR) dependent phagocytosis	2.25E+00	4.49E-02	NaN	ARPC2,PLA2G6,PRKCD,WIPF3	
22	GŒ±i Signaling	2.25E+00	3.52E-02	-2	ADCY2,RASD1,RGS12,RGS14,RGS4	
23	Dermatan Sulfate Biosynthesis (Late Stages)	2.23E+00	6.52E-02	NaN	HS3ST4,HS6ST2,WSCD1	
24	FcŒ≥ Receptor-mediated Phagocytosis in Macrophages and Mon	2.22E+00	4.40E-02	-1	ARPC2,PLA2G6,PRKCA,PRKCD	
25	nNOS Signaling in Neurons	2.18E+00	6.25E-02	NaN	PRKCA,PRKCD,RASD1	
26	Prolactin Signaling	2.17E+00	4.26E-02	NaN	NR3C1,PRKCA,PRKCD,RASD1	
27	eNOS Signaling	2.16E+00	3.36E-02	1	ADCY2,AQP4,Hspa1b,PRKCA,PRKCD	
28	Chondroitin Sulfate Biosynthesis (Late Stages)	2.16E+00	6.12E-02	NaN	HS3ST4,HS6ST2,WSCD1	
29	Transcriptional activity of SMAD2/SMAD3:SMAD4 heterotrimer	2.11E+00	5.88E-02	NaN	FURIN,MEN1,PARP1	
30	UVC-Induced MAPK Signaling	2.11E+00	5.88E-02	NaN	PRKCA,PRKCD,RASD1	
31	Neurexins and neuroligins	2.02E+00	5.45E-02	NaN	HOMER1,HOMER3,SYT12	
32	CXCR4 Signaling	2.00E+00	3.07E-02	0	ADCY2,BCAR1,PRKCA,PRKCD,RASD1	
33	Chondroitin Sulfate Biosynthesis	1.97E+00	5.26E-02	NaN	HS3ST4,HS6ST2,WSCD1	
34	Integration of energy metabolism	1.97E+00	3.74E-02	0	ACSL4,ADCY2,MARCKS,PRKCA	
35	Xenobiotic Metabolism PXR Signaling Pathway	1.96E+00	2.99E-02	0	HS3ST4,HS6ST2,PRKCA,PRKCD,WSCD1	
36	Signaling by ERBB4	1.93E+00	5.08E-02	NaN	GABRB1,MXD4,STMN1	
37	Dermatan Sulfate Biosynthesis	1.93E+00	5.08E-02	NaN	HS3ST4,HS6ST2,WSCD1	
38	Pregnenolone Biosynthesis	1.92E+00	9.52E-02	NaN	CYP26B1,CYP7B1	
39	L1CAM interactions	1.89E+00	3.54E-02	1	ALCAM,CHL1,GAP43,SCN3B	
40	Cholecystokinin/Gastrin-mediated Signaling	1.89E+00	3.54E-02	NaN	BCAR1,PRKCA,PRKCD,RASD1	
41	Histidine Degradation VI	1.88E+00	9.09E-02	NaN	CYP26B1,CYP7B1	
42	Virus Entry via Endocytic Pathways	1.88E+00	3.51E-02	NaN	B2M,PRKCA,PRKCD,RASD1	
43	Fc Epsilon RI Signaling	1.87E+00	3.48E-02	NaN	PLA2G6,PRKCA,PRKCD,RASD1	
44	Gap Junction Signaling	1.86E+00	2.21E-02	-0.447	ADCY2,DAGLA,PLA2G6,PRKCA,PRKCD,RASD1,Tubb2b	
45	RAB geranylgeranylation	1.86E+00	4.76E-02	NaN	RAB15,RAB18,RAB3C	
46	Thrombopoietin Signaling	1.86E+00	4.76E-02	NaN	PRKCA,PRKCD,RASD1	
47	Neuregulin Signaling	1.84E+00	3.42E-02	NaN	ITGA7,PRKCA,PRKCD,RASD1	
48	Amyloid fiber formation	1.84E+00	4.69E-02	NaN	B2M,FURIN,TTR	
49	Sperm Motility	1.83E+00	2.41E-02	NaN	EPHB1,KIT,PLA2G6,PRKCA,PRKCD,TYRO3	
50	RHO GTPases Activate NADPH Oxidases	1.81E+00	8.33E-02	NaN	PRKCA,PRKCD	
51	Renin-Angiotensin Signaling	1.81E+00	3.33E-02	NaN	ADCY2,PRKCA,PRKCD,RASD1	
52	Endothelin-1 Signaling	1.80E+00	2.73E-02	-1	ADCY2,PLA2G6,PRKCA,PRKCD,RASD1	
53	ERBB4 Signaling	1.79E+00	4.48E-02	NaN	PRKCA,PRKCD,RASD1	
54	Activation of Matrix Metalloproteinases	1.78E+00	8.00E-02	NaN	FURIN,SPOCK3	
55	Ubiquinol-10 Biosynthesis (Eukaryotic)	1.78E+00	8.00E-02	NaN	CYP26B1,CYP7B1	
56	ROBO SLIT Signaling Pathway	1.76E+00	3.23E-02	0	ARPC2,ROBO3,SLIT1,SLIT2	
57	14-3-3-mediated Signaling	1.75E+00	3.20E-02	NaN	PRKCA,PRKCD,RASD1,Tubb2b	
58	RAF/MAP kinase cascade	1.74E+00	2.31E-02	0	ARRB2,DUSP5,GFRA2,KIT,PPP2R5E,RASAL1	
59	Synaptic Long Term Depression	1.72E+00	2.60E-02	NaN	PLA2G6,PPP2R5E,PRKCA,PRKCD,RASD1	
60	Effects of PIP2 hydrolysis	1.71E+00	7.41E-02	NaN	DAGLA,PRKCD	
61	G Beta Gamma Signaling	1.70E+00	3.10E-02	NaN	ADCY2,PRKCA,PRKCD,RASD1	
62	HGF Signaling	1.69E+00	3.08E-02	NaN	ITGA7,PRKCA,PRKCD,RASD1	
63	Xenobiotic Metabolism General Signaling Pathway	1.69E+00	3.08E-02	0	KEAP1,PRKCA,PRKCD,RASD1	
64	fMLP Signaling in Neutrophils	1.68E+00	3.05E-02	NaN	ARPC2,PRKCA,PRKCD,RASD1	
65	CCR3 Signaling in Eosinophils	1.68E+00	3.05E-02	NaN	PLA2G6,PRKCA,PRKCD,RASD1	
66	P2Y Purinergic Receptor Signaling Pathway	1.68E+00	3.05E-02	NaN	ADCY2,PRKCA,PRKCD,RASD1	
67	Caveolar-mediated Endocytosis Signaling	1.67E+00	4.05E-02	NaN	B2M,ITGA7,PRKCA	
68	Human Embryonic Stem Cell Pluripotency	1.67E+00	2.53E-02	0.447	BMP3,PRKCA,PRKCD,RASD1,WNT7B	
69	DHCR24 Signaling Pathway	1.66E+00	3.01E-02	-1	PRKCA,PRKCD,RASD1,TTR	
70	Eicosanoid Signaling	1.66E+00	2.21E-02	-0.816	ADCY2,ARRB2,PLA2G6,PRKCA,PRKCD,RASD1	
71	Macropinocytosis Signaling	1.64E+00	3.95E-02	NaN	PRKCA,PRKCD,RASD1	
72	Ephrin Receptor Signaling	1.64E+00	2.48E-02	NaN	ARPC2,BCAR1,EPHB1,ITGA7,RASD1	
73	Integrin Signaling	1.63E+00	2.46E-02	0	ARPC2,ASAP1,BCAR1,ITGA7,RASD1	
74	NF-Œ∫B Activation by Viruses	1.63E+00	3.90E-02	NaN	PRKCA,PRKCD,RASD1	
75	Dopamine Receptor Signaling	1.63E+00	3.90E-02	NaN	ADCY2,PPP2R5E,SLC18A2	
76	NADH Repair	1.63E+00	3.33E-01	NaN	NAXE	
77	Role of MAPK Signaling in the Pathogenesis of Influenza	1.61E+00	3.85E-02	NaN	PLA2G6,PRKCA,RASD1	
78	Heparan Sulfate Biosynthesis (Late Stages)	1.61E+00	3.85E-02	NaN	HS3ST4,HS6ST2,WSCD1	
79	IL-3 Signaling	1.58E+00	3.75E-02	NaN	PRKCA,PRKCD,RASD1	
80	Apelin Endothelial Signaling Pathway	1.57E+00	2.82E-02	NaN	ADCY2,PRKCA,PRKCD,RASD1	
81	Serotonin Receptor Signaling	1.53E+00	1.76E-02	0	ADCY2,ARRB2,GAP43,PLA2G6,PRKCA,PRKCD,RASD1,SLC18A2	
82	LPS-stimulated MAPK Signaling	1.52E+00	3.53E-02	NaN	PRKCA,PRKCD,RASD1	
83	Heparan Sulfate Biosynthesis	1.52E+00	3.53E-02	NaN	HS3ST4,HS6ST2,WSCD1	
84	Thrombin Signaling	1.51E+00	2.29E-02	NaN	ADCY2,ARHGEF26,PRKCA,PRKCD,RASD1	
85	NGF processing	1.50E+00	2.50E-01	NaN	FURIN	
86	MIF-mediated Glucocorticoid Regulation	1.50E+00	5.71E-02	NaN	NR3C1,PLA2G6	
87	Factors Promoting Cardiogenesis in Vertebrates	1.49E+00	2.67E-02	1	BMP3,PRKCA,PRKCD,WNT7B	
88	Opioid Signalling	1.47E+00	3.37E-02	NaN	ADCY2,PRKCA,PRKCD	
89	NRF2-mediated Oxidative Stress Response	1.46E+00	2.22E-02	0	KEAP1,PPIB,PRKCA,PRKCD,RASD1	
90	Actin Nucleation by ARP-WASP Complex	1.45E+00	3.33E-02	NaN	ARPC2,ITGA7,RASD1	
91	ERBB Signaling	1.42E+00	3.23E-02	NaN	PRKCA,PRKCD,RASD1	
92	Neurotransmitter release cycle	1.41E+00	5.13E-02	NaN	PPFIA2,SLC18A2	
93	Orexin Signaling Pathway	1.40E+00	2.14E-02	-0.447	ADCY2,DAGLA,PLA2G6,PRKCA,PRKCD	
94	Bile acid and bile salt metabolism	1.39E+00	5.00E-02	NaN	CYP7B1,OSBPL1A	
95	RET signaling	1.39E+00	5.00E-02	NaN	GFRA2,PRKCA	
96	Mechanisms of Viral Exit from Host Cells	1.39E+00	5.00E-02	NaN	PRKCA,PRKCD	
97	Germ Cell-Sertoli Cell Junction Signaling	1.39E+00	2.47E-02	NaN	BCAR1,KEAP1,RASD1,Tubb2b	
98	Superpathway of Inositol Phosphate Compounds	1.38E+00	2.11E-02	-1	DUSP5,INPP5A,PPFIA2,PPP1R13B,PPP2R5E	
99	Melanocyte Development and Pigmentation Signaling	1.37E+00	3.09E-02	NaN	ADCY2,KIT,RASD1	
100	UVA-Induced MAPK Signaling	1.37E+00	3.09E-02	NaN	PARP1,PRKCA,RASD1	
101	Docosahexaenoic Acid (DHA) Signaling	1.37E+00	2.09E-02	-0.447	ADCY2,PLA2G6,PRKCA,PRKCD,SYT12	
102	Signaling by SCF-KIT	1.36E+00	4.76E-02	NaN	KIT,PRKCA	

	A	B	C	D	E	F
103	Apoptosis Signaling	1.33E+00	2.97E-02	NaN	PARP1,PRKCA,RASD1	
104	Netrin-1 signaling	1.32E+00	4.55E-02	NaN	SLIT1,SLIT2	
105	Post-translational protein phosphorylation	1.31E+00	2.91E-02	NaN	GOLM1,MEN1,PRSS23	
106	Neutrophil degranulation	1.31E+00	1.68E-02	0.378	ATP11B,B2M,PRKCD,PYGB,RAB18,SRP14,TTR	
107	RHO GTPases Activate WASPs and WAVEs	1.30E+00	4.44E-02	NaN	ARPC2,WIPF3	
108	WNK Renal Signaling Pathway	1.30E+00	2.88E-02	NaN	NR3C2,PRKCA,PRKCD	
109	Paxillin Signaling	1.28E+00	2.83E-02	NaN	BCAR1,ITGA7,RASD1	
110	HSP90 chaperone cycle for steroid hormone receptors in the pres	1.27E+00	4.26E-02	NaN	NR3C1,NR3C2	
111	Signaling by VEGF	1.27E+00	2.80E-02	NaN	BCAR1,PRKCA,PRKCD	
112	Role of MAPK Signaling in Promoting the Pathogenesis of Influenz	1.25E+00	2.75E-02	NaN	PLA2G6,PRKCA,RASD1	
113	Oxytocin in Brain Signaling Pathway	1.22E+00	2.17E-02	-1	PLA2G6,PRKCA,PRKCD,RASD1	
114	Dopamine-DARPP32 Feedback in cAMP Signaling	1.22E+00	2.17E-02	NaN	ADCY2,PPP2R5E,PRKCA,PRKCD	
115	D-myo-inositol (1,4,5,6)-Tetrakisphosphate Biosynthesis	1.22E+00	2.17E-02	NaN	DUSP5,PPFIA2,PPP1R13B,PPP2R5E	
116	D-myo-inositol (3,4,5,6)-tetrakisphosphate Biosynthesis	1.22E+00	2.17E-02	NaN	DUSP5,PPFIA2,PPP1R13B,PPP2R5E	
117	Signaling by ERBB2	1.22E+00	4.00E-02	NaN	PRKCA,PRKCD	
118	CDK5 Signaling	1.21E+00	2.65E-02	NaN	ADCY2,PPP2R5E,RASD1	
119	Phosphatidylethanolamine Biosynthesis II	1.21E+00	1.25E-01	NaN	SELENOI	
120	Circadian Rhythm Signaling	1.20E+00	1.87E-02	NaN	ADCY2,NR3C1,PRKCA,PRKCD,RASD1	
121	Cachexia Signaling Pathway	1.20E+00	1.70E-02	0	ADCY2,CEBPD,Hspa1b,NR3C1,PRKCA,PRKCD	
122	UVB-Induced MAPK Signaling	1.19E+00	3.85E-02	NaN	PRKCA,PRKCD	
123	Acetylcholine Receptor Signaling Pathway	1.19E+00	2.12E-02	1	ADCY2,KEAP1,PRKCA,PRKCD	
124	GNRH Signaling	1.19E+00	2.12E-02	NaN	ADCY2,PRKCA,PRKCD,RASD1	
125	Regulation of Insulin-like Growth Factor (IGF) transport and uptak	1.17E+00	2.56E-02	NaN	GOLM1,MEN1,PRSS23	
126	GPCR-Mediated Nutrient Sensing in Enteroendocrine Cells	1.17E+00	2.56E-02	NaN	ADCY2,PRKCA,PRKCD	
127	Adrenergic Receptor Signaling Pathway (Enhanced)	1.17E+00	2.08E-02	1	ADCY2,PRKCA,PRKCD,SLC18A2	
128	IL-15 Production	1.17E+00	2.54E-02	NaN	EPHB1,KIT,TYRO3	
129	Insulin Secretion Signaling Pathway	1.16E+00	1.82E-02	1.342	ADCY2,FURIN,PRKCA,PRKCD,SRP14	
130	Signaling by PTK6	1.16E+00	3.70E-02	NaN	BCAR1,NR3C1	
131	Pulmonary Healing Signaling Pathway	1.16E+00	2.06E-02	0	PRKCA,PRKCD,RASD1,WNT7B	
132	3-phosphoinositide Degradation	1.15E+00	2.05E-02	NaN	DUSP5,PPFIA2,PPP1R13B,PPP2R5E	
133	Oxytocin Signaling Pathway	1.15E+00	1.81E-02	-1.342	ARRB2,PLA2G6,PRKCA,PRKCD,RASD1	
134	Glycerophospholipid biosynthesis	1.13E+00	2.46E-02	NaN	CPNE7,PLA2G6,SELENOI	
135	Role of NANOG in Mammalian Embryonic Stem Cell Pluripotency	1.13E+00	2.46E-02	NaN	BMP3,RASD1,WNT7B	
136	Ferroptosis Signaling Pathway	1.12E+00	2.44E-02	NaN	ACSL4,KEAP1,RASD1	
137	Role of Tissue Factor in Cancer	1.12E+00	2.00E-02	-2	ARRB2,PPP2R5E,PRKCA,RASD1	
138	D-myo-inositol-5-phosphate Metabolism	1.12E+00	2.00E-02	NaN	DUSP5,PPFIA2,PPP1R13B,PPP2R5E	
139	Glycosaminoglycan metabolism	1.12E+00	2.42E-02	NaN	HS3ST4,HS6ST2,ST3GAL2	
140	Glycogen Degradation II	1.11E+00	1.00E-01	NaN	PYGB	
141	Signaling by PDGF	1.11E+00	3.45E-02	NaN	BCAR1,FURIN	
142	Visual phototransduction	1.11E+00	3.45E-02	NaN	PRKCA,TTR	
143	Glioma Signaling	1.11E+00	2.40E-02	NaN	PRKCA,PRKCD,RASD1	
144	IL-8 Signaling	1.11E+00	1.98E-02	NaN	ARRB2,PRKCA,PRKCD,RASD1	
145	HIF1Œ± Signaling	1.11E+00	1.98E-02	0	Hspa1b,PRKCA,PRKCD,RASD1	
146	Cardiac conduction	1.10E+00	2.38E-02	NaN	FKBP1B,FXYD6,SCN3B	
147	mTOR Signaling	1.09E+00	1.95E-02	NaN	PPP2R5E,PRKCA,PRKCD,RASD1	
148	Clathrin-mediated endocytosis	1.08E+00	2.34E-02	NaN	ARPC2,ARRB2,REPS2	
149	GABA receptor activation	1.08E+00	3.33E-02	NaN	ADCY2,GABRB1	
150	Passive transport by Aquaporins	1.07E+00	9.09E-02	NaN	AQP4	
151	Hepatic Cholestasis	1.06E+00	1.90E-02	1	ADCY2,CYP7B1,PRKCA,PRKCD	
152	3-phosphoinositide Biosynthesis	1.06E+00	1.90E-02	NaN	DUSP5,PPFIA2,PPP1R13B,PPP2R5E	
153	Synaptic Long Term Potentiation	1.05E+00	2.27E-02	NaN	PRKCA,PRKCD,RASD1	
154	Remodeling of Epithelial Adherens Junctions	1.05E+00	3.17E-02	NaN	ARPC2,Tubb2b	
155	HDR through MMEJ (alt-NHEJ)	1.04E+00	8.33E-02	NaN	PARP1	
156	Glycogen Degradation III	1.04E+00	8.33E-02	NaN	PYGB	
157	Œ≥-glutamyl Cycle	1.04E+00	8.33E-02	NaN	GGT7	
158	RHOGDI Signaling	1.03E+00	1.86E-02	NaN	ARPC2,CDH9,ITGA7,PRKCA	
159	RAC Signaling	1.02E+00	2.19E-02	NaN	ARPC2,ITGA7,RASD1	
160	Mitochondrial calcium ion transport	1.01E+00	7.69E-02	NaN	MCUB	
161	Nephron development	1.01E+00	7.69E-02	NaN	POU3F3	
162	G alpha (i) signalling events	1.00E+00	1.82E-02	2	ADCY2,RGS12,RGS14,RGS4	
163	Interferon gamma signaling	1.00E+00	2.99E-02	NaN	B2M,PRKCD	
164	Glutamate Receptor Signaling	1.00E+00	2.99E-02	NaN	HOMER1,HOMER3	
165	Glycation Signaling Pathway	9.98E-01	1.81E-02	0	PRKCA,PRKCD,RASD1,TTR	
166	Estrogen Receptor Signaling	9.88E-01	1.49E-02	0	ADCY2,NR3C1,PRKCA,PRKCD,RASD1,SDHD	
167	Role of NFAT in Cardiac Hypertrophy	9.82E-01	1.79E-02	NaN	ADCY2,PRKCA,PRKCD,RASD1	
168	Hepatic Fibrosis Signaling Pathway	9.81E-01	1.49E-02	0	ITGA7,PRKCA,PRKCD,RASD1,SDHD,WNT7B	
169	Growth Hormone Signaling	9.80E-01	2.90E-02	NaN	PRKCA,PRKCD	
170	Basal Cell Carcinoma Signaling	9.80E-01	2.90E-02	NaN	BMP3,WNT7B	
171	Role of Macrophages, Fibroblasts and Endothelial Cells in Rheuma	9.77E-01	1.60E-02	-0.447	CEBPD,PRKCA,PRKCD,RASD1,WNT7B	
172	DNA Double-Strand Break Repair by Non-Homologous End Joining	9.75E-01	7.14E-02	NaN	PARP1	
173	Leukotriene Biosynthesis	9.75E-01	7.14E-02	NaN	GGT7	
174	Signaling by ROBO receptors	9.71E-01	1.77E-02	-1	PRKCA,ROBO3,SLIT1,SLIT2	
175	Melatonin Signaling	9.59E-01	2.82E-02	NaN	PRKCA,PRKCD	
176	Rap1 signalling	9.47E-01	6.67E-02	NaN	RAP1GAP2	
177	Fatty Acid Activation	9.47E-01	6.67E-02	NaN	ACSL4	
178	Sertoli Cell-Germ Cell Junction Signaling Pathway (Enhanced)	9.46E-01	1.73E-02	-1	ARPC2,BCAR1,PPP2R5E,RASD1	
179	IL-17A Signaling in Fibroblasts	9.39E-01	2.74E-02	NaN	CEBPD,PRKCA	
180	cAMP-mediated signaling	9.31E-01	1.71E-02	-1	ADCY2,RGS12,RGS14,RGS4	
181	VDR/RXR Activation	9.29E-01	2.70E-02	NaN	PRKCA,PRKCD	
182	WNT/SHH Axonal Guidance Signaling Pathway	9.28E-01	2.00E-02	NaN	ADCY2,PRKCA,WNT7B	
183	PTEN Signaling	9.28E-01	2.00E-02	NaN	BCAR1,ITGA7,RASD1	
184	Corticotropin Releasing Hormone Signaling	9.22E-01	1.99E-02	NaN	ADCY2,PRKCA,PRKCD	
185	LPS/IL-1 Mediated Inhibition of RXR Function	9.21E-01	1.69E-02	NaN	ACSL4,HS3ST4,HS6ST2,WSCD1	
186	Pancreatic Secretion Signaling Pathway	9.21E-01	1.69E-02	1	ADCY2,CA10,PRKCA,PRKCD	
187	Processing of SMDT1	9.20E-01	6.25E-02	NaN	MCUB	
188	Granzyme B Signaling	9.20E-01	6.25E-02	NaN	PARP1	
189	D-myo-inositol (1,4,5)-trisphosphate Degradation	9.20E-01	6.25E-02	NaN	INPP5A	
190	Chemokine Signaling	9.20E-01	2.67E-02	NaN	PRKCA,RASD1	
191	Glucocorticoid Receptor Signaling	9.19E-01	1.35E-02	NaN	B2M,Hspa1b,NR3C1,NR3C2,PLA2G6,RASD1,SDHD	
192	Actin Cytoskeleton Signaling	9.16E-01	1.69E-02	NaN	ARPC2,BCAR1,ITGA7,RASD1	
193	Sertoli Cell-Sertoli Cell Junction Signaling	9.16E-01	1.69E-02	NaN	CDH9,KEAP1,RASD1,Tubb2b	
194	Type II Diabetes Mellitus Signaling	9.16E-01	1.97E-02	NaN	ACSL4,PRKCA,PRKCD	
195	GDNF Family Ligand-Receptor Interactions	9.11E-01	2.63E-02	NaN	GFRA2,RASD1	
196	Epithelial Adherens Junction Signaling	8.97E-01	1.94E-02	NaN	ARPC2,PPP2R5E,RASD1	
197	Gastrin-CREB signalling pathway via PKC and MAPK	8.96E-01	5.88E-02	NaN	PRKCA	
198	Paracetamol ADME	8.96E-01	5.88E-02	NaN	GGT7	
199	1D-myo-inositol Hexakisphosphate Biosynthesis II (Mammalian)	8.96E-01	5.88E-02	NaN	INPP5A	
200	D-myo-inositol (1,3,4)-trisphosphate Biosynthesis	8.96E-01	5.88E-02	NaN	INPP5A	
201	Phase I - Functionalization of compounds	8.83E-01	2.53E-02	NaN	CYP26B1,CYP7B1	
202	Class I peroxisomal membrane protein import	8.51E-01	5.26E-02	NaN	ABCD3	
203	Maturation of TCA enzymes and regulation of TCA cycle	8.51E-01	5.26E-02	NaN	SDHD	
204	Œ≥-linolenate Biosynthesis II (Animals)	8.51E-01	5.26E-02	NaN	ACSL4	

	A	B	C	D	E	F
205	Mitochondrial L-carnitine Shuttle Pathway	8.51E-01	5.26E-02	NaN	ACSL4	
206	Mitochondrial Division Signaling Pathway	8.45E-01	1.83E-02	NaN	PPP2R5E,PRKCD,RASD1	
207	Phagosome Formation	8.39E-01	1.24E-02	-1.414	ARPC2,BCAR1,ITGA7,MARCKS,PLA2G6,PRKCA,PRKCD,RASD1	
208	Natural Killer Cell Signaling	8.34E-01	1.81E-02	NaN	B2M,Hspa1b,RASD1	
209	Signaling by NODAL	8.30E-01	5.00E-02	NaN	FURIN	
210	Receptor-type tyrosine-protein phosphatases	8.30E-01	5.00E-02	NaN	PPFIA2	
211	Interleukin-12 family signaling	8.30E-01	5.00E-02	NaN	CRLF1	
212	Hepatitis B Chronic Liver Pathogenesis Signaling Pathway	8.28E-01	1.80E-02	NaN	PRKCA,PRKCD,RASD1	
213	GŒ±q Signaling	8.28E-01	1.80E-02	NaN	PRKCA,PRKCD,RGS4	
214	PDGF Signaling	8.24E-01	2.33E-02	NaN	PRKCA,RASD1	
215	Erythropoietin Signaling Pathway	8.23E-01	1.79E-02	NaN	PRKCA,PRKCD,RASD1	
216	Aldosterone Signaling in Epithelial Cells	8.23E-01	1.79E-02	NaN	NR3C2,PRKCA,PRKCD	
217	Glioblastoma Multiforme Signaling	8.17E-01	1.78E-02	NaN	PRKCD,RASD1,WNT7B	
218	BMP signaling pathway	8.16E-01	2.30E-02	NaN	BMP3,RASD1	
219	Citric acid cycle (TCA cycle)	8.11E-01	4.76E-02	NaN	SDHD	
220	Formation of the ureteric bud	8.11E-01	4.76E-02	NaN	SLIT2	
221	TCA Cycle II (Eukaryotic)	8.11E-01	4.76E-02	NaN	SDHD	
222	Regulation of Cellular Mechanics by Calpain Protease	8.08E-01	2.27E-02	NaN	ITGA7,RASD1	
223	Netrin Signaling	8.01E-01	1.74E-02	NaN	ARPC2,PRKCA,PRKCD	
224	Regulation of mRNA stability by proteins that bind AU-rich elemer	8.01E-01	2.25E-02	NaN	PRKCA,PRKCD	
225	Unfolded protein response	8.01E-01	2.25E-02	NaN	CEBPD,Hspa1b	
226	Signaling by Rho Family GTPases	8.00E-01	1.52E-02	NaN	ARPC2,CDH9,ITGA7,STMN1	
227	Keratinization	7.93E-01	2.22E-02	NaN	FURIN,PKP2	
228	Ceramide Signaling	7.93E-01	2.22E-02	NaN	PPP2R5E,RASD1	
229	Pyrimidine Deoxyribonucleotides De Novo Biosynthesis I	7.92E-01	4.55E-02	NaN	AK4	
230	Superpathway of D-myo-inositol (1,4,5)-trisphosphate Metabolism	7.92E-01	4.55E-02	NaN	INPP5A	
231	Ion channel transport	7.91E-01	1.72E-02	NaN	ATP11B,FKBP1B,FXYD6	
232	EPH-Ephrin signaling	7.86E-01	2.20E-02	NaN	ARPC2,EPHB1	
233	Acute Myeloid Leukemia Signaling	7.86E-01	2.20E-02	NaN	KIT,RASD1	
234	Acute Phase Response Signaling	7.81E-01	1.70E-02	NaN	NR3C1,RASD1,TTR	
235	Salvage Pathways of Pyrimidine Ribonucleotides	7.78E-01	2.17E-02	NaN	AK4,PRKCD	
236	RAF-independent MAPK1/3 activation	7.74E-01	4.35E-02	NaN	DUSP5	
237	Regulation of eIF4 and p70S6K Signaling	7.71E-01	1.69E-02	NaN	ITGA7,PPP2R5E,RASD1	
238	Non-Small Cell Lung Cancer Signaling	7.64E-01	2.13E-02	NaN	PRKCA,RASD1	
239	TCF dependent signaling in response to WNT	7.61E-01	1.67E-02	NaN	MEN1,PPP2R5E,RSPO2	
240	RHO GTPases activate IQGAPs	7.58E-01	4.17E-02	NaN	MEN1	
241	Interleukin-6 family signaling	7.58E-01	4.17E-02	NaN	CRLF1	
242	Glycogen metabolism	7.58E-01	4.17E-02	NaN	PYGB	
243	Huntington's Disease Signaling	7.52E-01	1.45E-02	NaN	Hspa1b,PRKCA,PRKCD,SDHD	
244	VEGF Signaling	7.50E-01	2.08E-02	NaN	PRKCA,RASD1	
245	Apelin Cardiomyocyte Signaling Pathway	7.43E-01	2.06E-02	NaN	PRKCA,PRKCD	
246	Production of Nitric Oxide and Reactive Oxygen Species in Macrop	7.41E-01	1.63E-02	NaN	PPP2R5E,PRKCA,PRKCD	
247	Gustation Pathway	7.41E-01	1.63E-02	NaN	ADCY2,GABRB1,SCN3B	
248	CLEAR Signaling Pathway	7.29E-01	1.42E-02	1	PPP2R5E,PRKCA,PRKCD,RASD1	
249	Leukocyte Extravasation Signaling	7.27E-01	1.60E-02	NaN	BCAR1,PRKCA,PRKCD	
250	WNT ligand biogenesis and trafficking	7.26E-01	3.85E-02	NaN	WNT7B	
251	Antigen Presentation Pathway	7.26E-01	3.85E-02	NaN	B2M	
252	Neuropathic Pain Signaling in Dorsal Horn Neurons	7.22E-01	2.00E-02	NaN	PRKCA,PRKCD	
253	PPARŒ±/RXRŒ± Activation	7.18E-01	1.59E-02	NaN	ADCY2,PRKCA,RASD1	
254	Neuroprotective Role of THOP1 in Alzheimer's Disease	7.16E-01	1.98E-02	NaN	FURIN,PRSS23	
255	Generic Transcription Pathway	7.14E-01	1.58E-02	NaN	NR3C1,NR3C2,NR4A2	
256	Syndecan interactions	7.12E-01	3.70E-02	NaN	PRKCA	
257	Integrin signaling	7.12E-01	3.70E-02	NaN	BCAR1	
258	Interconversion of nucleotide di- and triphosphates	7.12E-01	3.70E-02	NaN	AK4	
259	Sleep REM Signaling Pathway	7.09E-01	1.96E-02	NaN	HOMER1,NR4A2	
260	Potassium Channels	7.03E-01	1.94E-02	NaN	KCNA2,KCNA6	
261	Extracellular matrix organization	6.97E-01	1.92E-02	NaN	ITGA7,TTR	
262	Cargo recognition for clathrin-mediated endocytosis	6.97E-01	1.92E-02	NaN	ARRB2,REPS2	
263	Telomerase Signaling	6.84E-01	1.89E-02	NaN	PPP2R5E,RASD1	
264	FOXO-mediated transcription of oxidative stress, metabolic and n	6.84E-01	3.45E-02	NaN	NR3C1	
265	Sonic Hedgehog Signaling	6.84E-01	3.45E-02	NaN	ARRB2	
266	PI3K/AKT Signaling	6.83E-01	1.52E-02	NaN	ITGA7,PPP2R5E,RASD1	
267	Protein Kinase A Signaling	6.80E-01	1.26E-02	0.447	ADCY2,DUSP5,PRKCA,PRKCD,PYGB	
268	Regulation of Actin-based Motility by Rho	6.78E-01	1.87E-02	NaN	ARPC2,ITGA7	
269	Nicotinate metabolism	6.71E-01	3.33E-02	NaN	NAXE	
270	DAP12 interactions	6.71E-01	3.33E-02	NaN	B2M	
271	Activation of kainate receptors upon glutamate binding	6.71E-01	3.33E-02	NaN	NCALD	
272	O-linked glycosylation	6.66E-01	1.83E-02	NaN	GALNT17,ST3GAL2	
273	Glutamate binding, activation of AMPA receptors and synaptic pla	6.58E-01	3.23E-02	NaN	PRKCA	
274	Thrombin signalling through proteinase activated receptors (PARs	6.46E-01	3.12E-02	NaN	ARRB2	
275	Sialic acid metabolism	6.46E-01	3.12E-02	NaN	ST3GAL2	
276	PAK Signaling	6.32E-01	1.74E-02	NaN	ITGA7,RASD1	
277	RAR Activation	6.32E-01	1.21E-02	0.447	ADCY2,KIT,PARP1,PRKCD,PRMT2	
278	Cohesin Chromatin Regulation Pathway	6.26E-01	1.42E-02	NaN	PCDH7,PCDH9,PPP2R5E	
279	Transcriptional Regulation by E2F6	6.23E-01	2.94E-02	NaN	YAF2	
280	Transcriptional Regulation by NPAS4	6.23E-01	2.94E-02	NaN	NR3C1	
281	Fatty Acid Œ≤-oxidation I	6.23E-01	2.94E-02	NaN	ACSL4	
282	Nitric Oxide Signaling in the Cardiovascular System	6.22E-01	1.71E-02	NaN	PRKCA,PRKCD	
283	Cardiac Hypertrophy Signaling (Enhanced)	6.12E-01	1.14E-02	1	ADCY2,ITGA7,PRKCA,PRKCD,RASD1,WNT7B	
284	Transcriptional regulation by the AP-2 (TFAP2) family of transcript	6.12E-01	2.86E-02	NaN	KIT	
285	Fatty acyl-CoA biosynthesis	6.01E-01	2.78E-02	NaN	ACSL4	
286	NGF Signaling	6.01E-01	1.65E-02	NaN	PRKCD,RASD1	
287	Neurovascular Coupling Signaling Pathway	5.95E-01	1.37E-02	NaN	DAGLA,GABRB1,PLA2G6	
288	Cell Cycle Regulation by BTG Family Proteins	5.91E-01	2.70E-02	NaN	PPP2R5E	
289	Response to elevated platelet cytosolic Ca2+	5.86E-01	1.61E-02	NaN	PCDH7,PRKCA	
290	TP53 Regulates Transcription of Cell Death Genes	5.81E-01	2.63E-02	NaN	PPP1R13B	
291	Resolution of Abasic Sites (AP sites)	5.81E-01	2.63E-02	NaN	PARP1	
292	Notch Signaling	5.81E-01	2.63E-02	NaN	FURIN	
293	GP6 Signaling Pathway	5.81E-01	1.60E-02	NaN	PRKCA,PRKCD	
294	Endocannabinoid Developing Neuron Pathway	5.76E-01	1.59E-02	NaN	ADCY2,RASD1	
295	Signaling by Retinoic Acid	5.72E-01	2.56E-02	NaN	CYP26B1	
296	Pyrimidine Ribonucleotides Interconversion	5.72E-01	2.56E-02	NaN	AK4	
297	HER-2 Signaling in Breast Cancer	5.70E-01	1.33E-02	NaN	PRKCA,PRKCD,RASD1	
298	Retinoid metabolism and transport	5.62E-01	2.50E-02	NaN	TTR	
299	GŒ±12/13 Signaling	5.57E-01	1.54E-02	NaN	CDH9,RASD1	
300	Oncostatin M Signaling	5.53E-01	2.44E-02	NaN	RASD1	
301	GABA Receptor Signaling	5.52E-01	1.53E-02	NaN	ADCY2,GABRB1	
302	SNARE Signaling Pathway	5.48E-01	1.52E-02	NaN	ADCY2,SYT12	
303	Elastic fibre formation	5.44E-01	2.38E-02	NaN	FURIN	
304	Formation of WDR5-containing histone-modifying complexes	5.44E-01	2.38E-02	NaN	MEN1	
305	Pyrimidine Ribonucleotides De Novo Biosynthesis	5.44E-01	2.38E-02	NaN	AK4	
306	GABAergic Receptor Signaling Pathway (Enhanced)	5.39E-01	1.49E-02	NaN	ADCY2,GABRB1	

	A	B	C	D	E	F
307	Cyclophilin Signaling Pathway	5.37E-01	1.27E-02	NaN	B2M,PPIB,WNT7B	
308	MIF Regulation of Innate Immunity	5.36E-01	2.33E-02	NaN	PLA2G6	
309	BER (Base Excision Repair) Pathway	5.36E-01	2.33E-02	NaN	PARP1	
310	G-Protein Coupled Receptor Signaling	5.32E-01	1.04E-02	-1.89	ADCY2,ARRB2,PRKCA,RASD1,RGS12,RGS14,RGS4	
311	Retinoic acid Mediated Apoptosis Signaling	5.27E-01	2.27E-02	NaN	PARP1	
312	G alpha (s) signalling events	5.22E-01	1.45E-02	NaN	ADCY2,ARRB2	
313	Breast Cancer Regulation by Stathmin1	5.21E-01	1.05E-02	0.447	PPP2R5E,PRKCA,PRKCD,RASD1,STMN1,Tubb2b	
314	Reelin Signaling in Neurons	5.13E-01	1.43E-02	NaN	ARHGEF26,ARPC2	
315	Role of OCT4 in Mammalian Embryonic Stem Cell Pluripotency	5.11E-01	2.17E-02	NaN	PARP1	
316	Neutrophil Extracellular Trap Signaling Pathway	5.06E-01	1.13E-02	-1	PLA2G6,PRKCA,PRKCD,SDHD	
317	Role of Pattern Recognition Receptors in Recognition of Bacteria a	5.05E-01	1.41E-02	NaN	PRKCA,PRKCD	
318	Inositol phosphate metabolism	5.04E-01	2.13E-02	NaN	INPP5A	
319	Necroptosis Signaling Pathway	4.97E-01	1.39E-02	NaN	PLA2G6,TYRO3	
320	PIP3 activates AKT signaling	4.89E-01	1.37E-02	NaN	KIT,PPP2R5E	
321	PXR/RXR Activation	4.89E-01	2.04E-02	NaN	NR3C1	
322	Melanoma Signaling	4.89E-01	2.04E-02	NaN	RASD1	
323	nNOS Signaling in Skeletal Muscle Cells	4.89E-01	2.04E-02	NaN	SNTA1	
324	KEAP1-NFE2L2 pathway	4.85E-01	1.36E-02	NaN	KEAP1,PRKCD	
325	Apoptotic execution phase	4.82E-01	2.00E-02	NaN	PRKCD	
326	Signaling by TGFBR3	4.82E-01	2.00E-02	NaN	ARRB2	
327	Endocannabinoid Neuronal Synapse Pathway	4.81E-01	1.35E-02	NaN	ADCY2,DAGLA	
328	Transcriptional and post-translational regulation of MITF-M expre	4.75E-01	1.96E-02	NaN	KIT	
329	Intrinsic Pathway for Apoptosis	4.61E-01	1.89E-02	NaN	PPP1R13B	
330	Ovarian Cancer Signaling	4.55E-01	1.29E-02	NaN	RASD1,WNT7B	
331	Signaling by TGF-beta Receptor Complex	4.55E-01	1.85E-02	NaN	FURIN	
332	Activation of IRF by Cytosolic Pattern Recognition Receptors	4.55E-01	1.85E-02	NaN	PPIB	
333	Colorectal Cancer Metastasis Signaling	4.54E-01	1.14E-02	NaN	ADCY2,RASD1,WNT7B	
334	Triacylglycerol Degradation	4.48E-01	1.82E-02	NaN	DAGLA	
335	EGF Signaling	4.48E-01	1.82E-02	NaN	PRKCA	
336	SPINK1 General Cancer Pathway	4.48E-01	1.82E-02	NaN	RASD1	
337	CNTF Signaling	4.42E-01	1.79E-02	NaN	RASD1	
338	Phospholipase C Signaling	4.37E-01	9.56E-03	0	ADCY2,ITGA7,MARCKS,PLA2G6,PRKCA,PRKCD,RASD1	
339	Circadian Clock	4.36E-01	1.75E-02	NaN	NR3C1	
340	Gamma carboxylation, hypusinylation, hydroxylation, and arylsulf	4.36E-01	1.75E-02	NaN	FURIN	
341	Role of CHK Proteins in Cell Cycle Checkpoint Control	4.36E-01	1.75E-02	NaN	PPP2R5E	
342	Cancer Drug Resistance by Drug Efflux	4.30E-01	1.72E-02	NaN	RASD1	
343	Transcriptional Regulatory Network in Embryonic Stem Cells	4.25E-01	1.22E-02	NaN	RASD1,WNT7B	
344	p70S6K Signaling	4.22E-01	1.02E-02	NaN	PPP2R5E,PRKCA,PRKCD,RASD1	
345	Collagen degradation	4.18E-01	1.67E-02	NaN	FURIN	
346	Endometrial Cancer Signaling	4.18E-01	1.67E-02	NaN	RASD1	
347	PCP (Planar Cell Polarity) Pathway	4.18E-01	1.67E-02	NaN	WNT7B	
348	Androgen Signaling	4.15E-01	1.20E-02	NaN	PRKCA,PRKCD	
349	rRNA modification in the nucleus and cytosol	4.13E-01	1.64E-02	NaN	FCF1	
350	IL-2 Signaling	4.13E-01	1.64E-02	NaN	RASD1	
351	Pre-NOTCH Expression and Processing	4.07E-01	1.61E-02	NaN	FURIN	
352	WNT/Œ≤-catenin Signaling	4.05E-01	1.18E-02	NaN	PPP2R5E,WNT7B	
353	NCAM signaling for neurite out-growth	4.02E-01	1.59E-02	NaN	GFRA2	
354	Phospholipases	4.02E-01	1.59E-02	NaN	PLA2G6	
355	Stearate Biosynthesis I (Animals)	4.02E-01	1.59E-02	NaN	ACSL4	
356	Neuroinflammation Signaling Pathway	3.97E-01	1.05E-02	NaN	B2M,GABRB1,PLA2G6	
357	ERB2-ERBB3 Signaling	3.97E-01	1.56E-02	NaN	RASD1	
358	Senescence Pathway	3.94E-01	1.04E-02	NaN	PARP1,PPP2R5E,RASD1	
359	Phase II - Conjugation of compounds	3.92E-01	1.54E-02	NaN	GGT7	
360	Role of WNT/GSK-3Œ≤ Signaling in the Pathogenesis of Influenza	3.92E-01	1.54E-02	NaN	WNT7B	
361	Pyridoxal 5'-phosphate Salvage Pathway	3.92E-01	1.54E-02	NaN	PRKCD	
362	Collagen biosynthesis and modifying enzymes	3.87E-01	1.52E-02	NaN	PPIB	
363	Mitotic Roles of Polo-Like Kinase	3.87E-01	1.52E-02	NaN	PPP2R5E	
364	WNT/Ca+ pathway	3.87E-01	1.52E-02	NaN	PRKCA	
365	Cardiac Œ≤-adrenergic Signaling	3.84E-01	1.13E-02	NaN	ADCY2,PPP2R5E	
366	Autism Signaling Pathway	3.83E-01	1.02E-02	NaN	B2M,RASD1,WNT7B	
367	Agrin Interactions at Neuromuscular Junction	3.77E-01	1.47E-02	NaN	RASD1	
368	Role of JAK1 and JAK3 in Œ≥c Cytokine Signaling	3.77E-01	1.47E-02	NaN	RASD1	
369	GM-CSF Signaling	3.77E-01	1.47E-02	NaN	RASD1	
370	Sensory processing of sound by inner hair cells of the cochlea	3.72E-01	1.45E-02	NaN	CABP1	
371	Glioma Invasiveness Signaling	3.67E-01	1.43E-02	NaN	RASD1	
372	Granzyme A Signaling	3.67E-01	1.43E-02	NaN	PARP1	
373	NUR77 Signaling in T Lymphocytes	3.64E-01	9.93E-03	NaN	B2M,PRKCA,PRKCD	
374	Regulation of RUNX2 expression and activity	3.58E-01	1.39E-02	NaN	NR3C1	
375	Role of MAPK Signaling in Inhibiting the Pathogenesis of Influenza	3.58E-01	1.39E-02	NaN	PLA2G6	
376	Plasma lipoprotein assembly, remodeling, and clearance	3.54E-01	1.37E-02	NaN	FURIN	
377	ERK5 Signaling	3.54E-01	1.37E-02	NaN	RASD1	
378	Ephrin B Signaling	3.54E-01	1.37E-02	NaN	EPHB1	
379	Immunoregulatory interactions between a Lymphoid and a non-Ly	3.49E-01	1.35E-02	NaN	B2M	
380	SUMOylation of DNA damage response and repair proteins	3.49E-01	1.35E-02	NaN	PARP1	
381	GPCR-Mediated Integration of Enteroendocrine Signaling Exempli	3.49E-01	1.35E-02	NaN	ADCY2	
382	Intra-Golgi and retrograde Golgi-to-ER traffic	3.49E-01	1.05E-02	NaN	PLA2G6,RAB18	
383	Signaling by NOTCH1	3.45E-01	1.33E-02	NaN	ARRB2	
384	Leptin Signaling in Obesity	3.45E-01	1.33E-02	NaN	ADCY2	
385	Angiopoietin Signaling	3.41E-01	1.32E-02	NaN	RASD1	
386	Role of JAK family kinases in IL-6-type Cytokine Signaling	3.41E-01	1.32E-02	NaN	CEBPD	
387	CDX Gastrointestinal Cancer Signaling Pathway	3.41E-01	1.04E-02	NaN	BMP3,WNT7B	
388	Regulation of the Epithelial-Mesenchymal Transition Pathway	3.38E-01	1.03E-02	NaN	RASD1,WNT7B	
389	Antiproliferative Role of Somatostatin Receptor 2	3.37E-01	1.30E-02	NaN	RASD1	
390	Thyroid Cancer Signaling	3.37E-01	1.30E-02	NaN	RASD1	
391	Cilium Assembly	3.33E-01	1.02E-02	NaN	AHI1,ASAP1	
392	Transport of bile salts and organic acids, metal ions and amine co	3.33E-01	1.28E-02	NaN	SLC18A2	
393	Renal Cell Carcinoma Signaling	3.33E-01	1.28E-02	NaN	RASD1	
394	Neurotrophin/TRK Signaling	3.33E-01	1.28E-02	NaN	RASD1	
395	Adrenomedullin signaling pathway	3.31E-01	1.02E-02	NaN	ADCY2,RASD1	
396	G alpha (12/13) signalling events	3.29E-01	1.27E-02	NaN	ARHGEF26	
397	RHO GTPase cycle	3.28E-01	9.05E-03	0	ABCD3,ARHGEF26,PCDH7,WIPF3	
398	Post-translational modification: synthesis of GPI-anchored protei	3.25E-01	1.25E-02	NaN	RTN4RL1	
399	Estrogen-Dependent Breast Cancer Signaling	3.25E-01	1.25E-02	NaN	RASD1	
400	BEX2 Signaling Pathway	3.25E-01	1.25E-02	NaN	PPP2R5E	
401	Myelination Signaling Pathway	3.22E-01	9.29E-03	NaN	BMP3,RASD1,WNT7B	
402	Clathrin-mediated Endocytosis Signaling	3.21E-01	9.95E-03	NaN	ARPC2,ARRB2	
403	FLT3 Signaling in Hematopoietic Progenitor Cells	3.21E-01	1.23E-02	NaN	RASD1	
404	BAG2 Signaling Pathway	3.21E-01	1.23E-02	NaN	Hspa1b	
405	Beta-catenin independent WNT signaling	3.19E-01	9.90E-03	NaN	ARRB2,PRKCA	
406	Immunogenic Cell Death Signaling Pathway	3.17E-01	1.22E-02	NaN	Hspa1b	
407	Transcriptional regulation of white adipocyte differentiation	3.17E-01	1.22E-02	NaN	CEBPD	
408	JAK/STAT Signaling	3.17E-01	1.22E-02	NaN	RASD1	

	A	B	C	D	E	F
409	Cyclins and Cell Cycle Regulation	3.14E-01	1.20E-02	NaN	PPP2R5E	
410	Integrin cell surface interactions	3.10E-01	1.19E-02	NaN	ITGA7	
411	PEDF Signaling	3.10E-01	1.19E-02	NaN	RASD1	
412	IL-12 Signaling and Production in Macrophages	3.10E-01	9.71E-03	NaN	PRKCA,PRKCD	
413	Platelet homeostasis	3.06E-01	1.18E-02	NaN	PPP2R5E	
414	Regulation of the Epithelial Mesenchymal Transition in Developm	3.06E-01	1.18E-02	NaN	WNT7B	
415	HIPPO signaling	3.03E-01	1.16E-02	NaN	PPP2R5E	
416	FGF Signaling	3.03E-01	1.16E-02	NaN	PRKCA	
417	Nuclear Cytoskeleton Signaling Pathway	3.01E-01	9.52E-03	NaN	CDH9,ITGA7	
418	Mitotic Prophase	2.99E-01	1.15E-02	NaN	PRKCA	
419	Respiratory electron transport	2.99E-01	1.15E-02	NaN	SDHD	
420	Apelin Adipocyte Signaling Pathway	2.99E-01	1.15E-02	NaN	ADCY2	
421	RAB GEFs exchange GTP for GDP on RABs	2.92E-01	1.12E-02	NaN	RAB18	
422	Degradation of beta-catenin by the destruction complex	2.89E-01	1.11E-02	NaN	PPP2R5E	
423	Cell junction organization	2.89E-01	1.11E-02	NaN	CDH9	
424	Death Receptor Signaling	2.79E-01	1.08E-02	NaN	PARP1	
425	Role of Osteoblasts, Osteoclasts and Chondrocytes in Rheumatoid	2.78E-01	9.05E-03	NaN	BMP3,WNT7B	
426	Class B/2 (Secretin family receptors)	2.76E-01	1.06E-02	NaN	WNT7B	
427	p53 Signaling	2.73E-01	1.05E-02	NaN	PPP1R13B	
428	PD-1, PD-L1 cancer immunotherapy pathway	2.73E-01	1.05E-02	NaN	B2M	
429	ABC-family proteins mediated transport	2.70E-01	1.04E-02	NaN	ABCD3	
430	IL-1 Signaling	2.70E-01	1.04E-02	NaN	ADCY2	
431	ATM Signaling	2.67E-01	1.03E-02	NaN	PPP2R5E	
432	TGF-Œ≤ Signaling	2.67E-01	1.03E-02	NaN	RASD1	
433	Sumoylation Pathway	2.64E-01	1.02E-02	NaN	NR3C1	
434	GPER1 signaling	2.61E-01	1.01E-02	NaN	ADCY2	
435	CDC42 Signaling	2.59E-01	8.31E-03	NaN	ARPC2,B2M,ITGA7	
436	IL-13 Signaling Pathway	2.59E-01	1.00E-02	NaN	PRKCD	
437	Role of Osteoblasts in Rheumatoid Arthritis Signaling Pathway	2.57E-01	8.62E-03	NaN	BMP3,WNT7B	
438	Sphingolipid metabolism	2.50E-01	9.71E-03	NaN	ST3GAL2	
439	Interleukin-4 and Interleukin-13 signaling	2.50E-01	9.71E-03	NaN	CEBPD	
440	Mouse Embryonic Stem Cell Pluripotency	2.50E-01	9.71E-03	NaN	RASD1	
441	IGF-1 Signaling	2.50E-01	9.71E-03	NaN	RASD1	
442	MSP-RON Signaling in Macrophages Pathway	2.47E-01	9.62E-03	NaN	RASD1	
443	PPAR Signaling	2.45E-01	9.52E-03	NaN	RASD1	
444	AMPK Signaling	2.43E-01	8.33E-03	NaN	AK4,PPP2R5E	
445	Deubiquitination	2.41E-01	8.30E-03	NaN	ARRB2,KEAP1	
446	Oxidative Phosphorylation	2.40E-01	9.35E-03	NaN	SDHD	
447	MHC class II antigen presentation	2.37E-01	9.26E-03	NaN	OSBPL1A	
448	Nucleotide Excision Repair	2.37E-01	9.26E-03	NaN	PARP1	
449	Antioxidant Action of Vitamin C	2.37E-01	9.26E-03	NaN	PLA2G6	
450	Kinetochore Metaphase Signaling Pathway	2.37E-01	9.26E-03	NaN	PPP2R5E	
451	Wound Healing Signaling Pathway	2.36E-01	8.20E-03	NaN	PRKCA,RASD1	
452	Hedgehog 'off' state	2.35E-01	9.17E-03	NaN	ADCY2	
453	Irritable Bowel Syndrome Signaling Pathway	2.28E-01	8.03E-03	NaN	ARPC2,WNT7B	
454	Costimulation by the CD28 family	2.27E-01	8.93E-03	NaN	PPP2R5E	
455	Sphingosine-1-phosphate Signaling	2.27E-01	8.93E-03	NaN	ADCY2	
456	Prostate Cancer Signaling	2.25E-01	8.85E-03	NaN	RASD1	
457	Bladder Cancer Signaling	2.25E-01	8.85E-03	NaN	RASD1	
458	LXR/RXR Activation	2.22E-01	8.77E-03	NaN	TTR	
459	p38 MAPK Signaling	2.18E-01	8.62E-03	NaN	PLA2G6	
460	Atherosclerosis Signaling	2.16E-01	8.55E-03	NaN	PLA2G6	
461	Role of PKR in Interferon Induction and Antiviral Response	2.09E-01	8.33E-03	NaN	Hspa1b	
462	RHOA Signaling	2.09E-01	8.33E-03	NaN	ARPC2	
463	SRP-dependent cotranslational protein targeting to membrane	2.03E-01	8.13E-03	NaN	SRP14	
464	IL-27 Signaling Pathway	2.03E-01	8.13E-03	NaN	B2M	
465	GŒ±s Signaling	2.01E-01	8.06E-03	NaN	ADCY2	
466	IL-6 Signaling	2.01E-01	8.06E-03	NaN	RASD1	
467	Dilated Cardiomyopathy Signaling Pathway	0.00E+00	6.80E-03	NaN	ADCY2	
468	NAD Signaling Pathway	0.00E+00	7.09E-03	NaN	PARP1	
469	Pulmonary Fibrosis Idiopathic Signaling Pathway	0.00E+00	6.23E-03	NaN	RASD1,WNT7B	
470	ID1 Signaling Pathway	0.00E+00	5.03E-03	NaN	RASD1	
471	Macrophage Classical Activation Signaling Pathway	0.00E+00	6.41E-03	NaN	SDHD	
472	MicroRNA Biogenesis Signaling Pathway	0.00E+00	5.56E-03	NaN	RASD1	
473	Multiple Sclerosis Signaling Pathway	0.00E+00	5.24E-03	NaN	PARP1	
474	Role of Chondrocytes in Rheumatoid Arthritis Signaling Pathway	0.00E+00	7.35E-03	NaN	CEBPD	
475	Role of Osteoclasts in Rheumatoid Arthritis Signaling Pathway	0.00E+00	3.31E-03	NaN	RASD1	
476	Ribonucleotide Reductase Signaling Pathway	0.00E+00	6.17E-03	NaN	PARP1	
477	Macrophage Alternative Activation Signaling Pathway	0.00E+00	6.13E-03	NaN	NR3C1	
478	S100 Family Signaling Pathway	0.00E+00	7.01E-03	1.342	NR3C1,PLA2G6,PRKCA,PRKCD,WNT7B	
479	Chaperone Mediated Autophagy Signaling Pathway	0.00E+00	2.30E-03	NaN	Hspa1b	
480	IL-33 Signaling Pathway	0.00E+00	6.25E-03	NaN	KIT	
481	NOD1/2 Signaling Pathway	0.00E+00	6.10E-03	NaN	Hspa1b	
482	p75 NTR receptor-mediated signalling	0.00E+00	6.58E-03	NaN	ARHGEF26	
483	Mitotic Metaphase and Anaphase	0.00E+00	4.46E-03	NaN	PPP2R5E	
484	G alpha (q) signalling events	0.00E+00	6.10E-03	NaN	RGS4	
485	C-type lectin receptors (CLRs)	0.00E+00	7.46E-03	NaN	PRKCD	
486	Hedgehog 'on' state	0.00E+00	7.09E-03	NaN	ARRB2	
487	RHO GTPases Activate Formins	0.00E+00	7.63E-03	NaN	PPP2R5E	
488	Major pathway of rRNA processing in the nucleolus and cytosol	0.00E+00	5.59E-03	NaN	FCF1	
489	Mitotic Prometaphase	0.00E+00	5.18E-03	NaN	PPP2R5E	
490	Cell Cycle Checkpoints	0.00E+00	3.88E-03	NaN	PPP2R5E	
491	Transcriptional regulation by RUNX1	0.00E+00	7.35E-03	NaN	YAF2	
492	Neddylation	0.00E+00	4.13E-03	NaN	KEAP1	
493	Class I MHC mediated antigen processing and presentation	0.00E+00	5.46E-03	NaN	B2M,KEAP1	
494	Mitochondrial Dysfunction	0.00E+00	2.99E-03	NaN	SDHD	
495	CGAS-STING Signaling Pathway	0.00E+00	7.94E-03	NaN	PCDH7	
496	NAFLD Signaling Pathway	0.00E+00	4.63E-03	NaN	PPP2R5E	
497	Protein Sorting Signaling Pathway	0.00E+00	5.65E-03	NaN	ADCY2	
498	BBSome Signaling Pathway	0.00E+00	4.33E-03	NaN	ARRB2,PRKCD	
499	HEY1 Signaling Pathway	0.00E+00	6.37E-03	NaN	BMP3	
500	Lung Ionic Balance Signaling Pathway	0.00E+00	6.17E-03	NaN	PPP2R5E,PRKCA,PRKCD	
501	Tight Junction Signaling	0.00E+00	5.75E-03	NaN	PPP2R5E	
502	TR/RXR Activation	0.00E+00	7.94E-03	NaN	SYT12	
503	Role of NFAT in Regulation of the Immune Response	0.00E+00	1.54E-03	NaN	RASD1	
504	FcŒ≥RIIB Signaling in B Lymphocytes	0.00E+00	2.92E-03	NaN	RASD1	
505	CCR5 Signaling in Macrophages	0.00E+00	6.47E-03	NaN	PRKCA,PRKCD	
506	Calcium-induced T Lymphocyte Apoptosis	0.00E+00	7.66E-03	NaN	PRKCA,PRKCD	
507	Cytotoxic T Lymphocyte-mediated Apoptosis of Target Cells	0.00E+00	4.26E-03	NaN	B2M	
508	IL-17 Signaling	0.00E+00	6.25E-03	NaN	RASD1	
509	CTLA4 Signaling in Cytotoxic T Lymphocytes	0.00E+00	7.58E-03	NaN	B2M,PPP2R5E,RASD1	
510	CD28 Signaling in T Helper Cells	0.00E+00	3.13E-03	NaN	ARPC2	

	A	B	C	D	E	F
511	IL-15 Signaling	0.00E+00	2.95E-03	NaN	RASD1	
512	Dendritic Cell Maturation	0.00E+00	2.66E-03	NaN	B2M	
513	Cellular Effects of Sildenafil (Viagra)	0.00E+00	3.05E-03	NaN	ADCY2,PPP2R5E	
514	Relaxin Signaling	0.00E+00	6.54E-03	NaN	ADCY2	
515	Cardiac Hypertrophy Signaling	0.00E+00	7.94E-03	NaN	ADCY2,RASD1	
516	Molecular Mechanisms of Cancer	0.00E+00	7.31E-03	0	ADCY2,BMP3,ITGA7,PRKCA,PRKCD,RASD1	
517	Lipid Antigen Presentation by CD1	0.00E+00	4.48E-03	NaN	B2M	
518	HMGB1 Signaling	0.00E+00	6.41E-03	NaN	RASD1	
519	CREB Signaling in Neurons	0.00E+00	6.85E-03	0	ADCY2,PRKCA,PRKCD,RASD1	
520	Allograft Rejection Signaling	0.00E+00	3.73E-03	NaN	B2M	
521	Chronic Myeloid Leukemia Signaling	0.00E+00	7.27E-03	NaN	RASD1,WNT7B	
522	Communication between Innate and Adaptive Immune Cells	0.00E+00	1.85E-03	NaN	B2M	
523	Systemic Lupus Erythematosus Signaling	0.00E+00	1.56E-03	NaN	RASD1	
524	ILK Signaling	0.00E+00	5.21E-03	NaN	PPP2R5E	
525	FAK Signaling	0.00E+00	7.33E-03	-0.816	ARPC2,ASAP1,BCAR1,ITGA7,KIT,RASD1	
526	EIF2 Signaling	0.00E+00	4.61E-03	NaN	RASD1	
527	Hereditary Breast Cancer Signaling	0.00E+00	7.35E-03	NaN	RASD1	
528	Regulation of IL-2 Expression in Activated and Anergic T Lymphoc	0.00E+00	3.33E-03	NaN	RASD1	
529	PKCŒ∏ Signaling in T Lymphocytes	0.00E+00	2.79E-03	NaN	RASD1	
530	OX40 Signaling Pathway	0.00E+00	3.73E-03	NaN	B2M	
531	PI3K Signaling in B Lymphocytes	0.00E+00	2.49E-03	NaN	RASD1	
532	TEC Kinase Signaling	0.00E+00	7.79E-03	NaN	ITGA7,PRKCA,PRKCD	
533	STAT3 Pathway	0.00E+00	7.30E-03	NaN	RASD1	
534	Adipogenesis pathway	0.00E+00	7.30E-03	NaN	CEBPD	
535	Parkinson's Signaling Pathway	0.00E+00	3.47E-03	NaN	SLC18A2	
536	SAPK/JNK Signaling	0.00E+00	3.18E-03	NaN	RASD1	
537	Protein Ubiquitination Pathway	0.00E+00	3.77E-03	NaN	B2M	
538	IL-4 Signaling	0.00E+00	5.39E-03	NaN	NR3C1,RASD1	
539	B Cell Receptor Signaling	0.00E+00	2.26E-03	NaN	RASD1	
540	Insulin Receptor Signaling	0.00E+00	7.25E-03	NaN	RASD1	
541	NF-Œ∫B Signaling	0.00E+00	2.65E-03	NaN	RASD1	
542	T Cell Receptor Signaling	0.00E+00	7.37E-03	NaN	B2M,DUSP5,RASD1	
543	Phagosome Maturation	0.00E+00	6.62E-03	NaN	B2M	
544	Autophagy	0.00E+00	4.67E-03	NaN	PPP2R5E	
545	Osteoarthritis Pathway	0.00E+00	4.42E-03	NaN	ITGA7	
546	Sirtuin Signaling Pathway	0.00E+00	7.22E-03	NaN	PARP1,SDHD	
547	Iron homeostasis signaling pathway	0.00E+00	7.46E-03	NaN	BMP3	
548	Endocannabinoid Cancer Inhibition Pathway	0.00E+00	7.09E-03	NaN	ADCY2	
549	T Cell Exhaustion Signaling Pathway	0.00E+00	5.73E-03	NaN	PPP2R5E,RASD1	
550	Systemic Lupus Erythematosus in T Cell Signaling Pathway	0.00E+00	7.08E-03	NaN	B2M,PPP2R5E,RASD1	
551	Systemic Lupus Erythematosus in B Cell Signaling Pathway	0.00E+00	5.85E-03	NaN	PRKCA,PRKCD,RASD1	
552	White Adipose Tissue Browning Pathway	0.00E+00	7.30E-03	NaN	ADCY2	
553	Inhibition of ARE-Mediated mRNA Degradation Pathway	0.00E+00	6.37E-03	NaN	PPP2R5E	
554	HOTAIR Regulatory Pathway	0.00E+00	6.33E-03	NaN	WNT7B	
555	Semaphorin Neuronal Repulsive Signaling Pathway	0.00E+00	6.99E-03	NaN	ITGA7	
556	Regulation of the Epithelial Mesenchymal Transition by Growth Fa	0.00E+00	5.21E-03	NaN	RASD1	
557	Coronavirus Pathogenesis Pathway	0.00E+00	5.32E-03	NaN	FURIN	
558	Coronavirus Replication Pathway	0.00E+00	7.35E-03	NaN	Tubb2b	
559	Tumor Microenvironment Pathway	0.00E+00	5.85E-03	NaN	RASD1	
560	MSP-RON Signaling in Cancer Cells Pathway	0.00E+00	7.25E-03	NaN	RASD1	
561						

	A	B	C	D	E	F
1	**Supplementary Table 2- IPA pathways DG**					
2						
3	¬© 2000-2024 QIAGEN. All rights reserved.					
4						
5	**Ingenuity Canonical Pathways**	**-log(p-value**	**Ratio**	**z-score**	**Molecules**	
6	Axonal Guidance Signaling	5.03E+00	6.69E-02	NaN	ARPC4,BDNF,BMP1,COPS5,CXCL12,DCC,EFNA3,EPHA10,EPHA5,GNAO1,GNAZ,ITGA7,KALRN,LRRC4C,MMP17,MRPL17,NTF3,	
7					NTNG1,NTRK3,PLCH2,PLXNA2,PRKCA,PRKCB,PRKD1,RAP2B,RASD2,ROBO3,SEMA4D,SEMA7A,SLIT1,SLIT2,Tubb2b,TUBB4A	
8	Opioid Signaling Pathway	4.61E+00	7.91E-02	0.229	ADCY1,CACNB2,CACNG3,CAMK1D,GNAO1,GNAZ,GRIN3A,KCNJ3,NOS1,OPRL1,PDYN,PENK,PRKCA,PRKCB,PRKD1,RAP2B,	
9					RASD2,RGS14,RGS17,RGS4,RYR1,SRC	
10	Glutaminergic Receptor Signaling Pathway (Enhanced)	4.20E+00	7.26E-02	-1.46	ADCY1,BDNF,CACNB2,CACNG3,DGKB,DGKD,DGKH,GABRA2,GABRA5,GNAO1,GPLD1,GRIN3A,GRM2,KCNK2,NAPEPLD,	
11					PLCH2,PRKCA,PRKCB,PRKD1,SCN2B,SCN3A,SCN3B,SRC	
12	Cardiac conduction	4.09E+00	1.03E-01	0.632	AKAP9,ATP1B2,ATP2B4,CACNB2,FXYD2,FXYD6,KCNK2,NOS1,ORAI2,RYR1,SCN2B,SCN3A,SCN3B	
13	G Beta Gamma Signaling	3.40E+00	9.30E-02	0.905	ADCY1,CACNB2,CACNG3,GNAO1,GNAZ,KCNJ3,PRKCA,PRKCB,PRKD1,RAP2B,RASD2,SRC	
14	nNOS Signaling in Neurons	3.34E+00	1.46E-01	NaN	CAPN3,CAPN5,GRIN3A,NOS1,PRKCA,PRKCB,PRKD1	
15	RHO GTPase cycle	3.21E+00	5.88E-02	-1.961	ARHGAP12,ARHGAP15,ARHGAP20,ARHGAP21,ARHGAP39,ARHGDIG,ARHGEF28,CDC42EP4,DIAPH2,DOCK9,DSP,EFHD2,	
16					ELMO2,FILIP1,FMNL2,GFOD1,IQGAP2,KALRN,NET1,RASAL2,RHOB,SPATA13,SRC,STMN2,WDR6,WIPF3	
17	UVC-Induced MAPK Signaling	3.18E+00	1.37E-01	0	MAPK8,PRKCA,PRKCB,PRKD1,RAP2B,RASD2,SRC	
18	Serotonin Receptor Signaling	3.04E+00	5.73E-02	-0.392	ADCY1,BDNF,CACNB2,CACNG3,CCNE1,COPS5,FOXO3,GNAO1,GPLD1,HTR4,KALRN,KCNJ3,NAPEPLD,ORAI2,PLCH2,	
19					PRKCA,PRKCB,PRKD1,RAB4A,RAP2B,RASD2,RASGRF1,RHOB,RYR1,SRC,TRPC4	
20	Endothelin-1 Signaling	3.00E+00	7.65E-02	1.387	ADCY1,GNAO1,GNAZ,GPLD1,MAPK8,NAPEPLD,NOS1,PLCH2,PRKCA,PRKCB,PRKD1,RAP2B,RASD2,SRC	
21	CXCR4 Signaling	2.99E+00	7.98E-02	-0.333	ADCY1,CXCL12,ELMO2,GNAO1,GNAZ,MAPK8,PRKCA,PRKCB,PRKD1,RAP2B,RASD2,RHOB,SRC	
22	14-3-3-mediated Signaling	2.96E+00	8.80E-02	0.816	GFAP,MAPK8,PLCH2,PRKCA,PRKCB,PRKD1,RAP2B,RASD2,SRC,Tubb2b,TUBB4A	
23	Chemokine Signaling	2.82E+00	1.07E-01	-0.378	CAMK1D,CXCL12,MAPK8,PRKCA,PRKCB,RAP2B,RASD2,SRC	
24	Synaptic Long Term Depression	2.80E+00	7.29E-02	1.155	CACNB2,CACNG3,GAD1,GNAO1,GNAZ,GRM2,NOS1,PLCH2,PRKCA,PRKCB,PRKD1,RAP2B,RASD2,RYR1	
25	GADD45 Signaling	2.80E+00	1.19E-01	-0.378	ACVR1,ACVR1C,CCNE1,FOXO3,MAP3K4,MAPK8,TGFBR1	
26	Amyotrophic Lateral Sclerosis Signaling	2.79E+00	8.93E-02	1.633	CACNB2,CACNG3,CAPN3,CAPN5,GRIN3A,HECW1,NEFL,NEFM,NOS1,RNF19A	
27	Cachexia Signaling Pathway	2.76E+00	5.95E-02	-0.218	ACVR1,ACVR1C,ADCY1,CAPN3,CAPN5,CEBPB,CEBPD,DDIT4,FOXO3,FOXO6,IL1R1,LPL,MAPK8,NPY,PRKCA,PRKCB,PRKD1,	
28					PSMD1,PSME1,RYR1,TGFBR1	
29	L1CAM interactions	2.76E+00	8.85E-02	-0.632	ALCAM,CSNK2A2,DCX,FGFR1,SCN2B,SCN3A,SCN3B,SH3GL2,SRC,TUBB4A	
30	Human Embryonic Stem Cell Pluripotency	2.68E+00	7.07E-02	0	ACVR1,APC,BDNF,BMP1,FGFR1,ID2,NTF3,NTRK3,PRKCA,PRKCB,PRKD1,RAP2B,RASD2,TGFBR1	
31	G alpha (i) signalling events	2.68E+00	6.82E-02	-0.258	ADCY1,CXCL12,GNAZ,GRM2,NPY,NPY1R,NPY5R,OPRL1,PDYN,PENK,RGS14,RGS17,RGS4,SRC,SST	
32	Synaptogenesis Signaling Pathway	2.67E+00	6.09E-02	0.243	ADCY1,ARPC4,BDNF,CACNB2,CADM1,CDH11,CDH13,CDH9,EFNA3,EPHA10,EPHA5,GRIN3A,GRM2,KALRN,RAP2B,RASD2,	
33					RASGRF1,SRC,SYT16	
34	Circadian Rhythm Signaling	2.64E+00	6.37E-02	NaN	ADCY1,BDNF,BHLHE40,CACNB2,CACNG3,CSNK2A2,GAD1,GRIN3A,NOS1,PLCH2,PRKCA,PRKCB,PRKD1,RAP2B,RASD2,RYR1,SRC	
35	Remodeling of Epithelial Adherens Junctions	2.63E+00	1.11E-01	NaN	ACTN4,APC,ARPC4,MAPRE1,SRC,Tubb2b,TUBB4A	
36	Inositol phosphate metabolism	2.63E+00	1.28E-01	-0.816	INPP1,INPP4A,INPP5J,IP6K2,IPPK,PLCH2	
37	Neuropathic Pain Signaling in Dorsal Horn Neurons	2.58E+00	9.00E-02	-1	BDNF,CAMK1D,GRIN3A,GRM2,PLCH2,PRKCA,PRKCB,PRKD1,SRC	
38	G alpha (z) signalling events	2.58E+00	1.25E-01	0	ADCY1,GNAZ,PRKCA,PRKCB,RGS17,RGS4	
39	nNOS Signaling in Skeletal Muscle Cells	2.54E+00	1.22E-01	0.816	CACNB2,CACNG3,CAPN3,NOS1,RYR1,SNTA1	
40	GŒ±i Signaling	2.52E+00	7.75E-02	0	ADCY1,GNAO1,GNAZ,GRM2,NPY1R,OPRL1,RAP2B,RASD2,RGS14,RGS4,SRC	
41	Signaling by NOTCH3	2.49E+00	1.20E-01	0.447	FABP7,MAML2,MAMLD1,NEURL1,UBA52,WWC1	
42	PDGF Signaling	2.44E+00	9.30E-02	-0.378	CSNK2A2,INPP5J,MAPK8,PRKCA,PRKCB,RAP2B,RASD2,SRC	
43	PPARŒ±/RXRŒ± Activation	2.42E+00	6.88E-02	-0.632	ACVR1,ACVR1C,ADCY1,CHD5,IL1R1,LPL,MAPK8,PLCH2,PRKCA,PRKCB,RAP2B,RASD2,TGFBR1	
44	Regulation of Cellular Mechanics by Calpain Protease	2.38E+00	9.09E-02	1	ACTN4,CAPN3,CAPN5,CCNE1,ITGA7,RAP2B,RASD2,SRC	
45	Œ±-Adrenergic Signaling	2.36E+00	8.33E-02	-0.447	ADCY1,GNAO1,GNAZ,PRKCA,PRKCB,PRKD1,PYGB,RAP2B,RASD2	
46	PTEN Signaling	2.34E+00	7.33E-02	0.333	CSNK2A2,FGFR1,FOXO3,FOXO6,INPP5J,ITGA7,MAGI1,NTRK3,RAP2B,RASD2,TGFBR1	
47	Corticotropin Releasing Hormone Signaling	2.32E+00	7.28E-02	-1.265	ADCY1,BDNF,CACNB2,CACNG3,GAD1,GNAO1,JUND,NOS1,PRKCA,PRKCB,PRKD1	
48	Galactose catabolism	2.31E+00	5.00E-01	NaN	GALT,PGM1	
49	FcŒ≥ Receptor-mediated Phagocytosis in Macrophages and	2.29E+00	8.79E-02	-0.707	ARPC4,DGKB,GPLD1,NAPEPLD,PRKCA,PRKCB,PRKD1,SRC	
50	Cholecystokinin/Gastrin-mediated Signaling	2.23E+00	7.96E-02	0	MAPK8,PRKCA,PRKCB,PRKD1,RAP2B,RASD2,RHOB,SRC,SST	
51	Mechanisms of Viral Exit from Host Cells	2.23E+00	1.25E-01	NaN	CHMP4B,PRKCA,PRKCB,PRKD1,SH3GL2	
52	Signaling by NOTCH1	2.21E+00	9.33E-02	-0.378	DNER,HDAC11,ITCH,MAML2,MAMLD1,NEURL1,UBA52	
53	Role of NFAT in Cardiac Hypertrophy	2.20E+00	6.25E-02	0.577	ADCY1,CACNB2,CACNG3,CAMK1D,HDAC11,MAPK8,PLCH2,PRKCA,PRKCB,PRKD1,RAP2B,RASD2,SRC,TGFBR1	
54	IL-8 Signaling	2.18E+00	6.44E-02	0	GNAO1,GNAZ,GPLD1,LASP1,MAPK8,NAPEPLD,PRKCA,PRKCB,PRKD1,RAP2B,RASD2,RHOB,SRC	
55	Ephrin Receptor Signaling	2.18E+00	6.44E-02	0	ARPC4,CXCL12,EFNA3,EPHA10,EPHA5,GNAO1,GNAZ,GRIN3A,ITGA7,KALRN,RAP2B,RASD2,SRC	
56	Macropinocytosis Signaling	2.17E+00	9.21E-02	-0.447	ACTN4,PRKCA,PRKCB,PRKD1,RAP2B,RASD2,SRC	
57	Signaling by ROBO receptors	2.17E+00	6.19E-02	-1.155	ARHGAP39,CXCL12,DCC,LHX2,MAGOH,NELL2,PRKCA,PSMD1,PSME1,ROBO3,SLIT1,SLIT2,SRC,UBA52	
58	STAT3 Pathway	2.16E+00	7.30E-02	-0.378	FGFR1,IL11RA,IL1R1,MAPK8,NTRK3,RAP2B,RASD2,SRC,TGFA,TGFBR1	
59	Assembly of RNA Polymerase III Complex	2.15E+00	2.14E-01	NaN	BRF1,GTF3A,GTF3C1	
60	Signaling by ERBB4	2.14E+00	1.02E-01	-1.633	CXCL12,GFAP,ITCH,SRC,STMN1,UBA52	
61	Neuregulin Signaling	2.13E+00	7.69E-02	-0.378	ERRFI1,ITGA7,PRKCA,PRKCB,PRKD1,RAP2B,RASD2,SRC,TGFA	
62	Interconversion of nucleotide di- and triphosphates	2.13E+00	1.48E-01	1	AK1,AK4,GUK1,NUDT13	
63	Dopamine-DARPP32 Feedback in cAMP Signaling	2.10E+00	6.52E-02	0.333	ADCY1,CACNB2,CACNG3,CAMKK2,DRD5,GRIN3A,KCNJ3,NOS1,PLCH2,PRKCA,PRKCB,PRKD1	
64	Netrin-1 signaling	2.05E+00	1.14E-01	-0.447	DCC,SLIT1,SLIT2,SRC,TRPC4	
65	cAMP-mediated signaling	2.04E+00	5.98E-02	-1.387	ADCY1,AKAP9,CAMK1D,DRD5,GNAO1,GRM2,HTR4,NAPEPLD,NPY1R,OPRL1,PDE2A,RGS14,RGS4,SRC	
66	Thrombopoietin Signaling	2.00E+00	9.52E-02	-0.447	IRS2,PRKCA,PRKCB,PRKD1,RAP2B,RASD2	
67	Neuroinflammation Signaling Pathway	1.98E+00	5.57E-02	0.277	ACVR1,ACVR1C,B2M,BDNF,CALB1,CXCL12,GABRA2,GABRA5,GAD1,GRIN3A,HLA-DMA,IL1R1,KCNJ3,MAPK8,NTF3,TGFBR1	
68	PIP3 activates AKT signaling	1.97E+00	6.85E-02	-1.265	BDNF,FGFR1,FOXO3,FOXO6,IRS2,NTF3,NTRK3,PHLPP2,SRC,TGFA	
69	Thrombin Signaling	1.92E+00	5.96E-02	-0.333	ADCY1,ARHGEF28,CAMK1D,GNAO1,GNAZ,PLCH2,PRKCA,PRKCB,PRKD1,RAP2B,RASD2,RHOB,SRC	
70	Factors Promoting Cardiogenesis in Vertebrates	1.89E+00	6.67E-02	0	ACVR1,ACVR1C,APC,BMP1,MAPK8,PLCH2,PRKCA,PRKCB,PRKD1,TGFBR1	
71	GABA Receptor Signaling	1.83E+00	6.87E-02	NaN	ADCY1,CACNB2,CACNG3,GABRA2,GABRA5,GAD1,GNAO1,GNAZ,UBA52	
72	Synaptic Long Term Potentiation	1.81E+00	6.82E-02	0	ADCY1,GRIN3A,GRM2,PLCH2,PRKCA,PRKCB,PRKD1,RAP2B,RASD2	
73	Epithelial Adherens Junction Signaling	1.80E+00	6.45E-02	-1.265	ACVR1,ACVR1C,ARPC4,FGFR1,MAGI1,RAP2B,RASD2,SRC,TGFBR1,WWC1	
74	Inositol Pyrophosphates Biosynthesis	1.79E+00	2.86E-01	NaN	IP6K2,IPPK	
75	Signaling by NOTCH2	1.78E+00	1.18E-01	-1	MAML2,MAMLD1,NEURL1,UBA52	
76	GABAergic Receptor Signaling Pathway (Enhanced)	1.77E+00	6.72E-02	-1	ADCY1,CACNB2,CACNG3,GABRA2,GABRA5,GAD1,GNAO1,GRIN3A,KCNJ3	
77	EPH-Ephrin signaling	1.77E+00	7.69E-02	-1.134	ARHGEF28,ARPC4,EFNA3,EPHA10,EPHA5,KALRN,SRC	
78	Sleep NREM Signaling Pathway	1.75E+00	7.08E-02	-1.414	ADCY1,GABRA2,GABRA5,PARP1,PRKCA,RAP2B,RASD2,SIK3	
79	Virus Entry via Endocytic Pathways	1.73E+00	7.02E-02	0.378	B2M,FLNB,PRKCA,PRKCB,PRKD1,RAP2B,RASD2,SRC	
80	Signaling by EGFR	1.72E+00	9.43E-02	-1.342	PTPN3,SH3GL2,SRC,TGFA,UBA52	
81	ERBB Signaling	1.72E+00	7.53E-02	0	MAPK8,PRKCA,PRKCB,PRKD1,RAP2B,RASD2,TGFA	
82	Receptor-type tyrosine-protein phosphatases	1.71E+00	1.50E-01	NaN	NTRK3,PPFIA2,PTPRD	
83	IL-17A Signaling in Fibroblasts	1.71E+00	8.22E-02	-0.816	CEBPB,CEBPD,CXCL12,MAPK8,PRKCA,PRKCB	
84	Assembly and cell surface presentation of NMDA receptors	1.69E+00	1.11E-01	1	CASK,GRIN3A,NEFL,TUBB4A	
85	Signaling by TGF-beta Receptor Complex	1.69E+00	9.26E-02	-1.342	BAMBI,NEDD4L,STUB1,TGFBR1,UBA52	
86	Germ Cell-Sertoli Cell Junction Signaling	1.68E+00	6.17E-02	NaN	ACTN4,MAP3K4,MAPK8,RAP2B,RASD2,RHOB,SRC,TGFBR1,Tubb2b,TUBB4A	
87	RAF/MAP kinase cascade	1.68E+00	5.38E-02	-1.897	FGFR1,IRS2,KSR1,NEFL,PSMD1,PSME1,PTPN3,RASAL1,RASAL2,RASGRF1,SPRED1,SRC,TGFA,UBA52	
88	Transcriptional regulation by RUNX3	1.67E+00	7.37E-02	-2	FOXO3,MAML2,MAMLD1,PSMD1,PSME1,SRC,UBA52	
89	Pancreatic Secretion Signaling Pathway	1.66E+00	5.51E-02	-0.277	ADCY1,ARHGDIG,ATP2B4,CA10,CA12,LPL,PLCH2,PRKCA,PRKCB,PRKD1,RYR1,SLC4A4,SRC	
90	Leukocyte Extravasation Signaling	1.66E+00	5.88E-02	-0.905	ACTN4,ARHGAP12,CXCL12,EDIL3,MAP3K4,MAPK8,MMP17,PRKCA,PRKCB,PRKD1,SRC	
91	VEGF Signaling	1.65E+00	7.29E-02	0.447	ACTN4,FOXO3,PRKCA,PRKCB,RAP2B,RASD2,SRC	
92	Sertoli Cell-Sertoli Cell Junction Signaling	1.65E+00	5.49E-02	1.732	ACTN4,CDH11,CDH13,CDH9,IL1R1,MAP3K4,MAPK8,RAP2B,RASD2,SRC,TGFBR1,Tubb2b,TUBB4A	
93	GNRH Signaling	1.63E+00	5.82E-02	-0.707	ADCY1,CACNB2,CACNG3,MAP3K4,MAPK8,PRKCA,PRKCB,PRKD1,RAP2B,RASD2,SRC	
94	Activation of NMDA receptors and postsynaptic events	1.63E+00	7.89E-02	-0.447	ADCY1,CAMKK2,NEFL,RASGRF1,SRC,TUBB4A	
95	Apelin Endothelial Signaling Pathway	1.63E+00	6.34E-02	1.134	ADCY1,GNAO1,GNAZ,MAPK8,PRKCA,PRKCB,PRKD1,RAP2B,RASD2	
96	RHOGDI Signaling	1.61E+00	5.58E-02	1	ARHGAP12,ARHGDIG,ARPC4,CDH11,CDH13,CDH9,GNAO1,GNAZ,ITGA7,PRKCA,RHOB,SRC	
97	Antiproliferative Role of Somatostatin Receptor 2	1.60E+00	7.79E-02	1	GAD1,NOS1,RAP2B,RASD2,SRC,SST	
98	Thyroid Cancer Signaling	1.60E+00	7.79E-02	0	BDNF,IRS2,NTF3,NTRK3,RAP2B,RASD2	
99	Formation of paraxial mesoderm	1.60E+00	1.36E-01	NaN	FGFR1,MAML2,MAMLD1	
100	Neurotrophin/TRK Signaling	1.58E+00	7.69E-02	1	BDNF,MAPK8,NTF3,NTRK3,RAP2B,RASD2	
101	Caspase activation via Dependence Receptors in the absence	1.57E+00	2.22E-01	NaN	DAPK1,DCC	
102	Nephrin family interactions	1.55E+00	1.30E-01	NaN	ACTN4,CASK,KIRREL3	

	A	B	C	D	E	F
103	Apoptosis Signaling	1.55E+00	6.93E-02	1.633	CAPN3,CAPN5,MAPK8,PARP1,PRKCA,RAP2B,RASD2	
104	Hepatic Fibrosis Signaling Pathway	1.54E+00	4.70E-02	-0.258	ACVR1,ACVR1C,APC,BAMBI,CACNB2,CACNG3,CEBPB,FGFR1,IL1R1,IRS2,ITGA7,MAPK8,PRKCA,PRKCB,PRKD1,RAP2B,	
105					RASD2,RHOB,TGFBR1	
106	Endocannabinoid Neuronal Synapse Pathway	1.53E+00	6.08E-02	1.667	ADCY1,CACNB2,CACNG3,GNAO1,GRIN3A,KCNJ3,MAPK8,NAPEPLD,PLCH2	
107	Glioma Signaling	1.52E+00	6.40E-02	-0.816	CAMK1D,HDAC11,PRKCA,PRKCB,PRKD1,RAP2B,RASD2,TGFA	
108	RNA Polymerase III Transcription	1.51E+00	9.76E-02	2	BRF1,GTF3A,GTF3C1,POLR2L	
109	Glycogen metabolism	1.50E+00	1.25E-01	NaN	PGM1,PYGB,UBA52	
110	Glycogen Degradation II	1.48E+00	2.00E-01	NaN	PGM1,PYGB	
111	Transcriptional Regulation by MECP2	1.46E+00	8.06E-02	-0.447	BDNF,FKBP5,GAD1,PVALB,SST	
112	Signaling by NTRK2 (TRKB)	1.45E+00	1.20E-01	NaN	BDNF,NTF3,SRC	
113	Paxillin Signaling	1.45E+00	6.60E-02	0.447	ACTN4,ITGA7,MAPK8,PARVA,RAP2B,RASD2,SRC	
114	Integrin Signaling	1.44E+00	5.42E-02	0	ACTN4,ARPC4,CAPN3,CAPN5,ITGA7,MAPK8,PARVA,RAP2B,RASD2,RHOB,SRC	
115	Cardiac Hypertrophy Signaling (Enhanced)	1.43E+00	4.36E-02	-0.943	ACVR1,ACVR1C,ADCY1,CACNB2,CACNG3,DIAPH2,FGFR1,HDAC11,IL11RA,IL1R1,ITGA7,MAP3K4,MAPK8,NAPEPLD,	
116					PDE2A,PLCH2,PRKCA,PRKCB,PRKD1,RAP2B,RASD2,RYR1,TGFBR1	
117	HGF Signaling	1.43E+00	6.15E-02	0	ITGA7,MAP3K4,MAPK8,PRKCA,PRKCB,PRKD1,RAP2B,RASD2	
118	Xenobiotic Metabolism General Signaling Pathway	1.43E+00	6.15E-02	0.378	Gstm7,MAP3K4,MAPK8,PRKCA,PRKCB,PRKD1,RAP2B,RASD2	
119	CREB Signaling in Neurons	1.43E+00	4.28E-02	-0.853	ADCY1,CACNB2,CACNG3,DRD5,FGFR1,GNAO1,GNAZ,GPR22,GPRC5B,GRM2,HTR4,MAS1,NPY1R,NPY5R,NTRK3,	
120					OPRL1,PLCH2,POLR2L,PRKCA,PRKCB,PRKD1,RAP2B,RASD2,TGFA,TGFBR1	
121	LPS-stimulated MAPK Signaling	1.42E+00	7.06E-02	0.447	MAPK8,PRKCA,PRKCB,PRKD1,RAP2B,RASD2	
122	fMLP Signaling in Neutrophils	1.42E+00	6.11E-02	-0.447	ARPC4,GNAO1,GNAZ,PRKCA,PRKCB,PRKD1,RAP2B,RASD2	
123	Signaling by NTRK3 (TRKC)	1.41E+00	1.15E-01	NaN	NTF3,NTRK3,SRC	
124	Effects of PIP2 hydrolysis	1.37E+00	1.11E-01	NaN	DGKB,DGKD,DGKH	
125	Syndecan interactions	1.37E+00	1.11E-01	NaN	CASK,PRKCA,SDC3	
126	Protein Kinase A Signaling	1.36E+00	4.55E-02	1	ADCY1,AKAP9,DCC,DUSP11,FLNB,NAPEPLD,PDE2A,PLCH2,PRKCA,PRKCB,PRKD1,PTPN3,PTPRD,PTPRJ,PYGB,RYR1,	
127					TGFBR1,UBASH3B	
128	Gustation Pathway	1.36E+00	5.43E-02	-0.632	ADCY1,CACNB2,CACNG3,GABRA2,GABRA5,LPL,ORAI2,SCN2B,SCN3A,SCN3B	
129	Senescence Pathway	1.36E+00	4.86E-02	1.265	ACVR1,ACVR1C,BHLHE40,CACNB2,CACNG3,CAPN3,CAPN5,CCNE1,CEBPB,FOXO3,PARP1,RAP2B,RASD2,TGFBR1	
130	ERBB4 Signaling	1.34E+00	7.46E-02	0	PRKCA,PRKCB,PRKD1,RAP2B,RASD2	
131	Superpathway of Inositol Phosphate Compounds	1.34E+00	5.06E-02	-2.333	DUSP11,DUSP14,INPP5J,IP6K2,IPPK,PLCH2,PPFIA2,PPP1R16B,PPTC7,PTPN3,PTPRD,PTPRJ	
132	Glycogen Degradation III	1.33E+00	1.67E-01	NaN	PGM1,PYGB	
133	White Adipose Tissue Browning Pathway	1.32E+00	5.84E-02	-0.707	ADCY1,BDNF,CACNB2,CACNG3,CAMKK2,CEBPB,FGFR1,FNDC5	
134	Cell junction organization	1.32E+00	6.67E-02	0.816	CADM1,CADM3,CDH11,CDH13,CDH9,PARVA	
135	Pulmonary Fibrosis Idiopathic Signaling Pathway	1.30E+00	4.67E-02	0	ACVR1,ACVR1C,COL22A1,CXCL12,EZH1,FGFR1,FOXO3,FOXO6,IL11RA,MAPK8,MMP17,RAP2B,RASD2,TGFA,TGFBR1	
136	FOXO-mediated transcription of oxidative stress, metabolic a	1.29E+00	1.03E-01	NaN	FOXO3,FOXO6,NPY	
137	Fc Epsilon RI Signaling	1.29E+00	6.09E-02	0.816	INPP5J,MAPK8,PRKCA,PRKCB,PRKD1,RAP2B,RASD2	
138	GPCR-Mediated Nutrient Sensing in Enteroendocrine Cells	1.25E+00	5.98E-02	0.378	ADCY1,CACNB2,CACNG3,PLCH2,PRKCA,PRKCB,PRKD1	
139	Signaling by ALK	1.25E+00	1.00E-01	NaN	MYCN,PTN,SRC	
140	RIPK1-mediated regulated necrosis	1.25E+00	1.00E-01	NaN	ITCH,STUB1,UBA52	
141	Endosomal Sorting Complex Required For Transport (ESCRT)	1.25E+00	1.00E-01	NaN	CHMP4B,UBA52,VPS37D	
142	Melatonin Signaling	1.25E+00	7.04E-02	0.447	GNAO1,PLCH2,PRKCA,PRKCB,PRKD1	
143	Androgen Signaling	1.25E+00	5.39E-02	0	CACNB2,CACNG3,GNAO1,GNAZ,POLR2L,PRKCA,PRKCB,PRKD1,SRC	
144	GŒ±q Signaling	1.25E+00	5.39E-02	-0.378	GNAO1,GNAZ,GPLD1,NAPEPLD,PRKCA,PRKCB,PRKD1,RGS4,RHOB	
145	Prolactin Signaling	1.24E+00	6.38E-02	0	CEBPB,PRKCA,PRKCB,PRKD1,RAP2B,RASD2	
146	Non-Small Cell Lung Cancer Signaling	1.24E+00	6.38E-02	0	FOXO3,HDAC11,PRKCA,RAP2B,RASD2,TGFA	
147	Glutamate Dependent Acid Resistance	1.24E+00	5.00E-01	NaN	GAD1	
148	Signaling by ERBB2	1.24E+00	8.00E-02	-2	PRKCA,SRC,STUB1,UBA52	
149	Amyloid Processing	1.24E+00	8.00E-02	NaN	CAPN3,CAPN5,CSNK2A2,MARK1	
150	Pulmonary Healing Signaling Pathway	1.23E+00	5.15E-02	-0.632	CXCL12,FGFR1,MAPK8,MMP17,PRKCA,PRKCB,PRKD1,RAP2B,RASD2,SRC	
151	Molecular Mechanisms of Cancer	1.22E+00	3.90E-02	-1.616	ACVR1,ADCY1,APC,BMP1,CCNE1,DRD5,GNAO1,GNAZ,GPR22,GPRC5B,GRM2,HDAC11,HTR4,IL11RA,IL1R1,ITGA7,	
152					MAPK8,MAS1,MMP17,NPY1R,NPY5R,OPRL1,PRKCA,PRKCB,PRKD1,RAP2B,RASD2,RASGRF1,RHOB,SRC,TGFA,TGFBR1	
153	3-phosphoinositide Degradation	1.22E+00	5.13E-02	-2.646	DUSP11,DUSP14,INPP4A,INPP5J,PPFIA2,PPP1R16B,PPTC7,PTPN3,PTPRD,PTPRJ	
154	Glutamate binding, activation of AMPA receptors and synap	1.22E+00	9.68E-02	NaN	CACNG3,PRKCA,PRKCB	
155	Huntington's Disease Signaling	1.22E+00	4.73E-02	-0.707	BDNF,CAPN3,CAPN5,HDAC11,MAPK8,PENK,POLR2L,PRKCA,PRKCB,PRKD1,PSMD1,PSME1,UBA52	
156	Transcriptional activity of SMAD2/SMAD3:SMAD4 heterotrir	1.21E+00	7.84E-02	-1	CCNT1,NEDD4L,PARP1,UBA52	
157	Signaling by FGFR1	1.21E+00	7.84E-02	-1	FGFR1,SPRED1,SRC,UBA52	
158	LGI-ADAM interactions	1.21E+00	1.43E-01	NaN	CACNG3,LGI3	
159	Choline Biosynthesis III	1.21E+00	1.43E-01	NaN	GPLD1,NAPEPLD	
160	WNT/Œ≤-catenin Signaling	1.21E+00	5.29E-02	-0.707	ACVR1,ACVR1C,APC,CSNK2A2,GNAO1,SOX11,SRC,TGFBR1,UBA52	
161	Renin-Angiotensin Signaling	1.21E+00	5.83E-02	0.447	ADCY1,MAPK8,PRKCA,PRKCB,PRKD1,RAP2B,RASD2	
162	Oxytocin Signaling Pathway	1.20E+00	4.69E-02	1.387	ATP2B4,CACNB2,CACNG3,CAMKK2,GAD1,GNAO1,LPL,MAPK8,PRKCA,PRKCB,PRKD1,RAP2B,RASD2	
163	UVA-Induced MAPK Signaling	1.19E+00	6.19E-02	NaN	MAPK8,PARP1,PLCH2,PRKCA,RAP2B,RASD2	
164	TGF-Œ≤ Signaling	1.19E+00	6.19E-02	1.342	ACVR1,ACVR1C,MAPK8,RAP2B,RASD2,TGFBR1	
165	UVB-Induced MAPK Signaling	1.19E+00	7.69E-02	0	MAPK8,PRKCA,PRKCB,PRKD1	
166	VDR/RXR Activation	1.19E+00	6.76E-02	0.447	CALB1,CEBPB,PRKCA,PRKCB,PRKD1	
167	Caveolar-mediated Endocytosis Signaling	1.19E+00	6.76E-02	NaN	B2M,FLNB,ITGA7,PRKCA,SRC	
168	Netrin Signaling	1.18E+00	5.23E-02	0.333	ARPC4,CACNB2,CACNG3,DCC,MAPK8,PRKCA,PRKCB,PRKD1,RYR1	
169	Cargo concentration in the ER	1.18E+00	9.38E-02	NaN	CNIH1,LMAN2,TGFA	
170	Late endosomal microautophagy	1.18E+00	9.38E-02	NaN	CHMP4B,UBA52,VPS37D	
171	Cardiac Hypertrophy Signaling	1.17E+00	4.76E-02	0.378	ADCY1,CACNB2,CACNG3,GNAO1,GNAZ,MAP3K4,MAPK8,PLCH2,RAP2B,RASD2,RHOB,TGFBR1	
172	ID1 Signaling Pathway	1.17E+00	5.03E-02	1.265	ACVR1,ACVR1C,BHLHE40,CCNE1,FGFR1,FOXO3,RAP2B,RASD2,SRC,TGFBR1	
173	Pancreatic Adenocarcinoma Signaling	1.16E+00	5.69E-02	0	CCNE1,GPLD1,HDAC11,MAPK8,NAPEPLD,TGFA,TGFBR1	
174	Ion channel transport	1.16E+00	5.17E-02	-0.333	ASIC4,ATP1B2,ATP2B4,FXYD2,FXYD6,NEDD4L,RYR1,TRPC4,UBA52	
175	Triglyceride metabolism	1.15E+00	9.09E-02	NaN	DGAT2,FABP5,FABP7	
176	Glycosaminoglycan metabolism	1.15E+00	5.65E-02	-0.378	CEMIP,CSPG5,GLCE,HS6ST1,NDST4,SDC3,ST3GAL1	
177	ROBO SLIT Signaling Pathway	1.15E+00	5.65E-02	0.378	ARPC4,DCC,NELL2,ROBO3,SLIT1,SLIT2,SRC	
178	GŒ±s Signaling	1.15E+00	5.65E-02	-2.236	ADCY1,DRD5,GNAO1,GNAZ,HTR4,RYR1,SRC	
179	Signaling by PTK6	1.14E+00	7.41E-02	0	CCNE1,ELMO2,KHDRBS2,UBA52	
180	HIF1Œ± Signaling	1.14E+00	4.95E-02	0	CAMK1D,COPS5,MMP17,PRKCA,PRKCB,PRKD1,RAP2B,RASD2,STUB1,TGFA	
181	NF-Œ∫B Activation by Viruses	1.13E+00	6.49E-02	0	PRKCA,PRKCB,PRKD1,RAP2B,RASD2	
182	Role of Macrophages, Fibroblasts and Endothelial Cells in Rh	1.12E+00	4.47E-02	-1.069	APC,CEBPB,CEBPD,CXCL12,GNAO1,IGHM,IL1R1,PLCH2,PRKCA,PRKCB,PRKD1,RAP2B,RASD2,SRC	
183	Interleukin-1 family signaling	1.12E+00	5.56E-02	0	IL1R1,MAPK8,PELI2,PSMD1,PSME1,TNIP2,UBA52	
184	Endocannabinoid Developing Neuron Pathway	1.12E+00	5.56E-02	0.816	ADCY1,GNAO1,MAPK8,RAP2B,RASD2,SRC,STMN2	
185	EGF Signaling	1.12E+00	7.27E-02	-1	CSNK2A2,MAPK8,PRKCA,SRC	
186	Type II Diabetes Mellitus Signaling	1.11E+00	5.26E-02	0.816	CACNB2,CACNG3,CEBPB,IRS2,MAPK8,PRKCA,PRKCB,PRKD1	
187	FOXO-mediated transcription of cell death genes	1.11E+00	1.25E-01	NaN	BCL6,FOXO3	
188	D-myo-inositol (1,4,5)-trisphosphate Degradation	1.11E+00	1.25E-01	NaN	INPP1,INPP5J	
189	Potassium Channels	1.09E+00	5.83E-02	-0.816	KCNAB2,KCNC4,KCNF1,KCNG2,KCNJ3,KCNK2	
190	ESR-mediated signaling	1.09E+00	5.83E-02	-0.816	CCNT1,CXCL12,FKBP5,JUND,POLR2L,TGFA	
191	Post-translational protein phosphorylation	1.09E+00	5.83E-02	-0.816	CHGB,GOLM1,PENK,SCG2,TMEM132A,WFS1	
192	IGF-1 Signaling	1.09E+00	5.83E-02	0	CSNK2A2,FOXO3,IRS2,MAPK8,RAP2B,RASD2	
193	Signaling by MET	1.09E+00	6.33E-02	-0.447	PTPRJ,RAB4A,SH3GL2,SRC,UBA52	
194	Oxytocin in Spinal Neurons Signaling Pathway	1.09E+00	8.57E-02	NaN	CAMKK2,GAD1,NOS1	
195	Post-translational modification: synthesis of GPI-anchored p	1.07E+00	6.25E-02	0.447	GPLD1,LSAMP,LYPD1,NTNG1,RTN4RL1	
196	IL-3 Signaling	1.07E+00	6.25E-02	0	PRKCA,PRKCB,PRKD1,RAP2B,RASD2	
197	Diphthamide Biosynthesis	1.07E+00	3.33E-01	NaN	DPH6	
198	Methylglyoxal Degradation I	1.07E+00	3.33E-01	NaN	GLO1	
199	Glutathione Biosynthesis	1.07E+00	3.33E-01	NaN	GSS	
200	1D-myo-inositol Hexakisphosphate Biosynthesis V (from Ins(	1.07E+00	3.33E-01	NaN	IPPK	
201	L-serine Degradation	1.07E+00	3.33E-01	NaN	SRR	
202	Signaling by Rho Family GTPases	1.07E+00	4.56E-02	1	ARPC4,CDC42EP4,CDH11,CDH13,CDH9,GFAP,GNAO1,GNAZ,ITGA7,MAPK8,RHOB,STMN1	
203	GŒ±12/13 Signaling	1.06E+00	5.38E-02	-0.816	CDH11,CDH13,CDH9,MAPK8,RAP2B,RASD2,SRC	
204	FOXO-mediated transcription of cell cycle genes	1.06E+00	1.18E-01	NaN	FOXO3,PCBP4	

	A	B	C	D	E	F
205	1D-myo-inositol Hexakisphosphate Biosynthesis II (Mammal	1.06E+00	1.18E-01	NaN	INPP5J,IPPK	
206	Colorectal Cancer Metastasis Signaling	1.06E+00	4.55E-02	-1.414	ADCY1,APC,DCC,GNAO1,GNAZ,MAPK8,MMP17,RAP2B,RASD2,RHOB,SRC,TGFBR1	
207	Class A/1 (Rhodopsin-like receptors)	1.06E+00	4.45E-02	0.277	CXCL12,DRD5,HTR4,NLN,NPY,NPY1R,NPY5R,OPRL1,PDYN,PENK,PLPPR1,PLPPR3,SST	
208	PI Metabolism	1.06E+00	6.17E-02	-1.342	INPP4A,INPP5J,PLEKHA2,PLEKHA5,RAB4A	
209	Calcium Signaling	1.05E+00	4.76E-02	1.414	ATP2B4,CACNB2,CACNG3,CAMK1D,CAMKK2,GRIN3A,HDAC11,RAP2B,RYR1,TRPC4	
210	Semaphorin Signaling in Neurons	1.05E+00	6.90E-02	NaN	PLXNA2,RHOB,SEMA4D,SEMA7A	
211	Cancer Drug Resistance by Drug Efflux	1.05E+00	6.90E-02	1	FOXO3,FOXO6,RAP2B,RASD2	
212	CCR3 Signaling in Eosinophils	1.05E+00	5.34E-02	0	GNAO1,GNAZ,PRKCA,PRKCB,PRKD1,RAP2B,RASD2	
213	P2Y Purinergic Receptor Signaling Pathway	1.05E+00	5.34E-02	0	ADCY1,PLCH2,PRKCA,PRKCB,PRKD1,RAP2B,RASD2	
214	Oxytocin in Brain Signaling Pathway	1.04E+00	4.89E-02	1.667	CACNB2,CACNG3,CAMKK2,GNAO1,PRKCA,PRKCB,PRKD1,RAP2B,RASD2	
215	Transcriptional regulation of white adipocyte differentiation	1.04E+00	6.10E-02	-0.447	CEBPB,CEBPD,LPL,SREBF2,ZNF467	
216	Myelination Signaling Pathway	1.03E+00	4.33E-02	-0.832	ACVR1,APC,ARHGAP12,BDNF,BMP1,FGFR1,HDAC11,ID2,NTF3,RAP2B,RASD2,SRC,SREBF2,ZNF24	
217	AMPK Signaling	1.02E+00	4.58E-02	NaN	AK1,AK4,CAMKK2,FOXO3,FOXO6,GNAO1,GNAZ,IRS2,ORAI2,PFKP,PHLPP2	
218	VEGF Family Ligand-Receptor Interactions	1.02E+00	6.02E-02	0	PRKCA,PRKCB,PRKD1,RAP2B,RASD2	
219	Formation of the nephric duct	1.02E+00	1.11E-01	NaN	NPNT,PCDH19	
220	GABA receptor activation	1.01E+00	6.67E-02	-1	ADCY1,GABRA2,GABRA5,KCNJ3	
221	FLT3 Signaling	1.01E+00	7.89E-02	NaN	FOXO3,PTPRJ,UBA52	
222	Hedgehog 'off' state	1.00E+00	5.50E-02	0	ADCY1,ITCH,PSMD1,PSME1,TUBB4A,UBA52	
223	IL-2 Signaling	9.90E-01	6.56E-02	NaN	CSNK2A2,MAPK8,RAP2B,RASD2	
224	Wound Healing Signaling Pathway	9.88E-01	4.51E-02	0.302	ACVR1,ACVR1C,CEBPB,COL22A1,IL1R1,MAPK8,PRKCA,RAP2B,RASD2,TGFA,TGFBR1	
225	Role of Chondrocytes in Rheumatoid Arthritis Signaling Path	9.84E-01	5.15E-02	-1.89	CAMKK2,CEBPB,CEBPD,CXCL12,FOXO3,IL1R1,MMP17	
226	Transcriptional regulation by RUNX1	9.84E-01	5.15E-02	0.378	CSNK2A2,ITCH,PRKCB,PSMD1,PSME1,UBA52,YAF2	
227	MAP kinase activation	9.81E-01	7.69E-02	NaN	MAPK8,TNIP2,UBA52	
228	Mitophagy	9.81E-01	7.69E-02	NaN	CSNK2A2,SRC,UBA52	
229	Regulation of TP53 Activity through Methylation	9.79E-01	1.05E-01	NaN	SMYD2,UBA52	
230	FOXO-mediated transcription	9.79E-01	1.05E-01	NaN	FOXO3,FOXO6	
231	Signaling by the B Cell Receptor (BCR)	9.73E-01	5.41E-02	0	IGHM,ORAI2,PRKCB,PSMD1,PSME1,UBA52	
232	Adipogenesis pathway	9.72E-01	5.11E-02	-1.134	ACVR1,CEBPB,CEBPD,DGKD,FGFR1,HDAC11,LPL	
233	Neurovascular Coupling Signaling Pathway	9.62E-01	4.57E-02	-1.265	CACNB2,CACNG3,GABRA2,GABRA5,GAD1,GRIN3A,KCNJ3,NOS1,ORAI2,RYR1	
234	Adrenergic Receptor Signaling Pathway (Enhanced)	9.57E-01	4.69E-02	0.333	ADCY1,BDNF,CACNB2,CACNG3,GNAO1,PLCH2,PRKCA,PRKCB,PRKD1	
235	DAG and IP3 signaling	9.56E-01	7.50E-02	NaN	ADCY1,CAMKK2,PRKCA	
236	RET signaling	9.56E-01	7.50E-02	NaN	IRS2,PRKCA,SRC	
237	Retinoid metabolism and transport	9.56E-01	7.50E-02	NaN	LPL,LRP12,SDC3	
238	BMP signaling pathway	9.56E-01	5.75E-02	NaN	ACVR1,BMP1,MAPK8,RAP2B,RASD2	
239	Taurine Biosynthesis	9.52E-01	2.50E-01	NaN	CSAD	
240	Rapoport-Luebering Glycolytic Shunt	9.52E-01	2.50E-01	NaN	PGAM2	
241	N-acetylglucosamine Degradation II	9.52E-01	2.50E-01	NaN	NAGK	
242	Phospholipases	9.51E-01	6.35E-02	NaN	GPLD1,LPL,NAPEPLD,PLCH2	
243	CDK5 Signaling	9.46E-01	5.31E-02	-1.342	ADCY1,BDNF,DRD5,MAPK8,RAP2B,RASD2	
244	Sperm Motility	9.43E-01	4.42E-02	0.447	EPHA5,FGFR1,GNAO1,GNAZ,NTRK3,PDE2A,PLCH2,PRKCA,PRKCB,PRKD1,SRC	
245	Signaling by NODAL	9.41E-01	1.00E-01	NaN	ACVR1C,FOXO3	
246	Hepatitis B Chronic Liver Pathogenesis Signaling Pathway	9.33E-01	4.79E-02	1.414	CAMKK2,MAPK8,PRKCA,PRKCB,PRKD1,RAP2B,RASD2,SATB1	
247	Semaphorin interactions	9.33E-01	6.25E-02	-1	PLXNA2,RHOB,SEMA4D,SEMA7A	
248	Endocannabinoid Cancer Inhibition Pathway	9.24E-01	4.96E-02	1.134	ADCY1,CAMKK2,CCNE1,GNAO1,NOS1,SPTLC2,SRC	
249	Erythropoietin Signaling Pathway	9.23E-01	4.76E-02	0	FOXO3,IRS2,PRKCA,PRKCB,PRKD1,RAP2B,RASD2,SRC	
250	CLEAR Signaling Pathway	9.13E-01	4.27E-02	-1.155	DDIT4,FGFR1,NTRK3,PLBD2,PRKCA,PRKCB,PRKD1,RAP2B,RASD2,STUB1,TGFA,TGFBR1	
251	Actin Nucleation by ARP-WASP Complex	9.10E-01	5.56E-02	NaN	ARPC4,ITGA7,RAP2B,RASD2,RHOB	
252	Synaptic adhesion-like molecules	9.06E-01	9.52E-02	NaN	LRFN4,PTPRD	
253	Chaperone Mediated Autophagy	9.06E-01	9.52E-02	NaN	GFAP,UBA52	
254	Formation of the ureteric bud	9.06E-01	9.52E-02	NaN	NPNT,SLIT2	
255	MAPK6/MAPK4 signaling	8.95E-01	5.49E-02	NaN	FOXO3,KALRN,PSMD1,PSME1,UBA52	
256	Regulation of Insulin-like Growth Factor (IGF) transport and	8.94E-01	5.13E-02	-0.816	CHGB,GOLM1,PENK,SCG2,TMEM132A,WFS1	
257	Nitric Oxide Signaling in the Cardiovascular System	8.94E-01	5.13E-02	-1	CACNB2,CACNG3,PDE2A,PRKCA,PRKCB,PRKD1	
258	Salvage Pathways of Pyrimidine Ribonucleotides	8.81E-01	5.43E-02	1.342	AK1,AK4,CDK18,DAPK1,MAPK8	
259	Role of Tissue Factor in Cancer	8.79E-01	4.50E-02	0.333	ACVR1,ACVR1C,MAPK8,PRKCA,RAP2B,RASD2,SRC,TGFA,TGFBR1	
260	D-myo-inositol-5-phosphate Metabolism	8.79E-01	4.50E-02	-1.633	DUSP11,DUSP14,PLCH2,PPFIA2,PPP1R16B,PPTC7,PTPN3,PTPRD,PTPRJ	
261	Pyrimidine Deoxyribonucleotides De Novo Biosynthesis I	8.73E-01	9.09E-02	NaN	AK1,AK4	
262	Superpathway of D-myo-inositol (1,4,5)-trisphosphate Metal	8.73E-01	9.09E-02	NaN	INPP1,INPP5J	
263	G-Protein Coupled Receptor Signaling	8.70E-01	3.70E-02	-1.4	ADCY1,DRD5,FOXO3,FOXO6,GNAO1,GNAZ,GPR22,GPRC5B,GRM2,HTR4,MAP3K4,MAPK8,MAS1,	
264					NAPEPLD,NPY1R,NPY5R,OPRL1,PDE2A,PRKCA,PRKCB,RAP2B,RASD2,RGS14,RGS4,SRC	
265	Agrin Interactions at Neuromuscular Junction	8.63E-01	5.88E-02	NaN	MAPK8,RAP2B,RASD2,SRC	
266	Creatine-phosphate Biosynthesis	8.61E-01	2.00E-01	NaN	CKMT1B	
267	CMP-N-acetylneuraminate Biosynthesis I (Eukaryotes)	8.61E-01	2.00E-01	NaN	NAGK	
268	Citrulline-Nitric Oxide Cycle	8.61E-01	2.00E-01	NaN	NOS1	
269	Galactose Degradation I (Leloir Pathway)	8.61E-01	2.00E-01	NaN	GALT	
270	Glutamate Degradation III (via 4-aminobutyrate)	8.61E-01	2.00E-01	NaN	GAD1	
271	Gap Junction Signaling	8.59E-01	4.10E-02	0.577	ADCY1,GAD1,MAPK8,PLCH2,PRKCA,PRKCB,PRKD1,RAP2B,RASD2,SRC,TGFBR1,Tubb2b,TUBB4A	
272	Sertoli Cell-Germ Cell Junction Signaling Pathway (Enhanced	8.54E-01	4.33E-02	-0.632	ARPC4,FOSL2,IL1R1,MAP3K4,MAPK8,RAP2B,RASD2,SRC,TGFBR1,UBE2J1	
273	RHO GTPases Activate WASPs and WAVEs	8.43E-01	6.67E-02	NaN	ARPC4,SRC,WIPF3	
274	mTOR Signaling	8.33E-01	4.39E-02	-0.378	DDIT4,GPLD1,NAPEPLD,PRKCA,PRKCB,PRKD1,RAP2B,RASD2,RHOB	
275	Cardiac Œ≤-adrenergic Signaling	8.32E-01	4.52E-02	NaN	ADCY1,AKAP9,CACNB2,CACNG3,GNAO1,GNAZ,NAPEPLD,PDE2A	
276	Orexin Signaling Pathway	8.29E-01	4.27E-02	0	ADCY1,CACNB2,CACNG3,KCNJ3,PLCH2,PRKCA,PRKCB,PRKD1,SRC,TRPC4	
277	IL-1 Signaling	8.26E-01	5.21E-02	NaN	ADCY1,GNAO1,GNAZ,IL1R1,MAPK8	
278	Xenobiotic Metabolism Signaling	8.20E-01	4.17E-02	NaN	CAMK1D,Gstm7,HS6ST1,MAP3K4,MAPK8,NDST4,PRKCA,PRKCB,PRKD1,RAP2B,RASD2	
279	Apelin Cardiomyocyte Signaling Pathway	8.12E-01	5.15E-02	0.447	MAPK8,PLCH2,PRKCA,PRKCB,PRKD1	
280	MyD88 cascade initiated on plasma membrane	8.12E-01	8.33E-02	NaN	PELI2,UBA52	
281	RHO GTPases activate IQGAPs	8.12E-01	8.33E-02	NaN	IQGAP2,TUBB4A	
282	RHO GTPases Activate NADPH Oxidases	8.12E-01	8.33E-02	NaN	PRKCA,PRKCB	
283	Interleukin-6 family signaling	8.12E-01	8.33E-02	NaN	CRLF1,IL11RA	
284	IL-6 Signaling	8.09E-01	4.84E-02	0.447	CEBPB,CSNK2A2,IL1R1,MAPK8,RAP2B,RASD2	
285	TCF dependent signaling in response to WNT	8.03E-01	4.44E-02	0.707	APC,ASH2L,CSNK2A2,HECW1,PSMD1,PSME1,RBBP5,UBA52	
286	HSP90 chaperone cycle for steroid hormone receptors in the	8.03E-01	6.38E-02	NaN	FKBP5,NR3C2,TUBB4A	
287	XBP1(S) activates chaperone genes	8.03E-01	6.38E-02	NaN	ACADVL,CTDSP2,WFS1	
288	Sumoylation Pathway	8.00E-01	5.10E-02	0.447	ARHGDIG,DNMT3A,MAPK8,RHOB,STUB1	
289	Regulation of RUNX2 expression and activity	7.99E-01	5.56E-02	0	PSMD1,PSME1,STUB1,UBA52	
290	Nuclear Cytoskeleton Signaling Pathway	7.90E-01	4.29E-02	1	CDH11,CDH13,CDH9,CSNK2A2,ITGA7,KIF26B,KIF5A,SRC,TUBB4A	
291	Docosahexaenoic Acid (DHA) Signaling	7.88E-01	4.18E-02	0	ADCY1,BDNF,FABP5,FABP7,PLCH2,PRKCA,PRKCB,PRKD1,SYT16,TGFA	
292	Lipid particle organization	7.88E-01	1.67E-01	NaN	CIDEA	
293	DNA Double Strand Break Response	7.84E-01	6.25E-02	NaN	BAZ1B,MAPK8,UBA52	
294	Pyruvate metabolism	7.84E-01	6.25E-02	NaN	GLO1,PDP1,UBA52	
295	ERK5 Signaling	7.84E-01	5.48E-02	NaN	FOXO3,RAP2B,RASD2,SRC	
296	Ephrin B Signaling	7.84E-01	5.48E-02	0	CXCL12,GNAO1,GNAZ,KALRN	
297	D-myo-inositol (1,4,5,6)-Tetrakisphosphate Biosynthesis	7.67E-01	4.35E-02	-2.236	DUSP11,DUSP14,PPFIA2,PPP1R16B,PPTC7,PTPN3,PTPRD,PTPRJ	
298	D-myo-inositol (3,4,5,6)-tetrakisphosphate Biosynthesis	7.67E-01	4.35E-02	-2.236	DUSP11,DUSP14,PPFIA2,PPP1R16B,PPTC7,PTPN3,PTPRD,PTPRJ	
299	TP53 Regulates Transcription of Cell Cycle Genes	7.66E-01	6.12E-02	NaN	CCNE1,CNOT10,PCBP4	
300	Clathrin-mediated endocytosis	7.65E-01	4.69E-02	-0.816	ARPC4,FCHO1,PACSIN2,SH3GL2,TGFA,UBA52	
301	Glycolysis I	7.57E-01	7.69E-02	NaN	PFKP,PGAM2	
302	Antigen Presentation Pathway	7.57E-01	7.69E-02	NaN	B2M,HLA-DMA	
303	Apoptotic execution phase	7.48E-01	6.00E-02	NaN	APC,DSP,SATB1	
304	GDNF Family Ligand-Receptor Interactions	7.41E-01	5.26E-02	NaN	IRS2,MAPK8,RAP2B,RASD2	
305	RHO GTPases Activate Formins	7.33E-01	4.58E-02	0	DIAPH2,FMNL2,MAPRE1,RHOB,SRC,TUBB4A	
306	Kinesins	7.30E-01	5.88E-02	NaN	KIF26B,KIF5A,TUBB4A	

	A	B	C	D	E	F
307	FAT10 Cancer Signaling Pathway	7.30E-01	5.88E-02	NaN	ACVR1,ACVR1C,TGFBR1	
308	Epithelial-Mesenchymal Transition (EMT) during gastrulatior	7.27E-01	1.43E-01	NaN	FGFR1	
309	Ceramide Biosynthesis	7.27E-01	1.43E-01	NaN	SPTLC2	
310	IL-7 Signaling Pathway	7.27E-01	5.19E-02	NaN	BCL6,FOXO3,FOXO6,IGHM	
311	WNK Renal Signaling Pathway	7.26E-01	4.81E-02	-1.342	IRS2,NR3C2,PRKCA,PRKCB,PRKD1	
312	Cargo recognition for clathrin-mediated endocytosis	7.26E-01	4.81E-02	0.447	COPS5,FCHO1,SH3GL2,TGFA,UBA52	
313	Acetylcholine Receptor Signaling Pathway	7.24E-01	4.23E-02	0.707	ADCY1,CACNB2,CACNG3,GNAO1,PLCH2,PRKCA,PRKCB,PRKD1	
314	Transport of bile salts and organic acids, metal ions and ami	7.13E-01	5.13E-02	0	SLC2A13,SLC39A10,SLC39A6,SLC44A5	
315	Renal Cell Carcinoma Signaling	7.13E-01	5.13E-02	NaN	RAP2B,RASD2,TGFA,UBA52	
316	Role of MAPK Signaling in the Pathogenesis of Influenza	7.13E-01	5.13E-02	NaN	MAPK8,PRKCA,RAP2B,RASD2	
317	DHCR24 Signaling Pathway	7.13E-01	4.51E-02	0	PRKCA,PRKCB,PRKD1,RAP2B,RASD2,SREBF2	
318	Glycation Signaling Pathway	7.02E-01	4.07E-02	0.333	GLO1,MAPK8,PFKP,PRKCA,PRKCB,PRKD1,RAP2B,RASD2,SRC	
319	Estrogen Receptor Signaling	7.00E-01	3.73E-02	-0.277	ADCY1,CACNB2,CACNG3,FOXO3,FOXO6,GNAO1,GNAZ,MMP17,PLCH2,PRKCA,PRKCB,PRKD1,RAP2B,RASD2,SRC	
320	Regulation of Apoptosis	6.97E-01	5.66E-02	NaN	PSMD1,PSME1,UBA52	
321	Mitochondrial Division Signaling Pathway	6.90E-01	4.27E-02	1.134	CACNB2,CACNG3,INF2,NOS1,PRKD1,RAP2B,RASD2	
322	Transcriptional Regulatory Network in Embryonic Stem Cells	6.90E-01	4.27E-02	1.134	ACVR1,ACVR1C,CALB1,FGFR1,RAP2B,RASD2,TGFBR1	
323	MHC class II antigen presentation	6.81E-01	4.63E-02	2.236	HLA-DMA,KIF5A,OSBPL1A,SH3GL2,TUBB4A	
324	RAC Signaling	6.74E-01	4.38E-02	0	ARPC4,IQGAP2,ITGA7,MAPK8,RAP2B,RASD2	
325	NRF2-mediated Oxidative Stress Response	6.72E-01	4.00E-02	0	FKBP5,Gstm7,JUND,MAPK8,PRKCA,PRKCB,PRKD1,RAP2B,RASD2	
326	O-linked glycosylation	6.71E-01	4.59E-02	1.342	GALNT14,GALNT17,SEMA5B,SPON1,ST3GAL1	
327	Activin Inhibin Signaling Pathway	6.67E-01	4.08E-02	0	ACVR1,BAMBI,CEBPB,FOSL2,FOXO3,IGHM,IL1R1,MAPK8	
328	Neurexins and neuroligins	6.65E-01	5.45E-02	NaN	BEGAIN,CASK,HOMER3	
329	G alpha (s) signalling events	6.65E-01	4.35E-02	-0.816	ADCY1,DRD5,GNAZ,HTR4,PDE2A,SRC	
330	Insulin Receptor Signaling	6.65E-01	4.35E-02	0.447	FOXO3,INPP5J,IRS2,MAPK8,RAP2B,RASD2	
331	HER-2 Signaling in Breast Cancer	6.65E-01	3.98E-02	0.333	CCNE1,FOXO3,PRKCA,PRKCB,PRKD1,RAP2B,RASD2,SRC,TGFA	
332	mRNA 3 Prime End Processing Signaling Pathway	6.60E-01	4.55E-02	0.447	CNOT10,CPEB1,IGHM,PABPN1,PCBP4	
333	Xenobiotic Metabolism CAR Signaling Pathway	6.56E-01	4.17E-02	0	Gstm7,HS6ST1,NDST4,PRKCA,PRKCB,PRKD1,SRC	
334	TNF signaling	6.50E-01	5.36E-02	NaN	STUB1,TAX1BP1,UBA52	
335	Glioblastoma Multiforme Signaling	6.48E-01	4.14E-02	-0.447	APC,CCNE1,PLCH2,RAP2B,RASD2,RHOB,SRC	
336	Reelin Signaling in Neurons	6.46E-01	4.29E-02	0.447	ARHGEF28,ARPC4,DCX,GRIN3A,MAPK8,SRC	
337	Signaling by CSF1 (M-CSF) in myeloid cells	6.41E-01	6.45E-02	NaN	SRC,UBA52	
338	MyD88 dependent cascade initiated on endosome	6.41E-01	6.45E-02	NaN	PELI2,UBA52	
339	Transcriptional regulation of brown and beige adipocyte diff	6.41E-01	6.45E-02	NaN	CIDEA,USP46	
340	RHO GTPases Activate Rhotekin and Rhophilins	6.31E-01	1.11E-01	NaN	RHOB	
341	Serine biosynthesis	6.31E-01	1.11E-01	NaN	SRR	
342	GDP-glucose Biosynthesis	6.31E-01	1.11E-01	NaN	PGM1	
343	Bladder Cancer Signaling	6.29E-01	4.42E-02	NaN	DAPK1,HDAC11,MMP17,RAP2B,RASD2	
344	Platelet homeostasis	6.26E-01	4.71E-02	1	ATP2B4,NOS1,ORAI2,PDE2A	
345	Autism Signaling Pathway	6.22E-01	3.75E-02	0.905	APC,B2M,BDNF,CACNB2,CACNG3,CAMKK2,GRIN3A,GRM2,NOS1,RAP2B,RASD2	
346	Sensory perception of taste	6.21E-01	6.25E-02	NaN	SCN2B,SCN3A	
347	Glutathione-mediated Detoxification	6.21E-01	6.25E-02	NaN	Gsta4,Gstm7	
348	Cell surface interactions at the vascular wall	6.10E-01	4.35E-02	-0.447	ATP1B2,GRB14,IGHM,SDC3,SRC	
349	Inhibition of Angiogenesis by TSP1	6.01E-01	6.06E-02	NaN	MAPK8,TGFBR1	
350	IL-12 Signaling and Production in Macrophages	5.93E-01	3.88E-02	0	CAMKK2,CEBPB,IGHM,MAPK8,PRKCA,PRKCB,PRKD1,TNIP2	
351	NIK-->noncanonical NF-kB signaling	5.93E-01	5.00E-02	NaN	PSMD1,PSME1,UBA52	
352	Endometrial Cancer Signaling	5.93E-01	5.00E-02	NaN	FOXO3,RAP2B,RASD2	
353	PCP (Planar Cell Polarity) Pathway	5.93E-01	5.00E-02	NaN	JUND,MAPK8,PRICKLE1	
354	RHOBTB3 ATPase cycle	5.91E-01	1.00E-01	NaN	CCNE1	
355	Glucose and Glucose-1-phosphate Degradation	5.91E-01	1.00E-01	NaN	PGM1	
356	Calcium Transport I	5.91E-01	1.00E-01	NaN	ATP2B4	
357	Opioid Signalling	5.82E-01	4.49E-02	NaN	ADCY1,CAMKK2,PDYN,PRKCA	
358	Fcgamma receptor (FCGR) dependent phagocytosis	5.82E-01	4.49E-02	NaN	ARPC4,ELMO2,SRC,WIPF3	
359	Regulation of mRNA stability by proteins that bind AU-rich e	5.82E-01	4.49E-02	0	PRKCA,PSMD1,PSME1,UBA52	
360	Unfolded protein response	5.82E-01	4.49E-02	-1	CEBPB,CEBPD,MAPK8,SREBF2	
361	S100 Family Signaling Pathway	5.78E-01	3.37E-02	-1.633	BDNF,CACNB2,CACNG3,COPS5,DRD5,FGFR1,GPR22,GPRC5B,GRM2,HTR4,IGHM,MAPK8,MAS1,	
362					MMP17,NPY1R,NPY5R,NTF3,OPRL1,PLCH2,PRKCA,PRKCB,PRKD1,SRC,TGFA	
363	Degradation of beta-catenin by the destruction complex	5.71E-01	4.44E-02	1	APC,PSMD1,PSME1,UBA52	
364	Keratinization	5.71E-01	4.44E-02	0	DSP,KRT12,KRT2,PKP2	
365	Ceramide Signaling	5.71E-01	4.44E-02	NaN	KSR1,MAPK8,RAP2B,RASD2	
366	Hepatic Cholestasis	5.66E-01	3.81E-02	-0.707	ADCY1,CYP7B1,IL1R1,MAP3K4,MAPK8,PRKCA,PRKCB,PRKD1	
367	3-phosphoinositide Biosynthesis	5.66E-01	3.81E-02	-2.236	DUSP11,DUSP14,PPFIA2,PPP1R16B,PPTC7,PTPN3,PTPRD,PTPRJ	
368	WNT/SHH Axonal Guidance Signaling Pathway	5.62E-01	4.00E-02	-0.816	ADCY1,MAPK8,PLXNA2,PRKCA,SRC,TRPC4	
369	Regulation of TP53 Activity through Phosphorylation	5.61E-01	4.40E-02	0	CSNK2A2,DYRK2,NUAK1,UBA52	
370	Histone Modification Signaling Pathway	5.58E-01	3.66E-02	-0.632	BAZ1B,CSNK2A2,DNMT3A,EZH1,HDAC11,KDM7A,MAPK8,PKD1,PRKCB,SMYD2	
371	Signaling by Leptin	5.56E-01	9.09E-02	NaN	IRS2	
372	DNA methylation	5.56E-01	9.09E-02	NaN	DNMT3A	
373	RHO GTPases activate KTN1	5.56E-01	9.09E-02	NaN	KIF5A	
374	Alpha-protein kinase 1 signaling pathway	5.56E-01	9.09E-02	NaN	UBA52	
375	Pexophagy	5.56E-01	9.09E-02	NaN	UBA52	
376	Glycine Betaine Degradation	5.56E-01	9.09E-02	NaN	SRR	
377	NCAM signaling for neurite out-growth	5.54E-01	4.76E-02	NaN	CACNB2,FGFR1,SRC	
378	RAB geranylgeranylation	5.54E-01	4.76E-02	NaN	RAB33A,RAB3C,RAB4A	
379	Insulin Secretion Signaling Pathway	5.52E-01	3.65E-02	-1.414	ADCY1,Arxes1/Arxes2,CACNB2,CACNG3,PLCH2,PRKCA,PRKCB,PRKD1,RYR1,SRC	
380	Aggrephagy	5.48E-01	5.56E-02	NaN	TUBB4A,UBA52	
381	ERK/MAPK Signaling	5.47E-01	3.76E-02	0	ITGA7,KSR1,MYCN,PRKCA,PRKCB,RAP2B,RASD2,SRC	
382	Glycerophospholipid biosynthesis	5.46E-01	4.10E-02	-0.447	CSNK2A2,DGAT2,PITPNM1,PITPNM2,SLC44A5	
383	Role of NANOG in Mammalian Embryonic Stem Cell Pluripot	5.46E-01	4.10E-02	NaN	ACVR1,APC,BMP1,RAP2B,RASD2	
384	Amyloid fiber formation	5.42E-01	4.69E-02	NaN	B2M,CALB1,UBA52	
385	Relaxin Signaling	5.38E-01	3.92E-02	NaN	ADCY1,GAD1,GNAO1,GNAZ,NAPEPLD,PDE2A	
386	Hepatic Fibrosis / Hepatic Stellate Cell Activation	5.35E-01	3.80E-02	NaN	BAMBI,COL22A1,FGFR1,IL1R1,LHX2,TGFA,TGFBR1	
387	Chromatin organization	5.34E-01	3.72E-02	0	ASH2L,ATF7IP,ATXN7L3,DNMT3A,KDM7A,RBBP5,SMYD2,WDR77	
388	B-WICH complex positively regulates rRNA expression	5.32E-01	5.41E-02	NaN	BAZ1B,POLR2L	
389	Hedgehog ligand biogenesis	5.30E-01	4.62E-02	NaN	PSMD1,PSME1,UBA52	
390	Pyridoxal 5'-phosphate Salvage Pathway	5.30E-01	4.62E-02	NaN	CDK18,DAPK1,MAPK8	
391	Presynaptic depolarization and calcium channel opening	5.24E-01	8.33E-02	NaN	CACNB2	
392	Reversible hydration of carbon dioxide	5.24E-01	8.33E-02	NaN	CA12	
393	Trafficking and processing of endosomal TLR	5.24E-01	8.33E-02	NaN	CNPY3	
394	Signal regulatory protein family interactions	5.24E-01	8.33E-02	NaN	SRC	
395	GP1b-IX-V activation signalling	5.24E-01	8.33E-02	NaN	SRC	
396	HDR through MMEJ (alt-NHEJ)	5.24E-01	8.33E-02	NaN	PARP1	
397	Lysine catabolism	5.24E-01	8.33E-02	NaN	CRYM	
398	Z-decay: degradation of maternal mRNAs by zygotically expr	5.24E-01	8.33E-02	NaN	PABPN1	
399	Œ≥-glutamyl Cycle	5.24E-01	8.33E-02	NaN	GSS	
400	DNA Methylation and Transcriptional Repression Signaling	5.21E-01	4.21E-02	NaN	CDK18,CEBPB,CHD5,DNMT3A	
401	DDX58/IFIH1-mediated induction of interferon-alpha/beta	5.18E-01	4.55E-02	NaN	ITCH,TAX1BP1,UBA52	
402	Carboxyterminal post-translational modifications of tubulin	5.16E-01	5.26E-02	NaN	TTLL7,TUBB4A	
403	Breast Cancer Regulation by Stathmin1	5.14E-01	3.34E-02	-1.414	CAMK1D,DRD5,GPR22,GPRC5B,GRM2,HTR4,MAS1,NPY1R,NPY5R,OPRL1,PRKCA,PRKCB,PRKD1,RAP2B,	
404					RASD2,STMN1,TGFA,Tubb2b,TUBB4A	
405	ABC-family proteins mediated transport	5.12E-01	4.17E-02	1	PSMD1,PSME1,RNF5,UBA52	
406	HEY1 Signaling Pathway	5.09E-01	3.82E-02	-0.816	ACVR1,ACVR1C,BMP1,MMP17,NTRK3,TGFBR1	
407	TNFR2 non-canonical NF-kB pathway	5.07E-01	4.48E-02	NaN	PSMD1,PSME1,UBA52	
408	Glutamate Receptor Signaling	5.07E-01	4.48E-02	NaN	GRIN3A,GRM2,HOMER3	

	A	B	C	D	E	F
409	Melanocyte Development and Pigmentation Signaling	5.03E-01	4.12E-02	NaN	ADCY1,RAP2B,RASD2,SRC	
410	Neurotransmitter release cycle	5.01E-01	5.13E-02	NaN	CASK,PPFIA2	
411	NGF-stimulated transcription	5.01E-01	5.13E-02	NaN	ID2,JUND	
412	Pyrimidine Ribonucleotides Interconversion	5.01E-01	5.13E-02	NaN	AK1,AK4	
413	Role of JAK1 and JAK3 in Œ≥c Cytokine Signaling	4.96E-01	4.41E-02	NaN	IRS2,RAP2B,RASD2	
414	GM-CSF Signaling	4.96E-01	4.41E-02	NaN	PRKCB,RAP2B,RASD2	
415	Cytosolic iron-sulfur cluster assembly	4.95E-01	7.69E-02	NaN	CIAO2B	
416	Cleavage and Polyadenylation of Pre-mRNA	4.95E-01	7.69E-02	NaN	PABPN1	
417	Gap junction trafficking and regulation	4.86E-01	5.00E-02	NaN	SRC,TUBB4A	
418	Bile acid and bile salt metabolism	4.86E-01	5.00E-02	NaN	CYP7B1,OSBPL1A	
419	Signaling by FGFR3	4.86E-01	5.00E-02	NaN	SRC,UBA52	
420	Activation of anterior HOX genes in hindbrain during early e	4.85E-01	4.35E-02	NaN	ASH2L,POLR2L,RBBP5	
421	Sensory processing of sound by inner hair cells of the cochle	4.85E-01	4.35E-02	NaN	CABP1,CACNB2,CASK	
422	Growth Hormone Signaling	4.85E-01	4.35E-02	NaN	PRKCA,PRKCB,PRKD1	
423	ILK Signaling	4.83E-01	3.65E-02	-0.816	ACTN4,DSP,FLNB,IRS2,MAPK8,PARVA,RHOB	
424	Mitotic G1 phase and G1/S transition	4.81E-01	3.85E-02	0.447	CCNE1,PSMD1,PSME1,SRC,UBA52	
425	S Phase	4.76E-01	4.00E-02	1	CCNE1,PSMD1,PSME1,UBA52	
426	Glioma Invasiveness Signaling	4.75E-01	4.29E-02	NaN	RAP2B,RASD2,RHOB	
427	IL-10 Signaling	4.74E-01	3.82E-02	-0.447	BHLHE40,DDIT4,HLA-DMA,IL1R1,MAPK8	
428	Phagosome Formation	4.72E-01	3.25E-02	-2.4	ARPC4,DRD5,ELMO2,GPLD1,GPR22,GPRC5B,GRM2,HTR4,IGHM,ITGA7,MAS1,NAPEPLD,NPY1R,NPY5R,	
429					OPRL1,PRKCA,PRKCB,PRKD1,RAP2B,RASD2,SRC	
430	Signaling by FGFR4	4.72E-01	4.88E-02	NaN	SRC,UBA52	
431	Oncostatin M Signaling	4.72E-01	4.88E-02	NaN	RAP2B,RASD2	
432	DNA Double-Strand Break Repair by Non-Homologous End Jo	4.69E-01	7.14E-02	NaN	PARP1	
433	Colanic Acid Building Blocks Biosynthesis	4.69E-01	7.14E-02	NaN	GALT	
434	Guanosine Nucleotides Degradation III	4.69E-01	7.14E-02	NaN	GDA	
435	Osteoarthritis Pathway	4.68E-01	3.54E-02	-0.816	CEBPB,DDIT4,FGFR1,FOXO3,IL1R1,ITGA7,SIK3,TGFBR1	
436	Neuroprotective Role of THOP1 in Alzheimer's Disease	4.67E-01	3.96E-02	NaN	PDYN,PRSS12,SST,THOP1	
437	COPII-mediated vesicle transport	4.64E-01	4.23E-02	NaN	CNIH1,LMAN2,TGFA	
438	Signaling by SCF-KIT	4.58E-01	4.76E-02	NaN	PRKCA,SRC	
439	MyD88:MAL(TIRAP) cascade initiated on plasma membrane	4.58E-01	4.76E-02	NaN	PELI2,UBA52	
440	Regulation of beta-cell development	4.58E-01	4.76E-02	NaN	MAML2,MAMLD1	
441	Vasopressin regulates renal water homeostasis via Aquapori	4.58E-01	4.76E-02	NaN	ADCY1,MYO5B	
442	Formation of WDR5-containing histone-modifying complexe	4.58E-01	4.76E-02	NaN	ASH2L,RBBP5	
443	Pyrimidine Ribonucleotides De Novo Biosynthesis	4.58E-01	4.76E-02	NaN	AK1,AK4	
444	Cilium Assembly	4.58E-01	3.57E-02	1.134	AKAP9,ATAT1,IFT20,LZTFL1,MAPRE1,PKD1,TUBB4A	
445	Signaling by FGFR2	4.54E-01	4.17E-02	NaN	POLR2L,SRC,UBA52	
446	PI3K/AKT Signaling	4.52E-01	3.55E-02	NaN	FOXO3,IL11RA,IL1R1,INPP5J,ITGA7,RAP2B,RASD2	
447	Adrenomedullin signaling pathway	4.52E-01	3.55E-02	0.447	ADCY1,CEBPB,GAD1,MAPK8,PLCH2,RAP2B,RASD2	
448	Mouse Embryonic Stem Cell Pluripotency	4.51E-01	3.88E-02	NaN	APC,ID2,RAP2B,RASD2	
449	TBC/RABGAPs	4.45E-01	4.65E-02	NaN	RAB33A,RAB4A	
450	RUNX1 regulates megakaryocyte differentiation and platelet	4.45E-01	4.65E-02	NaN	ASH2L,RBBP5	
451	Regulation of cholesterol biosynthesis by SREBP (SREBF)	4.45E-01	6.67E-02	NaN	SREBF2	
452	Rap1 signalling	4.45E-01	6.67E-02	NaN	RAP1GAP2	
453	Response of EIF2AK1 (HRI) to heme deficiency	4.45E-01	6.67E-02	NaN	CEBPB	
454	Plasma lipoprotein assembly, remodeling, and clearance	4.45E-01	4.11E-02	NaN	BMP1,LPL,UBA52	
455	Xenobiotic Metabolism PXR Signaling Pathway	4.42E-01	3.59E-02	-0.447	Gstm7,HS6ST1,NDST4,PRKCA,PRKCB,PRKD1	
456	Hereditary Breast Cancer Signaling	4.38E-01	3.68E-02	0	POLR2L,RAP2B,RASD2,TGFA,UBA52	
457	Extra-nuclear estrogen signaling	4.35E-01	4.05E-02	NaN	FOXO3,SRC,TGFA	
458	GPCR-Mediated Integration of Enteroendocrine Signaling Ex	4.35E-01	4.05E-02	NaN	ADCY1,PLCH2,SST	
459	PI3K Cascade	4.33E-01	4.55E-02	NaN	FGFR1,IRS2	
460	Protein Ubiquitination Pathway	4.32E-01	3.40E-02	0	B2M,NEDD4L,PSMD1,PSME1,STUB1,THOP1,UBA52,UBE2J1,USP46	
461	Ephrin A Signaling	4.31E-01	3.65E-02	0.447	EFNA3,EPHA10,EPHA5,SRC,TGFA	
462	BBSome Signaling Pathway	4.28E-01	3.25E-02	-2.84	BDNF,CXCL12,DRD5,GPR22,GPRC5B,GRM2,HTR4,IFT20,LZTFL1,MAS1,NPY1R,NPY5R,OPRL1,PKD1,SSX2IP	
463	Degradation of the extracellular matrix	4.26E-01	4.00E-02	NaN	BMP1,CAPN3,CAPN5	
464	Leptin Signaling in Obesity	4.26E-01	4.00E-02	NaN	ADCY1,NPY,PLCH2	
465	Beta-catenin independent WNT signaling	4.24E-01	3.47E-02	0	GNAO1,PRICKLE1,PRKCA,PRKCB,PSMD1,PSME1,UBA52	
466	Signaling by NOTCH4	4.24E-01	3.62E-02	-0.447	MAML2,MAMLD1,PSMD1,PSME1,UBA52	
467	SUMOylation of DNA methylation proteins	4.23E-01	6.25E-02	NaN	DNMT3A	
468	Granzyme B Signaling	4.23E-01	6.25E-02	NaN	PARP1	
469	Chondroitin Sulfate Degradation (Metazoa)	4.23E-01	6.25E-02	NaN	CEMIP	
470	Superpathway of Citrulline Metabolism	4.23E-01	6.25E-02	NaN	NOS1	
471	tRNA Splicing	4.21E-01	4.44E-02	NaN	NAPEPLD,PDE2A	
472	Integration of energy metabolism	4.19E-01	3.74E-02	-1	ADCY1,CACNB2,KCNG2,PRKCA	
473	Signaling by VEGF	4.19E-01	3.74E-02	-1	ELMO2,PRKCA,PRKCB,SRC	
474	Class I MHC mediated antigen processing and presentation	4.18E-01	3.28E-02	-1.155	B2M,HECTD2,ITCH,NEDD4L,PSMD1,PSME1,RNF19A,SPSB1,STUB1,THOP1,UBA52,UBE2J1	
475	Tumor Microenvironment Pathway	4.17E-01	3.51E-02	0	CXCL12,FOXO3,FOXO6,MMP17,RAP2B,RASD2	
476	Cellular response to hypoxia	4.17E-01	3.95E-02	NaN	PSMD1,PSME1,UBA52	
477	Angiopoietin Signaling	4.17E-01	3.95E-02	NaN	GRB14,RAP2B,RASD2	
478	Role of JAK family kinases in IL-6-type Cytokine Signaling	4.17E-01	3.95E-02	NaN	CEBPD,IL11RA,MAPK8	
479	Nucleotide Excision Repair	4.12E-01	3.70E-02	0	COPS5,PARP1,POLR2L,UBA52	
480	Antioxidant Action of Vitamin C	4.12E-01	3.70E-02	-1	GPLD1,MAPK8,NAPEPLD,PLCH2	
481	Role of Osteoclasts in Rheumatoid Arthritis Signaling Pathwa	4.12E-01	3.31E-02	0	COL22A1,FOXO3,FOXO6,IL1R1,MAPK8,MMP17,RAP2B,RASD2,RHOB,SRC	
482	Actin Cytoskeleton Signaling	4.10E-01	3.38E-02	0.816	ACTN4,APC,ARPC4,DIAPH2,IQGAP2,ITGA7,RAP2B,RASD2	
483	M-decay: degradation of maternal mRNAs by maternally sto	4.09E-01	4.35E-02	NaN	CNOT10,CPEB1	
484	Role of OCT4 in Mammalian Embryonic Stem Cell Pluripoten	4.09E-01	4.35E-02	NaN	ASH2L,PARP1	
485	Dermatan Sulfate Biosynthesis (Late Stages)	4.09E-01	4.35E-02	NaN	HS6ST1,NDST4	
486	TCR signaling	4.04E-01	3.67E-02	0	PSMD1,PSME1,PTPRJ,UBA52	
487	Role of MAPK Signaling in Promoting the Pathogenesis of Inf	4.04E-01	3.67E-02	1	MAPK8,PRKCA,RAP2B,RASD2	
488	NAD Signaling Pathway	4.04E-01	3.55E-02	-1	CEBPB,PARP1,POLR2L,RYR1,TGFA	
489	Signaling by Type 1 Insulin-like Growth Factor 1 Receptor (IG	4.03E-01	5.88E-02	NaN	IRS2	
490	Gastrin-CREB signalling pathway via PKC and MAPK	4.03E-01	5.88E-02	NaN	PRKCA	
491	Azathioprine ADME	4.03E-01	5.88E-02	NaN	GUK1	
492	Dermatan Sulfate Degradation (Metazoa)	4.03E-01	5.88E-02	NaN	CEMIP	
493	D-myo-inositol (1,3,4)-trisphosphate Biosynthesis	4.03E-01	5.88E-02	NaN	INPP5J	
494	Eicosanoid Signaling	3.99E-01	3.31E-02	0.333	ADCY1,GNAO1,PLCH2,PRKCA,PRKCB,PRKD1,RAP2B,RASD2,SRC	
495	RNA polymerase II transcribes snRNA genes	3.99E-01	3.85E-02	NaN	CCNT1,INTS10,POLR2L	
496	Heparan Sulfate Biosynthesis (Late Stages)	3.99E-01	3.85E-02	NaN	GLCE,HS6ST1,NDST4	
497	Complex IV assembly	3.97E-01	4.26E-02	NaN	COX16,SMIM20	
498	G alpha (12/13) signalling events	3.91E-01	3.80E-02	NaN	KALRN,NET1,RHOB	
499	Interferon alpha/beta signaling	3.86E-01	4.17E-02	NaN	IP6K2,UBA52	
500	Chronic Myeloid Leukemia Signaling	3.86E-01	3.27E-02	2.333	DYRK2,FOXO3,HDAC11,ITCH,PLCH2,RAP2B,RASD2,SPRED1,TGFBR1	
501	Cellular hexose transport	3.84E-01	5.56E-02	NaN	SLC2A6	
502	GABA synthesis, release, reuptake and degradation	3.84E-01	5.56E-02	NaN	GAD1	
503	NFE2L2 regulating anti-oxidant/detoxification enzymes	3.84E-01	5.56E-02	NaN	SRXN1	
504	Protein Sorting Signaling Pathway	3.83E-01	3.39E-02	-1.633	ADCY1,GPLD1,NAPEPLD,PRKD1,SRC,VPS29	
505	Estrogen-Dependent Breast Cancer Signaling	3.82E-01	3.75E-02	NaN	RAP2B,RASD2,SRC	
506	Prostate Cancer Signaling	3.76E-01	3.54E-02	2	CCNE1,HDAC11,RAP2B,RASD2	
507	Melanoma Signaling	3.76E-01	4.08E-02	NaN	RAP2B,RASD2	
508	Chondroitin Sulfate Biosynthesis (Late Stages)	3.76E-01	4.08E-02	NaN	HS6ST1,NDST4	
509	Apelin Muscle Signaling Pathway	3.76E-01	4.08E-02	NaN	GNAO1,GNAZ	
510	Signaling by NTRK1 (TRKA)	3.74E-01	3.70E-02	NaN	IRS2,SH3GL2,SRC	

	A	B	C	D	E	F
511	BAG2 Signaling Pathway	3.74E-01	3.70E-02	NaN	PSMD1,PSME1,STUB1	
512	Dilated Cardiomyopathy Signaling Pathway	3.68E-01	3.40E-02	NaN	ADCY1,CACNB2,CACNG3,CAMK1D,PDE2A	
513	KEAP1-NFE2L2 pathway	3.68E-01	3.40E-02	-0.447	CSNK2A2,PSMD1,PSME1,SESN1,UBA52	
514	JAK/STAT Signaling	3.66E-01	3.66E-02	NaN	CEBPB,RAP2B,RASD2	
515	RAR Activation	3.66E-01	3.15E-02	-0.277	ACVR1,ACVR1C,ADCY1,CSNK2A2,FABP5,MAPK8,NAPEPLD,NRIP2,PARP1,PDE2A,RHOB,SRC,TGFBR1	
516	Signaling by Activin	3.66E-01	5.26E-02	NaN	ACVR1C	
517	RHO GTPases activate CIT	3.66E-01	5.26E-02	NaN	RHOB	
518	RHO GTPases Activate ROCKs	3.66E-01	5.26E-02	NaN	RHOB	
519	Purine Nucleotides Degradation II (Aerobic)	3.66E-01	5.26E-02	NaN	GDA	
520	Signaling by TGFBR3	3.66E-01	4.00E-02	NaN	MYCN,TGFBR1	
521	PAK Signaling	3.63E-01	3.48E-02	NaN	ITGA7,MAPK8,RAP2B,RASD2	
522	Autophagy	3.63E-01	3.27E-02	0.378	CAMKK2,DAPK1,FOXO3,IRS2,MAPK8,SESN1,TGFA	
523	PTEN Regulation	3.62E-01	3.38E-02	-0.447	CSNK2A2,PSMD1,PSME1,STUB1,UBA52	
524	eNOS Signaling	3.56E-01	3.36E-02	-0.447	ADCY1,PRKCA,PRKCB,PRKD1,STUB1	
525	PEDF Signaling	3.51E-01	3.57E-02	NaN	BDNF,RAP2B,RASD2	
526	Signaling by Hippo	3.50E-01	5.00E-02	NaN	WWC1	
527	PRC2 methylates histones and DNA	3.50E-01	5.00E-02	NaN	DNMT3A	
528	Synthesis, secretion, and deacylation of Ghrelin	3.50E-01	5.00E-02	NaN	Arxes1/Arxes2	
529	Interleukin-12 family signaling	3.50E-01	5.00E-02	NaN	CRLF1	
530	Endoplasmic Reticulum Stress Pathway	3.50E-01	5.00E-02	NaN	MAPK8	
531	Apelin Liver Signaling Pathway	3.50E-01	5.00E-02	NaN	MAPK8	
532	Lung Ionic Balance Signaling Pathway	3.47E-01	3.09E-02	-1.807	ATP1B2,DRD5,GPR22,GPRC5B,GRM2,HTR4,MAS1,NPY1R,NPY5R,OPRL1,PLCH2,PRKCA,PRKCB,PRKD1,SRC	
533	Production of Nitric Oxide and Reactive Oxygen Species in M	3.47E-01	3.26E-02	-0.816	MAP3K4,MAPK8,PRKCA,PRKCB,PRKD1,RHOB	
534	CSDE1 Signaling Pathway	3.46E-01	3.85E-02	NaN	FABP7,PABPN1	
535	NLR signaling pathways	3.46E-01	3.85E-02	NaN	ITCH,UBA52	
536	IL-15 Production	3.44E-01	3.39E-02	-1	EPHA5,FGFR1,NTRK3,SRC	
537	Gene Silencing by RNA	3.44E-01	3.53E-02	NaN	DROSHA,IPO8,POLR2L	
538	Heparan Sulfate Biosynthesis	3.44E-01	3.53E-02	NaN	GLCE,HS6ST1,NDST4	
539	Sheddase Signaling Pathway	3.42E-01	3.24E-02	-0.816	CEBPB,DDIT4,MAPK8,MMP17,SRC,TGFA	
540	Interleukin-3, Interleukin-5 and GM-CSF signaling	3.37E-01	3.77E-02	NaN	INPP5J,UBA52	
541	FGF Signaling	3.36E-01	3.49E-02	NaN	FGFR1,MAPK8,PRKCA	
542	Growth hormone receptor signaling	3.34E-01	4.76E-02	NaN	IRS2	
543	Pregnenolone Biosynthesis	3.34E-01	4.76E-02	NaN	CYP7B1	
544	Methionine Degradation I (to Homocysteine)	3.34E-01	4.76E-02	NaN	AHCYL2	
545	Regulation of mitotic cell cycle	3.29E-01	3.45E-02	NaN	PSMD1,PSME1,UBA52	
546	Mitotic Prophase	3.29E-01	3.45E-02	NaN	ARPP19,PRKCA,PRKCB	
547	Apelin Adipocyte Signaling Pathway	3.29E-01	3.45E-02	NaN	ADCY1,Gstm7,MAPK8	
548	Mitotic G2-G2/M phases	3.27E-01	3.19E-02	1.633	AKAP9,MAPRE1,PSMD1,PSME1,TUBB4A,UBA52	
549	Synthesis of DNA	3.26E-01	3.31E-02	1	CCNE1,PSMD1,PSME1,UBA52	
550	NGF Signaling	3.26E-01	3.31E-02	NaN	MAP3K4,MAPK8,RAP2B,RASD2	
551	Ovarian Cancer Signaling	3.24E-01	3.23E-02	NaN	APC,HDAC11,RAP2B,RASD2,SRC	
552	DNA Replication Pre-Initiation	3.22E-01	3.41E-02	NaN	PSMD1,PSME1,UBA52	
553	Asparagine N-linked glycosylation	3.20E-01	3.28E-02	0	MGAT3,NAGK,RNF5,UBA52	
554	IL-17A Signaling in Gastric Cells	3.20E-01	4.55E-02	NaN	MAPK8	
555	Histidine Degradation VI	3.20E-01	4.55E-02	NaN	CYP7B1	
556	HMGB1 Signaling	3.19E-01	3.21E-02	1	IL1R1,MAPK8,RAP2B,RASD2,RHOB	
557	Amino acids regulate mTORC1	3.19E-01	3.64E-02	NaN	FNIP2,SESN1	
558	Somitogenesis	3.19E-01	3.64E-02	NaN	PSMD1,PSME1	
559	Role of JAK2 in Hormone-like Cytokine Signaling	3.19E-01	3.64E-02	NaN	IRS2,NPY	
560	Triacylglycerol Degradation	3.19E-01	3.64E-02	NaN	LPL,NDST4	
561	Triacylglycerol Biosynthesis	3.19E-01	3.64E-02	NaN	DGAT2,PLPPR3	
562	SPINK1 General Cancer Pathway	3.19E-01	3.64E-02	NaN	RAP2B,RASD2	
563	FAT10 Signaling Pathway	3.19E-01	3.64E-02	NaN	PSMD1,PSME1	
564	Ferroptosis Signaling Pathway	3.14E-01	3.25E-02	2	GSS,RAP2B,RASD2,SREBF2	
565	Senescence-Associated Secretory Phenotype (SASP)	3.10E-01	3.57E-02	NaN	CEBPB,UBA52	
566	Deadenylation-dependent mRNA decay	3.10E-01	3.57E-02	NaN	CNOT10,LSM6	
567	CD27 Signaling in Lymphocytes	3.10E-01	3.57E-02	NaN	MAP3K4,MAPK8	
568	CNTF Signaling	3.10E-01	3.57E-02	NaN	RAP2B,RASD2	
569	Regulation of the Epithelial Mesenchymal Transition by Grov	3.09E-01	3.12E-02	0.816	FGFR1,ID2,MAPK8,RAP2B,RASD2,TGFBR1	
570	Th2 Pathway	3.09E-01	3.23E-02	0	ACVR1,ACVR1C,HLA-DMA,TGFBR1	
571	Interleukin-7 signaling	3.06E-01	4.35E-02	NaN	IRS2	
572	Pyroptosis	3.06E-01	4.35E-02	NaN	CHMP4B	
573	RAS processing	3.06E-01	4.35E-02	NaN	UBA52	
574	IL-22 Signaling	3.06E-01	4.35E-02	NaN	MAPK8	
575	Cysteine Biosynthesis III (mammalia)	3.06E-01	4.35E-02	NaN	AHCYL2	
576	GP6 Signaling Pathway	3.03E-01	3.20E-02	0	COL22A1,PRKCA,PRKCB,PRKD1	
577	Protein folding	3.02E-01	3.30E-02	NaN	CCNE1,CSNK2A2,TUBB4A	
578	RANK Signaling in Osteoclasts	3.02E-01	3.30E-02	NaN	MAP3K4,MAPK8,SRC	
579	Circadian Clock	3.02E-01	3.51E-02	NaN	UBA52,USP46	
580	Gamma carboxylation, hypusinylation, hydroxylation, and ar	3.02E-01	3.51E-02	NaN	DPH6,DRG2	
581	Polyamine Regulation in Colon Cancer	3.02E-01	3.51E-02	NaN	APC,CSNK2A2	
582	Chondroitin Sulfate Biosynthesis	3.02E-01	3.51E-02	NaN	HS6ST1,NDST4	
583	Regulation of the Epithelial-Mesenchymal Transition Pathwa	3.00E-01	3.09E-02	NaN	APC,FGFR1,ID2,RAP2B,RASD2,TGFBR1	
584	TR/RXR Activation	2.98E-01	3.17E-02	-1	PFKP,SLC16A2,SRC,SREBF2	
585	Visual phototransduction	2.94E-01	3.45E-02	NaN	NAPEPLD,PRKCA	
586	Regulation of RUNX1 Expression and Activity	2.93E-01	4.17E-02	NaN	SRC	
587	Death Receptor Signaling	2.90E-01	3.23E-02	NaN	MAPK8,PARP1,TNFRSF25	
588	Metabolism of polyamines	2.86E-01	3.39E-02	NaN	PSMD1,PSME1	
589	Dermatan Sulfate Biosynthesis	2.86E-01	3.39E-02	NaN	HS6ST1,NDST4	
590	Macrophage Alternative Activation Signaling Pathway	2.85E-01	3.07E-02	-0.447	CEBPB,HLA-DMA,IGHM,IRS2,LPL	
591	FAK Signaling	2.84E-01	2.93E-02	-1.46	ACVR1,ACVR1C,APC,ARPC4,CAPN3,CAPN5,DRD5,EFNA3,GPR22,GPRC5B,GRM2,HTR4,IL11RA,IL1R1	
592					,ITGA7,MAPK8,MAS1,NPY1R,NPY5R,OPRL1,RAP2B,RASD2,SRC,TGFBR1	
593	Activation of Matrix Metalloproteinases	2.81E-01	4.00E-02	NaN	MMP17	
594	Cholesterol biosynthesis	2.81E-01	4.00E-02	NaN	SREBF2	
595	Insulin processing	2.81E-01	4.00E-02	NaN	KIF5A	
596	Incretin synthesis, secretion, and inactivation	2.81E-01	4.00E-02	NaN	Arxes1/Arxes2	
597	Metabolism of cofactors	2.81E-01	4.00E-02	NaN	HPDL	
598	Signaling by Erythropoietin	2.81E-01	4.00E-02	NaN	IRS2	
599	Glutathione Redox Reactions I	2.81E-01	4.00E-02	NaN	Gstm7	
600	Ubiquinol-10 Biosynthesis (Eukaryotic)	2.81E-01	4.00E-02	NaN	CYP7B1	
601	PD-1, PD-L1 cancer immunotherapy pathway	2.78E-01	3.16E-02	NaN	B2M,CSNK2A2,HLA-DMA	
602	rRNA modification in the nucleus and cytosol	2.72E-01	3.28E-02	NaN	NHP2,UTP18	
603	Clathrin-mediated Endocytosis Signaling	2.71E-01	2.99E-02	NaN	ARPC4,CSNK2A2,RAB4A,SH3GL2,SRC,UBA52	
604	LPS/IL-1 Mediated Inhibition of RXR Function	2.71E-01	2.97E-02	NaN	FABP5,FABP7,Gstm7,HS6ST1,IL1R1,MAPK8,NDST4	
605	BMAL1:CLOCK,NPAS2 activates circadian gene expression	2.70E-01	3.85E-02	NaN	BHLHE40	
606	WNT ligand biogenesis and trafficking	2.70E-01	3.85E-02	NaN	VPS29	
607	ATF4 activates genes in response to endoplasmic reticulum s	2.70E-01	3.85E-02	NaN	CEBPB	
608	Class C/3 (Metabotropic glutamate/pheromone receptors)	2.70E-01	3.85E-02	NaN	GRM2	
609	Cardiomyocyte Differentiation via BMP Receptors	2.70E-01	3.85E-02	NaN	ACVR1	
610	Estrogen-mediated S-phase Entry	2.70E-01	3.85E-02	NaN	CCNE1	
611	D-myo-inositol (1,4,5)-Trisphosphate Biosynthesis	2.70E-01	3.85E-02	NaN	PLCH2	
612	Gluconeogenesis I	2.70E-01	3.85E-02	NaN	PGAM2	

	A	B	C	D	E	F
613	Mitochondrial translation	2.67E-01	3.09E-02	NaN	MRPL17,MRPL9,MRPS18C	
614	Small Cell Lung Cancer Signaling	2.67E-01	3.09E-02	NaN	CCNE1,HDAC11,NOS1	
615	Cytosolic sensors of pathogen-associated DNA	2.64E-01	3.23E-02	NaN	POLR2L,UBA52	
616	Pre-NOTCH Expression and Processing	2.64E-01	3.23E-02	NaN	MAML2,MAMLD1	
617	Aldosterone Signaling in Epithelial Cells	2.63E-01	2.98E-02	-0.447	NR3C2,PLCH2,PRKCA,PRKCB,PRKD1	
618	Fc epsilon receptor (FCERI) signaling	2.63E-01	3.01E-02	NaN	MAPK8,PSMD1,PSME1,UBA52	
619	Integrin signaling	2.59E-01	3.70E-02	NaN	SRC	
620	C-type lectin receptors (CLRs)	2.58E-01	2.99E-02	0	PSMD1,PSME1,SRC,UBA52	
621	GPER1 signaling	2.56E-01	3.03E-02	NaN	ADCY1,GNAZ,SRC	
622	Translocation of SLC2A4 (GLUT4) to the plasma membrane	2.51E-01	3.12E-02	NaN	RAB4A,TUBB4A	
623	Regulation of endogenous retroelements	2.51E-01	3.12E-02	NaN	ATF7IP,DNMT3A	
624	ERB2-ERBB3 Signaling	2.51E-01	3.12E-02	NaN	RAP2B,RASD2	
625	Sulfur amino acid metabolism	2.49E-01	3.57E-02	NaN	CSAD	
626	RHO GTPases activate PKNs	2.49E-01	3.57E-02	NaN	RHOB	
627	TNFs bind their physiological receptors	2.49E-01	3.57E-02	NaN	TNFRSF25	
628	SUMOylation of intracellular receptors	2.39E-01	3.45E-02	NaN	NR3C2	
629	mRNA Capping	2.39E-01	3.45E-02	NaN	POLR2L	
630	Processing of Capped Intronless Pre-mRNA	2.39E-01	3.45E-02	NaN	PABPN1	
631	Protein ubiquitination	2.39E-01	3.45E-02	NaN	UBA52	
632	EGR2 and SOX10-mediated initiation of Schwann cell myelin	2.39E-01	3.45E-02	NaN	SREBF2	
633	Collagen biosynthesis and modifying enzymes	2.38E-01	3.03E-02	NaN	BMP1,COL22A1	
634	WNT/Ca+ pathway	2.38E-01	3.03E-02	NaN	PLCH2,PRKCA	
635	ISG15 antiviral mechanism	2.32E-01	2.99E-02	NaN	FLNB,UBA52	
636	TP53 Regulates Transcription of DNA Repair Genes	2.32E-01	2.99E-02	NaN	CCNT1,POLR2L	
637	Interferon gamma signaling	2.32E-01	2.99E-02	NaN	B2M,TRIM2	
638	Cell Cycle: G1/S Checkpoint Regulation	2.32E-01	2.99E-02	NaN	CCNE1,HDAC11	
639	DAP12 interactions	2.30E-01	3.33E-02	NaN	B2M	
640	Signaling by CSF3 (G-CSF)	2.30E-01	3.33E-02	NaN	UBA52	
641	Regulation of CDH11 Expression and Function	2.30E-01	3.33E-02	NaN	CDH11	
642	TNFR2 Signaling	2.21E-01	3.23E-02	NaN	MAPK8	
643	Toll Like Receptor 3 (TLR3) Cascade	2.13E-01	3.12E-02	NaN	UBA52	
644	Thrombin signalling through proteinase activated receptors	2.13E-01	3.12E-02	NaN	SRC	
645	Sialic acid metabolism	2.13E-01	3.12E-02	NaN	ST3GAL1	
646	Signal amplification	2.05E-01	3.03E-02	NaN	SRC	
647	MAPK targets/ Nuclear events mediated by MAP kinases	2.05E-01	3.03E-02	NaN	MAPK8	
648	Oncogene Induced Senescence	1.97E-01	2.94E-02	NaN	UBA52	
649	Transcriptional Regulation by E2F6	1.97E-01	2.94E-02	NaN	YAF2	
650	Nucleotide catabolism	1.97E-01	2.94E-02	NaN	GDA	
651	Transcriptional Regulation by NPAS4	1.97E-01	2.94E-02	NaN	BDNF	
652	4-1BB Signaling in T Lymphocytes	1.97E-01	2.94E-02	NaN	MAPK8	
653	Inhibition of Matrix Metalloproteases	1.97E-01	2.94E-02	NaN	MMP17	
654	Nucleotide Excision Repair Pathway	1.97E-01	2.94E-02	NaN	POLR2L	
655	Pyroptosis Signaling Pathway	0.00E+00	2.50E-02	NaN	FOXO3,IL1R1	
656	IL-13 Signaling Pathway	0.00E+00	2.00E-02	NaN	FOSL2,SRC	
657	SNARE Signaling Pathway	0.00E+00	2.27E-02	NaN	ADCY1,CACNB2,SYT16	
658	CDX Gastrointestinal Cancer Signaling Pathway	0.00E+00	5.18E-03	NaN	BMP1	
659	Immunogenic Cell Death Signaling Pathway	0.00E+00	1.22E-02	NaN	DAPK1	
660	Macrophage Classical Activation Signaling Pathway	0.00E+00	6.41E-03	NaN	HLA-DMA	
661	MicroRNA Biogenesis Signaling Pathway	0.00E+00	2.78E-02	1.342	DROSHA,POLR2L,RAP2B,RASD2,TGFA	
662	Multiple Sclerosis Signaling Pathway	0.00E+00	2.62E-02	1.342	CAPN3,CAPN5,GRIN3A,HLA-DMA,PARP1	
663	Pathogen Induced Cytokine Storm Signaling Pathway	0.00E+00	2.22E-02	-0.378	BHLHE40,COL22A1,CXCL12,HLA-DMA,IL1R1,MAPK8,RYR1	
664	Role of Osteoblasts in Rheumatoid Arthritis Signaling Pathwa	0.00E+00	2.16E-02	0	ACVR1,APC,CXCL12,MMP17,SRC	
665	Ribonucleotide Reductase Signaling Pathway	0.00E+00	2.47E-02	-1	FOXO3,MAPK8,PARP1,SRC	
666	Glucocorticoid Receptor Signaling	0.00E+00	2.90E-02	NaN	B2M,FKBP5,FOXO3,HLA-DMA,IL11RA,IL1R1,KRT12,KRT2,MAPK8,NR3C2,POLR2L,RAP2B,RASD2,SRC,TGFBR1	
667	Natural Killer Cell Signaling	0.00E+00	2.41E-02	0	B2M,MAP3K4,RAP2B,RASD2	
668	Neutrophil Extracellular Trap Signaling Pathway	0.00E+00	2.82E-02	-1.265	COL22A1,IGHM,MAPK8,ORAI2,PLCH2,PPIF,PRKCA,PRKCB,PRKD1,SRC	
669	Chaperone Mediated Autophagy Signaling Pathway	0.00E+00	1.15E-02	0.447	GFAP,ITCH,MMP17,PSME1,STUB1	
670	IL-33 Signaling Pathway	0.00E+00	6.25E-03	NaN	MAPK8	
671	NOD1/2 Signaling Pathway	0.00E+00	6.10E-03	NaN	MAPK8	
672	ABRA Signaling Pathway	0.00E+00	2.30E-02	NaN	JUND,MAPK8	
673	ISGylation Signaling Pathway	0.00E+00	2.25E-02	NaN	FLNB,MAPK8	
674	Microautophagy Signaling Pathway	0.00E+00	2.60E-02	NaN	CHMP4B,COL22A1,PSMD1,PSME1	
675	Aryl Hydrocarbon Receptor Signaling	0.00E+00	2.70E-02	NaN	CCNE1,Gstm7,MAPK8,SRC	
676	p53 Signaling	0.00E+00	2.11E-02	NaN	MAPK8,RPRM	
677	Intrinsic Pathway for Apoptosis	0.00E+00	1.89E-02	NaN	MAPK8	
678	Signaling by Insulin receptor	0.00E+00	2.22E-02	NaN	IRS2	
679	GPVI-mediated activation cascade	0.00E+00	2.86E-02	NaN	RHOB	
680	Extracellular matrix organization	0.00E+00	1.92E-02	NaN	ITGA7,TNR	
681	Phase II - Conjugation of compounds	0.00E+00	1.54E-02	NaN	GSS	
682	Eukaryotic Translation Elongation	0.00E+00	9.62E-03	NaN	UBA52	
683	Telomere Maintenance	0.00E+00	2.70E-02	NaN	NHP2,POLR2L	
684	Mitochondrial biogenesis	0.00E+00	1.64E-02	NaN	USP46	
685	Macroautophagy	0.00E+00	1.54E-02	NaN	CHMP4B	
686	MyD88-independent TLR4 cascade	0.00E+00	2.70E-02	NaN	UBA52	
687	SRP-dependent cotranslational protein targeting to membra	0.00E+00	1.63E-02	NaN	Arxes1/Arxes2,UBA52	
688	Signaling by PDGF	0.00E+00	1.72E-02	NaN	SRC	
689	p75 NTR receptor-mediated signalling	0.00E+00	2.63E-02	-1	KALRN,MAPK8,NET1,UBA52	
690	Metabolism of non-coding RNA	0.00E+00	1.96E-02	NaN	WDR77	
691	Immunoregulatory interactions between a Lymphoid and a r	0.00E+00	1.35E-02	NaN	B2M	
692	trans-Golgi Network Vesicle Budding	0.00E+00	1.33E-02	NaN	SH3GL2	
693	Assembly of collagen fibrils and other multimeric structures	0.00E+00	1.72E-02	NaN	BMP1	
694	Generic Transcription Pathway	0.00E+00	2.63E-02	-1.342	HDAC11,MAML2,MAMLD1,NR3C2,ZNF189	
695	RNA Polymerase II Transcription	0.00E+00	2.74E-02	2	CCNT1,MAGOH,PABPN1,POLR2L	
696	Phase I - Functionalization of compounds	0.00E+00	1.27E-02	NaN	CYP7B1	
697	Integrin cell surface interactions	0.00E+00	1.19E-02	NaN	ITGA7	
698	Selenoamino acid metabolism	0.00E+00	7.81E-03	NaN	UBA52	
699	Activation of gene expression by SREBF (SREBP)	0.00E+00	2.50E-02	NaN	SREBF2	
700	Mitotic Metaphase and Anaphase	0.00E+00	2.68E-02	0.816	CHMP4B,MAPRE1,PSMD1,PSME1,TUBB4A,UBA52	
701	Oxidative Stress Induced Senescence	0.00E+00	2.78E-02	NaN	MAPK8,UBA52	
702	DNA Damage/Telomere Stress Induced Senescence	0.00E+00	2.50E-02	NaN	CCNE1	
703	SUMOylation of DNA damage response and repair proteins	0.00E+00	1.35E-02	NaN	PARP1	
704	Costimulation by the CD28 family	0.00E+00	8.93E-03	NaN	SRC	
705	Regulation of lipid metabolism by PPARalpha	0.00E+00	8.70E-03	NaN	SREBF2	
706	G alpha (q) signalling events	0.00E+00	1.83E-02	NaN	KALRN,RGS17,RGS4	
707	Transport of inorganic cations/anions and amino acids/oligo	0.00E+00	2.83E-02	NaN	AHCYL2,SLC24A2,SLC4A4	
708	Transport of vitamins, nucleosides, and related molecules	0.00E+00	2.63E-02	NaN	SLC16A2	
709	NoRC negatively regulates rRNA expression	0.00E+00	1.96E-02	NaN	POLR2L	
710	Sphingolipid metabolism	0.00E+00	2.91E-02	NaN	PRKD1,SPNS2,SPTLC2	
711	TAK1-dependent IKK and NF-kappa-B activation	0.00E+00	2.38E-02	NaN	UBA52	
712	Interleukin-2 family signaling	0.00E+00	2.27E-02	NaN	INPP5J	
713	SUMOylation of chromatin organization proteins	0.00E+00	1.72E-02	NaN	SATB1	
714	Signaling by Retinoic Acid	0.00E+00	2.56E-02	NaN	FABP5	

	A	B	C	D	E	F
715	TP53 Regulates Metabolic Genes	0.00E+00	2.35E-02	NaN	DDIT4,SESN1	
716	Hedgehog 'on' state	0.00E+00	2.84E-02	0	ITCH,PSMD1,PSME1,UBA52	
717	TP53 Regulates Transcription of Cell Death Genes	0.00E+00	2.63E-02	NaN	BCL6	
718	Deubiquitination	0.00E+00	2.49E-02	0.816	APC,PSMD1,PSME1,TGFBR1,TNIP2,UBA52	
719	HDR through Homologous Recombination (HRR) or Single St	0.00E+00	9.80E-03	NaN	UBA52	
720	Fanconi Anemia Pathway	0.00E+00	2.70E-02	NaN	UBA52	
721	Interleukin-10 signaling	0.00E+00	2.70E-02	NaN	IL1R1	
722	Interleukin-4 and Interleukin-13 signaling	0.00E+00	2.91E-02	NaN	BCL6,CEBPD,FOXO3	
723	Major pathway of rRNA processing in the nucleolus and cytc	0.00E+00	1.68E-02	NaN	RPP14,UBA52,UTP18	
724	Neutrophil degranulation	0.00E+00	2.88E-02	-0.577	B2M,COTL1,DSP,FABP5,IQGAP2,KCNAB2,PGM1,PSMD1,PTPRJ,PYGB,RAP2B,SYNGR1	
725	Regulation of TP53 Expression and Degradation	0.00E+00	2.63E-02	NaN	UBA52	
726	COPI-mediated anterograde transport	0.00E+00	1.08E-02	NaN	TUBB4A	
727	Intra-Golgi and retrograde Golgi-to-ER traffic	0.00E+00	2.63E-02	0.447	CYTH3,CYTH4,KIF26B,KIF5A,TUBB4A	
728	Mitotic Prometaphase	0.00E+00	2.07E-02	1	AKAP9,CSNK2A2,MAPRE1,TUBB4A	
729	Cell Cycle Checkpoints	0.00E+00	2.33E-02	1.633	CCNE1,MAPRE1,PCBP4,PSMD1,PSME1,UBA52	
730	Glucose metabolism	0.00E+00	2.47E-02	NaN	PFKP,PGAM2	
731	Processing of Capped Intron-Containing Pre-mRNA	0.00E+00	1.42E-02	2	LSM6,MAGOH,PABPN1,POLR2L	
732	Eukaryotic Translation Initiation	0.00E+00	7.63E-03	NaN	UBA52	
733	Eukaryotic Translation Termination	0.00E+00	9.71E-03	NaN	UBA52	
734	RNA Polymerase I Transcription	0.00E+00	1.75E-02	NaN	POLR2L	
735	DNA Damage Bypass	0.00E+00	2.08E-02	NaN	UBA52	
736	Resolution of Abasic Sites (AP sites)	0.00E+00	2.63E-02	NaN	PARP1	
737	Response to elevated platelet cytosolic Ca2+	0.00E+00	2.42E-02	NaN	ACTN4,PRKCA,PRKCB	
738	Mitochondrial Fatty Acid Beta-Oxidation	0.00E+00	2.86E-02	NaN	ACADVL	
739	Transcriptional Regulation by VENTX	0.00E+00	2.63E-02	NaN	CEBPB	
740	Transcriptional regulation by the AP-2 (TFAP2) family of tran	0.00E+00	2.86E-02	NaN	TGFA	
741	E3 ubiquitin ligases ubiquitinate target proteins	0.00E+00	2.17E-02	NaN	UBA52	
742	Transcriptional regulation by RUNX2	0.00E+00	1.96E-02	NaN	SRC	
743	Collagen chain trimerization	0.00E+00	2.33E-02	NaN	COL22A1	
744	Neddylation	0.00E+00	2.89E-02	0.378	COPS5,PSMD1,PSME1,RBBP5,SPSB1,TULP4,UBA52	
745	Peroxisomal protein import	0.00E+00	1.67E-02	NaN	UBA52	
746	Iron uptake and transport	0.00E+00	1.69E-02	NaN	UBA52	
747	Nonsense-Mediated Decay (NMD)	0.00E+00	2.68E-02	NaN	MAGOH,UBA52,UPF1	
748	Transcriptional regulation of granulopoiesis	0.00E+00	2.78E-02	NaN	CEBPB	
749	Response of EIF2AK4 (GCN2) to amino acid deficiency	0.00E+00	2.02E-02	NaN	CEBPB,UBA52	
750	Sensory processing of sound by outer hair cells of the cochle	0.00E+00	1.82E-02	NaN	CASK	
751	Heme signaling	0.00E+00	2.22E-02	NaN	USP46	
752	Expression and translocation of olfactory receptors	0.00E+00	1.18E-02	NaN	LHX2	
753	Factors involved in megakaryocyte development and platele	0.00E+00	2.60E-02	NaN	CARMIL1,DOCK9	
754	Acute Phase Response Signaling	0.00E+00	2.84E-02	0	CEBPB,IL1R1,MAPK8,RAP2B,RASD2	
755	LXR/RXR Activation	0.00E+00	1.75E-02	NaN	IL1R1,LPL	
756	Mitochondrial Dysfunction	0.00E+00	2.69E-02	0	ATP1B2,CACNB2,CACNG3,CAPN3,CAPN5,Gstm7,MAPK8,NOS1,PPIF	
757	NAFLD Signaling Pathway	0.00E+00	1.39E-02	NaN	FOXO3,IRS2,MAPK8	
758	FXR/RXR Activation	0.00E+00	2.34E-02	NaN	Gstm7,LPL,MAPK8,SRC	
759	Folate Signaling Pathway	0.00E+00	1.85E-02	NaN	SRC	
760	IL-27 Signaling Pathway	0.00E+00	1.63E-02	NaN	B2M,IL1R1	
761	Sleep REM Signaling Pathway	0.00E+00	9.80E-03	NaN	BDNF	
762	Cohesin Chromatin Regulation Pathway	0.00E+00	2.84E-02	1.633	CDHR1,FAT3,PCDH17,PCDH19,PCDH9,POLR2L	
763	Hematoma Resolution Signaling Pathway	0.00E+00	2.45E-02	-0.816	ATAT1,GRIN3A,MAPK8,MMP17,NOS1,SRC	
764	Ribosomal Quality Control Signaling Pathway	0.00E+00	1.66E-02	NaN	PSMD1,PSME1,UBA52	
765	PKR-mediated signaling	0.00E+00	1.52E-02	NaN	TUBB4A	
766	MITF-M-dependent gene expression	0.00E+00	1.09E-02	NaN	USP46	
767	Complex I biogenesis	0.00E+00	1.49E-02	NaN	NDUFAF2	
768	Cyclophilin Signaling Pathway	0.00E+00	2.12E-02	-0.447	B2M,BDNF,CXCL12,MAPK8,PPIF	
769	Irritable Bowel Syndrome Signaling Pathway	0.00E+00	1.61E-02	-2	IL1R1,MMP17,RHOB,TNFRSF25	
770	NAP1L1 Transcription Regulation Signaling Pathway	0.00E+00	1.28E-02	NaN	TUBB4A	
771	TRIM21 Intracellular Antibody Signaling Pathway	0.00E+00	1.07E-02	0	IGHM,PSMD1,PSME1,UBA52	
772	Tight Junction Signaling	0.00E+00	1.15E-02	NaN	CASK,TGFBR1	
773	Regulation of Actin-based Motility by Rho	0.00E+00	2.80E-02	NaN	ARPC4,ITGA7,RHOB	
774	Activation of IRF by Cytosolic Pattern Recognition Receptors	0.00E+00	1.85E-02	NaN	MAPK8	
775	Role of Pattern Recognition Receptors in Recognition of Bac	0.00E+00	2.82E-02	0	MAPK8,PRKCA,PRKCB,PRKD1	
776	Role of PKR in Interferon Induction and Antiviral Response	0.00E+00	8.33E-03	NaN	MAPK8	
777	MIF Regulation of Innate Immunity	0.00E+00	2.33E-02	NaN	MAPK8	
778	Role of NFAT in Regulation of the Immune Response	0.00E+00	9.26E-03	2	GNAO1,GNAZ,HLA-DMA,IGHM,RAP2B,RASD2	
779	FcŒ≥RIIB Signaling in B Lymphocytes	0.00E+00	1.75E-02	NaN	CACNB2,CACNG3,IGHM,MAPK8,RAP2B,RASD2	
780	CCR5 Signaling in Macrophages	0.00E+00	2.59E-02	0	CACNB2,CACNG3,GNAO1,GNAZ,MAPK8,PRKCA,PRKCB,PRKD1	
781	CD40 Signaling	0.00E+00	1.49E-02	NaN	MAPK8	
782	Calcium-induced T Lymphocyte Apoptosis	0.00E+00	1.53E-02	0	HLA-DMA,PRKCA,PRKCB,PRKD1	
783	Cytotoxic T Lymphocyte-mediated Apoptosis of Target Cells	0.00E+00	4.26E-03	NaN	B2M	
784	IL-17 Signaling	0.00E+00	2.50E-02	1	CEBPB,MAPK8,RAP2B,RASD2	
785	CTLA4 Signaling in Cytotoxic T Lymphocytes	0.00E+00	2.02E-02	-1.414	B2M,GPLD1,HLA-DMA,MAPK8,NAPEPLD,RAP2B,RASD2,SRC	
786	Induction of Apoptosis by HIV1	0.00E+00	1.59E-02	NaN	MAPK8	
787	B Cell Activating Factor Signaling	0.00E+00	2.33E-02	NaN	MAPK8	
788	T Helper Cell Differentiation	0.00E+00	1.10E-02	NaN	BCL6,HLA-DMA,TGFBR1	
789	IL-9 Signaling	0.00E+00	2.86E-02	NaN	IRS2	
790	CD28 Signaling in T Helper Cells	0.00E+00	9.37E-03	NaN	ARPC4,HLA-DMA,MAPK8	
791	IL-15 Signaling	0.00E+00	8.85E-03	NaN	IGHM,RAP2B,RASD2	
792	Dendritic Cell Maturation	0.00E+00	1.33E-02	2	B2M,HLA-DMA,IGHM,MAPK8,PLCH2	
793	Cellular Effects of Sildenafil (Viagra)	0.00E+00	2.74E-02	0.943	ACVR1,ACVR1C,ADCY1,CACNB2,CACNG3,CAPN3,CAPN5,DRD5,GPR22,GPRC5B,GRM2,HTR4,	
794					MAPK8,MAS1,NPY1R,NPY5R,OPRL1,TGFBR1	
795	ICOS-ICOSL Signaling in T Helper Cells	0.00E+00	6.45E-03	NaN	HLA-DMA,PLEKHA2	
796	Lipid Antigen Presentation by CD1	0.00E+00	4.48E-03	NaN	B2M	
797	Cell Cycle Regulation by BTG Family Proteins	0.00E+00	2.70E-02	NaN	CCNE1	
798	Maturity Onset Diabetes of Young (MODY) Signaling	0.00E+00	2.74E-02	NaN	CACNB2,CACNG3	
799	FLT3 Signaling in Hematopoietic Progenitor Cells	0.00E+00	2.47E-02	NaN	RAP2B,RASD2	
800	ATM Signaling	0.00E+00	1.03E-02	NaN	MAPK8	
801	Type I Diabetes Mellitus Signaling	0.00E+00	1.30E-02	NaN	GAD1,HLA-DMA,IL1R1,MAPK8	
802	Basal Cell Carcinoma Signaling	0.00E+00	2.90E-02	NaN	APC,BMP1	
803	Primary Immunodeficiency Signaling	0.00E+00	2.17E-02	NaN	IGHM	
804	Allograft Rejection Signaling	0.00E+00	1.12E-02	NaN	B2M,HLA-DMA,IGHM	
805	Autoimmune Thyroid Disease Signaling	0.00E+00	8.10E-03	NaN	HLA-DMA,IGHM	
806	Acute Myeloid Leukemia Signaling	0.00E+00	2.20E-02	NaN	RAP2B,RASD2	
807	Graft-versus-Host Disease Signaling	0.00E+00	4.15E-03	NaN	HLA-DMA	
808	p70S6K Signaling	0.00E+00	2.05E-02	0	IGHM,PLCH2,PRKCA,PRKCB,PRKD1,RAP2B,RASD2,SRC	
809	G Protein Signaling Mediated by Tubby	0.00E+00	7.12E-03	NaN	GNAO1,GNAZ	
810	Communication between Innate and Adaptive Immune Cells	0.00E+00	3.69E-03	NaN	B2M,IGHM	
811	Sphingosine-1-phosphate Signaling	0.00E+00	2.68E-02	NaN	ADCY1,PLCH2,RHOB	
812	Systemic Lupus Erythematosus Signaling	0.00E+00	6.23E-03	NaN	IGHM,LSM6,RAP2B,RASD2	
813	CDC42 Signaling	0.00E+00	2.22E-02	-0.447	APC,ARPC4,B2M,HLA-DMA,IQGAP2,ITGA7,MAPK8,SRC	
814	EIF2 Signaling	0.00E+00	1.84E-02	NaN	MYCN,RAP2B,RASD2,UBA52	
815	April Mediated Signaling	0.00E+00	2.38E-02	NaN	MAPK8	
816	Retinoic acid Mediated Apoptosis Signaling	0.00E+00	2.27E-02	NaN	PARP1	

	A	B	C	D	E	F
817	RHOA Signaling	0.00E+00	2.50E-02	NaN	ARHGAP12,ARPC4,CDC42EP4	
818	Role of Osteoblasts, Osteoclasts and Chondrocytes in Rheum	0.00E+00	2.71E-02	NaN	ACVR1,APC,BMP1,IL1R1,MAPK8,SRC	
819	Phospholipase C Signaling	0.00E+00	1.78E-02	-0.378	ADCY1,GPLD1,HDAC11,IGHM,ITGA7,NAPEPLD,PRKCA,PRKCB,PRKD1,RAP2B,RASD2,RHOB,SRC	
820	Altered T Cell and B Cell Signaling in Rheumatoid Arthritis	0.00E+00	3.64E-03	NaN	HLA-DMA,IGHM	
821	Atherosclerosis Signaling	0.00E+00	1.71E-02	NaN	CXCL12,LPL	
822	Regulation of eIF4 and p70S6K Signaling	0.00E+00	1.69E-02	NaN	ITGA7,RAP2B,RASD2	
823	B Cell Development	0.00E+00	6.92E-03	NaN	HLA-DMA,IGHM	
824	Regulation of IL-2 Expression in Activated and Anergic T Lym	0.00E+00	1.67E-02	0.447	MAPK8,RAP2B,RASD2,TGFBR1,TOB1	
825	Granzyme A Signaling	0.00E+00	1.43E-02	NaN	PARP1	
826	Role of WNT/GSK-3Œ≤ Signaling in the Pathogenesis of Influ	0.00E+00	1.54E-02	NaN	APC	
827	TWEAK Signaling	0.00E+00	2.78E-02	NaN	TNFRSF25	
828	NUR77 Signaling in T Lymphocytes	0.00E+00	1.66E-02	NaN	B2M,HLA-DMA,PRKCA,PRKCB,PRKD1	
829	PKCŒ∏ Signaling in T Lymphocytes	0.00E+00	1.95E-02	1	CACNB2,CACNG3,HLA-DMA,MAP3K4,MAPK8,RAP2B,RASD2	
830	TNFR1 Signaling	0.00E+00	2.00E-02	NaN	MAPK8	
831	Antiproliferative Role of TOB in T Cell Signaling	0.00E+00	1.26E-02	NaN	CCNE1,TGFBR1,TOB1	
832	OX40 Signaling Pathway	0.00E+00	1.12E-02	NaN	B2M,HLA-DMA,MAPK8	
833	PI3K Signaling in B Lymphocytes	0.00E+00	2.00E-02	NaN	FOXO3,IGHM,IRS2,PLCH2,PLEKHA2,PRKCB,RAP2B,RASD2	
834	Cyclins and Cell Cycle Regulation	0.00E+00	2.41E-02	NaN	CCNE1,HDAC11	
835	Cell Cycle Control of Chromosomal Replication	0.00E+00	1.85E-02	NaN	CDK18	
836	Assembly of RNA Polymerase II Complex	0.00E+00	2.08E-02	NaN	POLR2L	
837	Spliceosomal Cycle	0.00E+00	1.92E-02	NaN	MAGOH	
838	IL-17A Signaling in Airway Cells	0.00E+00	1.61E-02	NaN	MAPK8	
839	Role of IL-17A in Arthritis	0.00E+00	1.96E-02	NaN	MAPK8	
840	Telomerase Signaling	0.00E+00	2.83E-02	NaN	HDAC11,RAP2B,RASD2	
841	Hematopoiesis from Pluripotent Stem Cells	0.00E+00	4.10E-03	NaN	IGHM	
842	tRNA Charging	0.00E+00	2.63E-02	NaN	LARS2	
843	Retinol Biosynthesis	0.00E+00	2.38E-02	NaN	LPL	
844	Superpathway of Methionine Degradation	0.00E+00	2.78E-02	NaN	AHCYL2	
845	DNA damage-induced 14-3-3œÉ Signaling	0.00E+00	2.38E-02	NaN	CCNE1	
846	Agranulocyte Adhesion and Diapedesis	0.00E+00	1.63E-02	NaN	CXCL12,IL1R1,MMP17	
847	Granulocyte Adhesion and Diapedesis	0.00E+00	2.42E-02	NaN	CXCL12,IL1R1,MMP17,SDC3	
848	TEC Kinase Signaling	0.00E+00	2.60E-02	-1.134	GNAO1,GNAZ,ITGA7,MAPK8,PRKCA,PRKCB,PRKD1,RHOB,SRC,TNFRSF25	
849	BER (Base Excision Repair) Pathway	0.00E+00	2.33E-02	NaN	PARP1	
850	HIPPO signaling	0.00E+00	2.33E-02	NaN	ITCH,WWC1	
851	Parkinson's Signaling Pathway	0.00E+00	2.43E-02	1.633	CACNB2,CACNG3,GRIN3A,MAPK8,NOS1,TUBB4A,UBA52	
852	Toll-like Receptor Signaling	0.00E+00	2.70E-02	NaN	MAPK8,UBA52	
853	SAPK/JNK Signaling	0.00E+00	1.27E-02	NaN	MAP3K4,MAPK8,RAP2B,RASD2	
854	Cell Cycle: G2/M DNA Damage Checkpoint Regulation	0.00E+00	2.00E-02	NaN	RPRM	
855	IL-4 Signaling	0.00E+00	1.35E-02	1.342	COL22A1,HLA-DMA,IRS2,RAP2B,RASD2	
856	B Cell Receptor Signaling	0.00E+00	1.81E-02	0.816	BCL6,IGHM,INPP5J,MAP3K4,MAPK8,PRKCB,RAP2B,RASD2	
857	PPAR Signaling	0.00E+00	2.86E-02	NaN	IL1R1,RAP2B,RASD2	
858	Dopamine Receptor Signaling	0.00E+00	2.60E-02	NaN	ADCY1,DRD5	
859	p38 MAPK Signaling	0.00E+00	1.72E-02	NaN	IL1R1,TGFBR1	
860	Notch Signaling	0.00E+00	2.63E-02	NaN	MAML2	
861	NF-Œ∫B Signaling	0.00E+00	2.65E-02	-0.333	CSNK2A2,FGFR1,IL1R1,MAPK8,NTRK3,PRKCB,RAP2B,RASD2,TGFA,TGFBR1	
862	Hypoxia Signaling in the Cardiovascular System	0.00E+00	2.63E-02	NaN	COPS5,UBE2J1	
863	T Cell Receptor Signaling	0.00E+00	1.47E-02	2.449	B2M,FOXO3,HLA-DMA,MAPK8,RAP2B,RASD2	
864	Phagosome Maturation	0.00E+00	2.65E-02	NaN	B2M,DYNLT3,NOS1,TUBB4A	
865	Th1 and Th2 Activation Pathway	0.00E+00	2.56E-02	NaN	ACVR1,ACVR1C,HLA-DMA,TGFBR1	
866	Th1 Pathway	0.00E+00	9.26E-03	NaN	HLA-DMA	
867	Sirtuin Signaling Pathway	0.00E+00	1.81E-02	0.447	FOXO3,MYCN,PARP1,PGAM2,PPIF	
868	Iron homeostasis signaling pathway	0.00E+00	1.49E-02	NaN	BMP1,CIAO2B	
869	Th17 Activation Pathway	0.00E+00	3.60E-03	NaN	IL1R1	
870	SPINK1 Pancreatic Cancer Pathway	0.00E+00	1.96E-02	NaN	TGFBR1	
871	NER (Nucleotide Excision Repair, Enhanced Pathway)	0.00E+00	2.27E-02	NaN	COPS5,POLR2L	
872	Apelin Pancreas Signaling Pathway	0.00E+00	2.22E-02	NaN	MAPK8	
873	T Cell Exhaustion Signaling Pathway	0.00E+00	2.29E-02	1	ACVR1,ACVR1C,BCL6,HLA-DMA,MAPK8,RAP2B,RASD2,TGFBR1	
874	Systemic Lupus Erythematosus in T Cell Signaling Pathway	0.00E+00	1.89E-02	0	B2M,BCL6,HLA-DMA,NOS1,RAB4A,RAP2B,RASD2,RHOB	
875	Systemic Lupus Erythematosus in B Cell Signaling Pathway	0.00E+00	1.95E-02	0	FOXO3,FOXO6,IGHM,INPP5J,PRKCA,PRKCB,PRKD1,RAP2B,RASD2,SRC	
876	Inhibition of ARE-Mediated mRNA Degradation Pathway	0.00E+00	2.55E-02	NaN	CNOT10,PABPN1,PSMD1,PSME1	
877	HOTAIR Regulatory Pathway	0.00E+00	1.27E-02	NaN	ATXN1,MMP17	
878	BEX2 Signaling Pathway	0.00E+00	1.25E-02	NaN	MAPK8	
879	Necroptosis Signaling Pathway	0.00E+00	2.78E-02	0	CAPN3,CAPN5,DAPK1,PPIF	
880	Xenobiotic Metabolism AHR Signaling Pathway	0.00E+00	1.27E-02	NaN	Gstm7	
881	Semaphorin Neuronal Repulsive Signaling Pathway	0.00E+00	2.80E-02	NaN	CSPG5,ITGA7,PLXNA2,SEMA4D	
882	Regulation of the Epithelial Mesenchymal Transition in Deve	0.00E+00	1.18E-02	NaN	APC	
883	Kinetochore Metaphase Signaling Pathway	0.00E+00	9.26E-03	NaN	ARPP19	
884	Coronavirus Pathogenesis Pathway	0.00E+00	2.13E-02	0	CCNE1,HDAC11,MAPK8,TGFBR1	
885	Coronavirus Replication Pathway	0.00E+00	2.21E-02	NaN	Tubb2b,TUBB4A,UBA52	
886	MSP-RON Signaling in Cancer Cells Pathway	0.00E+00	2.17E-02	NaN	RAP2B,RASD2,SRC	
887	MSP-RON Signaling in Macrophages Pathway	0.00E+00	2.88E-02	NaN	HLA-DMA,RAP2B,RASD2	
888	Role of MAPK Signaling in Inhibiting the Pathogenesis of Infl	0.00E+00	1.39E-02	NaN	MAPK8	
889						

	A	B	C	D	E	F	G	H	I
1	**Supplementary Table 6- Syngo CA1**								
2									
3									
4	**your genelist input**	**gene hgnc_id**	**gene symbol**	**gene name**	**gene synonyms**	**GO term ID**	**GO term name**	**GO domain**	**SynGO annotation ID**
5	PRKCA	HGNC:9393	PRKCA	protein kinase C alpha	PKCA,PKCA	GO:0099523	presynaptic cytosol (GO:0099523)	CC	68
6	PRKCA	HGNC:9393	PRKCA	protein kinase C alpha	PKCA,PKCA	GO:2000300	regulation of synaptic vesicle exocytosis (GO:2000300)	BP	70
7	ADCY1	HGNC:232	ADCY1	adenylate cyclase 1	AC1,DFNB44	GO:0099061	integral component of postsynaptic density membrane (GO:0099061)	CC	321
8	ADCY1	HGNC:232	ADCY1	adenylate cyclase 1	AC1,DFNB44	GO:0099171	presynaptic modulation of chemical synaptic transmission (GO:0099171)	BP	324
9	ADCY1	HGNC:232	ADCY1	adenylate cyclase 1	AC1,DFNB44	GO:0050804	modulation of chemical synaptic transmission (GO:0050804)	BP	471
10	FXYD6	HGNC:4030	FXYD6	FXYD domain containing ion transport regulator 6		GO:0099056	integral component of presynaptic membrane (GO:0099056)	CC	730
11	FXYD6	HGNC:4030	FXYD6	FXYD domain containing ion transport regulator 6		GO:0099055	integral component of postsynaptic membrane (GO:0099055)	CC	731
12	SLC6A7	HGNC:11054	SLC6A7	solute carrier family 6 member 7	PROT	GO:0030285	integral component of synaptic vesicle membrane (GO:0030285)	CC	779
13	SLC6A7	HGNC:11054	SLC6A7	solute carrier family 6 member 7	PROT	GO:0099056	integral component of presynaptic membrane (GO:0099056)	CC	780
14	SLC6A7	HGNC:11054	SLC6A7	solute carrier family 6 member 7	PROT	SYNGO:metabolisr	metabolism	BP	783
15	ARPC2	HGNC:705	ARPC2	actin related protein 2/3 complex subunit 2	P34-ARC,ARC34	GO:0045202	synapse (GO:0045202)	CC	804
16	MARCKSL1	HGNC:7142	MARCKSL1	MARCKS like 1	F52,MACMARCKS,MLP1,MLP	GO:0099026	anchored component of presynaptic membrane (GO:0099026)	CC	831
17	MARCKSL1	HGNC:7142	MARCKSL1	MARCKS like 1	F52,MACMARCKS,MLP1,MLP	GO:0008021	synaptic vesicle (GO:0008021)	CC	832
18	MARCKSL1	HGNC:7142	MARCKSL1	MARCKS like 1	F52,MACMARCKS,MLP1,MLP	GO:0099523	presynaptic cytosol (GO:0099523)	CC	833
19	MARCKSL1	HGNC:7142	MARCKSL1	MARCKS like 1	F52,MACMARCKS,MLP1,MLP	GO:0099509	regulation of presynaptic cytosolic calcium levels (GO:0099509)	BP	834
20	DTNBP1	HGNC:17328	DTNBP1	dystrobrevin binding protein 1	DYSBINDIN,MY031,HPS7,DBND,BLOC1S8	GO:0030672	synaptic vesicle membrane (GO:0030672)	CC	866
21	DTNBP1	HGNC:17328	DTNBP1	dystrobrevin binding protein 1	DYSBINDIN,MY031,HPS7,DBND,BLOC1S8	GO:0014069	postsynaptic density (GO:0014069)	CC	867
22	CALB1	HGNC:1434	CALB1	calbindin 1	CALB	GO:0099524	postsynaptic cytosol (GO:0099524)	CC	883
23	CALB1	HGNC:1434	CALB1	calbindin 1	CALB	GO:0099523	presynaptic cytosol (GO:0099523)	CC	884
24	CALB1	HGNC:1434	CALB1	calbindin 1	CALB	GO:0099534	calcium ion binding involved in regulation of presynaptic cytosolic calcium levels (GO:0(	BP	885
25	PFN1	HGNC:8881	PFN1	profilin 1		GO:0098793	presynapse (GO:0098793)	CC	890
26	PFN1	HGNC:8881	PFN1	profilin 1		GO:0098794	postsynapse (GO:0098794)	CC	891
27	PENK	HGNC:8831	PENK	proenkephalin		GO:0099013	neuronal dense core vesicle lumen (GO:0099013)	CC	963
28	PENK	HGNC:8831	PENK	proenkephalin		GO:0034592	synaptic vesicle lumen (GO:0034592)	CC	965
29	PENK	HGNC:8831	PENK	proenkephalin		GO:0099538	synaptic signaling via neuropeptide (GO:0099538)	BP	975
30	NEFM	HGNC:7734	NEFM	neurofilament medium	NFM,NF-M,NEF3	GO:0099160	postsynaptic intermediate filament cytoskeleton (GO:0099160)	CC	997
31	NEFM	HGNC:7734	NEFM	neurofilament medium	NFM,NF-M,NEF3	GO:0099182	presynaptic intermediate filament cytoskeleton (GO:0099182)	CC	1002
32	SPARCL1	HGNC:11220	SPARCL1	SPARC like 1	MAST9	GO:0098965	extracellular matrix of synaptic cleft (GO:0098965)	CC	1003
33	SPARCL1	HGNC:11220	SPARCL1	SPARC like 1	MAST9	GO:0099560	synapse adhesion between pre- and post-synapse (GO:0099560)	BP	1004
34	WLS	HGNC:30238	WLS	Wnt ligand secretion mediator	FLJ23091,MRP,EVI,MIG-14,C1ORF139,GPR177	GO:0098794	postsynapse (GO:0098794)	CC	1023
35	WLS	HGNC:30238	WLS	Wnt ligand secretion mediator	FLJ23091,MRP,EVI,MIG-14,C1ORF139,GPR177	GO:0099056	integral component of presynaptic membrane (GO:0099056)	CC	1029
36	APH1A	HGNC:29509	APH1A	aph-1 homolog A, gamma-secretase subunit	APH-1A,CGI-78	GO:0099056	integral component of presynaptic membrane (GO:0099056)	CC	1034
37	WLS	HGNC:30238	WLS	Wnt ligand secretion mediator	FLJ23091,MRP,EVI,MIG-14,C1ORF139,GPR177	GO:0099157	trans-synaptic signalling via exosome (GO:0099157)	BP	1036
38	PCDH17	HGNC:14267	PCDH17	protocadherin 17	PCDH68,PCH68	GO:0099056	integral component of presynaptic membrane (GO:0099056)	CC	1084
39	SLC6A7	HGNC:11054	SLC6A7	solute carrier family 6 member 7	PROT	GO:0030285	integral component of synaptic vesicle membrane (GO:0030285)	CC	1173
40	EPS15	HGNC:3419	EPS15	epidermal growth factor receptor pathway substrate 15	AF-1P,MLLT5	GO:0048488	synaptic vesicle endocytosis (GO:0048488)	BP	1176
41	PCDH17	HGNC:14267	PCDH17	protocadherin 17	PCDH68,PCH68	GO:0099055	integral component of postsynaptic membrane (GO:0099055)	CC	1323
42	SLC6A7	HGNC:11054	SLC6A7	solute carrier family 6 member 7	PROT	SYNGO:metabolisr	metabolism	BP	1360
43	EPS15	HGNC:3419	EPS15	epidermal growth factor receptor pathway substrate 15	AF-1P,MLLT5	GO:0098894	extrinsic component of presynaptic endocytic zone membrane (GO:0098894)	CC	1363
44	DOC2B	HGNC:2986	DOC2B	double C2 domain beta	DOC2BL	GO:0098850	extrinsic component of synaptic vesicle membrane (GO:0098850)	CC	1397
45	DOC2B	HGNC:2986	DOC2B	double C2 domain beta	DOC2BL	GO:0099502	calcium-dependent activation of synaptic vesicle fusion (GO:0099502)	BP	1399
46	DOC2B	HGNC:2986	DOC2B	double C2 domain beta	DOC2BL	GO:0099502	calcium-dependent activation of synaptic vesicle fusion (GO:0099502)	BP	1402
47	KCNMB4	HGNC:6289	KCNMB4	potassium calcium-activated channel subfamily M regulatory beta subur		GO:0099508	voltage-gated ion channel activity involved in regulation of presynaptic membrane pote	BP	1448
48	HTR1A	HGNC:5286	HTR1A	5-hydroxytryptamine receptor 1A	5-HT1A,ADRB2RL1,ADRBRL1	GO:0099056	integral component of presynaptic membrane (GO:0099056)	CC	1482
49	CALB1	HGNC:1434	CALB1	calbindin 1	CALB	GO:0098794	postsynapse (GO:0098794)	CC	1624
50	PFN1	HGNC:8881	PFN1	profilin 1		GO:0060074	synapse maturation (GO:0060074)	BP	1663
51	ADGRA1	HGNC:13838	ADGRA1	adhesion G protein-coupled receptor A1	KIAA1828,GPR123	GO:0014069	postsynaptic density (GO:0014069)	CC	1664
52	GAP43	HGNC:4140	GAP43	growth associated protein 43	B-50,PP46,GAP-43	GO:0099150	regulation of postsynaptic specialization assembly (GO:0099150)	BP	1720
53	PCDH17	HGNC:14267	PCDH17	protocadherin 17	PCDH68,PCH68	GO:0099560	synapse adhesion between pre- and post-synapse (GO:0099560)	BP	1740
54	PFN1	HGNC:8881	PFN1	profilin 1		GO:0098885	modification of postsynaptic actin cytoskeleton (GO:0098885)	BP	1790
55	PTN	HGNC:9630	PTN	pleiotrophin	HBNF,HBGF8,NEGF1	GO:0098793	presynapse (GO:0098793)	CC	1901
56	PTN	HGNC:9630	PTN	pleiotrophin	HBNF,HBGF8,NEGF1	GO:0098794	postsynapse (GO:0098794)	CC	1928
57	HTR1A	HGNC:5286	HTR1A	5-hydroxytryptamine receptor 1A	5-HT1A,ADRB2RL1,ADRBRL1	GO:0099171	presynaptic modulation of chemical synaptic transmission (GO:0099171)	BP	1944
58	SPARCL1	HGNC:11220	SPARCL1	SPARC like 1	MAST9	GO:0050807	regulation of synapse organization (GO:0050807)	BP	1966
59	CACNG2	HGNC:1406	CACNG2	calcium voltage-gated channel auxiliary subunit gamma 2	STARGAZIN,MGC138502,MGC138504	GO:0099061	integral component of postsynaptic density membrane (GO:0099061)	CC	2045
60	DAGLA	HGNC:1165	DAGLA	diacylglycerol lipase alpha	KIAA0659,NSDDR,DAGLALPHA,C11ORF11	GO:0098921	retrograde trans-synaptic signaling by endocannabinoid (GO:0098921)	BP	2114
61	DAGLA	HGNC:1165	DAGLA	diacylglycerol lipase alpha	KIAA0659,NSDDR,DAGLALPHA,C11ORF11	GO:0099055	integral component of postsynaptic membrane (GO:0099055)	CC	2136
62	CALB1	HGNC:1434	CALB1	calbindin 1	CALB	GO:0099567	calcium ion binding involved in regulation of postsynaptic cytosolic calcium levels (GO:(	BP	2289
63	SHISA6	HGNC:34491	SHISA6	shisa family member 6	FLJ45455	GO:0099061	integral component of postsynaptic density membrane (GO:0099061)	CC	2342
64	SHISA6	HGNC:34491	SHISA6	shisa family member 6	FLJ45455	GO:0098970	postsynaptic neurotransmitter receptor diffusion trapping (GO:0098970)	BP	2343
65	SHISA6	HGNC:34491	SHISA6	shisa family member 6	FLJ45455	GO:0098962	regulation of postsynaptic neurotransmitter receptor activity (GO:0098962)	BP	2350
66	DTNBP1	HGNC:17328	DTNBP1	dystrobrevin binding protein 1	DYSBINDIN,MY031,HPS7,DBND,BLOC1S8	GO:2000300	regulation of synaptic vesicle exocytosis (GO:2000300)	BP	2383
67	CACNB2	HGNC:1402	CACNB2	calcium voltage-gated channel auxiliary subunit beta 2	MYSB,CACNLB2	GO:0099626	voltage-gated calcium channel activity involved in regulation of presynaptic cytosolic ca	BP	2420
68	CACNG2	HGNC:1406	CACNG2	calcium voltage-gated channel auxiliary subunit gamma 2	STARGAZIN,MGC138502,MGC138504	GO:0099072	regulation of postsynaptic membrane neurotransmitter receptor levels (GO:0099072)	BP	2502
69	PPP1CC	HGNC:9283	PPP1CC	protein phosphatase 1 catalytic subunit gamma	PP1C,PP1GAMMA	GO:0098794	postsynapse (GO:0098794)	CC	2515
70	PPP1CC	HGNC:9283	PPP1CC	protein phosphatase 1 catalytic subunit gamma	PP1C,PP1GAMMA	GO:0098793	presynapse (GO:0098793)	CC	2516
71	NR3C2	HGNC:7979	NR3C2	nuclear receptor subfamily 3 group C member 2	MR,MLR	GO:0099092	postsynaptic density, intracellular component (GO:0099092)	CC	2545
72	NR3C1	HGNC:7978	NR3C1	nuclear receptor subfamily 3 group C member 1	GR,GRL	GO:0099092	postsynaptic density, intracellular component (GO:0099092)	CC	2546
73	NR3C2	HGNC:7979	NR3C2	nuclear receptor subfamily 3 group C member 2	MR,MLR	GO:0098831	presynaptic active zone cytoplasmic component (GO:0098831)	CC	2547
74	NR3C2	HGNC:7979	NR3C2	nuclear receptor subfamily 3 group C member 2	MR,MLR	GO:0099171	presynaptic modulation of chemical synaptic transmission (GO:0099171)	BP	2548
75	NR3C2	HGNC:7979	NR3C2	nuclear receptor subfamily 3 group C member 2	MR,MLR	GO:0060078	regulation of postsynaptic membrane potential (GO:0060078)	BP	2549
76	SLITRK1	HGNC:20297	SLITRK1	SLIT and NTRK like family member 1	KIAA1910,LRRC12	GO:0099061	integral component of postsynaptic density membrane (GO:0099061)	CC	2586
77	SLITRK1	HGNC:20297	SLITRK1	SLIT and NTRK like family member 1	KIAA1910,LRRC12	GO:0099560	synapse adhesion between pre- and post-synapse (GO:0099560)	BP	2611
78	SLITRK1	HGNC:20297	SLITRK1	SLIT and NTRK like family member 1	KIAA1910,LRRC12	GO:1905606	regulation of presynapse assembly (GO:1905606)	BP	2614
79	SLITRK1	HGNC:20297	SLITRK1	SLIT and NTRK like family member 1	KIAA1910,LRRC12	GO:0050807	regulation of synapse organization (GO:0050807)	BP	2619
80	GRIN3A	HGNC:16767	GRIN3A	glutamate ionotropic receptor NMDA type subunit 3A	GLUN3A	GO:0099171	presynaptic modulation of chemical synaptic transmission (GO:0099171)	BP	2656
81	GRIN3A	HGNC:16767	GRIN3A	glutamate ionotropic receptor NMDA type subunit 3A	GLUN3A	GO:1904315	transmitter-gated ion channel activity involved in regulation of postsynaptic membrane	BP	2658
82	GRIN3A	HGNC:16767	GRIN3A	glutamate ionotropic receptor NMDA type subunit 3A	GLUN3A	GO:0099061	integral component of postsynaptic density membrane (GO:0099061)	CC	2659
83	ADD2	HGNC:244	ADD2	adducin 2	ADDB	GO:0007416	synapse assembly (GO:0007416)	BP	2687
84	ADD2	HGNC:244	ADD2	adducin 2	ADDB	GO:0098794	postsynapse (GO:0098794)	CC	2688
85	ADD2	HGNC:244	ADD2	adducin 2	ADDB	GO:0098793	presynapse (GO:0098793)	CC	2689
86	ADD2	HGNC:244	ADD2	adducin 2	ADDB	GO:0098794	postsynapse (GO:0098794)	CC	2690
87	CACNG2	HGNC:1406	CACNG2	calcium voltage-gated channel auxiliary subunit gamma 2	STARGAZIN,MGC138502,MGC138504	GO:0098970	postsynaptic neurotransmitter receptor diffusion trapping (GO:0098970)	BP	2697
88	SLITRK1	HGNC:20297	SLITRK1	SLIT and NTRK like family member 1	KIAA1910,LRRC12	GO:0099061	integral component of postsynaptic density membrane (GO:0099061)	CC	2698
89	CACNG2	HGNC:1406	CACNG2	calcium voltage-gated channel auxiliary subunit gamma 2	STARGAZIN,MGC138502,MGC138504	GO:0099645	neurotransmitter receptor localization to postsynaptic specialization membrane (GO:0(	BP	2733
90	MDGA1	HGNC:19267	MDGA1	MAM domain containing glycosylphosphatidylinositol anchor 1	GPIM,MAMDC3	GO:1905606	regulation of presynapse assembly (GO:1905606)	BP	2751
91	ARHGAP39	HGNC:29351	ARHGAP39	Rho GTPase activating protein 39	KIAA1688,VILSE,CRGAP	GO:0098794	postsynapse (GO:0098794)	CC	2816
92	ARHGAP39	HGNC:29351	ARHGAP39	Rho GTPase activating protein 39	KIAA1688,VILSE,CRGAP	GO:0099173	postsynapse organization (GO:0099173)	BP	2817
93	MDGA1	HGNC:19267	MDGA1	MAM domain containing glycosylphosphatidylinositol anchor 1	GPIM,MAMDC3	GO:0099179	regulation of synaptic membrane adhesion (GO:0099179)	BP	2840
94	EPS15	HGNC:3419	EPS15	epidermal growth factor receptor pathway substrate 15	AF-1P,MLLT5	GO:0045202	synapse (GO:0045202)	CC	2882
95	EPS15	HGNC:3419	EPS15	epidermal growth factor receptor pathway substrate 15	AF-1P,MLLT5	GO:0098884	postsynaptic neurotransmitter receptor endocytosis (GO:0098884)	BP	2883
96	PTN	HGNC:9630	PTN	pleiotrophin	HBNF,HBGF8,NEGF1	GO:0050804	modulation of chemical synaptic transmission (GO:0050804)	BP	3072
97	DAPK1	HGNC:2674	DAPK1	death associated protein kinase 1	DAPK	GO:0014069	postsynaptic density (GO:0014069)	CC	3089
98	PLXND1	HGNC:9107	PLXND1	plexin D1	KIAA0620	GO:0008039	synaptic target recognition (GO:0008039)	BP	3116
99	WLS	HGNC:30238	WLS	Wnt ligand secretion mediator	FLJ23091,MRP,EVI,MIG-14,C1ORF139,GPR177	GO:0099537	trans-synaptic signaling (GO:0099537)	BP	3673
100	PFN1	HGNC:8881	PFN1	profilin 1		GO:0050804	modulation of chemical synaptic transmission (GO:0050804)	BP	3696
101	NUMBL	HGNC:8061	NUMBL	NUMB like endocytic adaptor protein	NUMB-R,CTG3A,CAG3A,TNRC23,NUMBR,NUMBLIKE	GO:0150052	regulation of postsynapse assembly (GO:0150052)	BP	3707
102	ARPC2	HGNC:705	ARPC2	actin related protein 2/3 complex subunit 2	P34-ARC,ARC34	GO:0098794	postsynapse (GO:0098794)	CC	3987
103	NPY2R	HGNC:7957	NPY2R	neuropeptide Y receptor Y2		GO:0098793	presynapse (GO:0098793)	CC	4036
104	NPY2R	HGNC:7957	NPY2R	neuropeptide Y receptor Y2		GO:0099538	synaptic signaling via neuropeptide (GO:0099538)	BP	4056
105	DTNBP1	HGNC:17328	DTNBP1	dystrobrevin binding protein 1	DYSBINDIN,MY031,HPS7,DBND,BLOC1S8	GO:0098693	regulation of synaptic vesicle cycle (GO:0098693)	BP	4096
106	GAP43	HGNC:4140	GAP43	growth associated protein 43	B-50,PP46,GAP-43	GO:0098793	presynapse (GO:0098793)	CC	4166
107									

	A	B	C	D	E	F	G	H	I	J
1	**Supplementary Table 6- Syngo CA2**									
2										
3										
4	**your genelist input**	**gene hgnc_id**	**gene symbol**	**gene name**	**gene synonyms**	**GO term ID**	**GO term name**	**GO doma**	**SynGO annotation ID**	
5	PRKCA	HGNC:9393	PRKCA	protein kinase C alpha	PKCA,PKCA	GO:0099523	presynaptic cytosol (GO:0099523)	CC	68	
6	PRKCB	HGNC:9395	PRKCB	protein kinase C beta	PKCB,PRKCB2,PKCB,PRKCB1	GO:0099523	presynaptic cytosol (GO:0099523)	CC	69	
7	PRKCA	HGNC:9393	PRKCA	protein kinase C alpha	PKCA,PKCA	GO:2000300	regulation of synaptic vesicle exocytosis (GO:2000300)	BP	70	
8	PRKCB	HGNC:9395	PRKCB	protein kinase C beta	PKCB,PRKCB2,PKCB,PRKCB1	GO:2000300	regulation of synaptic vesicle exocytosis (GO:2000300)	BP	71	
9	CABP1	HGNC:1384	CABP1	calcium binding protein 1		GO:0099092	postsynaptic density, intracellular component (GO:0099092)	CC	108	
10	ITPR1	HGNC:6180	ITPR1	inositol 1,4,5-trisphosphate receptor type 1	INSP3R1,IP3R1,ACV,PPP1R94,SCA15,SCA16,SCA29	GO:0098695	inositol 1,4,5-trisphosphate receptor activity involved in regulation of postsynaptic cytosc	BP	159	
11	ITPR1	HGNC:6180	ITPR1	inositol 1,4,5-trisphosphate receptor type 1	INSP3R1,IP3R1,ACV,PPP1R94,SCA15,SCA16,SCA29	SYNGO:postsyn_s	integral component of postsynaptic SER membrane	CC	160	
12	ITPR1	HGNC:6180	ITPR1	inositol 1,4,5-trisphosphate receptor type 1	INSP3R1,IP3R1,ACV,PPP1R94,SCA15,SCA16,SCA29	SYNGO:presyn_se	integral component of presynaptic SER membrane	CC	161	
13	HOMER3	HGNC:17514	HOMER3	homer scaffold protein 3	HOMER-3	GO:0014069	postsynaptic density (GO:0014069)	CC	221	
14	CADM1	HGNC:5951	CADM1	cell adhesion molecule 1	NECL2,ST17,BL2,SYNCAM,IGSF4A,NECL-2,SYNCAM1,RA175,TSLC1,IGSF4	GO:0098880	maintenance of postsynaptic specialization structure (GO:0098880)	BP	230	
15	CADM1	HGNC:5951	CADM1	cell adhesion molecule 1	NECL2,ST17,BL2,SYNCAM,IGSF4A,NECL-2,SYNCAM1,RA175,TSLC1,IGSF4	GO:0099055	integral component of postsynaptic membrane (GO:0099055)	CC	231	
16	CADM3	HGNC:17601	CADM3	cell adhesion molecule 3	BIGR,FLJ10698,TSLL1,NECL1,SYNCAM3,NECL-1,IGSF4B	GO:0099056	integral component of presynaptic membrane (GO:0099056)	CC	236	
17	CADM1	HGNC:5951	CADM1	cell adhesion molecule 1	NECL2,ST17,BL2,SYNCAM,IGSF4A,NECL-2,SYNCAM1,RA175,TSLC1,IGSF4	GO:0099056	integral component of presynaptic membrane (GO:0099056)	CC	310	
18	ADCY1	HGNC:232	ADCY1	adenylate cyclase 1	DFNB44	GO:0099061	integral component of postsynaptic density membrane (GO:0099061)	CC	321	
19	ADCY1	HGNC:232	ADCY1	adenylate cyclase 1	DFNB44	GO:0099171	presynaptic modulation of chemical synaptic transmission (GO:0099171)	BP	324	
20	ERC2	HGNC:31922	ERC2	ELKS/RAB6-interacting/CAST family member 2	CAST1,KIAA0378,SPBC110,SPC110,ELKSL	GO:0098831	presynaptic active zone cytoplasmic component (GO:0098831)	CC	330	
21	ERC2	HGNC:31922	ERC2	ELKS/RAB6-interacting/CAST family member 2	CAST1,KIAA0378,SPBC110,SPC110,ELKSL	GO:0016082	synaptic vesicle priming (GO:0016082)	BP	332	
22	CACNA2D1	HGNC:1399	CACNA2D1	calcium voltage-gated channel auxiliary subunit alpha2delta 1	LNCRNA-N3,ALPHA2DELTA-1,CACNL2A,CACNA2,MHS3,LINC01112	GO:0099029	anchored component of presynaptic active zone membrane (GO:0099029)	CC	344	
23	MAL2	HGNC:13634	MAL2	mal, T cell differentiation protein 2		GO:0030285	integral component of synaptic vesicle membrane (GO:0030285)	CC	353	
24	CACNA1C	HGNC:1390	CACNA1C	calcium voltage-gated channel subunit alpha1 C	CAV1.2,CACH2,CACN2,LQT8,CCHL1A1,CACNL1A1,CACNA1C-IT2	GO:0099055	integral component of postsynaptic membrane (GO:0099055)	CC	385	
25	EFNB3	HGNC:3228	EFNB3	ephrin B3	LERK-8,EPLG8	GO:0099061	integral component of postsynaptic density membrane (GO:0099061)	CC	392	
26	EFNB3	HGNC:3228	EFNB3	ephrin B3	LERK-8,EPLG8	GO:0099056	integral component of presynaptic membrane (GO:0099056)	CC	393	
27	ERC2	HGNC:31922	ERC2	ELKS/RAB6-interacting/CAST family member 2	CAST1,KIAA0378,SPBC110,SPC110,ELKSL	GO:0098882	structural constituent of active zone (GO:0098882)	BP	403	
28	EPHA7	HGNC:3390	EPHA7	EPH receptor A7	HEK11	GO:0099060	integral component of postsynaptic specialization membrane (GO:0099060)	CC	449	
29	CACNA2D1	HGNC:1399	CACNA2D1	calcium voltage-gated channel auxiliary subunit alpha2delta 1	LNCRNA-N3,ALPHA2DELTA-1,CACNL2A,CACNA2,MHS3,LINC01112	GO:0099025	anchored component of postsynaptic membrane (GO:0099025)	CC	464	
30	ADCY1	HGNC:232	ADCY1	adenylate cyclase 1	DFNB44	GO:0050804	modulation of chemical synaptic transmission (GO:0050804)	BP	471	
31	CADPS2	HGNC:16018	CADPS2	calcium dependent secretion activator 2		GO:0098793	presynapse (GO:0098793)	CC	495	
32	CADPS2	HGNC:16018	CADPS2	calcium dependent secretion activator 2		GO:0016082	synaptic vesicle priming (GO:0016082)	BP	558	
33	CPLX2	HGNC:2310	CPLX2	complexin 2	CPX-2,DKFZP547D155	GO:0031630	regulation of synaptic vesicle fusion to presynaptic active zone membrane (GO:0031630)	BP	576	
34	CPLX2	HGNC:2310	CPLX2	complexin 2	CPX-2,DKFZP547D155	GO:0098793	presynapse (GO:0098793)	CC	580	
35	CPLX2	HGNC:2310	CPLX2	complexin 2	CPX-2,DKFZP547D155	GO:0098793	presynapse (GO:0098793)	CC	582	
36	CPLX2	HGNC:2310	CPLX2	complexin 2	CPX-2,DKFZP547D155	GO:0098794	postsynapse (GO:0098794)	CC	585	
37	CACNA1C	HGNC:1390	CACNA1C	calcium voltage-gated channel subunit alpha1 C	CAV1.2,CACH2,CACN2,LQT8,CCHL1A1,CACNL1A1,CACNA1C-IT2	GO:0099056	integral component of presynaptic membrane (GO:0099056)	CC	809	
38	LGI1	HGNC:6572	LGI1	leucine rich glioma inactivated 1	IB1099,EPITEMPIN,EPT	GO:0043083	synaptic cleft (GO:0043083)	CC	823	
39	MARCKS	HGNC:6759	MARCKS	myristoylated alanine rich protein kinase C substrate	PKCSL,80K-L,MACS	GO:0099571	postsynaptic cytoskeleton (GO:0099571)	CC	825	
40	MARCKS	HGNC:6759	MARCKS	myristoylated alanine rich protein kinase C substrate	PKCSL,80K-L,MACS	GO:1905274	regulation of modification of postsynaptic actin cytoskeleton (GO:1905274)	BP	829	
41	DTNBP1	HGNC:17328	DTNBP1	dystrobrevin binding protein 1	DYSBINDIN,MY031,HPS7,DBND,BLOC1S8	GO:0030672	synaptic vesicle membrane (GO:0030672)	CC	866	
42	DTNBP1	HGNC:17328	DTNBP1	dystrobrevin binding protein 1	DYSBINDIN,MY031,HPS7,DBND,BLOC1S8	GO:0014069	postsynaptic density (GO:0014069)	CC	867	
43	PFN1	HGNC:8881	PFN1	profilin 1		GO:0098793	presynapse (GO:0098793)	CC	890	
44	PFN1	HGNC:8881	PFN1	profilin 1		GO:0098794	postsynapse (GO:0098794)	CC	891	
45	PPP1R9B	HGNC:9298	PPP1R9B	protein phosphatase 1 regulatory subunit 9B	SPINO,PPP1R9	GO:0014069	postsynaptic density (GO:0014069)	CC	914	
46	PPFIA2	HGNC:9246	PPFIA2	PTPRF interacting protein alpha 2		GO:0045202	synapse (GO:0045202)	CC	931	
47	PPFIA2	HGNC:9246	PPFIA2	PTPRF interacting protein alpha 2		GO:2000300	regulation of synaptic vesicle exocytosis (GO:2000300)	BP	935	
48	PPFIA2	HGNC:9246	PPFIA2	PTPRF interacting protein alpha 2		GO:0099181	structural constituent of presynapse (GO:0099181)	BP	936	
49	CNTN1	HGNC:2171	CNTN1	contactin 1		GO:0099026	anchored component of presynaptic membrane (GO:0099026)	CC	945	
50	CNTN1	HGNC:2171	CNTN1	contactin 1		GO:0099025	anchored component of postsynaptic membrane (GO:0099025)	CC	946	
51	CAMK1	HGNC:1459	CAMK1	calcium/calmodulin dependent protein kinase I	CAMKI,CAMKI-ALPHA	GO:0014069	postsynaptic density (GO:0014069)	CC	979	
52	ADORA1	HGNC:262	ADORA1	adenosine A1 receptor	RDC7	GO:0099056	integral component of presynaptic membrane (GO:0099056)	CC	1006	
53	ADORA1	HGNC:262	ADORA1	adenosine A1 receptor	RDC7	GO:0099055	integral component of postsynaptic membrane (GO:0099055)	CC	1007	
54	CADM1	HGNC:5951	CADM1	cell adhesion molecule 1	NECL2,ST17,BL2,SYNCAM,IGSF4A,NECL-2,SYNCAM1,RA175,TSLC1,IGSF4	GO:0099560	synapse adhesion between pre- and post-synapse (GO:0099560)	BP	1016	
55	CADM1	HGNC:5951	CADM1	cell adhesion molecule 1	NECL2,ST17,BL2,SYNCAM,IGSF4A,NECL-2,SYNCAM1,RA175,TSLC1,IGSF4	GO:0098942	retrograde trans-synaptic signaling by trans-synaptic protein complex (GO:0098942)	BP	1019	
56	SEPT6	HGNC:15848	SEPTIN6	septin 6	KIAA0128,MGC16619,MGC20339,SEPTIN-6,SEPT6	GO:0098793	presynapse (GO:0098793)	CC	1027	
57	PPFIA2	HGNC:9246	PPFIA2	PTPRF interacting protein alpha 2		GO:0099054	presynapse assembly (GO:0099054)	BP	1037	
58	EFNB3	HGNC:3228	EFNB3	ephrin B3	LERK-8,EPLG8	GO:0099557	trans-synaptic signaling by trans-synaptic complex, modulating synaptic transmission (GO	BP	1046	
59	CDH9	HGNC:1768	CDH9	cadherin 9		GO:0099560	synapse adhesion between pre- and post-synapse (GO:0099560)	BP	1068	
60	PCDH17	HGNC:14267	PCDH17	protocadherin 17	PCDH68,PCH68	GO:0099056	integral component of presynaptic membrane (GO:0099056)	CC	1084	
61	MAL2	HGNC:13634	MAL2	mal, T cell differentiation protein 2		GO:0030285	integral component of synaptic vesicle membrane (GO:0030285)	CC	1087	
62	GRIK4	HGNC:4582	GRIK4	glutamate ionotropic receptor kainate type subunit 4	GLUK4,KA1,GRIK	GO:0099056	integral component of presynaptic membrane (GO:0099056)	CC	1115	
63	GRIK4	HGNC:4582	GRIK4	glutamate ionotropic receptor kainate type subunit 4	GLUK4,KA1,GRIK	GO:0099056	integral component of presynaptic membrane (GO:0099056)	CC	1120	
64	NSMF	HGNC:29843	NSMF	NMDA receptor synaptonuclear signaling and neuronal migra	NELF	GO:0098794	postsynapse (GO:0098794)	CC	1293	
65	NSMF	HGNC:29843	NSMF	NMDA receptor synaptonuclear signaling and neuronal migra	NELF	GO:0099527	postsynapse to nucleus signaling pathway (GO:0099527)	BP	1294	
66	GABRA1	HGNC:4075	GABRA1	gamma-aminobutyric acid type A receptor subunit alpha1	EJM5	GO:0099060	integral component of postsynaptic specialization membrane (GO:0099060)	CC	1301	
67	HOMER3	HGNC:17514	HOMER3	homer scaffold protein 3	HOMER-3	GO:0098962	regulation of postsynaptic neurotransmitter receptor activity (GO:0098962)	BP	1304	
68	CDH9	HGNC:1768	CDH9	cadherin 9		GO:0007416	synapse assembly (GO:0007416)	BP	1311	
69	PCDH17	HGNC:14267	PCDH17	protocadherin 17	PCDH68,PCH68	GO:0099055	integral component of postsynaptic membrane (GO:0099055)	CC	1323	
70	RPH3A	HGNC:17056	RPH3A	rabphilin 3A	KIAA0985,RABPHILIN,EXOPHILIN-1	GO:0016082	synaptic vesicle priming (GO:0016082)	BP	1403	
71	RPH3A	HGNC:17056	RPH3A	rabphilin 3A	KIAA0985,RABPHILIN,EXOPHILIN-1	GO:0098850	extrinsic component of synaptic vesicle membrane (GO:0098850)	CC	1405	
72	PLPPR4	HGNC:23496	PLPPR4	phospholipid phosphatase related 4	PRG-1,LPPR4,KIAA0455,PHP1	GO:0099061	integral component of postsynaptic density membrane (GO:0099061)	CC	1412	
73	GRIK4	HGNC:4582	GRIK4	glutamate ionotropic receptor kainate type subunit 4	GLUK4,KA1,GRIK	GO:0099055	integral component of postsynaptic membrane (GO:0099055)	CC	1456	
74	CABP1	HGNC:1384	CABP1	calcium binding protein 1		GO:0099527	postsynapse to nucleus signaling pathway (GO:0099527)	BP	1491	
75	CSPG5	HGNC:2467	CSPG5	chondroitin sulfate proteoglycan 5	NGC	GO:0099055	integral component of postsynaptic membrane (GO:0099055)	CC	1657	
76	PFN1	HGNC:8881	PFN1	profilin 1		GO:0060074	synapse maturation (GO:0060074)	BP	1663	
77	TUBB2B	HGNC:30829	TUBB2B	tubulin beta 2B class IIb	MGC8685,DKFZP566F223,BA506K6.1	GO:0050804	modulation of chemical synaptic transmission (GO:0050804)	BP	1686	
78	EPHA7	HGNC:3390	EPHA7	EPH receptor A7	HEK11	GO:0099061	integral component of postsynaptic density membrane (GO:0099061)	CC	1718	
79	EPHA7	HGNC:3390	EPHA7	EPH receptor A7	HEK11	GO:0099175	regulation of postsynapse organization (GO:0099175)	BP	1719	
80	GAP43	HGNC:4140	GAP43	growth associated protein 43	B-50,PP46,GAP-43	GO:0099150	regulation of postsynaptic specialization assembly (GO:0099150)	BP	1720	
81	PCDH17	HGNC:14267	PCDH17	protocadherin 17	PCDH68,PCH68	GO:0099560	synapse adhesion between pre- and post-synapse (GO:0099560)	BP	1740	
82	LGI1	HGNC:6572	LGI1	leucine rich glioma inactivated 1	IB1099,EPITEMPIN,EPT	GO:0099645	neurotransmitter receptor localization to postsynaptic specialization membrane (GO:009	BP	1748	
83	CADM1	HGNC:5951	CADM1	cell adhesion molecule 1	NECL2,ST17,BL2,SYNCAM,IGSF4A,NECL-2,SYNCAM1,RA175,TSLC1,IGSF4	GO:0007416	synapse assembly (GO:0007416)	BP	1753	
84	PFN1	HGNC:8881	PFN1	profilin 1		GO:0098885	modification of postsynaptic actin cytoskeleton (GO:0098885)	BP	1790	
85	PLPPR4	HGNC:23496	PLPPR4	phospholipid phosphatase related 4	PRG-1,LPPR4,KIAA0455,PHP1	GO:0099061	integral component of postsynaptic density membrane (GO:0099061)	CC	1904	
86	PLPPR4	HGNC:23496	PLPPR4	phospholipid phosphatase related 4	PRG-1,LPPR4,KIAA0455,PHP1	GO:0050804	modulation of chemical synaptic transmission (GO:0050804)	BP	1909	
87	PLPPR4	HGNC:23496	PLPPR4	phospholipid phosphatase related 4	PRG-1,LPPR4,KIAA0455,PHP1	GO:0099175	regulation of postsynapse organization (GO:0099175)	BP	1940	
88	CSPG5	HGNC:2467	CSPG5	chondroitin sulfate proteoglycan 5	NGC	GO:2000300	regulation of synaptic vesicle exocytosis (GO:2000300)	BP	1967	
89	CACNG3	HGNC:1407	CACNG3	calcium voltage-gated channel auxiliary subunit gamma 3		GO:0099061	integral component of postsynaptic density membrane (GO:0099061)	CC	2046	
90	GABRA1	HGNC:4075	GABRA1	gamma-aminobutyric acid type A receptor subunit alpha1	EJM5	GO:1904315	transmitter-gated ion channel activity involved in regulation of postsynaptic membrane p	BP	2054	
91	GABRB3	HGNC:4083	GABRB3	gamma-aminobutyric acid type A receptor subunit beta3		GO:1904315	transmitter-gated ion channel activity involved in regulation of postsynaptic membrane p	BP	2060	
92	GABRA1	HGNC:4075	GABRA1	gamma-aminobutyric acid type A receptor subunit alpha1	EJM5	GO:0099060	integral component of postsynaptic specialization membrane (GO:0099060)	CC	2061	
93	FLRT3	HGNC:3762	FLRT3	fibronectin leucine rich transmembrane protein 3		GO:0099560	synapse adhesion between pre- and post-synapse (GO:0099560)	BP	2103	
94	FLRT3	HGNC:3762	FLRT3	fibronectin leucine rich transmembrane protein 3		GO:0099055	integral component of postsynaptic membrane (GO:0099055)	CC	2120	
95	SCG2	HGNC:10575	SCG2	secretogranin II	CHGC	GO:0098992	neuronal dense core vesicle (GO:0098992)	CC	2132	
96	GABRB3	HGNC:4083	GABRB3	gamma-aminobutyric acid type A receptor subunit beta3		GO:0099055	integral component of postsynaptic membrane (GO:0099055)	CC	2170	
97	GABRB3	HGNC:4083	GABRB3	gamma-aminobutyric acid type A receptor subunit beta3		GO:0099060	integral component of postsynaptic specialization membrane (GO:0099060)	CC	2171	
98	GABRA1	HGNC:4075	GABRA1	gamma-aminobutyric acid type A receptor subunit alpha1	EJM5	GO:0099060	integral component of postsynaptic specialization membrane (GO:0099060)	CC	2173	
99	NCAM1	HGNC:7656	NCAM1	neural cell adhesion molecule 1	NCAM,CD56	GO:0099056	integral component of presynaptic membrane (GO:0099056)	CC	2224	
100	NECTIN1	HGNC:9706	NECTIN1	nectin cell adhesion molecule 1	PRR1,PVRR1,SK-12,HIGR,CLPED1,CD111,OFC7,NECTIN-1,HVEC,ED4,PVRL1	GO:0099059	integral component of presynaptic active zone membrane (GO:0099059)	CC	2232	
101	PTPRD	HGNC:9668	PTPRD	protein tyrosine phosphatase receptor type D	PTPD,HPTP	GO:0099056	integral component of presynaptic membrane (GO:0099056)	CC	2248	
102	PTPRO	HGNC:9678	PTPRO	protein tyrosine phosphatase receptor type O	PTPU2,GLEPP1,PTP-U2,PTP-OC,NPHS6	GO:0099061	integral component of postsynaptic density membrane (GO:0099061)	CC	2311	
103	CAPZB	HGNC:1491	CAPZB	capping actin protein of muscle Z-line subunit beta		GO:0014069	postsynaptic density (GO:0014069)	CC	2315	
104	SHISA6	HGNC:34491	SHISA6	shisa family member 6	FLJ45455	GO:0099061	integral component of postsynaptic density membrane (GO:0099061)	CC	2342	
105	SHISA6	HGNC:34491	SHISA6	shisa family member 6	FLJ45455	GO:0098970	postsynaptic neurotransmitter receptor diffusion trapping (GO:0098970)	BP	2343	
106	SHISA6	HGNC:34491	SHISA6	shisa family member 6	FLJ45455	GO:0098962	regulation of postsynaptic neurotransmitter receptor activity (GO:0098962)	BP	2350	
107	DTNBP1	HGNC:17328	DTNBP1	dystrobrevin binding protein 1	DYSBINDIN,MY031,HPS7,DBND,BLOC1S8	GO:2000300	regulation of synaptic vesicle exocytosis (GO:2000300)	BP	2383	
108	NCAM1	HGNC:7656	NCAM1	neural cell adhesion molecule 1	NCAM,CD56	GO:0099055	integral component of postsynaptic membrane (GO:0099055)	CC	2406	
109	PTPRO	HGNC:9678	PTPRO	protein tyrosine phosphatase receptor type O	PTPU2,GLEPP1,PTP-U2,PTP-OC,NPHS6	GO:0050807	regulation of synapse organization (GO:0050807)	BP	2408	
110	PTPRD	HGNC:9668	PTPRD	protein tyrosine phosphatase receptor type D	PTPD,HPTP	GO:0099560	synapse adhesion between pre- and post-synapse (GO:0099560)	BP	2465	
111	PTPRD	HGNC:9668	PTPRD	protein tyrosine phosphatase receptor type D	PTPD,HPTP	GO:0099560	synapse adhesion between pre- and post-synapse (GO:0099560)	BP	2473	
112	PTPRD	HGNC:9668	PTPRD	protein tyrosine phosphatase receptor type D	PTPD,HPTP	GO:0099560	synapse adhesion between pre- and post-synapse (GO:0099560)	BP	2478	
113	GRIA3	HGNC:4573	GRIA3	glutamate ionotropic receptor AMPA type subunit 3	GLUA3,GLURC,MRX94,GLUR-3,GLUR-C,GLUR-K3,IGLUR3,GLUR3	GO:1904315	transmitter-gated ion channel activity involved in regulation of postsynaptic membrane p	BP	2483	
114	GRIA3	HGNC:4573	GRIA3	glutamate ionotropic receptor AMPA type subunit 3	GLUA3,GLURC,MRX94,GLUR-3,GLUR-C,GLUR-K3,IGLUR3,GLUR3	GO:0099061	integral component of postsynaptic density membrane (GO:0099061)	CC	2484	
115	PPP1CC	HGNC:9283	PPP1CC	protein phosphatase 1 catalytic subunit gamma	PP1GAMMA	GO:0098794	postsynapse (GO:0098794)	CC	2515	
116	PPP1CC	HGNC:9283	PPP1CC	protein phosphatase 1 catalytic subunit gamma	PP1GAMMA	GO:0098793	presynapse (GO:0098793)	CC	2516	
117	NR3C2	HGNC:7979	NR3C2	nuclear receptor subfamily 3 group C member 2	MR,MLR	GO:0099092	postsynaptic density, intracellular component (GO:0099092)	CC	2545	
118	NR3C1	HGNC:7978	NR3C1	nuclear receptor subfamily 3 group C member 1	GR,GRL	GO:0099092	postsynaptic density, intracellular component (GO:0099092)	CC	2546	
119	NR3C2	HGNC:7979	NR3C2	nuclear receptor subfamily 3 group C member 2	MR,MLR	GO:0098831	presynaptic active zone cytoplasmic component (GO:0098831)	CC	2547	
120	NCAM1	HGNC:7656	NCAM1	neural cell adhesion molecule 1	NCAM,CD56	GO:0050804	modulation of chemical synaptic transmission (GO:0050804)	BP	2642	
121	PTPRD	HGNC:9668	PTPRD	protein tyrosine phosphatase receptor type D	PTPD,HPTP	GO:0050804	modulation of chemical synaptic transmission (GO:0050804)	BP	2648	
122	CACNG3	HGNC:1407	CACNG3	calcium voltage-gated channel auxiliary subunit gamma 3		GO:0099645	neurotransmitter receptor localization to postsynaptic specialization membrane (GO:009	BP	2724	
123	PPFIA2	HGNC:9246	PPFIA2	PTPRF interacting protein alpha 2		GO:0099523	presynaptic cytosol (GO:0099523)	CC	2735	
124	ERC2	HGNC:31922	ERC2	ELKS/RAB6-interacting/CAST family member 2	CAST1,KIAA0378,SPBC110,SPC110,ELKSL	GO:0098831	presynaptic active zone cytoplasmic component (GO:0098831)	CC	2736	
125	CNKSR2	HGNC:19701	CNKSR2	connector enhancer of kinase suppressor of Ras 2	KIAA0902,CNK2	GO:0099173	postsynapse organization (GO:0099173)	BP	2788	
126	GNB5	HGNC:4401	GNB5	G protein subunit beta 5	GB5	GO:0098793	presynapse (GO:0098793)	CC	2833	
127	RGS14	HGNC:9996	RGS14	regulator of G protein signaling 14		GO:0050804	modulation of chemical synaptic transmission (GO:0050804)	BP	2864	
128	RGS14	HGNC:9996	RGS14	regulator of G protein signaling 14		GO:0014069	postsynaptic density (GO:0014069)	CC	2865	
129	CABP1	HGNC:1384	CABP1	calcium binding protein 1		GO:0098885	modification of postsynaptic actin cytoskeleton (GO:0098885)	BP	2871	
130	ITPKA	HGNC:6178	ITPKA	inositol-trisphosphate 3-kinase A	IP3KA,IP3-3KA	GO:0098885	modification of postsynaptic actin cytoskeleton (GO:0098885)	BP	3035	
131	ITPKA	HGNC:6178	ITPKA	inositol-trisphosphate 3-kinase A	IP3KA,IP3-3KA	GO:0098871	postsynaptic actin cytoskeleton (GO:0098871)	CC	3036	
132	DAPK1	HGNC:2674	DAPK1	death associated protein kinase 1	DAPK	GO:0014069	postsynaptic density (GO:0014069)	CC	3089	
133	CNKSR2	HGNC:19701	CNKSR2	connector enhancer of kinase suppressor of Ras 2	KIAA0902,CNK2	GO:0099147	extrinsic component of postsynaptic density membrane (GO:0099147)	CC	3118	
134	EPHA7	HGNC:3390	EPHA7	EPH receptor A7	HEK11	GO:0050804	modulation of chemical synaptic transmission (GO:0050804)	BP	3691	
135	PFN1	HGNC:8881	PFN1	profilin 1		GO:0050804	modulation of chemical synaptic transmission (GO:0050804)	BP	3696	
136	ERC2	HGNC:31922	ERC2	ELKS/RAB6-interacting/CAST family member 2	CAST1,KIAA0378,SPBC110,SPC110,ELKSL	GO:0099509	regulation of presynaptic cytosolic calcium levels (GO:0099509)	BP	3721	
137	ERC2	HGNC:31922	ERC2	ELKS/RAB6-interacting/CAST family member 2	CAST1,KIAA0378,SPBC110,SPC110,ELKSL	GO:0150037	regulation of calcium-dependent activation of synaptic vesicle fusion (GO:0150037)	BP	3722	
138	ERC2	HGNC:31922	ERC2	ELKS/RAB6-interacting/CAST family member 2	CAST1,KIAA0378,SPBC110,SPC110,ELKSL	GO:0016082	synaptic vesicle priming (GO:0016082)	BP	3740	
139	HCN1	HGNC:4845	HCN1	hyperpolarization activated cyclic nucleotide gated potassium	BCNG-1,HAC-2,BCNG1	GO:0099059	integral component of presynaptic active zone membrane (GO:0099059)	CC	3799	
140	HCN1	HGNC:4845	HCN1	hyperpolarization activated cyclic nucleotide gated potassium	BCNG-1,HAC-2,BCNG1	GO:0140232	intracellular cAMP-activated cation channel activity involved in regulation of presynaptic	BP	3800	
141	HCN1	HGNC:4845	HCN1	hyperpolarization activated cyclic nucleotide gated potassium	BCNG-1,HAC-2,BCNG1	GO:0060078	regulation of postsynaptic membrane potential (GO:0060078)	BP	3801	
142	HCN1	HGNC:4845	HCN1	hyperpolarization activated cyclic nucleotide gated potassium	BCNG-1,HAC-2,BCNG1	GO:0099055	integral component of postsynaptic membrane (GO:0099055)	CC	3829	
143	GRIA3	HGNC:4573	GRIA3	glutamate ionotropic receptor AMPA type subunit 3	GLUA3,GLURC,MRX94,GLUR-3,GLUR-C,GLUR-K3,IGLUR3,GLUR3	GO:0099059	integral component of presynaptic active zone membrane (GO:0099059)	CC	3834	
144	ATP2B1	HGNC:814	ATP2B1	ATPase plasma membrane Ca2+ transporting 1	PMCA1	GO:0042734	presynaptic membrane (GO:0042734)	CC	3982	
145	ARPC2	HGNC:705	ARPC2	actin related protein 2/3 complex subunit 2	P34-ARC,ARC34	GO:0098794	postsynapse (GO:0098794)	CC	3987	
146	NECAP1	HGNC:24539	NECAP1	NECAP endocytosis associated 1	DKFZP566B183	GO:0140238	presynaptic endocytosis (GO:0140238)	BP	3999	
147	NECAP1	HGNC:24539	NECAP1	NECAP endocytosis associated 1	DKFZP566B183	GO:0098793	presynapse (GO:0098793)	CC	4000	
148	SCG2	HGNC:10575	SCG2	secretogranin II	CHGC	GO:0098992	neuronal dense core vesicle (GO:0098992)	CC	4017	
149	CACNA2D1	HGNC:1399	CACNA2D1	calcium voltage-gated channel auxiliary subunit alpha2delta 1	LNCRNA-N3,ALPHA2DELTA-1,CACNL2A,CACNA2,MHS3,LINC01112	GO:0098992	neuronal dense core vesicle (GO:0098992)	CC	4042	
150	CADPS2	HGNC:16018	CADPS2	calcium dependent secretion activator 2		GO:0099525	presynaptic dense core vesicle exocytosis (GO:0099525)	BP	4065	
151	SNPH	HGNC:15931	SNPH	syntaphilin	BA314N13.5	GO:0042734	presynaptic membrane (GO:0042734)	CC	4102	
152	GAP43	HGNC:4140	GAP43	growth associated protein 43	B-50,PP46,GAP-43	GO:0098793	presynapse (GO:0098793)	CC	4166	
153	PPFIA2	HGNC:9246	PPFIA2	PTPRF interacting protein alpha 2		GO:0099523	presynaptic cytosol (GO:0099523)	CC	4172	
154	PPFIA2	HGNC:9246	PPFIA2	PTPRF interacting protein alpha 2		GO:0099572	postsynaptic specialization (GO:0099572)	CC	4175	
155	PPFIA2	HGNC:9246	PPFIA2	PTPRF interacting protein alpha 2		GO:0042734	presynaptic membrane (GO:0042734)	CC	4184	
156	PPFIA2	HGNC:9246	PPFIA2	PTPRF interacting protein alpha 2		GO:0008021	synaptic vesicle (GO:0008021)	CC	4185	

	A	B	C	D	E	F	G	H	I	J
157	CNTNAP2	HGNC:13830	CNTNAP2	contactin associated protein 2	CASPR2,KIAA0868	GO:0099056	integral component of presynaptic membrane (GO:0099056)	CC	4189	
158	CNTNAP2	HGNC:13830	CNTNAP2	contactin associated protein 2	CASPR2,KIAA0868	GO:0099072	regulation of postsynaptic membrane neurotransmitter receptor levels (GO:0099072)	BP	4191	
159	PTPRD	HGNC:9668	PTPRD	protein tyrosine phosphatase receptor type D	PTPD,HPTP	GO:0099537	trans-synaptic signaling (GO:0099537)	BP	4208	
160	KCNK1	HGNC:6272	KCNK1	potassium two pore domain channel subfamily K member 1	K2P1.1,TWIK-1	GO:1905030	voltage-gated ion channel activity involved in regulation of postsynaptic membrane poter	BP	4559	
161	KCTD12	HGNC:14678	KCTD12	potassium channel tetramerization domain containing 12	KIAA1778,PFET1,C13ORF2	GO:0045211	postsynaptic membrane (GO:0045211)	CC	4579	
162	KCTD12	HGNC:14678	KCTD12	potassium channel tetramerization domain containing 12	KIAA1778,PFET1,C13ORF2	GO:0048787	presynaptic active zone membrane (GO:0048787)	CC	4581	
163	KCTD12	HGNC:14678	KCTD12	potassium channel tetramerization domain containing 12	KIAA1778,PFET1,C13ORF2	GO:0048787	presynaptic active zone membrane (GO:0048787)	CC	4584	
164	KCTD12	HGNC:14678	KCTD12	potassium channel tetramerization domain containing 12	KIAA1778,PFET1,C13ORF2	GO:0042734	presynaptic membrane (GO:0042734)	CC	4589	
165	KCTD12	HGNC:14678	KCTD12	potassium channel tetramerization domain containing 12	KIAA1778,PFET1,C13ORF2	GO:0045211	postsynaptic membrane (GO:0045211)	CC	4590	
166	NEGR1	HGNC:17302	NEGR1	neuronal growth regulator 1	KILON,MGC46680,NTRA,IGLON4	GO:0014069	postsynaptic density (GO:0014069)	CC	4613	
167	NECAB2	HGNC:23746	NECAB2	N-terminal EF-hand calcium binding protein 2	EFCBP2	GO:0098794	postsynapse (GO:0098794)	CC	4618	
168	NECAB2	HGNC:23746	NECAB2	N-terminal EF-hand calcium binding protein 2	EFCBP2	GO:0098793	presynapse (GO:0098793)	CC	4619	
169	SLIT2	HGNC:11086	SLIT2	slit guidance ligand 2	SLIT-2,SLIL3	GO:0060074	synapse maturation (GO:0060074)	BP	4634	
170	STX7	HGNC:11442	STX7	syntaxin 7		GO:0030285	integral component of synaptic vesicle membrane (GO:0030285)	CC	4668	
171	STX7	HGNC:11442	STX7	syntaxin 7		GO:0016189	synaptic vesicle to endosome fusion (GO:0016189)	BP	4669	
172	KCNQ3	HGNC:6297	KCNQ3	potassium voltage-gated channel subfamily Q member 3	KV7.3,EBN2	GO:0099055	integral component of postsynaptic membrane (GO:0099055)	CC	4916	
173	MDGA1	HGNC:19267	MDGA1	MAM domain containing glycosylphosphatidylinositol anchor	GPIM,MAMDC3	GO:0099031	anchored component of postsynaptic density membrane (GO:0099031)	CC	4926	
174	MDGA1	HGNC:19267	MDGA1	MAM domain containing glycosylphosphatidylinositol anchor	GPIM,MAMDC3	GO:0090128	regulation of synapse maturation (GO:0090128)	BP	4928	
175	KCTD12	HGNC:14678	KCTD12	potassium channel tetramerization domain containing 12	KIAA1778,PFET1,C13ORF2	GO:0042734	presynaptic membrane (GO:0042734)	CC	4948	
176	KCTD12	HGNC:14678	KCTD12	potassium channel tetramerization domain containing 12	KIAA1778,PFET1,C13ORF2	GO:0042734	presynaptic membrane (GO:0042734)	CC	4950	
177	GNB5	HGNC:4401	GNB5	G protein subunit beta 5	GB5	GO:0045211	postsynaptic membrane (GO:0045211)	CC	4956	
178	GNB5	HGNC:4401	GNB5	G protein subunit beta 5	GB5	GO:0042734	presynaptic membrane (GO:0042734)	CC	5013	
179	GNB5	HGNC:4401	GNB5	G protein subunit beta 5	GB5	GO:0045211	postsynaptic membrane (GO:0045211)	CC	5014	
180	GNB5	HGNC:4401	GNB5	G protein subunit beta 5	GB5	GO:0045211	postsynaptic membrane (GO:0045211)	CC	5015	
181	GNB5	HGNC:4401	GNB5	G protein subunit beta 5	GB5	GO:0042734	presynaptic membrane (GO:0042734)	CC	5016	
182	CNKSR2	HGNC:19701	CNKSR2	connector enhancer of kinase suppressor of Ras 2	KIAA0902,CNK2	GO:0099147	extrinsic component of postsynaptic density membrane (GO:0099147)	CC	5093	
183	CNKSR2	HGNC:19701	CNKSR2	connector enhancer of kinase suppressor of Ras 2	KIAA0902,CNK2	GO:0099084	postsynaptic specialization organization (GO:0099084)	BP	5094	
184	ZDHHC2	HGNC:18469	ZDHHC2	zinc finger DHHC-type palmitoyltransferase 2	ZNF372,DHHC2	GO:0014069	postsynaptic density (GO:0014069)	CC	5105	
185	ZDHHC2	HGNC:18469	ZDHHC2	zinc finger DHHC-type palmitoyltransferase 2	ZNF372,DHHC2	GO:0150054	regulation of postsynaptic neurotransmitter receptor diffusion trapping (GO:0150054)	BP	5106	
186	RGS14	HGNC:9996	RGS14	regulator of G protein signaling 14		GO:0098794	postsynapse (GO:0098794)	CC	5158	
187	PPP1R9B	HGNC:9298	PPP1R9B	protein phosphatase 1 regulatory subunit 9B	SPINO,PPP1R9	GO:0150052	regulation of postsynapse assembly (GO:0150052)	BP	5187	
188	PPP1R9B	HGNC:9298	PPP1R9B	protein phosphatase 1 regulatory subunit 9B	SPINO,PPP1R9	GO:0014069	postsynaptic density (GO:0014069)	CC	5188	
189	PPP1R9B	HGNC:9298	PPP1R9B	protein phosphatase 1 regulatory subunit 9B	SPINO,PPP1R9	GO:0097444	spine apparatus (GO:0097444)	CC	5189	
190	PPP1R9B	HGNC:9298	PPP1R9B	protein phosphatase 1 regulatory subunit 9B	SPINO,PPP1R9	GO:0098890	extrinsic component of postsynaptic membrane (GO:0098890)	CC	5190	
191	SYT17	HGNC:24119	SYT17	synaptotagmin 17	SYT-17	GO:0099149	regulation of postsynaptic neurotransmitter receptor endocytosis (GO:0099149)	BP	5244	
192	SRGAP3	HGNC:19744	SRGAP3	SLIT-ROBO Rho GTPase activating protein 3	KIAA0411,MEGAP,WRP,ARHGAP14	GO:0150052	regulation of postsynapse assembly (GO:0150052)	BP	5291	
193	CADM3	HGNC:17601	CADM3	cell adhesion molecule 3	BIGR,FLJ10698,TSLL1,NECL1,SYNCAM3,NECL-1,IGSF4B	GO:0099560	synapse adhesion between pre- and post-synapse (GO:0099560)	BP	5301	
194	PCDH7	HGNC:8659	PCDH7	protocadherin 7	BH-PCDH,PPP1R120	GO:0014069	postsynaptic density (GO:0014069)	CC	5349	
195	PCDH7	HGNC:8659	PCDH7	protocadherin 7	BH-PCDH,PPP1R120	GO:0150052	regulation of postsynapse assembly (GO:0150052)	BP	5350	
196	ARPC2	HGNC:705	ARPC2	actin related protein 2/3 complex subunit 2	P34-ARC,ARC34	GO:0030672	synaptic vesicle membrane (GO:0030672)	CC	5365	
197	ARPC2	HGNC:705	ARPC2	actin related protein 2/3 complex subunit 2	P34-ARC,ARC34	GO:0098793	presynapse (GO:0098793)	CC	5366	
198	ARPC2	HGNC:705	ARPC2	actin related protein 2/3 complex subunit 2	P34-ARC,ARC34	GO:0098794	postsynapse (GO:0098794)	CC	5367	
199	CPNE7	HGNC:2320	CPNE7	copine 7		GO:0045202	synapse (GO:0045202)	CC	5382	
200	WIPF3	HGNC:22004	WIPF3	WAS/WASL interacting protein family member 3	CR16,FLJ36931	GO:0045202	synapse (GO:0045202)	CC	5407	
201	MRTFB	HGNC:29819	MRTFB	myocardin related transcription factor B	MRTF-B,FLJ31823,MKL2	GO:0098793	presynapse (GO:0098793)	CC	5424	
202	MRTFB	HGNC:29819	MRTFB	myocardin related transcription factor B	MRTF-B,FLJ31823,MKL2	GO:0098794	postsynapse (GO:0098794)	CC	5425	
203	MRTFB	HGNC:29819	MRTFB	myocardin related transcription factor B	MRTF-B,FLJ31823,MKL2	GO:0099159	regulation of modification of postsynaptic structure (GO:0099159)	BP	5427	
204	PLK2	HGNC:19699	PLK2	polo like kinase 2	SNK	GO:0098794	postsynapse (GO:0098794)	CC	5464	
205	PLK2	HGNC:19699	PLK2	polo like kinase 2	SNK	GO:0140252	regulation protein catabolic process at postsynapse (GO:0140252)	BP	5465	
206	ASIC2	HGNC:99	ASIC2	acid sensing ion channel subunit 2	ASIC2A,BNAC1,HBNAC1,MDEG,ACCN,ACCN1	GO:0099061	integral component of postsynaptic density membrane (GO:0099061)	CC	5476	
207	ASIC2	HGNC:99	ASIC2	acid sensing ion channel subunit 2	ASIC2A,BNAC1,HBNAC1,MDEG,ACCN,ACCN1	GO:0150052	regulation of postsynapse assembly (GO:0150052)	BP	5477	
208	SPOCK1	HGNC:11251	SPOCK1	SPARC (osteonectin), cwcv and kazal like domains proteoglyca	TESTICAN-1,TIC1,SPOCK	GO:0014069	postsynaptic density (GO:0014069)	CC	5516	
209	CNTN1	HGNC:2171	CNTN1	contactin 1		GO:0045202	synapse (GO:0045202)	CC	5557	
210	CPNE4	HGNC:2317	CPNE4	copine 4	COPN4,CPN4	GO:0045202	synapse (GO:0045202)	CC	5558	
211	PLXNA1	HGNC:9099	PLXNA1	plexin A1	PLXN1	GO:0045202	synapse (GO:0045202)	CC	5559	
212	KCTD12	HGNC:14678	KCTD12	potassium channel tetramerization domain containing 12	KIAA1778,PFET1,C13ORF2	GO:0150047	G protein-coupled neurotransmitter receptor activity involved in regulation of presynapti	BP	5572	
213	KCTD12	HGNC:14678	KCTD12	potassium channel tetramerization domain containing 12	KIAA1778,PFET1,C13ORF2	GO:0099579	G protein-coupled neurotransmitter receptor activity involved in regulation of postsynap	BP	5576	
214										

	A	B	C	D	E	F	G	H	I
1	**Supplementary Table 6- Syngo CA3**								
2									
3									
4	**your genelist input**	**gene hgnc_id**	**gene symbol**	**gene name**	**gene synonyms**	**GO term ID**	**GO term name**	**GO domain**	**SynGO annotation ID**
5	RAB3C	HGNC:30269	RAB3C	RAB3C, member RAS oncogene family		GO:0098993	anchored component of synaptic vesicle membrane (GO:0098993)	CC	59
6	PRKCA	HGNC:9393	PRKCA	protein kinase C alpha	PKCA,PKCA	GO:0099523	presynaptic cytosol (GO:0099523)	CC	68
7	PRKCA	HGNC:9393	PRKCA	protein kinase C alpha	PKCA,PKCA	GO:2000300	regulation of synaptic vesicle exocytosis (GO:2000300)	BP	70
8	CABP1	HGNC:1384	CABP1	calcium binding protein 1		GO:0099092	postsynaptic density, intracellular component (GO:0099092)	CC	108
9	PRKCD	HGNC:9399	PRKCD	protein kinase C delta		GO:0099524	postsynaptic cytosol (GO:0099524)	CC	146
10	KCNA2	HGNC:6220	KCNA2	potassium voltage-gated channel subfamily A member 2	KV1.2,HK4	GO:0099056	integral component of presynaptic membrane (GO:0099056)	CC	182
11	KCNA2	HGNC:6220	KCNA2	potassium voltage-gated channel subfamily A member 2	KV1.2,HK4	GO:0099055	integral component of postsynaptic membrane (GO:0099055)	CC	183
12	KCNA2	HGNC:6220	KCNA2	potassium voltage-gated channel subfamily A member 2	KV1.2,HK4	GO:0099508	voltage-gated ion channel activity involved in regulation of presynaptic membrane potential (GO:009950	BP	191
13	KCNA2	HGNC:6220	KCNA2	potassium voltage-gated channel subfamily A member 2	KV1.2,HK4	GO:1905030	voltage-gated ion channel activity involved in regulation of postsynaptic membrane potential (GO:190503	BP	192
14	HOMER1	HGNC:17512	HOMER1	homer scaffold protein 1	VES-1,SYN47,HOMER-1B	GO:0014069	postsynaptic density (GO:0014069)	CC	219
15	HOMER3	HGNC:17514	HOMER3	homer scaffold protein 3	HOMER-3	GO:0014069	postsynaptic density (GO:0014069)	CC	221
16	HOMER1	HGNC:17512	HOMER1	homer scaffold protein 1	VES-1,SYN47,HOMER-1B	GO:0098962	regulation of postsynaptic neurotransmitter receptor activity (GO:0098962)	BP	238
17	HOMER1	HGNC:17512	HOMER1	homer scaffold protein 1	VES-1,SYN47,HOMER-1B	GO:0099524	postsynaptic cytosol (GO:0099524)	CC	239
18	RAB18	HGNC:14244	RAB18	RAB18, member RAS oncogene family		GO:0045202	synapse (GO:0045202)	CC	246
19	MAL2	HGNC:13634	MAL2	mal, T cell differentiation protein 2		GO:0030285	integral component of synaptic vesicle membrane (GO:0030285)	CC	353
20	SLC18A2	HGNC:10935	SLC18A2	solute carrier family 18 member A2	SVMT,SVAT,VMAT2	GO:0030285	integral component of synaptic vesicle membrane (GO:0030285)	CC	474
21	SLC18A2	HGNC:10935	SLC18A2	solute carrier family 18 member A2	SVMT,SVAT,VMAT2	GO:0098794	postsynapse (GO:0098794)	CC	475
22	SLC18A2	HGNC:10935	SLC18A2	solute carrier family 18 member A2	SVMT,SVAT,VMAT2	GO:0098700	synaptic vesicle neurotransmitter loading (GO:0098700)	BP	476
23	ELAVL2	HGNC:3313	ELAVL2	ELAV like RNA binding protein 2	HUB,HEL-N1	GO:0045202	synapse (GO:0045202)	CC	481
24	ELAVL4	HGNC:3315	ELAVL4	ELAV like RNA binding protein 4	PNEM,HUD	GO:0140245	regulation of translation at postsynapse (GO:0140245)	BP	499
25	FXYD6	HGNC:4030	FXYD6	FXYD domain containing ion transport regulator 6		GO:0099056	integral component of presynaptic membrane (GO:0099056)	CC	730
26	FXYD6	HGNC:4030	FXYD6	FXYD domain containing ion transport regulator 6		GO:0099055	integral component of postsynaptic membrane (GO:0099055)	CC	731
27	ARPC2	HGNC:705	ARPC2	actin related protein 2/3 complex subunit 2	P34-ARC,ARC34	GO:0045202	synapse (GO:0045202)	CC	804
28	MARCKS	HGNC:6759	MARCKS	myristoylated alanine rich protein kinase C substrate	PKCSL,80K-L,MACS	GO:0099571	postsynaptic cytoskeleton (GO:0099571)	CC	825
29	MARCKS	HGNC:6759	MARCKS	myristoylated alanine rich protein kinase C substrate	PKCSL,80K-L,MACS	GO:0099523	presynaptic cytosol (GO:0099523)	CC	826
30	MARCKS	HGNC:6759	MARCKS	myristoylated alanine rich protein kinase C substrate	PKCSL,80K-L,MACS	GO:0099026	anchored component of presynaptic membrane (GO:0099026)	CC	827
31	MARCKS	HGNC:6759	MARCKS	myristoylated alanine rich protein kinase C substrate	PKCSL,80K-L,MACS	GO:0099025	anchored component of postsynaptic membrane (GO:0099025)	CC	828
32	MARCKS	HGNC:6759	MARCKS	myristoylated alanine rich protein kinase C substrate	PKCSL,80K-L,MACS	GO:1905274	regulation of modification of postsynaptic actin cytoskeleton (GO:1905274)	BP	829
33	PPFIA2	HGNC:9246	PPFIA2	PTPRF interacting protein alpha 2		GO:0045202	synapse (GO:0045202)	CC	931
34	PPFIA2	HGNC:9246	PPFIA2	PTPRF interacting protein alpha 2		GO:2000300	regulation of synaptic vesicle exocytosis (GO:2000300)	BP	935
35	PPFIA2	HGNC:9246	PPFIA2	PTPRF interacting protein alpha 2		GO:0099181	structural constituent of presynapse (GO:0099181)	BP	936
36	MYO5B	HGNC:7603	MYO5B	myosin VB	KIAA1119	GO:0098871	postsynaptic actin cytoskeleton (GO:0098871)	CC	985
37	PPFIA2	HGNC:9246	PPFIA2	PTPRF interacting protein alpha 2		GO:0099054	presynapse assembly (GO:0099054)	BP	1037
38	CDH9	HGNC:1768	CDH9	cadherin 9		GO:0099560	synapse adhesion between pre- and post-synapse (GO:0099560)	BP	1068
39	MAL2	HGNC:13634	MAL2	mal, T cell differentiation protein 2		GO:0030285	integral component of synaptic vesicle membrane (GO:0030285)	CC	1087
40	SLC18A2	HGNC:10935	SLC18A2	solute carrier family 18 member A2	SVMT,SVAT,VMAT2	GO:0098992	neuronal dense core vesicle (GO:0098992)	CC	1149
41	GABRB1	HGNC:4081	GABRB1	gamma-aminobutyric acid type A receptor subunit beta1		GO:0099059	integral component of presynaptic active zone membrane (GO:0099059)	CC	1274
42	GABRB1	HGNC:4081	GABRB1	gamma-aminobutyric acid type A receptor subunit beta1		GO:0099060	integral component of postsynaptic specialization membrane (GO:0099060)	CC	1275
43	NSMF	HGNC:29843	NSMF	NMDA receptor synaptonuclear signaling and neuronal migration factor	NELF	GO:0098794	postsynapse (GO:0098794)	CC	1293
44	NSMF	HGNC:29843	NSMF	NMDA receptor synaptonuclear signaling and neuronal migration factor	NELF	GO:0099527	postsynapse to nucleus signaling pathway (GO:0099527)	BP	1294
45	HOMER3	HGNC:17514	HOMER3	homer scaffold protein 3	HOMER-3	GO:0098962	regulation of postsynaptic neurotransmitter receptor activity (GO:0098962)	BP	1304
46	CDH9	HGNC:1768	CDH9	cadherin 9		GO:0007416	synapse assembly (GO:0007416)	BP	1311
47	CDH9	HGNC:1768	CDH9	cadherin 9		GO:0099056	integral component of presynaptic membrane (GO:0099056)	CC	1312
48	CDH9	HGNC:1768	CDH9	cadherin 9		GO:0099055	integral component of postsynaptic membrane (GO:0099055)	CC	1313
49	GABRB1	HGNC:4081	GABRB1	gamma-aminobutyric acid type A receptor subunit beta1		GO:0099507	ligand-gated ion channel activity involved in regulation of presynaptic membrane potential (GO:0099507)	BP	1337
50	DOC2A	HGNC:2985	DOC2A	double C2 domain alpha		GO:0098850	extrinsic component of synaptic vesicle membrane (GO:0098850)	CC	1396
51	DOC2A	HGNC:2985	DOC2A	double C2 domain alpha		GO:0099502	calcium-dependent activation of synaptic vesicle fusion (GO:0099502)	BP	1398
52	CABP1	HGNC:1384	CABP1	calcium binding protein 1		GO:0099527	postsynapse to nucleus signaling pathway (GO:0099527)	BP	1491
53	SLC18A2	HGNC:10935	SLC18A2	solute carrier family 18 member A2	SVMT,SVAT,VMAT2	GO:0099066	integral component of neuronal dense core vesicle membrane (GO:0099066)	CC	1492
54	SEPT11	HGNC:25589	SEPTIN11	septin 11	FLJ10849,SEPT11	GO:0098794	postsynapse (GO:0098794)	CC	1616
55	SEPT11	HGNC:25589	SEPTIN11	septin 11	FLJ10849,SEPT11	GO:0050807	regulation of synapse organization (GO:0050807)	BP	1631
56	TUBB2B	HGNC:30829	TUBB2B	tubulin beta 2B class IIb	MGC8685,DKFZP566F223,BA506K6.1	GO:0050804	modulation of chemical synaptic transmission (GO:0050804)	BP	1686
57	SEPT11	HGNC:25589	SEPTIN11	septin 11	FLJ10849,SEPT11	GO:0099629	postsynaptic specialization of symmetric synapse (GO:0099629)	CC	1717
58	GAP43	HGNC:4140	GAP43	growth associated protein 43	B-50,PP46,GAP-43	GO:0099150	regulation of postsynaptic specialization assembly (GO:0099150)	BP	1720
59	EPHB1	HGNC:3392	EPHB1	EPH receptor B1	HEK6,EPHT2	GO:0050804	modulation of chemical synaptic transmission (GO:0050804)	BP	1738
60	MYO5B	HGNC:7603	MYO5B	myosin VB	KIAA1119	GO:0098944	postsynaptic recycling endosome membrane (GO:0098944)	CC	1804
61	MYO5B	HGNC:7603	MYO5B	myosin VB	KIAA1119	GO:0099090	synaptic vesicle recycling via endosome (GO:0036466)	BP	1805
62	MYO5B	HGNC:7603	MYO5B	myosin VB	KIAA1119	GO:0099159	regulation of modification of postsynaptic structure (GO:0099159)	BP	1806
63	MYO5B	HGNC:7603	MYO5B	myosin VB	KIAA1119	GO:0050804	modulation of chemical synaptic transmission (GO:0050804)	BP	1807
64	MYO5B	HGNC:7603	MYO5B	myosin VB	KIAA1119	GO:0099639	neurotransmitter receptor transport, endosome to plasma membrane (GO:0099639)	BP	1808
65	GABRB1	HGNC:4081	GABRB1	gamma-aminobutyric acid type A receptor subunit beta1		GO:0150047	G protein-coupled neurotransmitter receptor activity involved in regulation of presynaptic membrane pot	BP	1941
66	DAGLA	HGNC:1165	DAGLA	diacylglycerol lipase alpha	KIAA0659,NSDDR,DAGLALPHA,C11ORF11	GO:0098921	retrograde trans-synaptic signaling by endocannabinoid (GO:0098921)	BP	2114
67	DAGLA	HGNC:1165	DAGLA	diacylglycerol lipase alpha	KIAA0659,NSDDR,DAGLALPHA,C11ORF11	GO:0099055	integral component of postsynaptic membrane (GO:0099055)	CC	2136
68	NETO1	HGNC:13823	NETO1	neuropilin and tolloid like 1	BTCL1,BCTL1	GO:0099061	integral component of postsynaptic density membrane (GO:0099061)	CC	2146
69	GABRB1	HGNC:4081	GABRB1	gamma-aminobutyric acid type A receptor subunit beta1		GO:1904315	transmitter-gated ion channel activity involved in regulation of postsynaptic membrane potential (GO:19	BP	2189
70	TANC1	HGNC:29364	TANC1	tetratricopeptide repeat, ankyrin repeat and coiled-coil containing 1	KIAA1728,ROLSB	GO:0099092	postsynaptic density, intracellular component (GO:0099092)	CC	2249
71	TANC1	HGNC:29364	TANC1	tetratricopeptide repeat, ankyrin repeat and coiled-coil containing 1	KIAA1728,ROLSB	GO:0099175	regulation of postsynapse organization (GO:0099175)	BP	2410
72	NR3C2	HGNC:7979	NR3C2	nuclear receptor subfamily 3 group C member 2	MR,MLR	GO:0099092	postsynaptic density, intracellular component (GO:0099092)	CC	2545
73	NR3C1	HGNC:7978	NR3C1	nuclear receptor subfamily 3 group C member 1	GR,GRL	GO:0099092	postsynaptic density, intracellular component (GO:0099092)	CC	2546
74	NR3C2	HGNC:7979	NR3C2	nuclear receptor subfamily 3 group C member 2	MR,MLR	GO:0098831	presynaptic active zone cytoplasmic component (GO:0098831)	CC	2547
75	NR3C2	HGNC:7979	NR3C2	nuclear receptor subfamily 3 group C member 2	MR,MLR	GO:0099171	presynaptic modulation of chemical synaptic transmission (GO:0099171)	BP	2548
76	NR3C2	HGNC:7979	NR3C2	nuclear receptor subfamily 3 group C member 2	MR,MLR	GO:0060078	regulation of postsynaptic membrane potential (GO:0060078)	BP	2549
77	ASAP1	HGNC:2720	ASAP1	ArfGAP with SH3 domain, ankyrin repeat and PH domain 1	PAP,KIAA1249,ZG14P,CENTB4,DDEF1	GO:0099175	regulation of postsynapse organization (GO:0099175)	BP	2712
78	PPFIA2	HGNC:9246	PPFIA2	PTPRF interacting protein alpha 2		GO:0099523	presynaptic cytosol (GO:0099523)	CC	2735
79	RGS14	HGNC:9996	RGS14	regulator of G protein signaling 14		GO:0050804	modulation of chemical synaptic transmission (GO:0050804)	BP	2864
80	RGS14	HGNC:9996	RGS14	regulator of G protein signaling 14		GO:0014069	postsynaptic density (GO:0014069)	CC	2865
81	CABP1	HGNC:1384	CABP1	calcium binding protein 1		GO:0098885	modification of postsynaptic actin cytoskeleton (GO:0098885)	BP	2871
82	HOMER1	HGNC:17512	HOMER1	homer scaffold protein 1	VES-1,SYN47,HOMER-1B	GO:0099186	structural constituent of postsynapse (GO:0099186)	BP	2921
83	CDH9	HGNC:1768	CDH9	cadherin 9		GO:0045202	synapse (GO:0045202)	CC	2935
84	CDH9	HGNC:1768	CDH9	cadherin 9		GO:0099560	synapse adhesion between pre- and post-synapse (GO:0099560)	BP	2955
85	ARRB2	HGNC:712	ARRB2	arrestin beta 2	BARR2,DKFZP686L0365,ARR2	GO:0098926	postsynaptic signaling pathway (GO:0098926)	BP	3068
86	ARRB2	HGNC:712	ARRB2	arrestin beta 2	BARR2,DKFZP686L0365,ARR2	GO:0050804	modulation of chemical synaptic transmission (GO:0050804)	BP	3069
87	MYO5B	HGNC:7603	MYO5B	myosin VB	KIAA1119	GO:0098794	postsynapse (GO:0098794)	CC	3672
88	DOC2A	HGNC:2985	DOC2A	double C2 domain alpha		GO:0099502	calcium-dependent activation of synaptic vesicle fusion (GO:0099502)	BP	3745
89	NETO1	HGNC:13823	NETO1	neuropilin and tolloid like 1	BTCL1,BCTL1	GO:0140231	anterograde axonal transport of neurotransmitter receptor complex (GO:0140231)	BP	3843
90	ELAVL2	HGNC:3313	ELAVL2	ELAV like RNA binding protein 2	HUB,HEL-N1	GO:0051963	regulation of synapse assembly (GO:0051963)	BP	3848
91	NETO1	HGNC:13823	NETO1	neuropilin and tolloid like 1	BTCL1,BCTL1	GO:0099645	neurotransmitter receptor localization to postsynaptic specialization membrane (GO:0099645)	BP	3858
92	NETO1	HGNC:13823	NETO1	neuropilin and tolloid like 1	BTCL1,BCTL1	GO:0098962	regulation of postsynaptic neurotransmitter receptor activity (GO:0098962)	BP	3860
93	ARPC2	HGNC:705	ARPC2	actin related protein 2/3 complex subunit 2	P34-ARC,ARC34	GO:0098794	postsynapse (GO:0098794)	CC	3987
94	SYT12	HGNC:18381	SYT12	synaptotagmin 12	SRG1	GO:0030285	integral component of synaptic vesicle membrane (GO:0030285)	CC	4001
95	SYT12	HGNC:18381	SYT12	synaptotagmin 12	SRG1	GO:0099171	presynaptic modulation of chemical synaptic transmission (GO:0099171)	BP	4003
96	GAP43	HGNC:4140	GAP43	growth associated protein 43	B-50,PP46,GAP-43	GO:0098793	presynapse (GO:0098793)	CC	4166
97	PPFIA2	HGNC:9246	PPFIA2	PTPRF interacting protein alpha 2		GO:0099523	presynaptic cytosol (GO:0099523)	CC	4172
98	PPFIA2	HGNC:9246	PPFIA2	PTPRF interacting protein alpha 2		GO:0099572	postsynaptic specialization (GO:0099572)	CC	4175
99	PPFIA2	HGNC:9246	PPFIA2	PTPRF interacting protein alpha 2		GO:0042734	presynaptic membrane (GO:0042734)	CC	4184
100	PPFIA2	HGNC:9246	PPFIA2	PTPRF interacting protein alpha 2		GO:0008021	synaptic vesicle (GO:0008021)	CC	4185

	A	B	C	D	E	F	G	H	I	J
1	**Supplementary Table 6- Syngo DG**									
2										
3										
4	**your genelist**	**gene hgnc_id**	**gene symbol**	**gene name**	**gene synonyms**	**GO term ID**	**GO term name**	**GO domain**	**SynGO annotation ID**	
5	PRKCA	HGNC:9393	PRKCA	protein kinas	PKCA,PKCA	GO:0099523	presynaptic cytosol (GO:0099523)	CC	68	
6	PRKCA	HGNC:9393	PRKCA	protein kinas	PKCA,PKCA	GO:2000300	regulation of synaptic vesicle exocytosis (GO:2000300)	BP	70	
7	CABP1	HGNC:1384	CABP1	calcium bind		GO:0099092	postsynaptic density, intracellular component (GO:0099092)	CC	108	
8	RIMBP2	HGNC:30339	RIMBP2	RIMS binding	KIAA0318,RBP2,MGC15831,RIM-BP2,PPP1R133	GO:0098831	presynaptic active zone cytoplasmic component (GO:0098831)	CC	111	
9	RIMBP2	HGNC:30339	RIMBP2	RIMS binding	KIAA0318,RBP2,MGC15831,RIM-BP2,PPP1R133	GO:0098882	structural constituent of active zone (GO:0098882)	BP	112	
10	KCNC4	HGNC:6236	KCNC4	potassium vo	KV3.4,HKSHIIIC,C1ORF30	GO:0099056	integral component of presynaptic membrane (GO:0099056)	CC	173	
11	KCNC4	HGNC:6236	KCNC4	potassium vo	KV3.4,HKSHIIIC,C1ORF30	GO:0099055	integral component of postsynaptic membrane (GO:0099055)	CC	174	
12	KCNC4	HGNC:6236	KCNC4	potassium vo	KV3.4,HKSHIIIC,C1ORF30	GO:0099508	voltage-gated ion channel activity involved in regulation of presynapti	BP	175	
13	KCNC4	HGNC:6236	KCNC4	potassium vo	KV3.4,HKSHIIIC,C1ORF30	GO:1905030	voltage-gated ion channel activity involved in regulation of postsynapt	BP	176	
14	KCNC4	HGNC:6236	KCNC4	potassium vo	KV3.4,HKSHIIIC,C1ORF30	GO:0099056	integral component of presynaptic membrane (GO:0099056)	CC	177	
15	KCNC4	HGNC:6236	KCNC4	potassium vo	KV3.4,HKSHIIIC,C1ORF30	GO:0099055	integral component of postsynaptic membrane (GO:0099055)	CC	186	
16	HOMER3	HGNC:17514	HOMER3	homer scaffo	HOMER-3	GO:0014069	postsynaptic density (GO:0014069)	CC	221	
17	CTTNBP2	HGNC:15679	CTTNBP2	cortactin bin	KIAA1758,ORF4,CORTBP2,C7ORF8	GO:0098871	postsynaptic actin cytoskeleton (GO:0098871)	CC	224	
18	CTTNBP2	HGNC:15679	CTTNBP2	cortactin bin	KIAA1758,ORF4,CORTBP2,C7ORF8	GO:1905274	regulation of modification of postsynaptic actin cytoskeleton (GO:190	BP	225	
19	CADM1	HGNC:5951	CADM1	cell adhesion	NECL2,ST17,BL2,SYNCAM,IGSF4A,NECL-2,SYNCAM1,RA175,TSLC1,IGSF4	GO:0098880	maintenance of postsynaptic specialization structure (GO:0098880)	BP	230	
20	CADM1	HGNC:5951	CADM1	cell adhesion	NECL2,ST17,BL2,SYNCAM,IGSF4A,NECL-2,SYNCAM1,RA175,TSLC1,IGSF4	GO:0099055	integral component of postsynaptic membrane (GO:0099055)	CC	231	
21	NPTX2	HGNC:7953	NPTX2	neuronal per		GO:0098892	extrinsic component of postsynaptic specialization membrane (GO:00	CC	234	
22	CADM3	HGNC:17601	CADM3	cell adhesion	BIGR,FLJ10698,TSLL1,NECL1,SYNCAM3,NECL-1,IGSF4B	GO:0099056	integral component of presynaptic membrane (GO:0099056)	CC	236	
23	KCNJ3	HGNC:6264	KCNJ3	potassium in	KIR3.1,GIRK1,KGA	GO:0099056	integral component of presynaptic membrane (GO:0099056)	CC	284	
24	KCNJ3	HGNC:6264	KCNJ3	potassium in	KIR3.1,GIRK1,KGA	GO:0099508	voltage-gated ion channel activity involved in regulation of presynapti	BP	287	
25	RIMBP2	HGNC:30339	RIMBP2	RIMS binding	KIAA0318,RBP2,MGC15831,RIM-BP2,PPP1R133	GO:0098831	presynaptic active zone cytoplasmic component (GO:0098831)	CC	309	
26	CADM1	HGNC:5951	CADM1	cell adhesion	NECL2,ST17,BL2,SYNCAM,IGSF4A,NECL-2,SYNCAM1,RA175,TSLC1,IGSF4	GO:0099056	integral component of presynaptic membrane (GO:0099056)	CC	310	
27	NPTX2	HGNC:7953	NPTX2	neuronal per		GO:0099645	neurotransmitter receptor localization to postsynaptic specialization n	BP	312	
28	ADCY1	HGNC:232	ADCY1	adenylate cy	AC1,DFNB44	GO:0099061	integral component of postsynaptic density membrane (GO:0099061)	CC	321	
29	ADCY1	HGNC:232	ADCY1	adenylate cy	AC1,DFNB44	GO:0099171	presynaptic modulation of chemical synaptic transmission (GO:00991	BP	324	
30	CTTNBP2	HGNC:15679	CTTNBP2	cortactin bin	KIAA1758,ORF4,CORTBP2,C7ORF8	GO:0050807	regulation of synapse organization (GO:0050807)	BP	360	
31	ADCY1	HGNC:232	ADCY1	adenylate cy	AC1,DFNB44	GO:0050804	modulation of chemical synaptic transmission (GO:0050804)	BP	471	
32	MAGI1	HGNC:946	MAGI1	membrane a	BAP1,MAGI-1,TNRC19,AIP3,WWP3,BAIAP1	GO:0098793	presynapse (GO:0098793)	CC	543	
33	UBA52	HGNC:12458	UBA52	ubiquitin A-5	RPL40,CEP52,HUBCEP52,MGC57125,MGC126879,MGC126881,L40	GO:0045202	synapse (GO:0045202)	CC	608	
34	RNF19A	HGNC:13432	RNF19A	ring finger pr	DORFIN,DKFZP566B1346,RNF19	GO:0099576	regulation of protein catabolic process at postsynapse, modulating syn	BP	652	
35	CPEB1	HGNC:21744	CPEB1	cytoplasmic	FLJ13203,CPEB	GO:0099547	regulation of translation at synapse, modulating synaptic transmission	BP	654	
36	SH3GL2	HGNC:10831	SH3GL2	SH3 domain	SH3P4,SH3D2A,CNSA2,EEN-B1	GO:0048488	synaptic vesicle endocytosis (GO:0048488)	BP	666	
37	AKAP9	HGNC:379	AKAP9	A-kinase anc	KIAA0803,AKAP350,AKAP450,CG-NAP,YOTIAO,HYPERION,PRKA9,MU-RMS-40.16A,PPP1R45,LQT11	GO:0099147	extrinsic component of postsynaptic density membrane (GO:0099147	CC	675	
38	GRASP	HGNC:18707	TAMALIN	trafficking re	GRASP	GO:0014069	postsynaptic density (GO:0014069)	CC	714	
39	TNR	HGNC:11953	TNR	tenascin R		GO:0098966	perisynaptic extracellular matrix (GO:0098966)	CC	778	
40	MARCKSL1	HGNC:7142	MARCKSL1	MARCKS like	F52,MACMARCKS,MLP1,MLP	GO:0099026	anchored component of presynaptic membrane (GO:0099026)	CC	831	
41	MARCKSL1	HGNC:7142	MARCKSL1	MARCKS like	F52,MACMARCKS,MLP1,MLP	GO:0008021	synaptic vesicle (GO:0008021)	CC	832	
42	MARCKSL1	HGNC:7142	MARCKSL1	MARCKS like	F52,MACMARCKS,MLP1,MLP	GO:0099523	presynaptic cytosol (GO:0099523)	CC	833	
43	MARCKSL1	HGNC:7142	MARCKSL1	MARCKS like	F52,MACMARCKS,MLP1,MLP	GO:0099509	regulation of presynaptic cytosolic calcium levels (GO:0099509)	BP	834	
44	NET1	HGNC:11048	SLC6A2	solute carrier	NET,NET1,NAT1,SLC6A5	GO:0098810	neurotransmitter reuptake (GO:0098810)	BP	850	
45	NET1	HGNC:11048	SLC6A2	solute carrier	NET,NET1,NAT1,SLC6A5	GO:0099056	integral component of presynaptic membrane (GO:0099056)	CC	851	
46	NET1	HGNC:11048	SLC6A2	solute carrier	NET,NET1,NAT1,SLC6A5	GO:0030285	integral component of synaptic vesicle membrane (GO:0030285)	CC	852	
47	CALB1	HGNC:1434	CALB1	calbindin 1	CALB	GO:0099524	postsynaptic cytosol (GO:0099524)	CC	883	
48	CALB1	HGNC:1434	CALB1	calbindin 1	CALB	GO:0099523	presynaptic cytosol (GO:0099523)	CC	884	
49	CALB1	HGNC:1434	CALB1	calbindin 1	CALB	GO:0099534	calcium ion binding involved in regulation of presynaptic cytosolic calc	BP	885	
50	PPFIA2	HGNC:9246	PPFIA2	PTPRF intera		GO:0045202	synapse (GO:0045202)	CC	931	
51	PPFIA2	HGNC:9246	PPFIA2	PTPRF intera		GO:2000300	regulation of synaptic vesicle exocytosis (GO:2000300)	BP	935	
52	PPFIA2	HGNC:9246	PPFIA2	PTPRF intera		GO:0099181	structural constituent of presynapse (GO:0099181)	BP	936	
53	PENK	HGNC:8831	PENK	proenkephal		GO:0099013	neuronal dense core vesicle lumen (GO:0099013)	CC	963	
54	PENK	HGNC:8831	PENK	proenkephal		GO:0034592	synaptic vesicle lumen (GO:0034592)	CC	965	
55	PENK	HGNC:8831	PENK	proenkephal		GO:0099538	synaptic signaling via neuropeptide (GO:0099538)	BP	975	
56	MYO5B	HGNC:7603	MYO5B	myosin VB	KIAA1119	GO:0098871	postsynaptic actin cytoskeleton (GO:0098871)	CC	985	
57	NEFM	HGNC:7734	NEFM	neurofilamer	NFM,NF-M,NEF3	GO:0099160	postsynaptic intermediate filament cytoskeleton (GO:0099160)	CC	997	
58	NEFM	HGNC:7734	NEFM	neurofilamer	NFM,NF-M,NEF3	GO:0099182	presynaptic intermediate filament cytoskeleton (GO:0099182)	CC	1002	
59	CADM1	HGNC:5951	CADM1	cell adhesion	NECL2,ST17,BL2,SYNCAM,IGSF4A,NECL-2,SYNCAM1,RA175,TSLC1,IGSF4	GO:0099560	synapse adhesion between pre- and post-synapse (GO:0099560)	BP	1016	
60	CADM1	HGNC:5951	CADM1	cell adhesion	NECL2,ST17,BL2,SYNCAM,IGSF4A,NECL-2,SYNCAM1,RA175,TSLC1,IGSF4	GO:0099054	presynapse assembly (GO:0099054)	BP	1018	
61	CADM1	HGNC:5951	CADM1	cell adhesion	NECL2,ST17,BL2,SYNCAM,IGSF4A,NECL-2,SYNCAM1,RA175,TSLC1,IGSF4	GO:0098942	retrograde trans-synaptic signaling by trans-synaptic protein complex	BP	1019	
62	PPFIA2	HGNC:9246	PPFIA2	PTPRF intera		GO:0099054	presynapse assembly (GO:0099054)	BP	1037	
63	CDH9	HGNC:1768	CDH9	cadherin 9		GO:0099560	synapse adhesion between pre- and post-synapse (GO:0099560)	BP	1068	
64	PCDH17	HGNC:14267	PCDH17	protocadheri	PCDH68,PCH68	GO:0099056	integral component of presynaptic membrane (GO:0099056)	CC	1084	
65	SYNGR1	HGNC:11498	SYNGR1	synaptogyrin		GO:0030285	integral component of synaptic vesicle membrane (GO:0030285)	CC	1164	
66	SYNGR1	HGNC:11498	SYNGR1	synaptogyrin		GO:0098693	regulation of synaptic vesicle cycle (GO:0098693)	BP	1165	
67	SV2C	HGNC:30670	SV2C	synaptic vesi		GO:2000300	regulation of synaptic vesicle exocytosis (GO:2000300)	BP	1207	
68	SV2C	HGNC:30670	SV2C	synaptic vesi		GO:0030285	integral component of synaptic vesicle membrane (GO:0030285)	CC	1236	
69	GABRA5	HGNC:4079	GABRA5	gamma-amin		GO:0099056	integral component of presynaptic membrane (GO:0099056)	CC	1263	
70	GABRA5	HGNC:4079	GABRA5	gamma-amin		GO:0099060	integral component of postsynaptic specialization membrane (GO:009	CC	1264	
71	GABRA2	HGNC:4076	GABRA2	gamma-amin		GO:0099059	integral component of presynaptic active zone membrane (GO:00990	CC	1266	
72	GABRA2	HGNC:4076	GABRA2	gamma-amin		GO:0099060	integral component of postsynaptic specialization membrane (GO:009	CC	1267	
73	NEFL	HGNC:7739	NEFL	neurofilamer	NFL,CMT1F,CMT2E,NF68,PPP1R110	GO:0099160	postsynaptic intermediate filament cytoskeleton (GO:0099160)	CC	1288	
74	HOMER3	HGNC:17514	HOMER3	homer scaffo	HOMER-3	GO:0098962	regulation of postsynaptic neurotransmitter receptor activity (GO:009	BP	1304	
75	CDH9	HGNC:1768	CDH9	cadherin 9		GO:0007416	synapse assembly (GO:0007416)	BP	1311	
76	CDH9	HGNC:1768	CDH9	cadherin 9		GO:0099056	integral component of presynaptic membrane (GO:0099056)	CC	1312	
77	CDH9	HGNC:1768	CDH9	cadherin 9		GO:0099055	integral component of postsynaptic membrane (GO:0099055)	CC	1313	
78	PCDH17	HGNC:14267	PCDH17	protocadheri	PCDH68,PCH68	GO:0099055	integral component of postsynaptic membrane (GO:0099055)	CC	1323	
79	GABRA2	HGNC:4076	GABRA2	gamma-amin		GO:0099507	ligand-gated ion channel activity involved in regulation of presynaptic	BP	1326	
80	GABRA5	HGNC:4079	GABRA5	gamma-amin		GO:0099507	ligand-gated ion channel activity involved in regulation of presynaptic	BP	1331	
81	GABRA2	HGNC:4076	GABRA2	gamma-amin		GO:1904315	transmitter-gated ion channel activity involved in regulation of postsy	BP	1332	
82	CABP1	HGNC:1384	CABP1	calcium bind		GO:0099527	postsynapse to nucleus signaling pathway (GO:0099527)	BP	1491	
83	CALB1	HGNC:1434	CALB1	calbindin 1	CALB	GO:0098794	postsynapse (GO:0098794)	CC	1624	
84	PLEKHA5	HGNC:30036	PLEKHA5	pleckstrin ho	PEPP2,KIAA1686,FLJ10667	GO:0014069	postsynaptic density (GO:0014069)	CC	1665	
85	KALRN	HGNC:4814	KALRN	kalirin RhoGE	DUO,HS.8004,TRAD,DUET,KALIRIN,ARHGEF24,HAPIP	GO:0098793	presynapse (GO:0098793)	CC	1669	
86	TUBB2B	HGNC:30829	TUBB2B	tubulin beta	MGC8685,DKFZP566F223,BA506K6.1	GO:0050804	modulation of chemical synaptic transmission (GO:0050804)	BP	1686	
87	NEFL	HGNC:7739	NEFL	neurofilamer	NFL,CMT1F,CMT2E,NF68,PPP1R110	GO:0099182	presynaptic intermediate filament cytoskeleton (GO:0099182)	CC	1687	
88	NEFL	HGNC:7739	NEFL	neurofilamer	NFL,CMT1F,CMT2E,NF68,PPP1R110	GO:0090128	regulation of synapse maturation (GO:0090128)	BP	1688	
89	SH3GL2	HGNC:10831	SH3GL2	SH3 domain	SH3P4,SH3D2A,CNSA2,EEN-B1	GO:0045202	synapse (GO:0045202)	CC	1701	
90	PCDH17	HGNC:14267	PCDH17	protocadheri	PCDH68,PCH68	GO:0099560	synapse adhesion between pre- and post-synapse (GO:0099560)	BP	1740	
91	GRASP	HGNC:18707	TAMALIN	trafficking re	GRASP	GO:0099152	regulation of neurotransmitter receptor transport, endosome to posts	BP	1744	
92	AKAP9	HGNC:379	AKAP9	A-kinase anc	KIAA0803,AKAP350,AKAP450,CG-NAP,YOTIAO,HYPERION,PRKA9,MU-RMS-40.16A,PPP1R45,LQT11	GO:0098962	regulation of postsynaptic neurotransmitter receptor activity (GO:009	BP	1746	
93	CADM1	HGNC:5951	CADM1	cell adhesion	NECL2,ST17,BL2,SYNCAM,IGSF4A,NECL-2,SYNCAM1,RA175,TSLC1,IGSF4	GO:0007416	synapse assembly (GO:0007416)	BP	1753	
94	NPTX2	HGNC:7953	NPTX2	neuronal per		GO:0098962	regulation of postsynaptic neurotransmitter receptor activity (GO:009	BP	1769	
95	SRC	HGNC:11283	SRC	SRC proto-on	ASV,C-SRC,SRC1	GO:0099091	postsynaptic specialization, intracellular component (GO:0099091)	CC	1779	
96	SRC	HGNC:11283	SRC	SRC proto-on	ASV,C-SRC,SRC1	GO:0098962	regulation of postsynaptic neurotransmitter receptor activity (GO:009	BP	1784	
97	HPCA	HGNC:5144	HPCA	hippocalcin	DYT2	GO:0099149	regulation of postsynaptic neurotransmitter receptor endocytosis (GO	BP	1785	
98	HPCA	HGNC:5144	HPCA	hippocalcin	DYT2	GO:0098794	postsynapse (GO:0098794)	CC	1786	
99	MYO5B	HGNC:7603	MYO5B	myosin VB	KIAA1119	GO:0098944	postsynaptic recycling endosome membrane (GO:0098944)	CC	1804	
100	MYO5B	HGNC:7603	MYO5B	myosin VB	KIAA1119	GO:0099090	synaptic vesicle recycling via endosome (GO:0036466)	BP	1805	
101	MYO5B	HGNC:7603	MYO5B	myosin VB	KIAA1119	GO:0099159	regulation of modification of postsynaptic structure (GO:0099159)	BP	1806	
102	MYO5B	HGNC:7603	MYO5B	myosin VB	KIAA1119	GO:0050804	modulation of chemical synaptic transmission (GO:0050804)	BP	1807	
103	MYO5B	HGNC:7603	MYO5B	myosin VB	KIAA1119	GO:0099639	neurotransmitter receptor transport, endosome to plasma membrane	BP	1808	
104	PTPRD	HGNC:9668	PTPRD	protein tyros	PTPD,HPTP	GO:0099545	trans-synaptic signaling by trans-synaptic complex (GO:0099545)	BP	1825	
105	PTPRD	HGNC:9668	PTPRD	protein tyros	PTPD,HPTP	GO:0099054	presynapse assembly (GO:0099054)	BP	1826	
106	PTPRD	HGNC:9668	PTPRD	protein tyros	PTPD,HPTP	GO:0099151	regulation of postsynaptic density assembly (GO:0099151)	BP	1829	
107	NTRK3	HGNC:8033	NTRK3	neurotrophic	TRKC	GO:0099055	integral component of postsynaptic membrane (GO:0099055)	CC	1842	
108	NTRK3	HGNC:8033	NTRK3	neurotrophic	TRKC	GO:0097107	postsynaptic density assembly (GO:0097107)	BP	1844	
109	NTRK3	HGNC:8033	NTRK3	neurotrophic	TRKC	GO:1905606	regulation of presynapse assembly (GO:1905606)	BP	1845	
110	SH3GL2	HGNC:10831	SH3GL2	SH3 domain	SH3P4,SH3D2A,CNSA2,EEN-B1	GO:0099523	presynaptic cytosol (GO:0099523)	CC	2002	
111	GABRA2	HGNC:4076	GABRA2	gamma-amin		GO:0099060	integral component of postsynaptic specialization membrane (GO:009	CC	2058	
112	GABRA5	HGNC:4079	GABRA5	gamma-amin		GO:0099055	integral component of postsynaptic membrane (GO:0099055)	CC	2083	
113	BDNF	HGNC:1033	BDNF	brain derivec		GO:0099183	trans-synaptic signaling by BDNF, modulating synaptic transmission (G	BP	2090	
114	KALRN	HGNC:4814	KALRN	kalirin RhoGE	DUO,HS.8004,TRAD,DUET,KALIRIN,ARHGEF24,HAPIP	GO:0098696	regulation of neurotransmitter receptor localization to postsynaptic sp	BP	2101	
115	SCG2	HGNC:10575	SCG2	secretogranii	CHGC,SGII,SN	GO:0098992	neuronal dense core vesicle (GO:0098992)	CC	2132	
116	GABRA5	HGNC:4079	GABRA5	gamma-amin		GO:0099060	integral component of postsynaptic specialization membrane (GO:009	CC	2169	
117	GABRA2	HGNC:4076	GABRA2	gamma-amin		GO:0099060	integral component of postsynaptic specialization membrane (GO:009	CC	2172	
118	GABRA5	HGNC:4079	GABRA5	gamma-amin		GO:1904315	transmitter-gated ion channel activity involved in regulation of postsy	BP	2179	
119	DGKB	HGNC:2850	DGKB	diacylglycero	KIAA0718,DGK,DGK-BETA,DAGK2	GO:0099147	extrinsic component of postsynaptic density membrane (GO:0099147	CC	2195	
120	NEFL	HGNC:7739	NEFL	neurofilamer	NFL,CMT1F,CMT2E,NF68,PPP1R110	GO:0099184	structural constituent of postsynaptic intermediate filament cytoskele	BP	2202	
121	LRRC4C	HGNC:29317	LRRC4C	leucine rich r	KIAA1580,NGL-1	GO:0099061	integral component of postsynaptic density membrane (GO:0099061)	CC	2221	
122	KALRN	HGNC:4814	KALRN	kalirin RhoGE	DUO,HS.8004,TRAD,DUET,KALIRIN,ARHGEF24,HAPIP	GO:1905274	regulation of modification of postsynaptic actin cytoskeleton (GO:190	BP	2237	
123	KALRN	HGNC:4814	KALRN	kalirin RhoGE	DUO,HS.8004,TRAD,DUET,KALIRIN,ARHGEF24,HAPIP	GO:0014069	postsynaptic density (GO:0014069)	CC	2238	
124	PTPRD	HGNC:9668	PTPRD	protein tyros	PTPD,HPTP	GO:0099056	integral component of presynaptic membrane (GO:0099056)	CC	2248	
125	GABRA2	HGNC:4076	GABRA2	gamma-amin		GO:0099060	integral component of postsynaptic specialization membrane (GO:009	CC	2264	
126	CALB1	HGNC:1434	CALB1	calbindin 1	CALB	GO:0099567	calcium ion binding involved in regulation of postsynaptic cytosolic ca	BP	2289	
127	PLS3	HGNC:9091	PLS3	plastin 3	T-PLASTIN	GO:0098699	structural constituent of presynaptic actin cytoskeleton (GO:0098699)	BP	2295	
128	CDH11	HGNC:1750	CDH11	cadherin 11	OB,CAD11	GO:0045202	synapse (GO:0045202)	CC	2318	
129	CDH11	HGNC:1750	CDH11	cadherin 11	OB,CAD11	GO:0050804	modulation of chemical synaptic transmission (GO:0050804)	BP	2319	
130	NTNG1	HGNC:23319	NTNG1	netrin G1	KIAA0976,LMNT1	GO:0099029	anchored component of presynaptic active zone membrane (GO:0099	CC	2321	
131	PTPRD	HGNC:9668	PTPRD	protein tyros	PTPD,HPTP	GO:0099151	regulation of postsynaptic density assembly (GO:0099151)	BP	2356	
132	TNR	HGNC:11953	TNR	tenascin R		GO:0050804	modulation of chemical synaptic transmission (GO:0050804)	BP	2382	
133	LRRC4C	HGNC:29317	LRRC4C	leucine rich r	KIAA1580,NGL-1	GO:0050804	modulation of chemical synaptic transmission (GO:0050804)	BP	2403	
134	NTNG1	HGNC:23319	NTNG1	netrin G1	KIAA0976,LMNT1	GO:0050804	modulation of chemical synaptic transmission (GO:0050804)	BP	2404	
135	CACNB2	HGNC:1402	CACNB2	calcium volta	MYSB,CACNLB2	GO:0099626	voltage-gated calcium channel activity involved in regulation of presyr	BP	2420	
136	PLS3	HGNC:9091	PLS3	plastin 3	T-PLASTIN	GO:0098693	regulation of synaptic vesicle cycle (GO:0098693)	BP	2447	
137	PTPRD	HGNC:9668	PTPRD	protein tyros	PTPD,HPTP	GO:0099560	synapse adhesion between pre- and post-synapse (GO:0099560)	BP	2465	
138	PTPRD	HGNC:9668	PTPRD	protein tyros	PTPD,HPTP	GO:0099560	synapse adhesion between pre- and post-synapse (GO:0099560)	BP	2473	
139	PTPRD	HGNC:9668	PTPRD	protein tyros	PTPD,HPTP	GO:0099560	synapse adhesion between pre- and post-synapse (GO:0099560)	BP	2478	
140	DRD5	HGNC:3026	DRD5	dopamine re	DRD1B,DRD1L2	GO:0099061	integral component of postsynaptic density membrane (GO:0099061)	CC	2532	
141	DRD5	HGNC:3026	DRD5	dopamine re	DRD1B,DRD1L2	GO:0099056	integral component of presynaptic membrane (GO:0099056)	CC	2534	
142	DRD5	HGNC:3026	DRD5	dopamine re	DRD1B,DRD1L2	GO:0099072	regulation of postsynaptic membrane neurotransmitter receptor level	BP	2542	
143	NR3C2	HGNC:7979	NR3C2	nuclear recep	MR,MLR	GO:0099092	postsynaptic density, intracellular component (GO:0099092)	CC	2545	
144	NR3C2	HGNC:7979	NR3C2	nuclear recep	MR,MLR	GO:0098831	presynaptic active zone cytoplasmic component (GO:0098831)	CC	2547	

	A	B	C	D	E	F	G	H	I	J
145	NR3C2	HGNC:7979	NR3C2	nuclear recep	MR,MLR	GO:0099171	presynaptic modulation of chemical synaptic transmission (GO:00991	BP	2548	
146	NR3C2	HGNC:7979	NR3C2	nuclear recep	MR,MLR	GO:0060078	regulation of postsynaptic membrane potential (GO:0060078)	BP	2549	
147	DGKB	HGNC:2850	DGKB	diacylglycero	KIAA0718,DGK,DGK-BETA,DAGK2	GO:0050804	modulation of chemical synaptic transmission (GO:0050804)	BP	2597	
148	DGKB	HGNC:2850	DGKB	diacylglycero	KIAA0718,DGK,DGK-BETA,DAGK2	GO:0099175	regulation of postsynapse organization (GO:0099175)	BP	2598	
149	PTPRD	HGNC:9668	PTPRD	protein tyros	PTPD,HPTP	GO:0050804	modulation of chemical synaptic transmission (GO:0050804)	BP	2648	
150	GABRA2	HGNC:4076	GABRA2	gamma-amin		GO:0099060	integral component of postsynaptic specialization membrane (GO:009	CC	2649	
151	LRRC4C	HGNC:29317	LRRC4C	leucine rich r	KIAA1580,NGL-1	GO:0099560	synapse adhesion between pre- and post-synapse (GO:0099560)	BP	2678	
152	PPFIA2	HGNC:9246	PPFIA2	PTPRF intera		GO:0099523	presynaptic cytosol (GO:0099523)	CC	2735	
153	NTNG1	HGNC:23319	NTNG1	netrin G1	KIAA0976,LMNT1	GO:0099560	synapse adhesion between pre- and post-synapse (GO:0099560)	BP	2766	
154	ARHGAP39	HGNC:29351	ARHGAP39	Rho GTPase a	KIAA1688,VILSE,CRGAP	GO:0098794	postsynapse (GO:0098794)	CC	2816	
155	ARHGAP39	HGNC:29351	ARHGAP39	Rho GTPase a	KIAA1688,VILSE,CRGAP	GO:0099173	postsynapse organization (GO:0099173)	BP	2817	
156	RGS14	HGNC:9996	RGS14	regulator of G		GO:0050804	modulation of chemical synaptic transmission (GO:0050804)	BP	2864	
157	RGS14	HGNC:9996	RGS14	regulator of G		GO:0014069	postsynaptic density (GO:0014069)	CC	2865	
158	USP46	HGNC:20075	USP46	ubiquitin spe	FLJ12552	GO:0045202	synapse (GO:0045202)	CC	2868	
159	CABP1	HGNC:1384	CABP1	calcium bind		GO:0098885	modification of postsynaptic actin cytoskeleton (GO:0098885)	BP	2871	
160	SH3GL2	HGNC:10831	SH3GL2	SH3 domain	SH3P4,SH3D2A,CNSA2,EEN-B1	GO:0030672	synaptic vesicle membrane (GO:0030672)	CC	2898	
161	PACSIN2	HGNC:8571	PACSIN2	protein kinas	SDPII	GO:0050804	modulation of chemical synaptic transmission (GO:0050804)	BP	2915	
162	USP46	HGNC:20075	USP46	ubiquitin spe	FLJ12552	GO:0099149	regulation of postsynaptic neurotransmitter receptor endocytosis (GO	BP	2923	
163	CDH9	HGNC:1768	CDH9	cadherin 9		GO:0045202	synapse (GO:0045202)	CC	2935	
164	CDH9	HGNC:1768	CDH9	cadherin 9		GO:0099560	synapse adhesion between pre- and post-synapse (GO:0099560)	BP	2955	
165	SRCIN1	HGNC:29506	SRCIN1	SRC kinase si	SNIP,P140CAP,KIAA1684	GO:0051963	regulation of synapse assembly (GO:0051963)	BP	2958	
166	SH3GL2	HGNC:10831	SH3GL2	SH3 domain	SH3P4,SH3D2A,CNSA2,EEN-B1	GO:0098794	postsynapse (GO:0098794)	CC	2959	
167	SH3GL2	HGNC:10831	SH3GL2	SH3 domain	SH3P4,SH3D2A,CNSA2,EEN-B1	GO:0098974	postsynaptic actin cytoskeleton organization (GO:0098974)	BP	2960	
168	SRCIN1	HGNC:29506	SRCIN1	SRC kinase si	SNIP,P140CAP,KIAA1684	GO:0098974	postsynaptic actin cytoskeleton organization (GO:0098974)	BP	2961	
169	NPTX2	HGNC:7953	NPTX2	neuronal per		GO:0043083	synaptic cleft (GO:0043083)	CC	2963	
170	CPEB1	HGNC:21744	CPEB1	cytoplasmic p	FLJ13203,CPEB	GO:0099547	regulation of translation at synapse, modulating synaptic transmission	BP	3015	
171	CPEB1	HGNC:21744	CPEB1	cytoplasmic p	FLJ13203,CPEB	GO:0014069	postsynaptic density (GO:0014069)	CC	3016	
172	STAU2	HGNC:11371	STAU2	staufen doub	39K2	GO:0098964	anterograde dendritic transport of messenger ribonucleoprotein comp	BP	3029	
173	KCNAB2	HGNC:6229	KCNAB2	potassium vo	AKR6A5,KCNA2B,HKVBETA2.1,HKVBETA2.2	GO:0099061	integral component of postsynaptic density membrane (GO:0099061)	CC	3037	
174	BCL11A	HGNC:13221	BCL11A	BAF chromat	BCL11A-XL,BCL11A-L,BCL11A-S,CTIP1,HBFQTL5,ZNF856,SMARCM1,EVI9	GO:0098794	postsynapse (GO:0098794)	CC	3048	
175	DROSHA	HGNC:17904	DROSHA	drosha ribon	RNASE3L,ETOHI2,HSA242976,RN3,RNASEN	GO:0014069	postsynaptic density (GO:0014069)	CC	3051	
176	GAD1	HGNC:4092	GAD1	glutamate de	GAD	GO:0098793	presynapse (GO:0098793)	CC	3121	
177	GAD1	HGNC:4092	GAD1	glutamate de	GAD	GO:0042136	neurotransmitter biosynthetic process (GO:0042136)	BP	3122	
178	UBA52	HGNC:12458	UBA52	ubiquitin A-5	RPL40,CEP52,HUBCEP52,MGC57125,MGC126879,MGC126881,L40	SYNGO:posts	postsynaptic ribosome	CC	3203	
179	UBA52	HGNC:12458	UBA52	ubiquitin A-5	RPL40,CEP52,HUBCEP52,MGC57125,MGC126879,MGC126881,L40	GO:0140236	translation at presynapse (GO:0140236)	BP	3532	
180	UBA52	HGNC:12458	UBA52	ubiquitin A-5	RPL40,CEP52,HUBCEP52,MGC57125,MGC126879,MGC126881,L40	SYNGO:presy	presynaptic ribosome	CC	3581	
181	UBA52	HGNC:12458	UBA52	ubiquitin A-5	RPL40,CEP52,HUBCEP52,MGC57125,MGC126879,MGC126881,L40	GO:0140242	translation at postsynapse (GO:0140242)	BP	3631	
182	RIMBP2	HGNC:30339	RIMBP2	RIMS binding	KIAA0318,RBP2,MGC15831,RIM-BP2,PPP1R133	GO:0150037	regulation of calcium-dependent activation of synaptic vesicle fusion (	BP	3654	
183	CPEB1	HGNC:21744	CPEB1	cytoplasmic p	FLJ13203,CPEB	GO:0050804	modulation of chemical synaptic transmission (GO:0050804)	BP	3664	
184	MYO5B	HGNC:7603	MYO5B	myosin VB	KIAA1119	GO:0098794	postsynapse (GO:0098794)	CC	3672	
185	NEFL	HGNC:7739	NEFL	neurofilamer	NFL,CMT1F,CMT2E,NF68,PPP1R110	GO:0099170	postsynaptic modulation of chemical synaptic transmission (GO:00991	BP	3684	
186	RIMBP2	HGNC:30339	RIMBP2	RIMS binding	KIAA0318,RBP2,MGC15831,RIM-BP2,PPP1R133	GO:0099626	voltage-gated calcium channel activity involved in regulation of presyn	BP	3739	
187	BTBD9	HGNC:21228	BTBD9	BTB domain	KIAA1880,DJ322I12.1	GO:0050804	modulation of chemical synaptic transmission (GO:0050804)	BP	3787	
188	NPY1R	HGNC:7956	NPY1R	neuropeptide	NPYR	GO:0099509	regulation of presynaptic cytosolic calcium levels (GO:0099509)	BP	3802	
189	NPY1R	HGNC:7956	NPY1R	neuropeptide	NPYR	GO:0099056	integral component of presynaptic membrane (GO:0099056)	CC	3803	
190	NPY1R	HGNC:7956	NPY1R	neuropeptide	NPYR	GO:2000300	regulation of synaptic vesicle exocytosis (GO:2000300)	BP	3827	
191	CPEB1	HGNC:21744	CPEB1	cytoplasmic p	FLJ13203,CPEB	GO:0098794	postsynapse (GO:0098794)	CC	3867	
192	MAPK8	HGNC:6881	MAPK8	mitogen-acti	JNK,JNK1,SAPK1,PRKM8	GO:0050804	modulation of chemical synaptic transmission (GO:0050804)	BP	3949	
193	RIMBP2	HGNC:30339	RIMBP2	RIMS binding	KIAA0318,RBP2,MGC15831,RIM-BP2,PPP1R133	GO:0099508	voltage-gated ion channel activity involved in regulation of presynapti	BP	3956	
194	SYNGR1	HGNC:11498	SYNGR1	synaptogyrin		GO:0030285	integral component of synaptic vesicle membrane (GO:0030285)	CC	3966	
195	SYNGR1	HGNC:11498	SYNGR1	synaptogyrin		GO:0098693	regulation of synaptic vesicle cycle (GO:0098693)	BP	3967	
196	RHOB	HGNC:668	RHOB	ras homolog	RHOH6,MST081,ARH6,ARHB	GO:0045202	synapse (GO:0045202)	CC	3994	
197	RHOB	HGNC:668	RHOB	ras homolog	RHOH6,MST081,ARH6,ARHB	GO:0099159	regulation of modification of postsynaptic structure (GO:0099159)	BP	3995	
198	GRM2	HGNC:4594	GRM2	glutamate m	GPRC1B,MGLU2,MGLUR2	GO:0099171	presynaptic modulation of chemical synaptic transmission (GO:00991	BP	4014	
199	SCG2	HGNC:10575	SCG2	secretograni	CHGC,SGII,SN	GO:0098992	neuronal dense core vesicle (GO:0098992)	CC	4017	
200	NPY1R	HGNC:7956	NPY1R	neuropeptide	NPYR	GO:0098992	neuronal dense core vesicle (GO:0098992)	CC	4035	
201	NPY5R	HGNC:7958	NPY5R	neuropeptide	NPYR5	GO:0098793	presynapse (GO:0098793)	CC	4052	
202	NPY5R	HGNC:7958	NPY5R	neuropeptide	NPYR5	GO:0099538	synaptic signaling via neuropeptide (GO:0099538)	BP	4053	
203	NPY1R	HGNC:7956	NPY1R	neuropeptide	NPYR	GO:0099538	synaptic signaling via neuropeptide (GO:0099538)	BP	4055	
204	VPS29	HGNC:14340	VPS29	VPS29 retron	PEP11,DC7,DC15	GO:1900242	regulation of synaptic vesicle endocytosis (GO:1900242)	BP	4107	
205	RIMBP2	HGNC:30339	RIMBP2	RIMS binding	KIAA0318,RBP2,MGC15831,RIM-BP2,PPP1R133	GO:0099505	regulation of presynaptic membrane potential (GO:0099505)	BP	4141	
206	PPFIA2	HGNC:9246	PPFIA2	PTPRF intera		GO:0099523	presynaptic cytosol (GO:0099523)	CC	4172	
207	PPFIA2	HGNC:9246	PPFIA2	PTPRF intera		GO:0099572	postsynaptic specialization (GO:0099572)	CC	4175	
208	BDNF	HGNC:1033	BDNF	brain derived		GO:0098992	neuronal dense core vesicle (GO:0098992)	CC	4178	
209	PPFIA2	HGNC:9246	PPFIA2	PTPRF intera		GO:0042734	presynaptic membrane (GO:0042734)	CC	4184	
210	PPFIA2	HGNC:9246	PPFIA2	PTPRF intera		GO:0008021	synaptic vesicle (GO:0008021)	CC	4185	
211	PTPRD	HGNC:9668	PTPRD	protein tyros	PTPD,HPTP	GO:0099056	integral component of presynaptic membrane (GO:0099056)	CC	4204	
212	PTPRD	HGNC:9668	PTPRD	protein tyros	PTPD,HPTP	GO:0099537	trans-synaptic signaling (GO:0099537)	BP	4208	
213	TNR	HGNC:11953	TNR	tenascin R		GO:0098965	extracellular matrix of synaptic cleft (GO:0098965)	CC	4509	
214										
